# Citrate Synthase and OGDH as Potential Biomarkers of Atherosclerosis under Chronic Stress

**DOI:** 10.1155/2021/9957908

**Published:** 2021-09-08

**Authors:** Ling-bing Meng, Gai-feng Hu, Meng-jie Shan, Yuan-meng Zhang, Ze-mou Yu, Yun-qing Liu, Hong-xuan Xu, Li Wang, Tao Gong, De-ping Liu

**Affiliations:** ^1^Department of Cardiology, Beijing Hospital, National Center of Gerontology, Institute of Geriatric Medicine, Chinese Academy of Medical Sciences, No. 1 DaHua Road, Dong Dan, Beijing 100730, China; ^2^Graduate School, Chinese Academy of Medical Sciences & Peking Union Medical College, No. 9 Dongdansantiao, Dongcheng District, Beijing 100730, China; ^3^Department of Cardiology, The First Affiliated Hospital of Wenzhou Medical University, Nanbaixiang, Wenzhou 325000, China; ^4^Department of Plastic Surgery, Peking Union Medical College Hospital, Beijing 100730, China; ^5^Department of Internal Medicine, the Third Medical Centre of Chinese PLA General Hospital, The Training Site for Postgraduate of Jinzhou Medical University, Beijing, China; ^6^Department of Neurology, Beijing Children's Hospital, No. 56 Nanlishi Road, Xicheng District, Beijing 100045, China; ^7^Department of Neurology, Beijing Hospital, National Center of Gerontology, National Center of Gerontology, Institute of Geriatric Medicine, Chinese Academy of Medical Sciences, No. 1 DaHua Road, Dong Dan, Beijing 100730, China

## Abstract

**Background:**

Pathological changes of the adrenal gland and the possible underlying molecular mechanisms are currently unclear in the case of atherosclerosis (AS) combined with chronic stress (CS).

**Methods:**

New Zealand white rabbits were used to construct a CS and AS animal model. Proteomics and bioinformatics were employed to identify hub proteins in the adrenal gland related to CS and AS. Hub proteins were detected using immunohistochemistry, immunofluorescence assays, and Western blotting. Real-time quantitative polymerase chain reaction (RT-qPCR) was used to analyze the expression of genes. In addition, a neural network model was constructed. The quantitative relationships were inferred by cubic spline interpolation. Enzymatic activity of mitochondrial citrate synthase and OGDH was detected by the enzymatic assay kit. Function of citrate synthase and OGDH with knockdown experiments in the adrenal cell lines was performed. Furthermore, target genes-TF-miRNA regulatory network was constructed. Coimmunoprecipitation (IP) assay and molecular docking study were used to detect the interaction between citrate synthase and OGDH.

**Results:**

Two most significant hub proteins (citrate synthase and OGDH) that were related to CS and AS were identified in the adrenal gland using numerous bioinformatic methods. The hub proteins were mainly enriched in mitochondrial proton transport ATP synthase complex, ATPase activation, and the AMPK signaling pathway. Compared with the control group, the adrenal glands were larger and more disordered, irregular, and necrotic in the AS+CS group. The expression of citrate synthase and OGDH was higher in the AS+CS group than in the control group, both at the protein and mRNA levels (*P* < 0.05). There were strong correlations among the cross-sectional areas of adrenal glands, citrate synthase, and OGDH (*P* < 0.05) via Spearman's rho analysis, receiver operating characteristic curves, a neural network model, and cubic spline interpolation. Enzymatic activity of citrate synthase and OGDH increased under the situation of atherosclerosis and chronic stress. Through the CCK8 assay, the adrenal cell viability was downregulated significantly after the knockdown experiment of citrate synthase and OGDH. Target genes-TF-miRNA regulatory network presented the close interrelations among the predicted microRNA, citrate synthase and OGDH. After Coimmunoprecipitation (IP) assay, the result manifested that the citrate synthase and OGDH were coexpressed in the adrenal gland. The molecular docking study showed that the docking score of optimal complex conformation between citrate synthase and OGDH was -6.15 kcal/mol.

**Conclusion:**

AS combined with CS plays a significant role on the hypothalamic–pituitary–adrenal (HPA) axis, promotes adrenomegaly, increases the release of glucocorticoid (GC), and might enhance ATP synthesis and energy metabolism in the body through citrate synthase and OGDH gene targets, providing a potential research direction for future related explorations into this mechanism.

## 1. Introduction

Atherosclerosis (AS) is a chronic progressive disease characterized by the accumulation and deposition of lipid and fibrous components and an inflammatory response that leads to the development of lesions; AS is the most common and important member of a group of vascular diseases collectively called arteriosclerosis [1]. AS is the main cause of coronary heart disease, cerebral infarction, and peripheral vascular disease, and its incidence has increased in recent years, seriously threatening human life and health [2, 3]. AS is characterized by lesions of the affected artery, starting from the intima and followed by a combination of various lesions, including local lipid and complex carbohydrate accumulation, fibrous hyperplasia, and calcinosis, which form plaques that lead to a gradual degeneration of the arterial media, followed by secondary signs including intraplaque hemorrhage, plaque rupture, and local thrombosis “or” followed by secondary lesions and other signs including intraplaque hemorrhage, plaque rupture, and local thrombosis [4]. The etiology of AS has not yet been fully determined, although studies have shown that this disease can be caused by a variety of factors acting on different pathways. The main risk factors include age, sex, dyslipidemia, hypertension, smoking, diabetes and impaired glucose tolerance, chronic stress, obesity, and a family history of AS.

Chronic stress (CS) is a nonspecific systemic response that occurs when the body is stimulated by psychological, environmental, or physiological stressors over a long period of time [5]. Physiological responses to CS have long been recognized as effective regulators of the development of AS. Studies have shown that chronic psychological stress increases the risk of developing atherosclerotic diseases, including stroke and coronary heart disease [6]. CS can activate various stress pathways in the body [7], including the hypothalamic–pituitary–adrenal (HPA) axis and the sympathetic nervous system (SNS). This can have adverse effects on the cardiovascular, endocrine, and immune systems, leading to the occurrence and development of stress-related diseases and threatening human health [8, 9]. However, the pathological changes that occur in the adrenal gland and the possible molecular mechanisms involved in this are currently unclear in the case of AS combined with CS.

Bioinformatics is the science of storing, retrieving, and analyzing biological information using computers as tools in the study of life sciences. With the continuous development of biomedical research technologies, especially sequencing technologies and bioinformatics algorithms, increasing quantities of genomics information are exponentially accumulating [10, 11].

Citrate synthase [12] and oxoglutarate dehydrogenase (OGDH) [13] catalyze the condensation of acetyl-CoA from glycolysis or other dissimilatory reactions with oxaloacetate to synthesize citric acid. They are the rate-limiting enzymes in the mitochondrial tricarboxylic acid (TCA) cycle and participate in many important biochemical processes. At present, the relationship between pathological changes in the adrenal gland and these two molecules under conditions of AS combined with CS is not clear.

This research was conducted to study the changes in the adrenal glands under AS combined with CS and uncover the core genes involved in these changes via enrichment analysis, pathway analysis, construction of a neural network model, and verification via animal experiments.

## 2. Materials and Methods

### 2.1. Animals and Groups

A total of 50 New Zealand white rabbits (3 months old, 3.0 ± 0.2 kg) were obtained from the Institute of Laboratory Animal Sciences, Chinese Academy of Medical Sciences (CAMS) & Peking Union Medical College (PUMC). The rabbits were randomly divided into four groups: normal group (*n* = 10), CS group (*n* = 10), AS group (*n* = 15), and AS+CS group (*n* = 15). These animals could freely obtain food and water in their own cage (temperature 25 ± 2°C, relative humidity 60% ± 2%). An animal ethical agreement was granted by the Animal Care and Use Committee of CAMS&PUMC.

### 2.2. Construction of the CS Model

Construction of the CS model mainly included subjecting animals to unstable social and physical stress for two months. Changing partners of rabbits was the main method used to induce social stress. The methods used to generate physical stress comprised white noise (80 dB), overnight illumination, tilting the cage, foot shock (1 mA), and stroboscopic illumination, as described in a previous publication [14, 15]. In addition, the detailed information of construction of CS model and the assessment of CS animal model were presented in the Supplement 1.

### 2.3. Construction of the as Model

Prior to surgery, rabbits in the AS and AS+CS groups were narcotized with 3% sodium pentobarbital solution (3 mg/kg of animal body weight; provided by CAMS&PUMC, Beijing, China) via the marginal ear vein. A longitudinal incision was performed in the right groin and the femoral artery was isolated. A 3 mm × 20 mm balloon catheter was advanced into the abdominal artery, inflated with 10 atm and then gently denuded three times. The procedures were performed under sterile conditions. Before being placed in their cages, the animals were allowed to recover under observation. In the first five days after surgery, benzylpenicillin sodium was administered by intramuscular injection at a dose of 40000 U per day. The animals were provided with a normal chow diet for 4 weeks, followed by a high-fat diet (6% bean oil and 1% cholesterol) for 8 weeks [15, 16].

### 2.4. Obtaining Tissues

After the experiment of establishing animal model, the animals were narcotized with 3% pentobarbital solution (300 mg/kg of body weight), and air embolization was performed. The death of the rabbits was judged by cardiac and breathing arrest.

The whole abdominal aorta was removed and then evenly cut into four segments. Two of these parts (near iliac artery) were embedded in the Tissue-Tek OCT compound (Sakura, Tokyo, Japan) for frozen sectioning, and the others were snap-frozen in liquid nitrogen for molecular experiments.

The bilateral adrenal glands were removed. The half part of fresh left adrenal glands could be used to measure the enzymatic activity of mitochondrial citrate synthase and OGDH. And the other part of fresh left adrenal glands would be embedded in the Tissue-Tek OCT compound (Sakura, Tokyo, Japan) for frozen sectioning, hematoxylin-eosin (HE) staining, immunohistochemistry, and immunofluorescence assays. The right adrenal glands were snap-frozen in liquid nitrogen for molecular experiments, including RT-qPCR, Western blotting, and coimmunoprecipitation (IP) assay.

General data of the animals about body weight, food intake, biochemistry, inflammation, and blood morphology was presented in the Supplement 2.

### 2.5. Isobaric Tags or Relative and Absolute Quantitation (iTRAQ) Proteomics

#### 2.5.1. Principle

The iTRAQ proteomic quantitative technique could use multiple (2-10) stable isotope tags to specifically label the amino groups of the peptides for tandem mass spectrometry analysis. It could compare the relative content of protein in different samples at the same time and could be used to study the difference of protein expression level in samples under different pathological conditions.

Three adrenal samples were randomly selected from each of the four groups. Next, protein extraction was performed using a vortex mixer (Haimen Qilin Bell Instrument Manufacturing Co. LTD, QL-901, Haimen, China) and an ultrasonic cell crusher (Nanjing Xianou Instrument Manufacturing Co. LTD, XO, Nanjing, China). The protein was reduced and sealed. After that, the protein was cut into peptides using trypsin, with an iTRAQ® kit (AB Sciex, PN: 4381664, CA, USA). The peptide mixtures in each group were labeled with different 8-plex iTRAQ reagents (AB Sciex, PN: 4381663, CA, USA). The labeled peptides in each sample were mixed in equal quantities. Liquid chromatography with tandem mass spectrometry (LC-MS/MS) detection and analysis was performed with Durashell-C18, 4.6 mm × 250 mm, 5 *μ*m, 100 Å (Agela, Item No: DC952505-0, Beijing, China). Protein analysis was carried out by Na-upgrade reversed-phase chromatography Q Exactive HF with a high-performance liquid chromatograph. The MS analysis of iTRAQ was performed using a Thermo Q Exactive HF-type mass spectrometer, and the original mass spectrometry files generated were processed by the supporting commercial software Proteome Discoverer 2.1 from Thermo.

### 2.6. Overall Analysis of Identification Results and Differentially Expressed Protein (DEP) Screening

Cluster analysis was performed on all proteins, which were presented in the form of a heatmap. After the median value was normalized, the quantitative values were further normalized using Perseuse (Max-Planck-Institute of Biochemistry, 2019). As the number of repetitions of samples was more than three, a paired *t*-test was used directly for difference analyses, with a *P* value ≤ 0.05 and a Log (fold change) ≥ 10 to identify the common hub DEPs.

### 2.7. The Protein–Protein Interaction (PPI) Network and Hub Proteins

The DEPs were imported into the Search Tool for the Retrieval of Interacting Genes (STRING; http://string-db.org) (version 10.5) [17], and the PPI network was generated when a medium confidence of >0.4 was set. Venn diagrams were used to identify common DEPs among different groups. PPI networks were visualized using Cytoscape (version 3.6.1) [18]. Molecular Complex Detection (MCODE) [19] (version 1.5.1) was used to discover tightly coupled regions in the PPI network, which could then be used to identify the most important module in the PPI network. The criteria for MCODE were as follows: MCODE score > 5, degree cut − off = 2, *k* − score = 2, maximum depth = 100, and node score cut − off = 0.2. When the degree ≥ 10, hub proteins were identified. The MCC and EPC algorithms were also used to screen the hub proteins.

### 2.8. Verification Using Metascape

Two lists of DEPs (DEPs between the CS and normal groups and DEPs between the AS+CS and AS groups) were entered into Metascape for further analyses [20]. The overlaps between these lists were shown by Circos plots [21]. In particular, pie charts were used to visualize whether terms were shared by multiple lists and to understand how the terms related to each other. To further explore the associations between terms, enriched terms were chosen and presented as a network, where these terms had a similarity > 0.3. Two ontology categories (DisGeNET [22] and PaGenBase [23]) were used to identify the gene list enrichments. The criteria for enrichment were a *P* value < 0.01, an enrichment factor > 1.5, and a minimum count of 3.

### 2.9. Weighted Gene Coexpression Network Analysis (WGCNA)

WGCNA can be used to explore coexpressed gene modules and analyze associations between gene networks. In addition, it can be used to find the relationships between modules and traits and further screen hub nodes in a network. The detailed process involved the following. First, the integrity of the data was checked. Second, a value was selected to establish an adjacent matrix according to the degree of connectivity, so that our gene distribution conformed to a scale-free network. Third, a topological matrix was used to cluster the genes with by degrees of difference, and the gene cluster tree was cut into different modules using the dynamic shear method. Fourth, correlation analysis between module and sample traits was performed. Fifth, topologically overlapping heatmaps were drawn using randomly selected genes. Sixth, the hub genes were verified by the WGCNA.

### 2.10. Enrichment Analysis

Enrichment analysis can be used to find a class of overexpressed genes or proteins in a group of genes or proteins, so it is easier to find valuable functional categories or pathways among a list of proteins identified by omics. Based on a hypergeometric distribution statistical significance test, function enrichment analysis used overrepresentation analysis to calculate the response difference identification or protein in one Gene Ontology (GO) functional category or Kyoto Encyclopedia of Genes and Genomes (KEGG) pathway if there was a significant degree of enrichment based on *P* values, and false discovery rates (FDR) correction. The smaller the *P* value or FDR value, the higher the enrichment value, and the higher the degree of enrichment.

### 2.11. Identification of Hub Proteins Associated with Adrenal Cortex Diseases.

The Comparative Toxicogenomics Database (CTD; http://ctdbase.org/) provides information about interactions between genes or proteins and diseases. The relationships between adrenal cortex diseases and hub proteins were analyzed using the CTD.

### 2.12. HE Staining

#### 2.12.1. Principle

Hematoxylin dye is alkaline, mainly making the chromatin in the nucleus and the ribosome in the cytoplasm purple blue. Eosin is an acid dye, mainly making the components of the cytoplasm and extracellular matrix red. HE staining is the most basic technique in histology, embryology, and pathology.

HE staining stains nuclei a bluish-purple and the cytoplasm red. HE staining was used to perform a general examination of the adrenal glands. The specific steps were as follows. First, the tissue sections were dried at room temperature for 20 minutes. Second, hematoxylin dye was added for 3 minutes. Third, 0.5% hydrochloric acid alcohol was added for a few seconds to differentiate the samples. Fourth, eosin was added for 30 minutes, dehydrated with gradient alcohol, and then any unstained sample was rendered transparent using xylene. The specimens were observed under a stereoscopic microscope (Axio Zoom V16, ZEISS, Germany).

### 2.13. Immunohistochemical and Immunofluorescence Assay

#### 2.13.1. Principle

Immunohistochemical technique based on the basic principle of antigen-antibody reaction, namely the specific combination of antigen and antibody, was performed. Through the chemical reaction, antibody-labeled chromogenic agents (luciferin, enzymes, metal ions, and isotopes) could produce color to identify intracellular antigens (peptides and proteins) in tissues. Then the localization, qualitative, and quantitative studies were performed for these antigens. The principle of immunofluorescence assay was the same as the immunohistochemical technique. However, the antigen was fluorescently labeled in the immunofluorescence assay.

The adrenal tissue was paraffin-embedded and sectioned. Paraffin sections were dewaxed with water, sealed with hydrogen peroxide, and then washed with distilled water. The paraffin sections were further washed with phosphate-buffered saline (PBS, Servicebio, G1202, Wuhan, China, pH = 6.0). The slices were sealed with 10% goat serum (TransGen Biotech, Beijing, China) at 37°C for 20 minutes. The serum was removed with filter paper. A citrate synthase polyclonal antibody (dilution rate = 1 : 200, 16131-1-AP, ProteinTech, Rosemont, USA) was applied dropwise, and the samples were incubated overnight at 4°C. PBS was used to wash the samples three times (5 min/time), and then the samples were incubated with a second antibody (dilution rate = 1 : 5000) for 1 h at 37°C. Diaminobenzidine (Servicebio, G1211, Wuhan, China) was used for color development. Each paraffin section was photographed in six fields. The citrate synthase level was also detected using an immunofluorescence assay.

The next process, to detect the expression level of oxoglutarate dehydrogenase (OGDH), was similar to the above, but the first antibody used was OGDH polyclonal antibody (dilution rate = 1 : 200, 15212-1-AP, ProteinTech, Rosemont, USA). The OGDH level was also detected using an immunofluorescence assay.

### 2.14. Real-Time Quantitative Polymerase Chain Reaction (RT-qPCR)

#### 2.14.1. Principle

RT-qPCR was used to label and track the PCR products by fluorescent labeled probes, and the reaction process was monitored online in real time. Combined with the corresponding software, this assay could calculate the initial concentration of the sample. It could be used to detect the gene expression level of mRNA.

To isolate RNA, a power homogenizer was used to homogenize adrenal tissue samples in 1 mL of TRIZOL reagent (Servicebio, G3013, Wuhan, China) per 100 mg of adrenal tissue. The concentration and purity of RNA were determined using an ultramicrospectrophotometer (Nanodrop 2000, Thermo, Waltham, Massachusetts, USA). Reverse transcription used is as follows: template RNA, 2 *μ*g; Oligo (dT) 18 Primer 0.5 *μ*L and gene-specific primer 1 *μ*; water, nuclease-free to 15 *μ*L, total volume 15 *μ*L.  Table 1 shows the primer sequences used for CS and OGDH. The following components were added in the indicated order: 5X Reaction Buffer 4 *μ*L, Servicebio®RT Enzyme Mix^a^, 1 *μ*L (Servicebio, G3330, Wuhan, China), total volume, 20 *μ*L. PCR amplification was performed, and the results were processed with 2^-*ΔΔ*Ct^. Actin was used as an endogenous control.

### 2.15. Western Blotting

#### 2.15.1. Principle

Western blotting assay could separate proteins with different molecular weights by SDS-PAGE glue and then transferred the target protein to PVDF membrane. Based on the principle of specific binding of antigen and antibody, the specific primary antibody could bind the target protein, and then the HRP-labeled secondary antibody could bind the primary antibody, which could be colored by the ECL luminous fluid. Finally, this assay could detect the expression of certain proteins in tissues or cells.

The adrenal tissue blocks were washed two to three times with precooled PBS, and lysis buffer (Servicebio, G2002, Wuhan, China) was added to isolate the total protein. There were 50 to 100 *μ*g of protein added to each lane, and then 10% sodium dodecyl sulfate polyacrylamide gel electrophoresis (SDS-PAGE) was used for electrophoresis. Next, the protein was transferred from the gel to polyvinylidene fluoride (PVDF) membranes, and 5% skimmed milk was used to seal the membrane for 1 hour. Primary antibody was applied dropwise, and the samples were incubated overnight at 4°C. Horseradish peroxidase-labeled secondary antibody (1 : 2500) was added, and the samples were incubated for 2 h at room temperature.

GAPDH was used as an endogenous control. Mouse anti-rabbit GAPDH polyclonal antibody (dilution rate = 1 : 1000, GB12002, Servicebio, Wuhan, China) was used to detect GAPDH, and the secondary antibody was also a goat anti-mouse monoclonal antibody (dilution rate = 1 : 5000, ab205719, Abcam, Cambridge, UK). Citrate synthase protein was detected using a citrate synthase polyclonal antibody (dilution rate = 1 : 5000, 16131-1-AP, ProteinTech, Rosemont, USA). OGDH proteins were detected using an OGDH polyclonal antibody (dilution rate = 1 : 20000, 15212-1-AP, ProteinTech, Rosemont, USA). A secondary antibody was applied (dilution rate = 1 : 5000). The film was scanned with an enhanced chemiluminescence kit (Millipore, Billerica, Massachusetts, USA). Image-Pro Plus 6.0 (Media Cybernetics Inc., Chicago, USA) was used to analyze the optical density value of the target band.

### 2.16. Enzymatic Activity of Mitochondrial Citrate Synthase and OGDH

#### 2.16.1. Principle

The activity of mitochondrial citrate synthase was measured by enzymatic assay kit (Abcam, ab119692). The microplate assay ab119692 is used to determine mitochondrial citrate synthase (CS) activity in a sample at an immunocapture-based manner. The enzyme is captured within the wells of the microplate, and activity is determined by recording color development of TNB, which is generated from DTNB present in the reaction of citrate synthesis. The overall reaction product, TNB, absorbs at 412 nm.

The activity of OGDH was measured by the enzymatic assay kit (USBiological Life Science, 219370). The OGDH BioAssay™ kit provides a way for monitoring OGDH activity. In the assay, OGDH converts alpha-ketoglutarate into an intermediate which reduces the probe to a colored product with strong absorbance at 450 nm. The assay can detect OGDH activity lower than 0.1 mU in a variety of samples.

Fresh adrenal tissues were prepared as instructed. The protein concentration of extracts were determined. All reagents were brought to room temperature. The sample was diluted to desired protein concentration in incubation buffer, and 125 *μ*g proteins were used for this assay. 100 L sample was added to each well used. The plate was sealed in a plate shaker for all incubation steps at 300 rpm and incubated for 3 hours at room temperature. Aspirate and wash each well twice. Each well was wash and aspirated, and this was repeated once more for a total of two washes, washed by aspirating or decanting from wells then dispensing 300 *μ*L 1x wash buffer into each well as described above. Complete removal of liquid at each step is essential to good performance. After the last wash, the remaining buffer was removed by aspiration or decanting. The plate was inverted, and it was blotted against clean paper towels to remove excess liquid. 100 *μ*L freshly prepared 1x activity solution was gently added to each well minimizing the production of bubbles. The bubbles were popped, and the absorbance in the microplate reader with time at 412 nm (citrate synthase) or 450 nm (OGDH) for 15 to 30 minutes was recorded immediately. Based on the protocol, the enzyme activity (1 U) could be expressed as the change in absorbance per minute per amount of sample loaded into the well.

### 2.17. Function of Citrate Synthase and OGDH with Knockdown Experiments in the Adrenal Cell Lines

Adrenal cells were purchased from Zhongke Quality Inspection Co., Ltd. The medium condition was DMEM (Gibco, 12800 − 017) + 10%FBS (Excell Bio, FSP500) + 1%double antibody (Gibco, 15140 − 122). The citrate synthase siRNA and OGDH siRNA were compounded by Beijing Qingke Biotechnology Co., LTD. The expression level of citrate synthase and OGDH after intervention of siRNA by RT-qPCR was recorded. CCK-8 detection was performed to test the proliferation activity of adrenal cells after citrate synthase and OGDH with knockdown experiments. The experiment assay was followed by the specification of the CCK8 kit (C0038, BIYUNTIAN, China). After plating overnight, drugs were added. Then, the 96-well plate was placed in a 37°C 5% CO_2_ incubator (Thermo, 3111) to incubate. 10 *μ*L CCK-8 solution was added to each well and incubated for 1-4 hours in the cell incubator. Absorbance was detected at 450 nm with the microplate reader (Shanghai Ouying Experimental Equipment Co., Ltd, K3). Cell viability (%) = (experimental group OD − blank group OD)/(control group OD − blank group OD).

### 2.18. Construction and Analysis of Target Genes-TF-miRNA Regulatory Network

TFmiR (https://service.bioinformatik.uni-saarland.de/tfmir/) is a free web server for integrating and analyzing interactions between TF-miRNA-target genes. All three are related to the pathogenesis of the disease. The internal workings of the cell depend on the proper functioning of an extremely complex activation-suppression system, which may be perturbed in many ways. TFmiR is helpful to better elucidation of the cellular mechanism from the molecular level of the network. TFmiR provides topological properties and functional analysis, making it a credible systems biology tool for researchers in the life sciences.

### 2.19. Coimmunoprecipitation (IP) Assay to Detect the Interaction between Citrate Synthase and OGDH

#### 2.19.1. Principle

Coimmunoprecipitation assay could be used to study protein-protein interactions based on the specific binding between antibodies and antigens and be often used to verify whether there is interaction between two known proteins.

Adrenal tissues were washed 2-3 times with precooled PBS to remove blood stain, cut into small pieces, and placed in homogenate tube. 10 times IP lysate (add protease inhibitor within a few minutes before use) was added and homogenated by machine. The homogenate was transferred to a 1.5 mL centrifuge tube and cracked on ice for 30 min. The supernatant was collected after centrifugation (12000 rpm, 4°C for 10 min). And the protein concentration was determined by the BCA method. A small amount of supernatant was denatured for input experiment, that is, Western blotting was used to detect target protein.

Citrate synthase protein (molecular weight: 45-50 kD) was detected using a citrate synthase polyclonal antibody (dilution rate = 1 : 1000, 16131-1-AP, ProteinTech, Rosemont, USA). OGDH proteins (molecular weight : 110 kD) were detected using an OGDH polyclonal antibody (dilution rate = 1 : 1000, #26865, CST). A secondary antibody was applied (mouse anti-rabbit IgG light chain specific, HRP conjugate, catalog number: SA00001-7L, dilution rate = 1 : 3000).

1.0 *μ*g IgG and 20 *μ*L Protein A/G beads were into the negative control (IgG) group supernatant (mix well before use). 20 *μ*L Protein A/G beads were added directly into the experimental group, shaken, and incubated at 4°C for 1 h. The supernatant was extracted after centrifugation at 4°C for 5 min at 2000 rpm. 1-10 *μ*L (0.2-2 *μ*g) antibody was added and incubated overnight at 4°C. 80 *μ*L Protein A/G beads (well mixed before use) was added and incubated at 4°C for 2 h. After centrifugation at 4°C for 5 min at 2000 rpm, the supernatant was carefully aspirated, and the immunoprecipitation complex was collected. The immunoprecipitation complex was washed 4 times with 1 mL precooled IP lysate (without adding various inhibitors) and centrifuged at 4°C, 2000 rpm, for 5 min, and the supernatant carefully discarded after each washing. After the last washing, supernatant was absorbed as much as possible. Then, 80 *μ*L 1× reduced sample loading buffer was added and boiled for 10 min. The supernatant was centrifuged at 1000 rpm at 4°C for 5 min. 10 *μ*L supernatant sample was taken for Western blotting test.

### 2.20. Docking Study of OGDH and Citrate Synthase

The software PyMol 2.3.4 (DeLano Scientific LLC, https://pymol.org/2/) was used to dehydrate and remove ligand for the receptor protein (OGDH). The MGLTools software (Molecular Graphics Laboratory, The Scripps Research Institute, http://mgltools.scripps.edu/) was used to hydrogenate the receptor protein and calculate the charge. The appropriate box center and box grid point parameters were set to include the active pocket sites that the ligand (citrate synthase) might bind to. AutoDock Vina 1.5.6 (http://vina.scripps.edu/) was used for molecular docking of receptor proteins. Twenty conformations were obtained by the docking study, and the complex conformation with the best docking score was visualized using PyMol 2.3.4.

### 2.21. Statistics

All assays in the research were replicated three times. Statistical analyses were performed using the SPSS software (version 21.0; IBM Corporation, New York, USA). A *P* value < 0.05 was considered statistically significant. Sample size calculation was performed by the PASS15.0 software, and the detailed information was presented in the Supplement 3.

Quantitative variables are presented as the mean ± standard deviation (SD) or standard error of mean (SEM), and normality was tested using the Shapiro–Wilk test. Qualitative variables are presented as absolute frequencies and percentages. Statistical significance was determined using Student's *t*-test when two groups were compared or by ANOVA and a post hoc two-tailed Newman-Keuls test when three or more groups were compared. The homogeneity of variance was checked by the Levene test. In the Levene test, *P* ≤ 0.05 represents that data accorded with homogeneity of variance test, and *P* > 0.05 represents that data were not in accordance with the homogeneity of variance test. When data accorded with homogeneity of variance test, LSD(L) was used. When data were not in accordance with the homogeneity of variance test, Tamhane's T2 multiple comparison test was used. Spearman's rho test was used to analyze correlations among citrate synthase, OGDH, and the cross-sectional area of adrenal glands. In addition, we constructed receiver operating characteristic (ROC) curves and applied the area under the curve (AUC) to assess the accuracy and sensitivity of each factor in diagnosing the cross-sectional area of the adrenal glands. A neural network model was constructed using citrate synthase, OGDH, and the cross-sectional area of the adrenal glands to explore the value of citrate synthase and OGDH for predicting the cross-sectional area of the adrenal glands. Finally, quantitative relationships among citrate synthase, OGDH, and the cross-sectional area of the adrenal glands were inferred by cubic spline interpolation.

## 3. Results

### 3.1. Screening of DEPs

**Figure 1:**
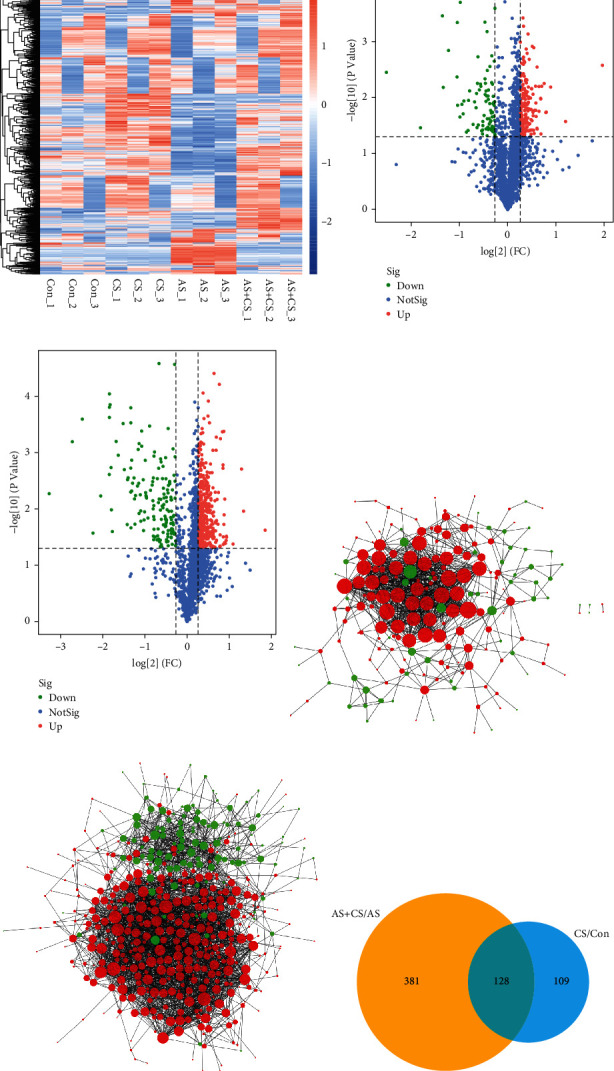
Screening of differentially expressed proteins (DEPs). (a) The heatmap shows all proteins. (b) DEPs between the control (Con) and chronic stress (CS) group. (c) DEPs between atherosclerosis+chronic stress (AS+CS) and atherosclerosis (AS). (d) The protein-protein interact (PPI) network between the Con and CS groups. (e) The PPI network between the AS+CS and AS groups. (f) A Venn diagram showing common DEPs.

All of the proteins were clustered and displayed in a heatmap, which showed that numerous differences existed among the four groups (Figure 1(a)). One volcano plot shows the DEPs between the Con and CS groups (Figure 1(b)), and the other shows the DEPs between the AS+CS and AS groups (Figure 1(c)). Supplement 4 presented the Limma of full length of DEPs with probe ID, logFC, *P* value, adj. *P* value, *t* value, and gene name. And Supplement 5 manifested the protein ID_gene name of DEPs. There were close relationships among the DEPs between the Con and CS groups in the first PPI network (Figure 1(d)), and there were also close relationships among the DEPs between the AS+CS and AS groups in the second PPI network (Figure 1(e)). A total of 128 DEPs were simultaneously present in the two PPI networks, which were defined as common DEPs (Figure 1(f)).

### 3.2. Identification of Hub Proteins

**Figure 2:**
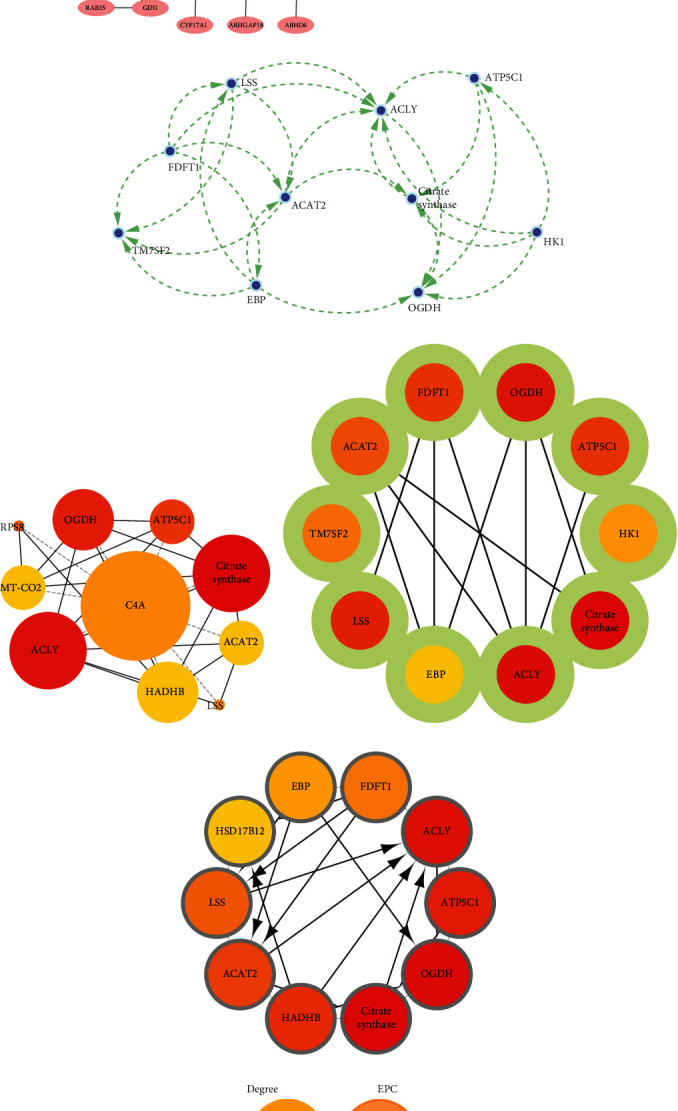
Identification of hub proteins. (a) The PPI network of common DEPs. (b) MCODE analysis. (c) Degree, (d) MCC, and (e) EPC algorithms were applied to identify hub proteins. (f) A total of six common hub proteins were obtained, as shown in the Venn diagram. (g) The heatmap shows the level of expression of the six common hub proteins.

The STRING online database was used to construct a PPI network of common DEPs, and the Cytoscape software was used to visualize this (Figure 2(a)). MCODE analysis was employed to detect significant modules within the PPI network, which is shown in Figure 2(b). Three different algorithms were applied to identify hub proteins. If the degree was ≥10, hub proteins could be identified (Figure 2(c)). In addition, ten hub proteins were highlighted via MCC (Figure 2(d)). The EPC algorithm was used to identify a further ten hub proteins (Figure 2(e)). A total of six common hub proteins was obtained from the Venn diagram, including LSS, ACAT2, ACLY, citrate synthase, OGDH, and ATP5C1 (Figure 2(f)). The general information of the common hub differently expressed proteins is presented in Table 2. The heatmap shows the level of expression of the six common hub proteins among the four groups. Compared with the AS group, the six common hub proteins were upregulated in the AS+CS group. Compared with the control group, the six common hub proteins were upregulated in the CS group. Compared with the control group, the hub proteins (citrate synthase and OGDH) were upregulated in the CS group (Figure 2(g)).

### 3.3. Most Significant Hub Proteins

**Figure 3:**
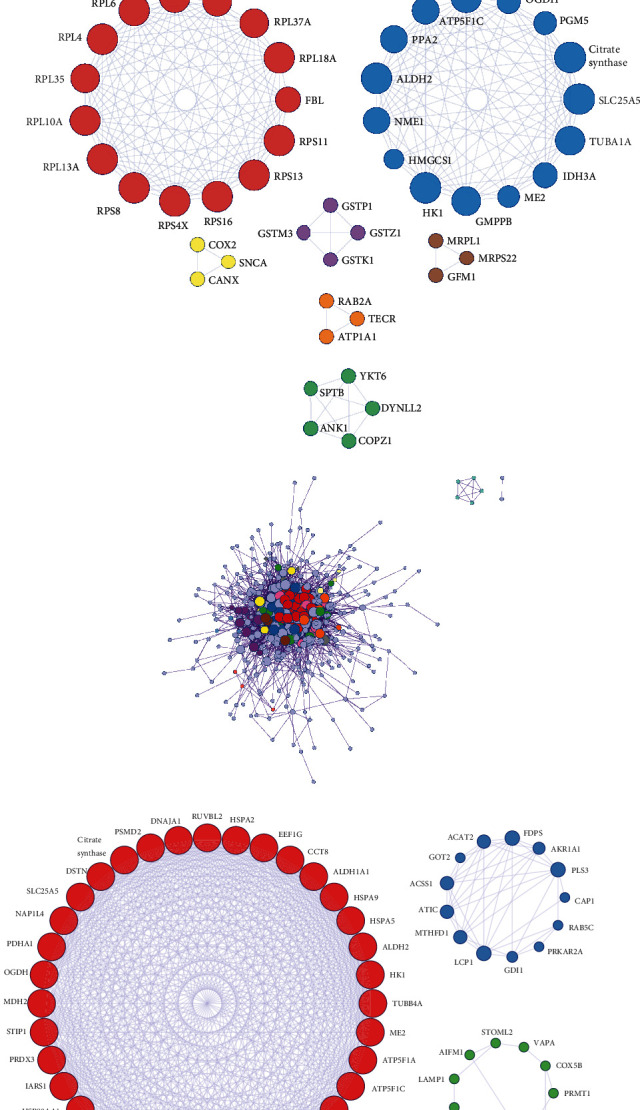
The most significant hub proteins. (a) Overlap between gene lists at the gene level; purple curves link identical genes. (b) Overlap between gene lists at the shared term level; blue curves link genes that belong to the same enriched ontology term. The inner circle represents gene lists, where hits are arranged along the arc. Genes that hit multiple lists are colored in dark orange, and genes unique to a list are shown in light orange. (c) Metascape analysis revealed the PPI network of DEPs between the control and CS groups. (d) Significant module was identified using MCODE. (e) Metascape analysis revealed the PPI network of DEPs between the AS and AS+CS groups. (f) Significant module was identified using MCODE. (g) Eleven common proteins between the two lists. (h) The two most significant hub proteins (OGDH and citrate synthase) were found using Metascape and the animal model.

The overlaps between these lists (one list included the DEPs between the control and CS groups; the other list included the DEPs between the AS and AS+CS groups) are shown in a Circos^3^ plot (Figure 3(a)). Another useful representation is to overlap genes based on their functions or shared pathways. The overlaps between gene lists can be significantly improved by considering overlaps between genes sharing the same enriched Gene Ontology terms (Figure 3(b)). Using Metascape, the PPI network of the DEPs between the control and CS groups was determined and is shown in Figure 3(c), and significant modules were identified using MCODE (Figure 3(d)). The PPI network of the DEPs between the AS and AS+CS groups was also determined and is shown in Figure 3(e), and significant modules were again identified using MCODE (Figure 3(f)). A total of 11 common proteins between the two lists were identified (Figure 3(g)). A Venn diagram shows that the two most significant hub proteins (OGDH and citrate synthase) were found by Metascape and the animal model (Figure 3(h)).

### 3.4. Weighted Coexpression Network Construction and Module Preservation Analysis

**Figure 4:**
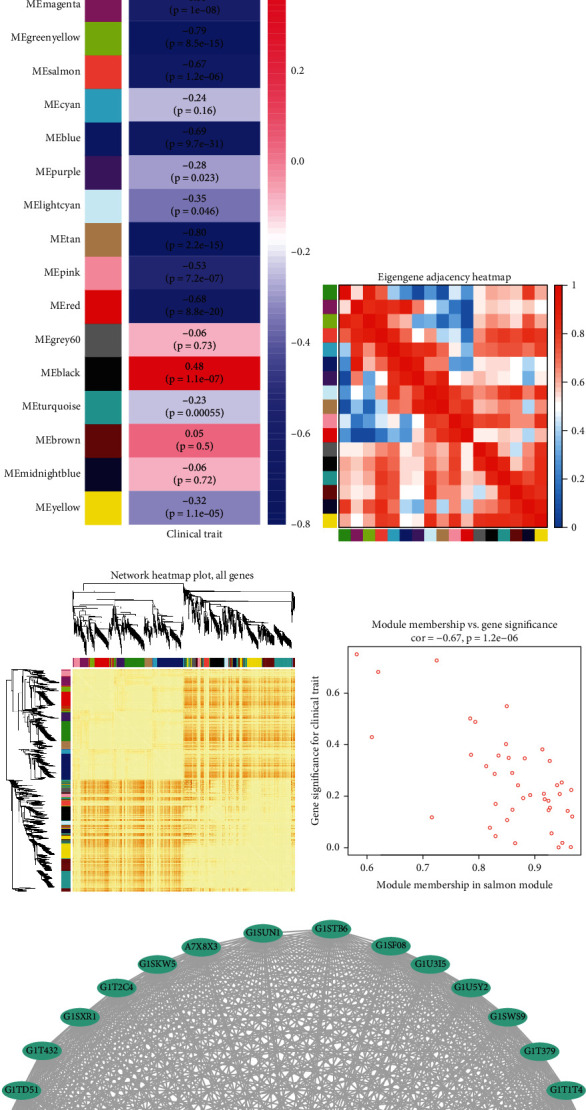
Verification of the most significant hub proteins (OGDH and citrate synthase) using WGCNA. (a) The power of *β* = 19 was chosen as the soft thresholding to guarantee a scale-free network. (b) Hierarchical clustering analysis was performed with the flashClust function. (c) Correlation coefficients and *P* values between each module and each clinical feature. (d) The heatmap was used to quantify groups of correlated eigengenes. (e) Interactions of the 17 coexpression modules were analyzed with all genes; light colors represent high overlap, and progressively darker red colors indicate lower overlap. Blocks of lighter colors along the diagonal represent the coexpression modules. (f) The scatter diagram clearly shows the negative correlation between MEsalmon and clinical traits (*R* = −0.67, *P* = 1.2*e* − 06). (g) Citrate synthase and OGDH play important roles in the PPI network of MEsalmon.

In this study, we used the R software package “WGCNA” to construct a coexpression network. All expression profiles of genes were placed into modules using a cluster analysis. We chose the power of *β* = 19 as the soft threshold to guarantee a scale-free network (Figure 4(a)). A sample dendrogram and trait heatmap is shown in “Supplement 6: Figure S2A”. Hierarchical clustering analysis was performed using the flashClust function, and the results are presented in Figure 4(b); this included 17 modules. The 17 modules were clustered using an eigengene dendrogram (“Supplement 6: Figure S2B”). The associations between the clinical traits we were interested in and modules were identified based on correlation between eigengene modules and the clinical traits. We calculated correlation coefficients and *P* values between each module and each clinical feature to select the modules we were interested in. As we chose *P* < 0.05 as the threshold of significance, the results showed that a close relationship existed between salmon module and clinical traits (*R* = −0.67, *P* = 1.2*e* − 06) (Figure 4(c)). The heatmap was used to quantify groups of correlated eigengenes (Figure 4(d)). Interactions among the 17 coexpressed modules were analyzed for all genes; a light color represents high overlap, and progressively darker red color indicates lower overlap. The blocks of lighter color along the diagonal represent the coexpression modules (Figure 4(e)). A scatter diagram clearly showed the negative correlation between salmon module and clinical traits (*R* = −0.67, *P* = 1.2*e* − 06) (Figure 4(f)). Other scatter diagrams showed the members of the other 16 modules vs. gene significance (Supplement 6: Figure S2C and Figure S3A). Citrate synthase and OGDH played important roles in the PPI network of MEsalmon (Figure 4(g)). Interactions of the selected genes with the 17 coexpression modules were also analyzed; light color represents high overlap, and progressively darker red color indicates lower overlap. Blocks of lighter colors along the diagonal represent the coexpression modules (Supplement 6: Figure S3B).

### 3.5. Enrichment Analysis of the DEPs

**Figure 5:**
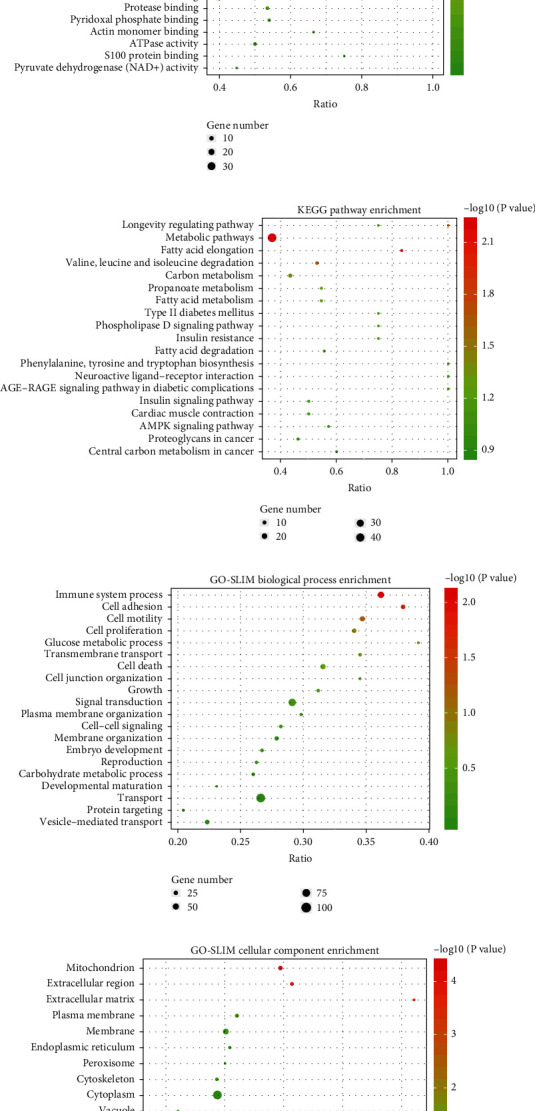
Enrichment analysis of the DEPs. (a) GO biological process enrichment analysis. (b) GO cellular component enrichment analysis. (c) GO molecular function enrichment analysis. (d) KEGG pathway enrichment analysis. (e) GO-slim biological process enrichment analysis. (f) GO-slim cellular component enrichment analysis. (g) GO-slim molecular function enrichment analysis. (h) Stress disorders and adrenal gland diseases related to hub genes, based on the CTD database analysis.

The enrichment analysis results of GO biological processes showed DEPs were mainly enriched in negative regulation of the calcineurin-NFAT cascade, response to calcium ions, and positive regulation of protein phosphorylation (Figure 5(a)). The enrichment analysis results of GO cellular components showed DEPs were mainly enriched in mitochondrial matrix, mitochondria, mitochondrial inner membrane, and mitochondrial proton-transporting ATP synthase complex (Figure 5(b)). The enrichment analysis results of GO molecular function showed DEPs were mainly enriched in calcium ion binding and ATPase activity (Figure 5(c)). The results of KEGG pathway enrichment analysis showed DEPs were mainly enriched in metabolic pathways and the AMPK signaling pathway (Figure 5(d)). Furthermore, a GO-slim enrichment analysis showed DEPs were also mainly enriched in carbohydrate metabolic processes, mitochondria, and metal ion binding (Figures 5(e)–5(g)).

Metascape analysis showed that DEPs were mainly enriched in the generation of precursor metabolites and energy and mitochondrial fatty acid beta-oxidation (Supplement 6: Figure S4A). The network of enriched terms was represented as pie charts, where the charts were color-coded based on the identities of their gene lists (Supplement 6: Figure S4B). A network of enriched terms colored by cluster ID was drawn (Supplement 6: Figure S4C); terms containing more genes tended to have a more significant *P* value (Supplement 6: Figure S4D). The enrichment analysis in DisGeNET showed that DEPs were mainly enriched in mental and motor retardation and cognitive delay (Supplement 6: Figure S4E). In addition, the enrichment analysis in PaGenBase showed DEPs were mainly enriched in the adrenal gland (Supplement 6: Figure S4F).

### 3.6. Identification of Hub Genes

The CTD database shows common hub genes (citrate synthase and OGDH) associated with stress disorders and adrenal gland diseases, as shown in Figure 5(h).

### 3.7. Pathology Staining Analysis

**Figure 6:**
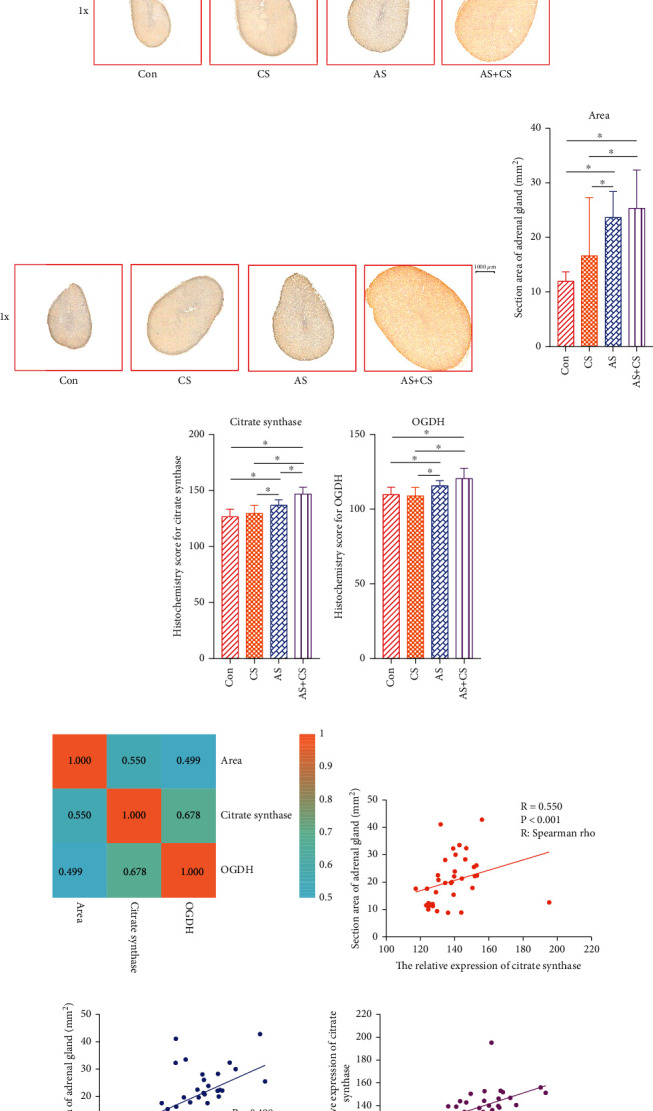
Pathology staining analysis and correlation analysis among the cross-sectional area of adrenal glands, citrate synthase, and OGDH. (a) HE staining of the adrenal glands. (b) Immunohistochemical analysis for the expression of citrate synthase protein. The depth and area of brownish-yellow indicate the expression level of citrate synthase protein. (c) Immunohistochemical analysis for the expression of OGDH. The depth and area of brownish-yellow indicate the expression level of OGDH. (d–f) Quantitative analysis showed that the cross-sectional area of adrenal glands in the AC+CS group was larger than in the control group (*P* < 0.05) and that the expression of citrate synthase (*P* < 0.05) and OGDH (*P* < 0.05) proteins was higher in the AS+CS group than in the control group. (g) The heatmap shows the correlation analysis among the cross-sectional area of adrenal glands, citrate synthase, and OGDH. (h) A strong relationship existed between the cross-sectional area of adrenal glands and relative protein expression of citrate synthase. (i) The cross-sectional area of adrenal glands was related to the relative protein expression of OGDH. (j) A strong relationship existed between the cross-sectional area of adrenal glands and the relative protein expression of citrate synthase.

According to the HE staining analysis, adrenal cells were arranged in a regular order in the control group. Compared with their size in the control group, the adrenal glands were larger in the CS group, while the arrangement of adrenal cells was disordered, irregular, and necrotic in the AS group. Compared with the adrenal glands in the control group, the adrenal glands in the AS+CS group were larger and more disordered, irregular, and necrotic (Figure 6(a)).

Immunohistochemical analysis showed that the expression of citrate synthase (Figure 6(b)) and OGDH (Figure 6(c)) proteins was higher in the AS+CS group than in the control group.

The quantitative analysis showed that the cross-sectional area of the adrenal glands in the AC+CS group was larger than this area in the control group (*P* < 0.05, Figure 6(d)) and that the expression of citrate synthase (*P* < 0.05, Figure 6(e)) and OGDH (*P* < 0.05, Figure 6(f)) proteins was higher in the AS+CS group than in the control group.

### 3.8. Correlation Analysis of the Cross-Sectional Area of the Adrenal Glands, Citrate Synthase, and OGDH

A heatmap showed there were strong correlations among the cross-sectional area of adrenal glands, citrate synthase, and OGDH (Figure 6(g)). Spearman's rho analysis showed that a strong relationship existed between the cross-sectional area of the adrenal glands and relative protein expression of citrate synthase (*P* < 0.001, *R* = 0.550, Figure 6(h)). The cross-sectional area of adrenal glands was also related to the relative protein expression of OGDH (*P* = 0.002, *R* = 0.499, Figure 6(i)). In addition, a strong relationship existed between the cross-sectional area of adrenal glands and the relative protein expression of citrate synthase (*P* < 0.001, *R* = 0.678, Figure 6(j)).

### 3.9. Immunofluorescence Assay Analysis

**Figure 7:**
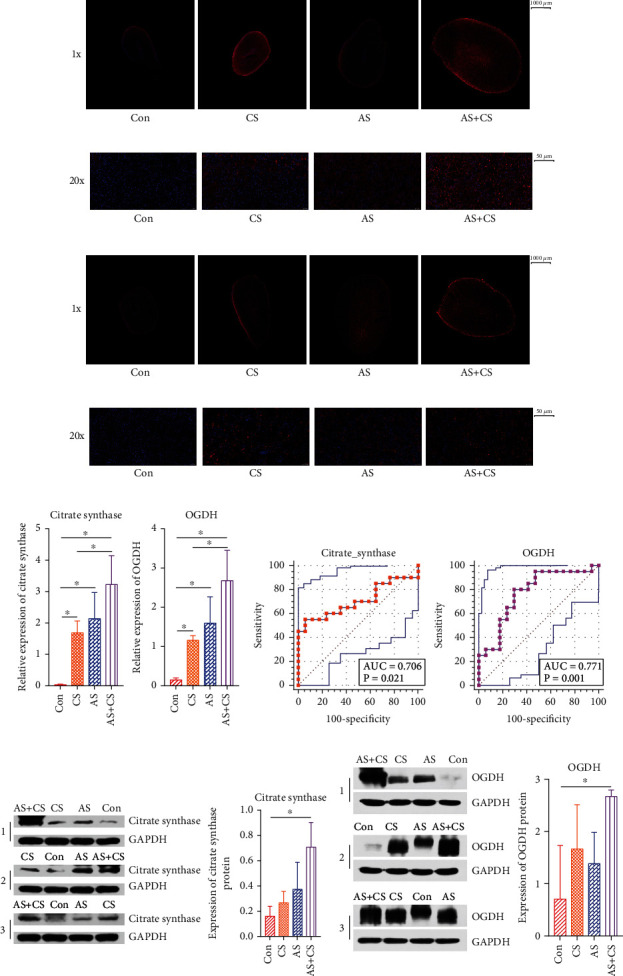
The expression of citrate synthase and OGDH was investigated using immunofluorescence assay, RT-qPCR, and Western blotting analysis, and ROC curves were constructed to determine the effect of these two proteins on the cross-sectional area of adrenal glands. (a, b) The immunofluorescence assay showed that the citrate synthase protein was upregulated in the AS+CS group compared with its expression in the control group. The depth and area of red indicate the expression level of citrate synthase. (c, d) The immunofluorescence assay also showed that the OGDH protein was upregulated in the AS+CS group compared with its expression in the control group. The depth and area of red indicate the expression level of OGDH. (e) The relative expression of citrate synthase and OGDH mRNA was higher in the AS+CS group than in the control group. (f) ROC curve of citrate synthase and OGDH for the cross-sectional area of adrenal glands. (g, h) Western blotting analysis showed that the expression of citrate synthase and OGDH proteins was higher in the AS+CS group than in the control group.

The immunofluorescence assay showed that citrate synthase protein expression was upregulated in the AS+CS group compared with its expression in the control group (Figures 7(a) and 7(b)). OGDH protein expression was upregulated in the AS+CS group compared with its expression in the control group (Figures 7(c) and 7(d)).

### 3.10. RT-qPCR Assay

The RT-qPCR assay enabled citrate synthase and OGDH expression to be detected at the mRNA level. The relative expression of citrate synthase and OGDH mRNA was higher in the AS+CS group than in the control group (*P* < 0.05, Figure 7(e)).

### 3.11. ROC Curve

ROC curves were constructed to determine the effect of citrate synthase and OGDH on the cross-sectional area of adrenal glands, with the degree of confidence judged by the area under the curve (AUC): citrate synthase (AUC = 0.706, *P* = 0.021) and OGDH (AUC = 0.771, *P* = 0.001), as shown in Figure 7(f).

### 3.12. Expression of Citrate Synthase and OGDH at the Protein Level

Western blotting analysis showed that the expression of citrate synthase (Figure 7(g)) and OGDH (Figure 7(h)) proteins was higher in the AS+CS group than in the control group.

### 3.13. Neural Network Model and Cubic Spline Interpolation

**Figure 8:**
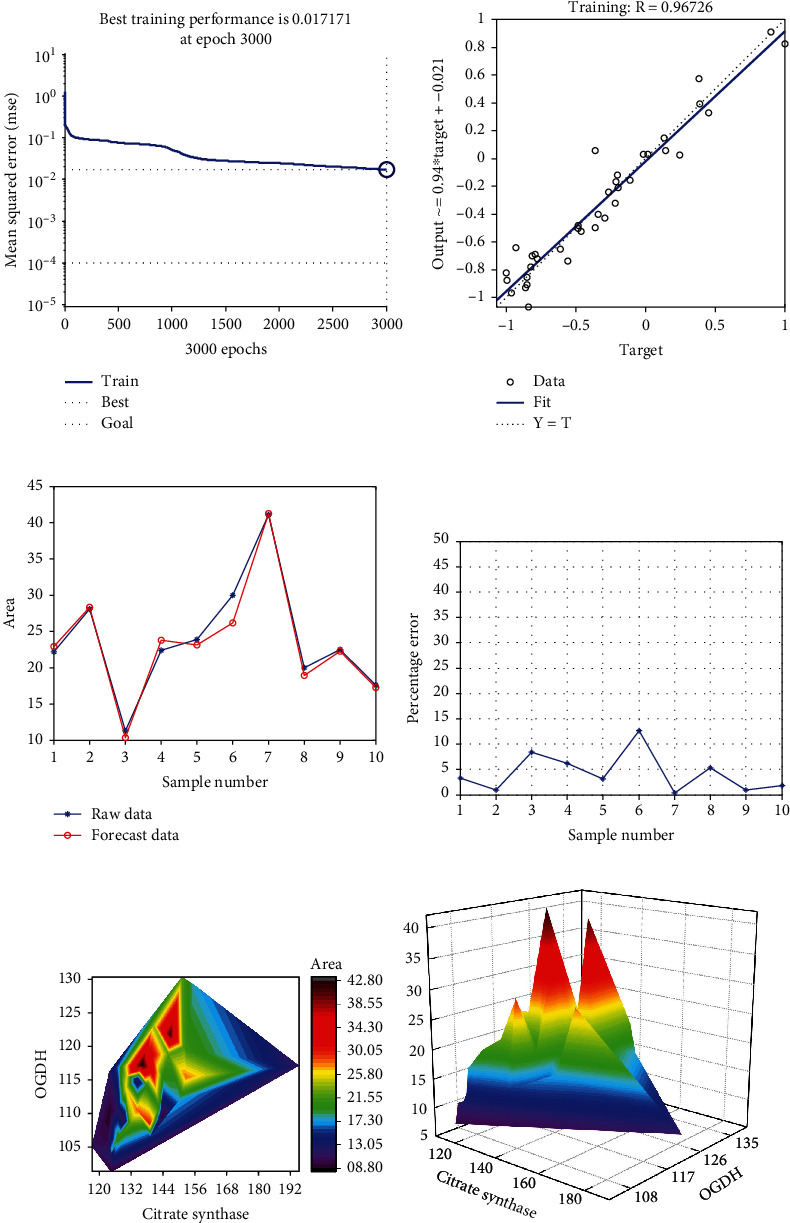
The neural network model for prediction of the cross-sectional area of adrenal glands and cubic spline interpolation. (a) The best training performance was 0.017171 at epoch 3000. (b) The final training model of the neural network prediction model, with a relativity of 0.96726. (c) The predictive value of the data was verified against the actual value. (d) The data error percentage curve was verified. (e, f) The high-risk warning range of the cross-sectional area of adrenal glands at the level of the platform and three-dimensional level.

After training, the neural network prediction model achieved the best effect, where the mean squared error was 0.017171 at epoch 3000 (Figure 8(a)), and the relativity was 0.96726 (Figure 8(b)). Through verifying the predicted value of the data against the actual values, we found there were only small differences (Figures 8(c) and 8(d)). Based on the above results, we could speculate that the level of citrate synthase and OGDH expression might be predictive indexes for the cross-sectional area of adrenal glands. Using the cubic spline interpolation algorithm, we found a high-risk warning indicator of the cross-sectional area of adrenal glands: 132 < citrate synthase < 150 and 115 < OGDH < 130 (Figures 8(e) and 8(f)).

### 3.14. Enzymatic Activity of Mitochondrial Citrate Synthase and OGDH Increased under the Situation of Atherosclerosis and Chronic Stress

**Figure 9:**
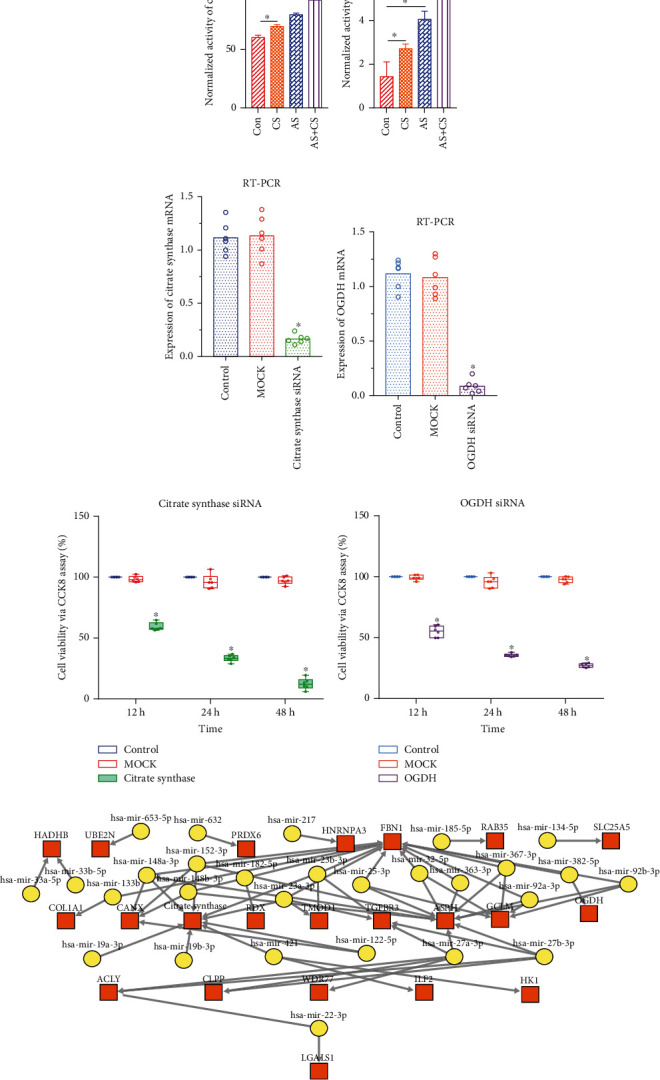
Enzymatic activity of mitochondrial citrate synthase and OGDH, functional verification of citrate synthase and OGDH with overexpression, and knockdown experiments in the corresponding cell lines, target genes-TF regulatory-miRNA regulatory network. (a) Enzymatic activity of mitochondrial citrate synthase. (b) Enzymatic activity of mitochondrial OGDH. (c) Expression of citrate synthase mRNA via RT-PCR after the siRNA. (d) Expression of OGDH mRNA via RT-PCR after the siRNA. (e) Adrenal cell viability decreased after the citrate synthase siRNA. (f) Adrenal cell viability decreased after the OGDH siRNA. (g) Target genes-TF-miRNA regulatory network.

The enzymatic activity of mitochondrial citrate synthase in the AC+CS group was higher than that in the control, CS, and AS groups (*P* < 0.05, Figure 9(a)). Also, the enzymatic activity of mitochondrial OGDH in the AC+CS group was higher than that in the control, CS, and AS groups (*P* < 0.05, Figure 9(b)).

### 3.15. Function of Citrate Synthase and OGDH with Knockdown Experiments in the Adrenal Cell Lines

After siRNA interference to citrate synthase gene, the expression of citrate synthase was detected by RT-PCR, and the expression of citrate synthase in the siRNA group was significantly lower than the control and MOCK groups (Figure 9(c)). In addition, the expression of OGDH in the siRNA group was significantly lower than the control and MOCK groups (Figure 9(d)). Through the CCK8 assay, the adrenal cell viability was downregulated significantly after the knockdown experiment of citrate synthase (Figure 9(e)). Furthermore, the adrenal cell viability was lower in the OGDH siRNA group than that in the control and MOCK groups (Figure 9(f)).

### 3.16. Target Genes-TF-miRNA Regulatory Network

In the target genes-TF-miRNA regulatory network, the close interrelations among the predicted microRNA, citrate synthase, and OGDH were presented (Figure 9(g)).

### 3.17. Coimmunoprecipitation (IP) Assay to Detect the Interaction between Citrate Synthase and OGDH

**Figure 10:**
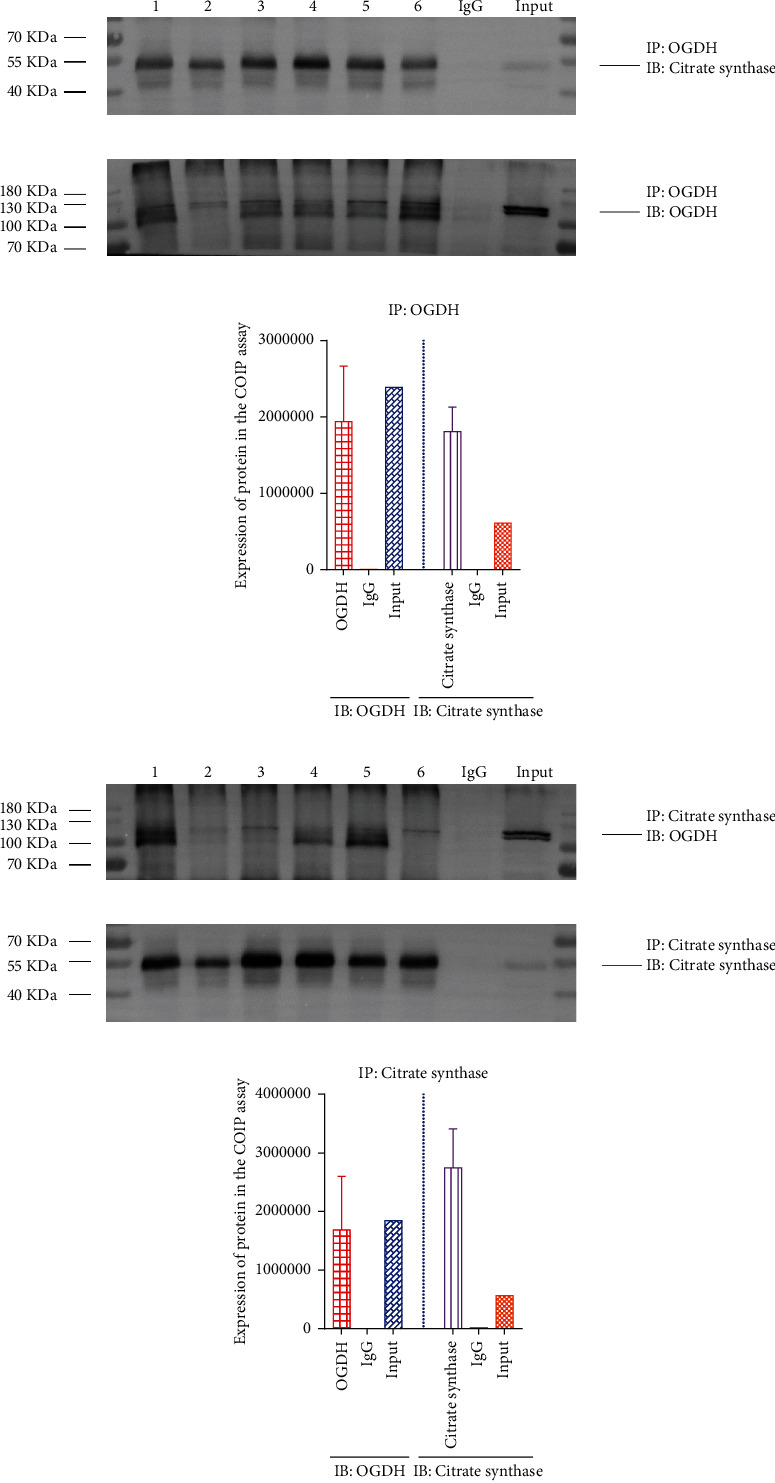
Coimmunoprecipitation assay to detect the interaction between citrate synthase and OGDH. (a, b) The OGDH was as the immunoprecipitation, and then the citrate synthase was measured by the Western blotting. (c, d) The citrate synthase was as the immunoprecipitation, and then the OGDH was measured by the Western blotting.

Through the coimmunoprecipitation assay, firstly, the OGDH was as the immunoprecipitation, and then the citrate synthase was measured by the Western blotting (Figures 10(a) and 10(b)). Furthermore, the citrate synthase was as the immunoprecipitation, and then the OGDH was measured by the Western blotting (Figures 10(c) and 10(d)). After this procedure, the Co-IP experiment manifested that the citrate synthase and OGDH were coexpressed in the adrenal gland.

### 3.18. Molecular Docking Study of OGDH and Citrate Synthase

**Figure 11:**
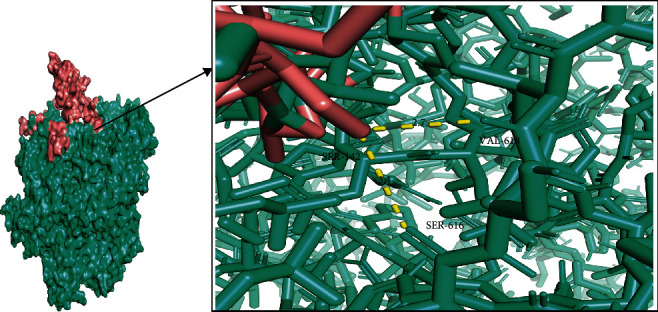
Binding mode of optimal complex conformation between the OGDH and citrate synthase (visualize the selected conformation, Cluster 1).

The binding pattern between the ligand (citrate synthase) and the receptor protein (OGDH) was studied by molecular docking. The binding energy of docking conformations between citrate synthase and OGDH was calculated, and the docking score between the OGDH and citrate synthase was -6.15 kcal/mol (Table 3). The lowest binding energy of optimal complex conformation was -6.15 kcal/mol, and the mean binding energy was -6.03 kcal/mol in the Cluster 1 (Table 4).  Figure 11 shows the binding mode between the OGDH and citrate synthase (visualize the selected conformation, Cluster 1). Hydrogen bond interaction exists between the amino acid residues in OGDH (VAL618, SER616, ARG631, GLU223, LEU151, ALA139, ASN470, and HIS221) and the ligand (citrate synthase) (Table 5).

## 4. Discussion

AS is a disease characterized by the formation of atheromatous plaques in arteries and resulting vascular stenosis as well as sclerosis, which is the main cause of cardiovascular and cerebrovascular diseases [24]. With the rapid development of the modern social economy, social and environmental pressures are increasing, as are CS events from individuals' work and life, affecting people's lives to varying degrees. Studies have shown that long-term CS events are one of the main factors inducing psychological and physical diseases [25]. CS leads to the release of stress hormones, such as cortisol and catecholamines, which can regulate blood flow and blood pressure, leading to endothelial damage, platelet drilling, and hematopoietic stem cell proliferation. Sympathetic stimulation leads to the release of catecholamines, which cause not only coronary artery constriction but also the rupture of vulnerable plaques [26, 27]. The increase in inflammatory cytokines and the expression of adhesion molecules through certain pathways can induce monocyte accumulation and lymphocyte adhesion [28]. In addition, CS leads to an imbalance in lipid metabolism, affects epigenetic patterns, induces depression, directly activates macrophages, promotes foam cell formation, and induces the formation of atherosclerotic plaques [29]. Through epidemiological investigations and laboratory studies, it has been found that stress load has become one of the most important factors in the development of AS in modern society [15]. It is extremely important to fully explore the molecular mechanisms by which CS leads to AS. The main results of this study were that citrate synthase and OGDH genes were highly expressed in adrenal tissue during AS combined with CS, and the degree of adrenal enlargement was more severe when both genes were highly expressed. The DEPs are mainly enriched in calcium ion reactions in terms of biological processes, mitochondrial proton transport ATP synthase complex in cellular localization, ATPase activation in terms of molecular function, and AMPK signaling pathways in the KEGG pathway analysis. These four enriched terms are mainly associated with energy metabolism pathways. These findings could serve as biomarkers for the diagnosis and treatment of AS. In addition, the role of these two proteins (citrate synthase and OGDH) on the pathogenesis of AS and chronic stress deserves special attention.

Citrate synthase, also known as citrate condensation enzyme, is an important component of energy metabolism. It catalyzes the reaction of acetyl-CoA and oxaloacetate, releasing coenzyme A and citric acid, which participate in the TCA cycle as rate-limiting enzymes and help regulate the glyoxylate cycle [30]. This enzyme is present in almost all cells that can undertake oxidative metabolism. In the TCA cycle, citrate synthase activity is inhibited by succinyl-CoA but not by acetyl-CoA or oxaloacetate [31]. In addition, recent studies have revealed the regulatory effects of citrate synthase on other biological processes, such as the respiratory quotient and energy expenditure of mouse mitochondria, human lymphocyte growth, and certain diseases [32]. The OGDH gene encodes a subunit of the 2-oxoglutarate dehydrogenase complex. OGDH-related pathways include the TCA cycle and valproic acid pathways. Gene Ontology (GO) annotations associated with this gene include partner binding and oxidoreductase activity, acting on the aldehyde or oxyl groups of the donor, with disulfide bonds as acceptors. In the TCA cycle, this complex catalyzes the conversion of 2-oxoglutarate (*α*-ketoglutarate) to succinyl-CoA and CO_2_ [33]. Its encoded protein is located in the mitochondrial matrix and uses thiamine pyrophosphate as a cofactor [34]. Thus, high expression of citrate synthase and OGDH promotes the TCA cycle, strengthens the activation of ATP synthase complexes and ATPases, and then promotes ATP production and energy metabolism.

The adrenal gland is an important endocrine gland in the human body and is divided into two parts: the cortex and medulla. The zona fasciculata and zona reticularis of the adrenal gland mainly secrete glucocorticoids (GC), mainly cortisol [35]. GC can act through genomic and nongenomic effects, and GC receptors are present in most tissues in the body, so their effects are extensive. GC has significant effects on ATP production and energy metabolism by enhancing glucose metabolism. GC increases blood glucose mainly by reducing glucose utilization in tissues and accelerating hepatic gluconeogenesis [36]. The main links in the pathway include the following: first, enhancing the activity of enzymes required for intrahepatic gluconeogenesis and glycogen synthesis, using amino acids produced by protein decomposition in peripheral tissues, especially muscle tissues, to accelerate hepatic gluconeogenesis; second, strengthening the responsiveness of the liver to gluconeogenic hormones during fasting; third, inhibiting the oxidation of NADH, thereby reducing glucose glycolysis and reducing the utilization of glucose by peripheral tissue cells; and fourth, inhibiting the binding of insulin to its receptors, reducing the sensitivity of tissue cells to insulin, and reducing the utilization of sugar by peripheral tissues, especially muscle and adipose tissue [37].

GC can be involved in the CS response. When the body suffers from various harmful stimuli, such as trauma, surgery, infection, cold, and fear, the adenohypophysis immediately releases a large amount of adrenocorticotropic hormone. This acts on the adrenal gland which rapidly produces and secretes large quantities of GC, inducing a nonspecific defensive response in the body [38]. GC improves glucose metabolism and energy metabolism, which is conducive to helping the body to fight stressors. On the basis of comprehensive mobilization of overall function, it is of great significance for improving the body's tolerance to harmful stimuli and reducing various adverse reactions to maintain the body's activities [39]. In addition, GC is involved in the regulation of stress-induced secretion. When the body is stimulated by stressors, the secretory function of hypothalamic neurons is enhanced and levels of corticotropin-releasing hormone increase. This stimulates the secretion of adrenocorticotropic hormone by adenohypophysis and ultimately results in a massive secretion of adrenocorticotropic hormone to improve the body's tolerance to noxious stimuli. The increase in adrenocorticotropic hormone (ACTH) secretion during AS is almost exclusively controlled by corticotropin-releasing hormone (CRH) released from the paraventricular nucleus of the hypothalamus [40, 41]. Therefore, we speculate that when the body is in a CS state for a long time, by enhancing the activity of CRH–ACTH–GC system, the central nervous system can significantly increase the secretion of ACTH, completely free of negative feedback. ACTH then continuously acts on the adrenal gland, activating its function. The adrenal gland becomes enlarged to compensate for this and secretes more GC. This increases the expression of citrate synthase and OGDH, promoting the TCA cycle, strengthening the activation of ATP synthase complex and ATPase, and promoting the generation of ATP and energy metabolism. Therefore, it is speculated that citrate synthase and OGDH genes play an important role in the process of adrenomegaly under AS combined with CS.

Our paper not only conducted the study by bioinformatics methods but also verified these findings with animal model and experimental results, which could be benefit for the researchers.

### 4.1. Limitations

Despite the rigorous analysis for vast amounts of data, this study still has some limitations. The above opinion is only based on the animal experiment. However, there are still differences between animals and human. At present, due to ethical issues, it is difficult to obtain normal adrenal tissue from healthy people in clinical practice. Therefore, the results have not been demonstrated by the clinical samples. In addition, no drug intervention trial is performed to further validate the function of these proteins in human. Therefore, in future studies, these aspects should be explored in depth.

## 5. Conclusion

AS combined with CS plays an important role in the HPA axis, promoting adrenomegaly, increasing the release of GC, and possibly enhancing ATP synthesis and energy metabolism in the body through citrate synthase and OGDH gene targets. These findings provide a potential direction for future research into the exploration of related mechanisms.

## Figures and Tables

**Table 1:** Primers and their sequences for RT-PCR analysis.

Primer	Sequence (5′–3′)
*β*-Actin-hF	AGCAAGCAGGAGTATGACGAGT
*β*-Actin-hR	ATGCCAATCTCGTCTCGTTTCT
Citrate synthase-hF	CGTTTCCGAGGTCATAGTATCCC
Citrate synthase-hR	GCTGAGACATAGGGTGTAGGTTGG
OGDH-hF	GCCCACCACCACTTTCATC
OGDH-hR	CCGCTTCTCCTCGTTGGT

**Table 2:** The general information of the common hub differently expressed proteins between AS+CS and AS.

Protein ID	logFC	*P* value	Adj. *P* value	*t* value	Gene name	Description
G1SGG6	65.40	8.64E-06	5.18E-05	14.40	LSS	Terpene cyclase/mutase family member; lanosterol synthase (2,3-oxidosqualene-lanosterol cyclase); belongs to the terpene cyclase/mutase family (733 aa)
G1T671	33.97	0.0801	0.0801	2.11	ACAT2	Uncharacterized protein; acetyl-CoA acetyltransferase 2 (703 aa)
G1T4Z2	39.73	0.0001	0.0004	8.72	ACLY	ATP-citrate synthase; ATP-citrate synthase is the primary enzyme responsible for the synthesis of cytosolic acetyl-CoA in many tissues; in the C-terminal section; belongs to the succinate/malate CoA ligase alpha subunit family (1089 aa)
G1SSK8	41.20	0.0153	0.0195	3.39	CS	Citrate synthase
G1SHI9	38.57	0.0162	0.0195	3.34	OGDH	Oxoglutarate (alpha-ketoglutarate) dehydrogenase (lipoamide) (1032 aa)
G1SM77	37.33	0.0028	0.0057	4.9263	ATP5C1	ATP synthase, H+ transporting, mitochondrial F1 complex, gamma polypeptide 1 (298 aa)

**Table 3:** The docking score between OGDH and citrate synthase.

Protein	Grid_size	Docking score (kcal/mol)
		Citrate synthase
OGDH	86 × 96 × 102	-6.15

**Table 4:** Binding energy of docking conformations between citrate synthase and OGDH.

Cluster rank	Lowest binding energy (kcal/mol)	Mean binding energy (kcal/mol)	No. in cluster
1	-6.15	-6.03	3
2	-5.82	-5.56	8
3	-4.25	-4.02	7
4	-4.20	-4.00	2
5	-3.98	-3.98	1

**Table 5:** Hydrogen bond interaction between the amino acid residues in OGDH and citrate synthase.

	OGDH	Citrate synthase	Distance (A)
Docking scheme 1	VAL618	SER142	2.7
	SER616	SER142	3.4
	ARG631	HIS123	2.6
	GLU223	THR23	3.1
	LEU151	HIS137	2.1
	ALA139	LEU141	2.2
	ASN470	GLN140	1.9
	HIS221	GLN140	2.5

## Data Availability

The data used to support the findings of this study are available from the corresponding authors upon request.

## Supplementary Materials

Supplementary Materials Supplement 1: the detailed information of establishment and assessment of chronic stress model. Table S1: chronic stress procedures. Supplement 2: general data of the animals about body weight, food intake, biochemistry, inflammation, and blood morphology. Table S2: general data of the animals. Figure S1: blood morphology via the wright staining. Supplement 3: sample size calculation. Supplement 4: Limma of full length of DEPs with probe ID, logFC, *P* value, adj. *P* value, *t* value, and gene name. Supplement 5: protein ID_gene name of DEPs. Supplement 6: Figure S2: weighted coexpression network construction. (A) Sample dendrogram and trait heatmap. (B) The eigengene dendrogram could cluster the 17 modules. (C) The scatter diagrams show the membership of the other nine modules vs. gene significance. Figure S3: module preservation analysis. (A) The scatter diagrams show the membership of the other seven modules vs. gene significance. (B) Interactions of the 17 coexpression modules were analyzed with the selected genes; light colors represent high overlap, and progressively darker red color indicates lower overlap. Blocks of lighter colors along the diagonal represent the coexpression modules. Figure S4: enrichment analysis for the DEPs using Metascape. (A) Heatmap of enriched terms across input gene lists, colored by *P* values. (B) The network of enriched terms represented as pie charts, where the charts are color-coded based on the identities of the gene lists. (C) The network of enriched terms colored by cluster ID, where nodes that share the same cluster ID are typically close to each other. (D) The network of enriched terms colored by *P* values, where terms containing more genes tend to have a more significant *P* value. (E) Summary of enrichment analysis using DisGeNET. (F) Summary of enrichment analysis using PaGenBase.
