# Supplementary material for: Citrate Synthase and OGDH as Potential Biomarkers of Atherosclerosis under Chronic Stress
Source: Oxid Med Cell Longev. 2021 Sep 8;2021:9957908. doi: 10.1155/2021/9957908 (PMC8445721; doi:10.1155/2021/9957908)
Supplement: Supplementary Materials — Supplement 1: the detailed information of establishment and assessment of chronic stress model. Table S1: chronic stress procedures. Supplement 2: general data of the animals about body weight, food intake, biochemistry, inflammation, and blood morphology. Table S2: general data of the animals. Figure S1: blood morphology via the wright staining. Supplement 3: sample size calculation. Supplement 4: Limma of full length of DEPs with probe ID, logFC, P value, adj. P value, t value, and gene name. Supplement 5: protein ID_gene name of DEPs. Supplement 6: Figure S2: weighted coexpression network construction. (A) Sample dendrogram and trait heatmap. (B) The eigengene dendrogram could cluster the 17 modules. (C) The scatter diagrams show the membership of the other nine modules vs. gene significance. Figure S3: module preservation analysis. (A) The scatter diagrams show the membership of the other seven modules vs. gene significance. (B) Interactions of the 17 coexpression modules were analyzed with the selected genes; light colors represent high overlap, and progressively darker red color indicates lower overlap. Blocks of lighter colors along the diagonal represent the coexpression modules. Figure S4: enrichment analysis for the DEPs using Metascape. (A) Heatmap of enriched terms across input gene lists, colored by P values. (B) The network of enriched terms represented as pie charts, where the charts are color-coded based on the identities of the gene lists. (C) The network of enriched terms colored by cluster ID, where nodes that share the same cluster ID are typically close to each other. (D) The network of enriched terms colored by P values, where terms containing more genes tend to have a more significant P value. (E) Summary of enrichment analysis using DisGeNET. (F) Summary of enrichment analysis using PaGenBase. [file 9957908.f1.pdf]

Content

Supplement 1:……………………………………………………………………………Page 2
The Detailed information of establishment and assessment of chronic stress model.

Supplement 2:……………………………………………………………………………Page 7
General data of the animals about body weight, food intake, biochemistry, inflammation and blood morphology.

Supplement 3:……………………………………………………………………………Page 12
Sample size calculation.

Supplement 4:……………………………………………………………………………Page 14
Limma of full length of DEPs with probe ID, logFC, pValue, adj.P.Val, t value and Gene Name.

Supplement 5: ……………………………………………………………………………Page122
Protein ID_Gene Name of DEPs.

Supplement 6: ……………………………………………………………………………Page164
Figure S2: Weighted Co-expression Network Construction. (A) Sample dendrogram and trait heatmap. (B) The eigengene dendrogram could cluster the 17 modules. (C) The scatter diagrams show the membership of the other nine modules vs. gene significance.
Figure S3: Module Preservation Analysis. (A) The scatter diagrams show the membership of the other seven modules vs. gene significance. (B) Interactions of the 17 co-expression modules were analyzed with the selected genes; light colors represent high overlap and progressively darker red color indicates lower overlap. Blocks of lighter colors along the diagonal represent the co-expression modules.
Figure S4: Enrichment analysis for the DEPs using Metascape. (A) Heatmap of enriched terms across input gene lists, colored by p-values. (B) The network of enriched terms represented as pie charts, where the charts are color-coded based on the identities of the gene lists. (C) The network of enriched terms colored by cluster ID, where nodes that share the same cluster ID are typically close to each other. (D) The network of enriched terms colored by p-values, where terms containing more genes tend to have a more significant p-value. (E) Summary of enrichment analysis using DisGeNET. (F) Summary of enrichment analysis using PaGenBase.

Supplement 1

Supplement 1: The Detailed information of establishment and assessment of chronic stress model.

Methods:

Detailed information of establishment of chronic stress model[1]

Stress is defined as a threatened state provoked by physiologic, psychological, or environmental stressors [2]. As there is no standard stress procedure for rabbits, we adopted both social stress (referred to as unstable social environment model) and physiologic stress (referred to as rat chronic unpredicted mild stress model, CUMS) methods (Table S1). These two stress groups (CS group and AS+CS group) were assigned in a separate room to avoid interfering the non-stress groups (Normal group and AS group). To reduce gastrointestinal injuries, stress manipulation was conducted after feeding, at least half an hour later.

Social stress procedures: animals were paired together in one cage for 4 hours per day and the pairings were exchanged within the own groups every week, forcing rabbits to continually reestablish social relationships [3]. The time that rabbits in other cage (as intruder) and in home cage during the entire experiments should be balanced.

Physical stress procedures: continuous overnight illumination, 2 hours of white noise (a tone of 80 dB), and 2 hours of high-intensity stroboscopic illumination (300 flashes/min) were selected separately at different days. In order to reduce habituation, one foot shock (1mA) was added (several seconds/time, five times in total)[4].

The stress regime was started at the end of week 4 and lasted for 8 weeks.

Table S1. Chronic stress procedures

Methods	Time					
	Monday	Tuesday	Wednesday	Thursday	Friday	Saturday
**Social stress**	4h	4h	4h	4h	4h	4h
**Physical stress**						
Overnight illumination	12h				-	-
White noise（80db）		2h			-	-
Stroboscopic illumination			2h		-	-
Foot shock（1mA）				5times	-	-

Assessment of chronic stress animal model[1]

The rabbits were carefully inspected after stress exposure. The fur scoring was recorded to reflect the basic health status (referred to as the standard of mouse) [5]. To obtain the information on the impact of the stressors on animal behavior in natural state, all rabbits were recorded for 10 min on video, between 8:00 am and 9:00 am, before the rabbits began eating. The video was recorded 3 - 4 times every week. The individual behavioral assessment of the same group was summed to provide a total score of each 4-week stress period.

Fur score: 1 point represents clean, shiny, and tidy fur; 2 points represent dull and irregular fur, with a few minor wounds; 3 points represent less hair fur, more marked with minor wounds; and 4 points represent lack of hair, ruffled fur, with several minor wounds. Rabbits with obvious wounding were excluded from the study.

Behavior score: it was observed that rabbit behaviors were mainly classified into inactivity, locomotor, grooming, and drinking. Inactivity was defined as rabbits sitting or huddling quietly in the cage. Locomotor behaviors comprised of cage exploration, walking, stretching out, and reacting to the other rabbits in the same room. Grooming behavior included licking their paws and using their paws to wipe and wash their face,

ears, trunk or hind quarters [6]. The behavioral activity of the 4 categories for each rabbit was scored as the percentage of 10-min observation period. The total percent time in each group was calculated every 4 weeks after stress exposure (at week 8 and week 12).

Results:

The CS animal model was established successfully, which was presented in our previous publication. And please see the "Assessments of chronic stress model" section in the "Results" (PMID: 29336364)[1].

References

[1] Z. M. Yu, X. T. Deng, R. M. Qi, L. Y. Xiao, C. Q. Yang, and T. Gong, "Mechanism of Chronic Stress-induced Reduced Atherosclerotic Medial Area and Increased Plaque Instability in Rabbit Models of Chronic Stress," *Chinese medical journal*, vol. 131, no. 2, pp. 161-170, 2018.

[2] P. H. Black and L. D. Garbutt, "Stress, inflammation and cardiovascular disease," *Journal of psychosomatic research*, vol. 52, no. 1, pp. 1-23, 2002.

[3] P. M. McCabe, J. A. Gonzales, J. Zaias et al., "Social environment influences the progression of atherosclerosis in the watanabe heritable hyperlipidemic rabbit," *Circulation*, vol. 105, no. 3, pp. 354-359, 2002.

[4] C. Rabasa, C. Muñoz-Abellán, N. Daviu, R. Nadal, and A. Armario, "Repeated

exposure to immobilization or two different footshock intensities reveals differential adaptation of the hypothalamic-pituitary-adrenal axis," *Physiology & behavior*, vol. 103, no. 2, pp. 125-133, 2011.

[5] H. M. Savignac, N. P. Hyland, T. G. Dinan, and J. F. Cryan, "The effects of repeated social interaction stress on behavioural and physiological parameters in a stress-sensitive mouse strain," *Behavioural brain research*, vol. 216, no. 2, pp. 576-584, 2011.

[6] V. J. Aloyo and K. D. Dave, "Behavioral response to emotional stress in rabbits: role of serotonin and serotonin2A receptors," *Behavioural pharmacology*, vol. 18, no. 7, pp. 651-659, 2007.

Supplement 2

Supplement 2: General data of the animals about body weight, food intake, biochemistry, inflammation and blood morphology

Material and methods:

Blood sampling, blood routine and biochemical assays

Rabbit blood samples were obtained from the central ear artery between 7:00 a.m. and 9:00 a.m. every 4 weeks, after 12 h of fasting. Then take 0.4ml blood and put it into the EDTA anticoagulant tube. The serum samples separated by centrifugation at 4°C were stored in aliquots at −80°C.

The relevant parameters of blood routine, such as white blood cell (WBC), red blood cell (RBC), mean corpuscular volume (MCV), mean corpuscular hemoglobin (MCH), mean erythrocyte hemoglobin concentration (MCHC), red cell distribution width (RDW), platelet (PLT), and mean platelet volume (MPV) were measured by enzymatic assays using a full blood count analyzer (Beckman, USA).

The concentrations of total cholesterol (TC), triglyceride (TG), low-density lipoprotein cholesterin (LDLC), and high-density lipoprotein cholesterin (HDLC), alanine transaminase (ALT), aspartate transaminase (AST), blood glucose (GLU), blood urea (BUN), blood uric acid (URIC) were measured by an automated analyzer (Beckman, USA). Interleukin-6 (IL-6) were measured by commercially available enzyme-linked immunosorbent assay (ELISA) kits (R and D Systems, Minneapolis, MN, USA). High-sensitivity C-reactive protein (hs-CRP) was measured by enhanced immunoturbidimetric assay.

Wright staining

Wright's Giemsa staining kit was bought from the Servicebio (G1009). Use capillary pipette to absorb 5-7 UL of EDTA anticoagulant peripheral blood and drop blood to one end of the slide. The push piece and the slide form an Angle of 30 degrees, and push the blood to the other end of the slide with a uniform speed. Label the blood smear, put the blood smear flat on the staining frame, and drop liquid A to cover the whole blood membrane. After 1 minute, add liquid B, mix liquid A and B evenly. The ratio of liquid A and B is 1:2. After staining in a horizontal position for 5-10 minutes, lift the slide and shake it gently so that the dye is no longer attached to the blood membrane. Use the pure water to wash the dye solution on the tissue, waiting for natural drying. Rapid observation under microscope (Axio Zoom V16, ZEISS, Germany).

Results:

General data of the animals about body weight, food intake, biochemistry, inflammation

The general data of body weight, food intake, biochemistry, inflammation of the animals was presented in the Table S2. There were no differences among the four groups at aspects of body weight, food intake, ALT, AST, BUN, URIC, WBC, RBC, MCV, MCH, RDW, PLT, MPV. Compared with the control group, the TC level in the AS (38.09±2.38 mmol/L) and AS+CS (43.96±0.67 mmol/L) groups was higher ($P<0.05$). The concentration of TG in the AS+CS (5.84±0.72 mmol/L) was higher than the control group (2.77±0.58 mmol/L) ($P<0.05$). Compared with the control group, the

LDLC level in the AS (17.25±1.22 mmol/L) and AS+CS (19.37±0.32 mmol/L) groups was higher (P<0.05). Compared with the control group, the HDLC level in the AS (0.93±0.06 mmol/L) and AS+CS (0.85±0.06 mmol/L) groups was higher (P<0.05). The concentration of GLU in the AS+CS (5.78±0.17 mmol/L) was lower than the control group (6.76±0.24 mmol/L) (P<0.05). Compared with the control group, the MCHC level in the CS (351.31±1.73 g/L) and AS (351.62±1.88 g/L) groups was lower (P<0.05). Compared with the control group, the IL-6 level in the AS (555.52±47.41μg/ml) and AS+CS (787.03±84.59 μg/ml) groups was higher (P<0.05). The concentration of hs-CRP in the AS+CS (1.71±0.20 μg/ml) was higher than the control group (0.63±0.04 μg/ml) (P<0.05).

Table S2. General data of the animals

	Con (Mean±SEM)	CS (Mean±SEM)	AS (Mean±SEM)	AS+CS (Mean±SEM)
Weight (kg)	3.00±0.05	2.95±0.09	3.07±0.04	2.89±0.07
Food (kg/week)	2.41±0.15	2.80±0.05	2.21±0.12	2.19±0.05
TC (mmol/L)	1.81±0.19	2.10±0.35	38.09±2.38*	43.96±0.67*
TG (mmol/L)	2.77±0.58	2.28±0.57	3.86±0.44	5.84±0.72*
LDLC (mmol/L)	0.88±0.13	1.03±0.29	17.25±1.22*	19.37±0.32*
HDLC (mmol/L)	0.63±0.07	0.84±0.09	0.93±0.06*	0.85±0.06*
ALT (U/L)	57.91±7.60	57.77±4.97	65.62±8.98	58.86±5.92
AST (U/L)	45.09±11.63	61.39±15.96	48.54±7.40	38.71±2.68
GLU (mmol/L)	6.76±0.24	6.29±0.17	6.75±0.38	5.78±0.17*
BUN (mmol/L)	4.92±0.25	5.55±0.30	5.31±0.16	5.42±0.30
URIC (μmol/L)	5.27±0.94	3.77±0.56	8.08±3.24	4.21±0.43
WBC (10^9/L)	10.27±0.88	9.96±0.78	12.23±1.07	11.28±0.77
RBC (10^{12}/L)	5.69±0.29	6.13±0.17	6.13±0.10	5.64±0.30
MCV (fl)	66.64±1.81	64.62±1.06	65.08±0.47	65.93±1.34
MCH (pg)	24.24±0.95	22.69±0.38	22.88±0.22	23.55±0.59
MCHC (g/L)	362.27±5.26	351.31±1.73*	351.62±1.88*	356.07±2.92
RDW (%)	12.22±0.45	12.79±0.33	12.18±0.21	12.24±0.26
PLT (10^9/L)	422.18±30.28	367.77±48.58	359.08±57.93	461.14±52.14
MPV (fl)	6.17±0.22	6.46±0.31	5.90±0.19	6.45±0.19
IL-6 (μg/ml)	69.10±3.87	84.70±8.71	555.52±47.41*	787.03±84.59*

hs-CRP (μg/ml)	0.63±0.04	1.18±0.20	1.11±0.17	1.71±0.20*

*Compared with the Con group, P<0.05. TC: total cholesterol, TG: triglyceride, LDLC: low density lipoprotein cholesterin, HDLC: highdensity lipoprotein cholesterin, ALT: alanine transaminase, AST: aspartate transaminase, GLU: blood glucose, BUN: blood urea, URIC: blood uric acid, WBC: white blood cell, RBC: red blood cell, MCV: mean corpuscular volume, MCH: mean corpuscular hemoglobin, MCHC: mean erythrocyte hemoglobin concentration, RDW: red cell distribution width, PLT: platelet, MPV: mean platelet volume, IL-6: interleukin- 6, hs-CRP: hypersensitive C-reactive protein.

Blood morphology

Through the wright staining, there were large number of red blood cells, small size, without nucleus, pink or flesh pink. Neutrophils were slightly larger than red blood cells, nucleus was purple-blue leaf-shaped, and cytoplasm was almost colorless. There were no significant differences among the four groups at aspects of blood morphology (Figure S1).

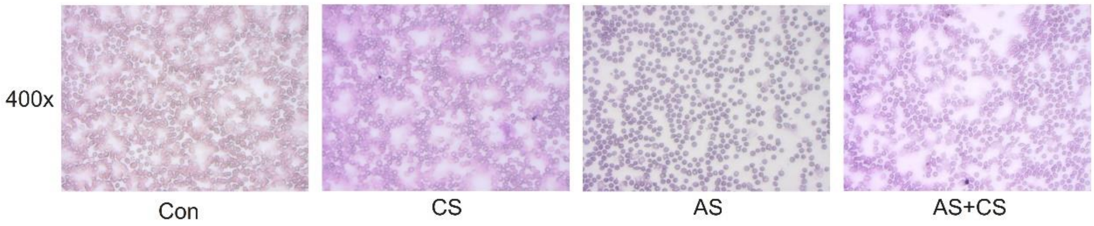

Figure S1: Blood morphology via the wright staining.

Supplement 3

Supplement 3: Sample size calculation

The purpose of this study is to compare the differences in cross-sectional area of adrenal glands among the four groups of subjects. According to the main outcome indicators, cross-sectional areas of adrenal glands, PASS15.0 software was used, and calculation was made according to the sample size formula for comparison of multiple groups of samples. Referring to the pre-experimental data, the mean and standard deviation of the four groups were: 11.97±1.71, 16.62±10.65, 23.63±4.97, 25.31±7.02. The sample size of the four groups was set at 1:1:1:1, and the class I error (α) of the test was assumed to be 0.05. A bilateral test was used, and the class II error (β) was 0.1. The calculated sample size was n1 = n2 = n3 = n4 =9. Considering that the data missing rate was 10%, the final sample size was n1 = n2 = n3 = n4 =10, and 50% more samples were added for the 3 and 4 groups to consider the surgical risk, so that the final sample size was n1 = n2 =10, n3 = n4 =15.

Power	Average n	G	Total N	K	Std Dev of Means σm	Standard Deviation σ	Effect Size	Alpha
0.9934	2.00	4	8	1.00	5.38	1.71	3.1459	0.0500
0.9055	4.00	4	16	1.00	5.38	4.79	1.1231	0.0500
0.9386	8.00	4	32	1.00	5.38	7.02	0.7663	0.0500
0.9022	15.00	4	60	1.00	5.38	10.65	0.5051	0.0500

n1=n2=n3=n4= (2+4+8+15)/4 =9

Appendix: Sample size calculation formula:

$$n = \psi^2 [\sum_{i=1}^{k} s_i^2 / k] / [\sum_{i=1}^{k} (\bar{X}_i - \bar{X})^2 / (k-1)]$$

Supplement 4

Tag	logFC	t	P.Value	adj.P.Val
G1SEF9$$Peptidylprolyl isomerase OS=Oryctolagus cuniculus OX=9986 GN=FKBP5 PE=4 SV=1	-389.0333	-39.4350556	6.58E-08	8.29E-05
P00563$$Sorting nexin-12 OS=Homo sapiens GN=SNX12 PE=1 SV=1	-285.6333	-35.03515778	1.25E-07	8.29E-05
A0A0G2JH24$$Glucosidase 2 subunit beta OS=Homo sapiens GN=PRKCSH PE=1 SV=1	-287.8	-33.2285317	1.67E-07	8.29E-05
P35748$$NADPH--cytochrome P450 reductase OS=Oryctolagus cuniculus OX=9986 GN=POR PE=1 SV=1	-265.1667	-32.77048499	1.80E-07	8.29E-05
G1TJ91$$Integrin alpha-6 OS=Homo sapiens GN=ITGA6 PE=1 SV=5	-323.3333	-31.31775614	2.30E-07	8.49E-05
G1SVK5$$Uncharacterized protein OS=Oryctolagus cuniculus OX=9986 GN=RMDN3 PE=4 SV=1	-244.8333	-29.92409497	2.94E-07	8.50E-05
Q8MJF1$$Uncharacterized protein OS=Oryctolagus cuniculus OX=9986 GN=KCNJ5 PE=3 SV=1	-235.8333	-29.42477138	3.23E-07	8.50E-05
P58776$$Uncharacterized protein OS=Oryctolagus cuniculus OX=9986 GN=ABCC4 PE=4 SV=1	-303.6	-27.21347261	4.93E-07	0.0001136
G1U9R6$$Uncharacterized protein OS=Oryctolagus cuniculus OX=9986 GN=SEC13 PE=3 SV=2	-301.8667	-26.48624789	5.70E-07	0.0001169
U3KML1$$Uncharacterized protein OS=Oryctolagus cuniculus OX=9986 GN=GNAI2 PE=4 SV=1	-243.5333	-24.82508803	8.10E-07	0.0001435
G1SHX8$$""Peroxisomal 2,4-dienoyl-CoA reductase OS=Homo sapiens GN=DECR2 PE=1 SV=1""	-298.1	-24.18976698	9.32E-07	0.0001435
P58772$$Uncharacterized protein OS=Oryctolagus cuniculus OX=9986 GN=RCN1 PE=4 SV=2	-260.9	-24.18155387	9.33E-07	0.0001435
G1T8T3$$""Carnitine O-palmitoyltransferase 2, mitochondrial OS=Homo sapiens GN=CPT2 PE=1 SV=1""	-194.7333	-23.17256583	1.18E-06	0.0001668
G1ST64$$Glycerol-3-phosphate dehydrogenase 1-like protein OS=Homo sapiens GN=GPD1L PE=1 SV=1	-222.7667	-22.25691929	1.46E-06	0.0001848
G1SUY3$$Vacuolar protein sorting-associated protein 28 homolog OS=Oryctolagus cuniculus OX=9986 GN=VPS28 PE=3 SV=1	-210.7333	-22.14195522	1.50E-06	0.0001848
G1TKX3$$Uncharacterized protein OS=Oryctolagus cuniculus OX=9986 GN=CD44 PE=4 SV=2	-169.6667	-20.41583274	2.33E-06	0.0002685
G1TNJ2$$Uncharacterized protein OS=Oryctolagus cuniculus OX=9986 GN=SHPK PE=4 SV=1	-175.3333	-20.03624714	2.58E-06	0.0002796

G1T8V2$$Uncharacterized protein OS=Oryctolagus cuniculus OX=9986 GN=LOC100344979 PE=4 SV=1	-467.4667	-19.82135167	2.73E-06	0.0002799
P29294$$Uncharacterized protein OS=Oryctolagus cuniculus OX=9986 GN=TET1 PE=4 SV=2	-201.9667	-19.47335277	3.00E-06	0.0002837
G1TPZ1$$Galectin OS=Oryctolagus cuniculus OX=9986 GN=LGALS1 PE=4 SV=1	-164.9	-19.38811307	3.08E-06	0.0002837
Q28888$$Uncharacterized protein OS=Oryctolagus cuniculus OX=9986 GN=SRSF5 PE=4 SV=1	-183.8	-18.10962254	4.44E-06	0.00039
G1SDR2$$RNA cytidine acetyltransferase OS=Oryctolagus cuniculus OX=9986 GN=NAT10 PE=3 SV=1	-332.0333	-17.78971767	4.88E-06	0.0003945
G1T0X2$$Alpha-L-fucosidase OS=Oryctolagus cuniculus OX=9986 GN=FUCA1 PE=3 SV=2	-151.3	-17.72617193	4.98E-06	0.0003945
G1TNW2$$Uncharacterized protein OS=Oryctolagus cuniculus OX=9986 GN=TFAM PE=4 SV=1	-208.0667	-17.62677972	5.13E-06	0.0003945
G1SQ27$$Uncharacterized protein OS=Oryctolagus cuniculus OX=9986 GN=NIT1 PE=4 SV=2	-186.3333	-16.65184453	6.96E-06	0.000514
G1T4W4$$""1-phosphatidylinositol 4,5-bisphosphate phosphodiesterase gamma OS=Oryctolagus cuniculus OX=9986 GN=PLCG1 PE=4 SV=1""	-212.9	-16.02209	8.56E-06	0.0006075
G1SML5$$Transcription factor p65 OS=Homo sapiens GN=RELA PE=1 SV=2	119.53333	15.22329488	1.13E-05	0.0007691
G1T933$$Uncharacterized protein OS=Oryctolagus cuniculus OX=9986 GN=CP PE=3 SV=1	-160.8667	-14.67173238	1.37E-05	0.0008962
G1TUH9$$Phosphoglycerate kinase OS=Oryctolagus cuniculus OX=9986 GN=PGK1 PE=3 SV=1	-138.6333	-14.51242465	1.45E-05	0.0008962
G1SXK6$$60S ribosomal protein L29 OS=Oryctolagus cuniculus OX=9986 PE=3 SV=1	-177.8	-14.50404329	1.46E-05	0.0008962
G1U2V8$$60S ribosomal protein L36a OS=Homo sapiens GN=RPL36A PE=1 SV=1	-214.5	-14.29379532	1.58E-05	0.0009218
G1SNC7$$Uncharacterized protein OS=Oryctolagus cuniculus OX=9986 GN=SEPT2 PE=3 SV=2	-171.8667	-14.25403525	1.60E-05	0.0009218
G1T7U6$$Uncharacterized protein OS=Oryctolagus cuniculus OX=9986 GN=ANP32B PE=4 SV=1	-133.2	-13.85163477	1.86E-05	0.0010313
G1SJI4$$Membrane-associated progesterone receptor component 1 OS=Homo sapiens GN=PGRMC1 PE=1 SV=3	-282.9667	-13.79845511	1.90E-05	0.0010313

G1SRW4$$Eukaryotic translation initiation factor 4 gamma 3 OS=Homo sapiens GN=EIF4G3 PE=1 SV=1	-147.4	-13.38923206	2.23E-05	0.0011753
G1T8T5$$Uncharacterized protein OS=Oryctolagus cuniculus OX=9986 GN=PRDX1 PE=4 SV=1	-242.4667	-13.3193111	2.29E-05	0.0011753
G1TD94$$Copine-1 OS=Homo sapiens GN=CPNE1 PE=1 SV=1	-112	-12.63626582	3.03E-05	0.0014658
G1SP97$$Mannose-1-phosphate guanyltransferase alpha OS=Homo sapiens GN=GMPPA PE=1 SV=1	-173.8333	-12.61897197	3.05E-05	0.0014658
G1TEG0$$IlvB acetolactate synthase like OS=Oryctolagus cuniculus OX=9986 GN=ILVBL PE=3 SV=1	97.266667	12.58441385	3.10E-05	0.0014658
G1SNE8$$Uncharacterized protein OS=Oryctolagus cuniculus OX=9986 PE=4 SV=1	-116.6	-12.47388627	3.25E-05	0.0014975
G1TV17$$DnaJ homolog subfamily C member 8 OS=Homo sapiens GN=DNAJC8 PE=1 SV=2	-167.8667	-12.03350816	3.93E-05	0.0017667
G1TVQ3$$Uncharacterized protein OS=Oryctolagus cuniculus OX=9986 GN=COPS5 PE=4 SV=1	108.56667	11.56152264	4.85E-05	0.0020772
G1SVY8$$Transmembrane 9 superfamily member OS=Oryctolagus cuniculus OX=9986 GN=TM9SF4 PE=3 SV=2	-100.0333	-11.53470497	4.91E-05	0.0020772
P41975$$Uncharacterized protein OS=Oryctolagus cuniculus OX=9986 GN=TXNDC17 PE=4 SV=1	-99.06667	-11.51421849	4.95E-05	0.0020772
G1TT06$$Peptidase M20 domain-containing protein 2 OS=Oryctolagus cuniculus OX=9986 GN=PM20D2 PE=3 SV=1	113.3	11.40078245	5.22E-05	0.0021397
P18287$$Cell division cycle 37 OS=Oryctolagus cuniculus OX=9986 GN=CDC37 PE=1 SV=1	-255.0333	-11.34256593	5.36E-05	0.0021503
G1SHB9$$Uncharacterized protein OS=Oryctolagus cuniculus OX=9986 GN=PPT1 PE=4 SV=1	-152.8333	-11.08434575	6.05E-05	0.0023753
G1U9R4$$Uncharacterized protein OS=Oryctolagus cuniculus OX=9986 GN=MIEN1 PE=4 SV=1	-125.8	-10.59478005	7.67E-05	0.0029015
G1TY29$$Uncharacterized protein OS=Oryctolagus cuniculus OX=9986 GN=SBDS PE=4 SV=1	-105.6333	-10.58463839	7.71E-05	0.0029015
G1T824$$Ubiquitin-fold modifier-conjugating enzyme 1 OS=Homo sapiens GN=UFC1 PE=1 SV=3	-100.8	-10.45648957	8.21E-05	0.0030305
G1STS9$$Transgelin OS=Oryctolagus cuniculus OX=9986 GN=TAGLN2 PE=3 SV=1	82.166667	10.31372315	8.82E-05	0.0031073
G1SQG6$$Uncharacterized protein OS=Oryctolagus cuniculus OX=9986 GN=CKAP5 PE=4 SV=1	-82.46667	-10.3126373	8.83E-05	0.0031073

G1TWR0$$Uncharacterized protein OS=Oryctolagus cuniculus OX=9986 PE=4 SV=1	-95.4	-10.29109941	8.93E-05	0.0031073
P18055$$Uncharacterized protein OS=Oryctolagus cuniculus OX=9986 GN=DST PE=4 SV=2	-85.66667	-9.914444766	0.00010841	0.0037038
G1TY57$$Uncharacterized protein OS=Oryctolagus cuniculus OX=9986 GN=VCL PE=4 SV=1	-267.2667	-9.743523563	0.00011866	0.0039806
G1SSV1$$Glycogenin-1 OS=Homo sapiens GN=GYG1 PE=1 SV=4	281.63333	9.309029221	0.00015031	0.0049523
G1SW82$$Transmembrane 9 superfamily member OS=Oryctolagus cuniculus OX=9986 GN=TM9SF3 PE=3 SV=1	-160.1	-9.056422065	0.00017326	0.0056081
G1SL41$$Uncharacterized protein OS=Oryctolagus cuniculus OX=9986 GN=SYNPO2 PE=4 SV=1	-102.3333	-8.99697401	0.00017924	0.005624
G1SVI9$$VAMP associated protein B and C OS=Oryctolagus cuniculus OX=9986 GN=VAPB PE=4 SV=1	74.866667	8.991087591	0.00017985	0.005624
G1T4Q8$$""NAD-dependent protein deacylase sirtuin-5, mitochondrial OS=Oryctolagus cuniculus OX=9986 GN=SIRT5 PE=3 SV=1""	-82.03333	-8.801228592	0.00020073	0.0061724
G1SKM2$$Serine/threonine-protein phosphatase 2A 55 kDa regulatory subunit B alpha isoform OS=Oryctolagus cuniculus OX=9986 GN=PPP2R2A PE=2 SV=1	-147.4333	-8.747806582	0.00020711	0.0061784
G1THZ6$$Annexin OS=Oryctolagus cuniculus OX=9986 GN=ANXA2 PE=3 SV=2	-82.03333	-8.743567998	0.00020762	0.0061784
G1U2Q6$$CAP-Gly domain containing linker protein 1 OS=Oryctolagus cuniculus OX=9986 GN=CLIP1 PE=4 SV=2	-353.5333	-8.699603862	0.00021307	0.0062398
G1SY86$$Uncharacterized protein OS=Oryctolagus cuniculus OX=9986 GN=RAB14 PE=4 SV=1	82.6	8.660523323	0.00021805	0.0062859
P01870$$NADH dehydrogenase [ubiquinone] 1 alpha subcomplex subunit 8 OS=Oryctolagus cuniculus OX=9986 GN=NDUFA8 PE=3 SV=1	-81.36667	-8.622027307	0.00022309	0.0063323
G1SGJ7$$Uncharacterized protein OS=Oryctolagus cuniculus OX=9986 GN=IVD PE=3 SV=2	75.133333	8.338767929	0.00026468	0.007399
G1SVR6$$Uncharacterized protein OS=Oryctolagus cuniculus OX=9986 GN=KIF13B PE=3 SV=2	115.23333	8.150691986	0.00029734	0.0081879
G1SGG6$$Catalase OS=Oryctolagus cuniculus OX=9986 GN=CAT PE=3 SV=2	65.4	14.40150789	8.64E-06	5.18E-05

G1T6J2$$Phosphatidylethanolamine-binding protein 1 OS=Oryctolagus cuniculus OX=9986 GN=PEBP1 PE=4 SV=1	70.533333	8.032759505	0.00032022	0.0085625
G1U685$$Uncharacterized protein OS=Oryctolagus cuniculus OX=9986 GN=EPHX2 PE=4 SV=2	-61.83333	-7.934728158	0.00034083	0.0089832
G1T642$$Peptidyl-prolyl cis-trans isomerase FKBP4 OS=Oryctolagus cuniculus OX=9986 GN=FKBP4 PE=1 SV=3	67.733333	7.879255346	0.00035317	0.0090696
G1TAQ9$$Fibrinogen beta chain OS=Oryctolagus cuniculus OX=9986 GN=FGB PE=4 SV=2	89.533333	7.875914172	0.00035393	0.0090696
G1TMB7$$Regulator of nonsense transcripts 1 OS=Homo sapiens GN=UPF1 PE=1 SV=2	109.23333	7.797424097	0.00037235	0.0092752
G1TCC2$$Guanine nucleotide-binding protein subunit gamma OS=Homo sapiens GN=DNAJC25-GNG10 PE=3 SV=1	60.433333	7.789821518	0.0003742	0.0092752
G1TWS0$$AP-2 complex subunit mu OS=Homo sapiens GN=AP2M1 PE=1 SV=1	65.7	7.745480547	0.00038516	0.0092752
G1U7K4$$""Lamina-associated polypeptide 2, isoform alpha OS=Homo sapiens GN=TMPO PE=1 SV=2""	-62.53333	-7.739723599	0.00038661	0.0092752
G1T8H8$$Ankyrin 1 OS=Oryctolagus cuniculus OX=9986 GN=ANK1 PE=4 SV=2	59.633333	7.737813436	0.0003871	0.0092752
G1TLZ2$$3-hydroxyisobutyrate dehydrogenase OS=Oryctolagus cuniculus OX=9986 GN=HIBADH PE=3 SV=1	63.6	7.674749482	0.00040344	0.0095429
U3KMY5$$Uncharacterized protein OS=Oryctolagus cuniculus OX=9986 GN=COL4A1 PE=4 SV=1	64.433333	7.593269218	0.00042577	0.0098353
Q08863$$Uncharacterized protein OS=Oryctolagus cuniculus OX=9986 GN=FUBP1 PE=4 SV=1	-67.46667	-7.590801446	0.00042646	0.0098353
G1SEN8$$Peptidyl-prolyl cis-trans isomerase OS=Oryctolagus cuniculus OX=9986 GN=PPIB PE=3 SV=1	70.833333	7.504934063	0.00045162	0.0102229
G1SHC5$$Gephyrin OS=Homo sapiens GN=GPHN PE=1 SV=1	78	7.495951627	0.00045435	0.0102229
G1SMH2$$V-type proton ATPase subunit OS=Homo sapiens GN=ATP6V0D1 PE=1 SV=1	-89.73333	-7.454981045	0.00046705	0.010382
G1TT82$$Uncharacterized protein OS=Oryctolagus cuniculus OX=9986 GN=RUFY2 PE=4 SV=2	-72.2	-7.427723966	0.00047573	0.0104402
G1SLS1$$Ras-related protein Rab-3A OS=Homo sapiens GN=RAB3A PE=1 SV=1	59.233333	7.41151163	0.00048098	0.0104402

G1T2A9$$Uncharacterized protein OS=Oryctolagus cuniculus OX=9986 GN=ALDH6A1 PE=4 SV=2	-100.9333	-7.356123201	0.00049945	0.0107148
G1T8Z0$$Transforming growth factor-beta-induced protein ig-h3 OS=Oryctolagus cuniculus OX=9986 GN=TGFBI PE=4 SV=1	64.033333	7.252803979	0.00053614	0.0113509
G1T7A2$$Uncharacterized protein OS=Oryctolagus cuniculus OX=9986 GN=LMCD1 PE=4 SV=1	-89.53333	-7.227139925	0.00054574	0.0113509
G1SQF0$$Uncharacterized protein OS=Oryctolagus cuniculus OX=9986 PE=4 SV=1	-109.3333	-7.222359127	0.00054755	0.0113509
G1SX37$$Uncharacterized protein OS=Oryctolagus cuniculus OX=9986 GN=PYCARD PE=4 SV=1	-87.33333	-7.173566103	0.00056643	0.0114567
G1T8X9$$Probable ATP-dependent RNA helicase DDX46 OS=Homo sapiens GN=DDX46 PE=1 SV=1	59.833333	7.168866399	0.00056829	0.0114567
G1SN70$$Uncharacterized protein OS=Oryctolagus cuniculus OX=9986 GN=HSPB6 PE=3 SV=1	73.033333	7.161320627	0.00057128	0.0114567
G1TA53$$Annexin OS=Oryctolagus cuniculus OX=9986 GN=ANXA3 PE=3 SV=2	58.4	7.129798258	0.00058401	0.011586
Q09YN6$$PRA1 family protein OS=Oryctolagus cuniculus OX=9986 GN=ARL6IP5 PE=3 SV=1	-130.9667	-7.028899612	0.00062703	0.0123072
G1T6M1$$Calponin-1 OS=Homo sapiens GN=CNN1 PE=1 SV=2	68.333333	6.990175215	0.00064452	0.0125172
G1TAL6$$40S ribosomal protein S27 OS=Oryctolagus cuniculus OX=9986 GN=LOC100345694 PE=1 SV=1	85.8	6.954979201	0.0006609	0.0127017
G1TF67$$Fibrinogen alpha chain OS=Oryctolagus cuniculus OX=9986 GN=FGA PE=4 SV=1	-91.13333	-6.881107653	0.0006969	0.0130353
G1U328$$Uncharacterized protein OS=Oryctolagus cuniculus OX=9986 GN=FHIT PE=4 SV=2	83.966667	6.879754402	0.00069758	0.0130353
G1TBD3$$Uncharacterized protein OS=Oryctolagus cuniculus OX=9986 GN=GLOD4 PE=4 SV=2	94.833333	6.87604139	0.00069946	0.0130353
G1U6H6$$Uncharacterized protein OS=Oryctolagus cuniculus OX=9986 GN=RPL21 PE=1 SV=1	-197	-6.855225258	0.00071005	0.0131005
G1T006$$Splicing factor U2AF 65 kDa subunit OS=Homo sapiens GN=U2AF2 PE=1 SV=4	65.7	6.789498003	0.00074477	0.0135741
Q8MI17$$Uncharacterized protein OS=Oryctolagus cuniculus OX=9986 GN=LOC100347914 PE=3 SV=1	-59.4	-6.779109346	0.00075044	0.0135741
G1T7Q3$$Uncharacterized protein OS=Oryctolagus cuniculus OX=9986 GN=AAMP PE=4 SV=1	-64.26667	-6.755845347	0.00076331	0.0136729

G1SRM4$$Peptidylprolyl isomerase (Fragment) OS=Homo sapiens GN=FKBP15 PE=1 SV=1	52.7	6.657520459	0.00082064	0.0145584
G1TED0$$Transgelin-2 OS=Homo sapiens GN=TAGLN2 PE=1 SV=3	51.333333	6.595783866	0.00085919	0.0149732
G1T7S0$$Uncharacterized protein OS=Oryctolagus cuniculus OX=9986 GN=ADHFE1 PE=4 SV=1	-74.23333	-6.594138606	0.00086025	0.0149732
G1SI41$$Uncharacterized protein OS=Oryctolagus cuniculus OX=9986 GN=GPLD1 PE=4 SV=2	107.53333	6.56734286	0.00087766	0.0151131
G1SQ90$$Uncharacterized protein OS=Oryctolagus cuniculus OX=9986 GN=PA2G4 PE=4 SV=1	57.266667	6.55673612	0.00088467	0.0151131
G1TVS8$$Very-long-chain (3R)-3-hydroxyacyl-CoA dehydratase OS=Oryctolagus cuniculus OX=9986 GN=HACD2 PE=3 SV=1	64.2	6.497874491	0.00092475	0.0156529
G1SJI7$$Uncharacterized protein OS=Oryctolagus cuniculus OX=9986 GN=TBCB PE=4 SV=1	79.066667	6.483210399	0.00093507	0.0156836
G1SH81$$Uncharacterized protein OS=Oryctolagus cuniculus OX=9986 GN=GSTP1 PE=4 SV=2	60.8	6.451869617	0.00095756	0.0157935
G1T4A5$$Ras-related protein R-Ras2 OS=Homo sapiens GN=RRAS2 PE=1 SV=1	-140.5333	-6.448230946	0.00096021	0.0157935
G1ST81$$Heat shock 70 kDa protein 4 OS=Homo sapiens GN=HSPA4 PE=1 SV=4	55.466667	6.398837284	0.00099707	0.0157935
G1TDK0$$Importin-5 (Fragment) OS=Homo sapiens GN=IPO5 PE=1 SV=1	-115	-6.39503052	0.00099998	0.0157935
G1SM70$$Uncharacterized protein OS=Oryctolagus cuniculus OX=9986 GN=MPZ PE=4 SV=2	66.366667	6.388316296	0.00100513	0.0157935
G1TRK9$$Uncharacterized protein OS=Oryctolagus cuniculus OX=9986 GN=PHB PE=4 SV=1	-60.7	-6.386003943	0.00100691	0.0157935
G1TYE2$$Uncharacterized protein OS=Oryctolagus cuniculus OX=9986 GN=PSPH PE=4 SV=1	54.633333	6.383314059	0.00100899	0.0157935
G1U754$$Uncharacterized protein OS=Oryctolagus cuniculus OX=9986 GN=CFH PE=4 SV=2	-123.7333	-6.373276837	0.00101679	0.0157935
G1T437$$Uncharacterized protein OS=Oryctolagus cuniculus OX=9986 GN=LCP1 PE=4 SV=1	118.6	6.370876235	0.00101866	0.0157935
G1SQT0$$Uncharacterized protein OS=Oryctolagus cuniculus OX=9986 GN=LOC100341515 PE=3 SV=1	90.866667	6.343438922	0.00104038	0.0159959
G1U6I6$$RNA-binding protein 4 OS=Oryctolagus cuniculus OX=9986 GN=RBM4 PE=4 SV=1	60.2	6.284757949	0.00108867	0.0165215
P67873$$Complement C1q C chain OS=Oryctolagus cuniculus OX=9986 GN=C1QC PE=4 SV=2	56.266667	6.271208959	0.00110018	0.0165215

G1SFV8$$""Eukaryotic translation initiation factor 1A, Y-chromosomal OS=Homo sapiens GN=EIF1AY PE=1 SV=4""	68.366667	6.255945606	0.00111333	0.0165215
G1T2Z5$$Structural maintenance of chromosomes protein 1A OS=Homo sapiens GN=SMC1A PE=1 SV=2	-117.8667	-6.242130681	0.00112538	0.0165215
G1T2K6$$C-Jun-amino-terminal kinase-interacting protein 4 OS=Homo sapiens GN=SPAG9 PE=1 SV=4	65.166667	6.24209016	0.00112541	0.0165215
G1SFE9$$Calcium-binding mitochondrial carrier protein Aralar1 OS=Homo sapiens GN=SLC25A12 PE=1 SV=2	83.066667	6.238809057	0.0011283	0.0165215
G1T276$$Uncharacterized protein OS=Oryctolagus cuniculus OX=9986 GN=FGG PE=4 SV=1	75.933333	6.190420789	0.00117186	0.0168485
G1T6S6$$TSC22 domain family protein 1 OS=Homo sapiens GN=TSC22D1 PE=1 SV=3	48.733333	6.174721858	0.00118641	0.0168485
G1SQI0$$Uncharacterized protein OS=Oryctolagus cuniculus OX=9986 GN=ABCE1 PE=1 SV=1	84.333333	6.168537724	0.0011922	0.0168485
G1SWF6$$Acyl-CoA dehydrogenase short/branched chain OS=Oryctolagus cuniculus OX=9986 GN=ACADSB PE=3 SV=2	119.36667	6.156586805	0.00120348	0.0168485
G1SR53$$Cofilin-1 OS=Homo sapiens GN=CFL1 PE=1 SV=1	-54.93333	-6.155356294	0.00120465	0.0168485
G1SU66$$Fibromodulin OS=Oryctolagus cuniculus OX=9986 GN=FMOD PE=4 SV=1	80.9	6.143945363	0.00121555	0.0168485
P26202$$Rab GDP dissociation inhibitor OS=Oryctolagus cuniculus OX=9986 PE=3 SV=1	97.633333	6.143551906	0.00121593	0.0168485
G1TB78$$Collagen alpha-1(I) chain OS=Oryctolagus cuniculus OX=9986 GN=COL1A1 PE=4 SV=1	80.133333	6.135504715	0.00122369	0.0168485
G1TXK8$$Uncharacterized protein OS=Oryctolagus cuniculus OX=9986 GN=ALDH7A1 PE=3 SV=2	-63.7	-6.087140203	0.00127155	0.0173778
G1TGH1$$40S ribosomal protein S5 OS=Homo sapiens GN=RPS5 PE=1 SV=1	59.866667	6.035513186	0.00132505	0.0179758
G1TTR4$$Triokinase and FMN cyclase OS=Oryctolagus cuniculus OX=9986 GN=TKFC PE=4 SV=1	63.233333	5.966882301	0.00140027	0.0187309
G1SIK0$$Matrin-3 OS=Homo sapiens GN=MATR3 PE=1 SV=1	-52.06667	-5.960694212	0.00140729	0.0187309
G1U9D3$$40S ribosomal protein S17 OS=Homo sapiens GN=RPS17 PE=1 SV=1	52.833333	5.957303559	0.00141116	0.0187309

G1SG31$$Uncharacterized protein OS=Oryctolagus cuniculus OX=9986 GN=PDCD6IP PE=4 SV=1	51.8	5.939375374	0.0014318	0.0187534
P01697$$Mitogen-activated protein kinase 1 OS=Homo sapiens GN=MAPK1 PE=1 SV=3	-75.8	-5.938184647	0.00143319	0.0187534
G1SCE7$$Fibulin-1 OS=Homo sapiens GN=FBLN1 PE=1 SV=1	74.6	5.924540152	0.00144915	0.0188288
G1SVZ8$$S-(hydroxymethyl)glutathione dehydrogenase OS=Oryctolagus cuniculus OX=9986 GN=ADH5 PE=3 SV=2	50.866667	5.899324886	0.0014792	0.0190848
G1U2R1$$Leucine-rich repeat flightless-interacting protein 1 OS=Homo sapiens GN=LRRFIP1 PE=1 SV=2	60.433333	5.881768549	0.00150055	0.0191536
G1TAD3$$MICOS complex subunit OS=Oryctolagus cuniculus OX=9986 GN=CHCHD3 PE=3 SV=2	60.666667	5.874205775	0.00150985	0.0191536
G1TE76$$Transmembrane emp24 domain-containing protein 10 OS=Oryctolagus cuniculus OX=9986 GN=TMED10 PE=3 SV=1	59.8	5.869496733	0.00151568	0.0191536
G1SE63$$Uncharacterized protein OS=Oryctolagus cuniculus OX=9986 GN=CCDC58 PE=4 SV=1	58.733333	5.836716715	0.00155698	0.0193951
G1SZ00$$""Steroidogenic acute regulatory protein, mitochondrial OS=Oryctolagus cuniculus OX=9986 GN=STAR PE=4 SV=1""	-114.6	-5.833043206	0.00156169	0.0193951
G1SK09$$Integrin beta OS=Oryctolagus cuniculus OX=9986 GN=ITGB2 PE=3 SV=1	60.733333	5.829440875	0.00156633	0.0193951
G1U4R5$$Uncharacterized protein OS=Oryctolagus cuniculus OX=9986 GN=LOC100342438 PE=3 SV=1	45.633333	5.792410816	0.0016149	0.0197888
G1SWN7$$L-lactate dehydrogenase B chain OS=Homo sapiens GN=LDHB PE=1 SV=2	44.9	5.788917171	0.00161957	0.0197888
G1SDV3$$Peptidylprolyl isomerase OS=Oryctolagus cuniculus OX=9986 GN=LOC100348481 PE=4 SV=1	53.5	5.756486906	0.0016637	0.0201942
O79431$$Uncharacterized protein OS=Oryctolagus cuniculus OX=9986 GN=LPCAT3 PE=3 SV=1	61.333333	5.70657368	0.00173436	0.0209143
G1TAV9$$Uncharacterized protein OS=Oryctolagus cuniculus OX=9986 GN=ERLIN1 PE=4 SV=2	51.3	5.644880713	0.00182656	0.0218831
G1SVM2$$Uncharacterized protein OS=Oryctolagus cuniculus OX=9986 GN=AKR1B10 PE=4 SV=2	61.266667	5.629709286	0.00185009	0.0220221

G1T673$$Uncharacterized protein OS=Oryctolagus cuniculus OX=9986 PE=4 SV=1	56.833333	5.609082306	0.00188266	0.022152
G1T2H2$$""Aspartate aminotransferase, mitochondrial OS=Oryctolagus cuniculus OX=9986 GN=GOT2 PE=1 SV=2""	52	5.605815441	0.00188787	0.022152
B7NZQ6$$Uncharacterized protein OS=Oryctolagus cuniculus OX=9986 GN=MRPL1 PE=4 SV=2	54.933333	5.599983332	0.00189723	0.022152
G1TCF3$$Uncharacterized protein OS=Oryctolagus cuniculus OX=9986 GN=GMPPB PE=4 SV=2	88.6	5.592672218	0.00190904	0.022152
G1U8P2$$Serotransferrin OS=Oryctolagus cuniculus OX=9986 GN=TF PE=3 SV=1	69.9	5.567477526	0.00195037	0.0224902
G1T676$$Uncharacterized protein OS=Oryctolagus cuniculus OX=9986 GN=CAVIN2 PE=4 SV=1	-58.53333	-5.554371318	0.00197229	0.0225625
G1SPQ0$$Uncharacterized protein OS=Oryctolagus cuniculus OX=9986 GN=VCP PE=3 SV=1	-119.2667	-5.54525981	0.00198769	0.0225625
G1U3B8$$Catenin delta-1 OS=Homo sapiens GN=CTNND1 PE=1 SV=1	118.3	5.541945069	0.00199333	0.0225625
G1SJ74$$Uncharacterized protein OS=Oryctolagus cuniculus OX=9986 GN=HSPA4 PE=3 SV=2	53.566667	5.506459147	0.00205486	0.0231171
G1TRJ6$$Uncharacterized protein OS=Oryctolagus cuniculus OX=9986 GN=RHOA PE=4 SV=1	45.466667	5.488157547	0.00208745	0.0233414
G1THL4$$Peptidyl-prolyl cis-trans isomerase FKBP2 OS=Homo sapiens GN=FKBP2 PE=1 SV=2	47.333333	5.457438193	0.0021435	0.0238238
U3KND5$$Clustered mitochondria protein homolog OS=Oryctolagus cuniculus OX=9986 GN=CLUH PE=3 SV=1	47	5.43988802	0.00217631	0.0239049
G1T5V5$$cAMP-dependent protein kinase type I-alpha regulatory subunit (Fragment) OS=Homo sapiens GN=PRKAR1A PE=1 SV=8	-55.63333	-5.439676274	0.00217671	0.0239049
G1TT75$$Uncharacterized protein OS=Oryctolagus cuniculus OX=9986 GN=TOMM70 PE=4 SV=2	55.1	5.421077692	0.00221211	0.02415
P98049$$Damage specific DNA binding protein 1 OS=Oryctolagus cuniculus OX=9986 GN=DDB1 PE=4 SV=1	45.833333	5.408824311	0.0022358	0.0242651
G1TFD8$$Uncharacterized protein OS=Oryctolagus cuniculus OX=9986 GN=TMOD1 PE=4 SV=1	59.266667	5.380871163	0.00229096	0.0246515
G1T9T5$$Uncharacterized protein OS=Oryctolagus cuniculus OX=9986 GN=ACSF2 PE=4 SV=2	61.566667	5.376802881	0.00229911	0.0246515

G1SLS3$$AP-1 complex subunit gamma OS=Oryctolagus cuniculus OX=9986 GN=AP1G1 PE=3 SV=2	45.266667	5.37065894	0.0023115	0.0246515
G1U6R8$$Isocitrate dehydrogenase [NADP] OS=Oryctolagus cuniculus OX=9986 GN=IDH1 PE=3 SV=1	-79.23333	-5.33276539	0.00238959	0.0252444
G1T1W6$$Uncharacterized protein OS=Oryctolagus cuniculus OX=9986 GN=LRPPRC PE=4 SV=2	61.5	5.328309518	0.00239897	0.0252444
B7NZM0$$Calpastatin OS=Oryctolagus cuniculus OX=9986 GN=CAST PE=4 SV=2	-65.36667	-5.319131048	0.00241843	0.0252444
G1SKT4$$Uncharacterized protein OS=Oryctolagus cuniculus OX=9986 GN=PLA2G12A PE=3 SV=2	49.233333	5.317543049	0.00242182	0.0252444
G1TA01$$T-complex protein 1 subunit zeta OS=Oryctolagus cuniculus OX=9986 GN=CCT6 PE=1 SV=3	59.866667	5.295140092	0.00247017	0.0255805
G1TX74$$Obg-like ATPase 1 OS=Homo sapiens GN=OLA1 PE=1 SV=1	55.266667	5.289827938	0.00248179	0.0255805
G1SFP0$$Annexin A11 OS=Oryctolagus cuniculus OX=9986 GN=ANXA11 PE=1 SV=1	49.966667	5.257504637	0.00255391	0.0261776
G1TA37$$Pyruvate kinase PKM OS=Oryctolagus cuniculus OX=9986 GN=PKM PE=1 SV=4	-47.7	-5.250511331	0.00256983	0.0261953
G1TRG9$$Proteasome subunit beta OS=Oryctolagus cuniculus OX=9986 GN=PSMB7 PE=3 SV=2	43.1	5.223937333	0.00263138	0.0266753
G1TRZ2$$Eukaryotic translation initiation factor 4E OS=Oryctolagus cuniculus OX=9986 GN=EIF4E PE=3 SV=1	-68.36667	-5.211017178	0.00266192	0.0268374
G1TUY5$$Spectrin beta chain OS=Oryctolagus cuniculus OX=9986 GN=SPTB PE=3 SV=1	42.9	5.180712164	0.00273517	0.0272418
G1SEC9$$GDH/6PGL endoplasmic bifunctional protein OS=Oryctolagus cuniculus OX=9986 GN=H6PD PE=4 SV=2	-106	-5.180164858	0.00273652	0.0272418
G1SZ93$$Heat shock protein HSP 90-beta OS=Oryctolagus cuniculus OX=9986 GN=HSP90AB1 PE=1 SV=2	41.6	5.17303475	0.0027541	0.0272418
G1SHL9$$Coatomer subunit gamma OS=Oryctolagus cuniculus OX=9986 GN=COPG1 PE=3 SV=1	50.133333	5.170211299	0.00276109	0.0272418
G1SW97$$DNA topoisomerase 2-beta OS=Homo sapiens GN=TOP2B PE=1 SV=3	60.5	5.155267943	0.00279847	0.0274637
G1U8K3$$Aspartate aminotransferase OS=Oryctolagus cuniculus OX=9986 GN=GOT1 PE=4 SV=1	45.066667	5.13909686	0.00283958	0.0277197

G1SSJ7$$Uncharacterized protein OS=Oryctolagus cuniculus OX=9986 GN=PALD1 PE=4 SV=1	58.533333	5.093366137	0.00295964	0.0287397
G1T035$$Uncharacterized protein OS=Oryctolagus cuniculus OX=9986 GN=RAB31 PE=4 SV=1	45.766667	5.050240011	0.00307826	0.0294317
G1T9R5$$Uncharacterized protein OS=Oryctolagus cuniculus OX=9986 GN=TXNDC5 PE=3 SV=1	-48.53333	-5.047910062	0.00308482	0.0294317
G1SVH8$$EH domain-containing protein 1 OS=Homo sapiens GN=EHD1 PE=1 SV=1	117.13333	5.04506054	0.00309287	0.0294317
A0A140TAW0$$Laminin subunit alpha 4 OS=Oryctolagus cuniculus OX=9986 GN=LAMA4 PE=4 SV=2	40.633333	5.044408383	0.00309472	0.0294317
G1SWY0$$Uncharacterized protein OS=Oryctolagus cuniculus OX=9986 GN=HSPH1 PE=3 SV=1	55.366667	5.035372808	0.00312042	0.0295239
G1TXN1$$Uncharacterized protein OS=Oryctolagus cuniculus OX=9986 GN=MANF PE=4 SV=1	40.6	5.029443495	0.00313741	0.0295333
P12345$$Tubulin beta-6 chain OS=Homo sapiens GN=TUBB6 PE=1 SV=1	41.633333	5.005978771	0.00320573	0.0300232
G1ST52$$Uncharacterized protein OS=Oryctolagus cuniculus OX=9986 GN=ALDH4A1 PE=3 SV=2	-102	-4.983144408	0.00327386	0.0305064
G1SPD1$$Histone H2B OS=Homo sapiens GN=HIST1H2BN PE=1 SV=1	56.566667	4.961720962	0.0033393	0.0309599
G1SJK0$$Coatomer subunit gamma-2 OS=Homo sapiens GN=COPG2 PE=1 SV=1	92.7	4.953157283	0.00336588	0.0310502
G1SW61$$U6 snRNA-associated Sm-like protein LSm3 OS=Oryctolagus cuniculus OX=9986 GN=LSM3 PE=3 SV=1	49.5	4.936980742	0.00341676	0.0311025
G1T9N3$$Uncharacterized protein OS=Oryctolagus cuniculus OX=9986 PE=4 SV=1	73.333333	4.933974017	0.00342631	0.0311025
G1U1W3$$Uncharacterized protein OS=Oryctolagus cuniculus OX=9986 GN=PSMB10 PE=4 SV=2	43.333333	4.930630306	0.00343697	0.0311025
G1T4Z2$$U6 snRNA-associated Sm-like protein LSm8 OS=Oryctolagus cuniculus OX=9986 GN=LSM8 PE=3 SV=1	39.733333	8.720513581	0.00014436	0.0004331
G1TWC3$$Transcription elongation factor A protein 1 OS=Homo sapiens GN=TCEA1 PE=1 SV=1	46.933333	4.914791434	0.00348798	0.0311025
G1T3A6$$Proteasome subunit alpha type-7 OS=Homo sapiens GN=PSMA7 PE=1 SV=1	79.7	4.910998144	0.00350033	0.0311025
G1U5Q7$$Uncharacterized protein OS=Oryctolagus cuniculus OX=9986 PE=4 SV=2	-45.93333	-4.910474111	0.00350204	0.0311025

G1TXW6$$Uncharacterized protein OS=Oryctolagus cuniculus OX=9986 GN=AASS PE=4 SV=2	-265.5667	-4.909136758	0.00350641	0.0311025
G1SPB8$$Coatomer subunit beta' OS=Oryctolagus cuniculus OX=9986 GN=COPB2 PE=3 SV=2	42.5	4.903886536	0.00352361	0.0311056
G1U487$$Uncharacterized protein OS=Oryctolagus cuniculus OX=9986 GN=ECM1 PE=4 SV=2	62.6	4.887638511	0.00357748	0.0314
G1SUU2$$Uncharacterized protein OS=Oryctolagus cuniculus OX=9986 GN=ACAD10 PE=4 SV=1	40.966667	4.883604551	0.003591	0.0314
G1SG72$$NADH dehydrogenase [ubiquinone] 1 alpha subcomplex subunit 2 OS=Oryctolagus cuniculus OX=9986 GN=NDUFA2 PE=4 SV=1	46.366667	4.858710033	0.00367575	0.0319894
G1SV04$$Uncharacterized protein OS=Oryctolagus cuniculus OX=9986 GN=COL4A2 PE=4 SV=1	80.433333	4.851912391	0.00369929	0.0320267
G1TIZ5$$Uncharacterized protein OS=Oryctolagus cuniculus OX=9986 GN=PRMT1 PE=3 SV=1	55.2	4.847473078	0.00371475	0.0320267
G1SCP8$$Uncharacterized protein OS=Oryctolagus cuniculus OX=9986 PE=4 SV=2	-51.73333	-4.830585072	0.00377427	0.0322403
G1T894$$Sodium/potassium-transporting ATPase subunit alpha-1 OS=Oryctolagus cuniculus OX=9986 GN=ATP1A1 PE=1 SV=2	53.533333	4.830529432	0.00377447	0.0322403
P30801$$Uncharacterized protein OS=Oryctolagus cuniculus OX=9986 GN=TRA2B PE=4 SV=1	-120.8667	-4.821951021	0.00380513	0.0323523
G1SXE6$$NADH dehydrogenase [ubiquinone] 1 alpha subcomplex subunit 13 OS=Homo sapiens GN=NDUFA13 PE=1 SV=3	64.033333	4.793860114	0.00390753	0.033012
G1SRL3$$Uncharacterized protein OS=Oryctolagus cuniculus OX=9986 GN=ATIC PE=3 SV=1	131.16667	4.7909015	0.00391849	0.033012
G1SNX5$$Uncharacterized protein OS=Oryctolagus cuniculus OX=9986 GN=RABGGTA PE=4 SV=2	42.966667	4.785875586	0.00393721	0.0330188
G1T1T4$$Uncharacterized protein OS=Oryctolagus cuniculus OX=9986 GN=TMED7 PE=3 SV=1	59.766667	4.768314089	0.0040034	0.0334221
U3KNU8$$Mimecan OS=Oryctolagus cuniculus OX=9986 GN=OGN PE=1 SV=1	180.53333	4.749388149	0.00407618	0.0338763
G1SD77$$""3-hydroxyisobutyryl-CoA hydrolase, mitochondrial OS=Oryctolagus cuniculus OX=9986 GN=HIBCH PE=3 SV=1""	47.233333	4.730710965	0.00414949	0.034331
G1TI10$$Uncharacterized protein OS=Oryctolagus cuniculus OX=9986 GN=PEPD PE=3 SV=1	39.233333	4.702709016	0.00426226	0.0351065

G1SDL3$$Methionine aminopeptidase 2 OS=Oryctolagus cuniculus OX=9986 GN=METAP2 PE=3 SV=1	47.166667	4.679355611	0.00435899	0.0356306
G1SV35$$Vitronectin OS=Oryctolagus cuniculus OX=9986 GN=VTN PE=4 SV=1	43.766667	4.678041641	0.00436451	0.0356306
G1SUP4$$Calcium-transporting ATPase OS=Oryctolagus cuniculus OX=9986 GN=ATP2B3 PE=3 SV=2	62.3	4.66019854	0.00444023	0.0360891
G1SS66$$Uncharacterized protein OS=Oryctolagus cuniculus OX=9986 GN=RNPEP PE=4 SV=1	-88.56667	-4.626917957	0.00458553	0.0371066
G1T647$$Complement C3 OS=Homo sapiens GN=C3 PE=1 SV=2	37.733333	4.621151095	0.00461126	0.0371518
G1T888$$Uncharacterized protein OS=Oryctolagus cuniculus OX=9986 GN=PCCA PE=4 SV=2	56.833333	4.594443927	0.00473258	0.0379635
G1SSX5$$""Proteasome 26S subunit, non-ATPase 13 OS=Oryctolagus cuniculus OX=9986 GN=PSMD13 PE=4 SV=1""	53.433333	4.564853283	0.00487129	0.038825
G1U8Y2$$Protein kinase C delta type OS=Oryctolagus cuniculus OX=9986 GN=PRKCD PE=3 SV=2	61.966667	4.562596172	0.00488206	0.038825
G1T521$$""Nascent polypeptide-associated complex subunit alpha, muscle-specific form OS=Homo sapiens GN=NACA PE=1 SV=1""	-56.13333	-4.550346868	0.00494099	0.039125
G1TH03$$Myosin-14 OS=Homo sapiens GN=MYH14 PE=1 SV=2	40	4.481275773	0.00528891	0.0416269
G1U7L4$$Uncharacterized protein OS=Oryctolagus cuniculus OX=9986 GN=CCDC91 PE=4 SV=1	47.333333	4.473907083	0.00532766	0.0416269
G1T5S9$$Uncharacterized protein OS=Oryctolagus cuniculus OX=9986 GN=LOC100354063 PE=1 SV=1	39.9	4.464730266	0.00537637	0.0416269
G1TKA4$$Uncharacterized protein OS=Oryctolagus cuniculus OX=9986 GN=TFRC PE=4 SV=2	54.3	4.463079191	0.00538518	0.0416269
G1U4E6$$Staphylococcal nuclease domain-containing protein OS=Oryctolagus cuniculus OX=9986 GN=SND1 PE=4 SV=2	66.266667	4.462819395	0.00538657	0.0416269
G1STU0$$""NADPH:adrenodoxin oxidoreductase, mitochondrial OS=Homo sapiens GN=FDXR PE=1 SV=3""	80.7	4.459099933	0.0054065	0.0416269
G1T8I8$$Uncharacterized protein OS=Oryctolagus cuniculus OX=9986 GN=LPCAT2 PE=4 SV=2	50.933333	4.457540661	0.00541488	0.0416269
G1SHH5$$Uncharacterized protein OS=Oryctolagus cuniculus OX=9986 GN=NAPA PE=4 SV=1	-98.7	-4.44725831	0.00547051	0.0418801

G1U2V6$$Uncharacterized protein OS=Oryctolagus cuniculus OX=9986 GN=GLS PE=3 SV=1	39.866667	4.439255175	0.00551427	0.0420406
G1SGE9$$Uncharacterized protein OS=Oryctolagus cuniculus OX=9986 GN=ESYT1 PE=4 SV=2	35.533333	4.426117685	0.00558696	0.0424195
G1SQ02$$""GTP:AMP phosphotransferase AK3, mitochondrial OS=Oryctolagus cuniculus OX=9986 GN=AK3 PE=3 SV=1""	36.4	4.420927579	0.00561598	0.0424651
G1SH78$$Uncharacterized protein OS=Oryctolagus cuniculus OX=9986 GN=LOC100353185 PE=4 SV=2	-40.63333	-4.404821119	0.00570714	0.0429782
G1SP34$$Uncharacterized protein OS=Oryctolagus cuniculus OX=9986 GN=NDUFS1 PE=3 SV=1	-34.7	-4.394711895	0.00576522	0.043089
G1SR13$$Uncharacterized protein OS=Oryctolagus cuniculus OX=9986 GN=PSMC6 PE=3 SV=1	51.466667	4.394133928	0.00576856	0.043089
G1T2N1$$Uncharacterized protein OS=Oryctolagus cuniculus OX=9986 GN=ATP5IF1 PE=4 SV=2	-36.16667	-4.38960652	0.00579481	0.0431105
G1SYM7$$""Dynein light chain 2, cytoplasmic OS=Homo sapiens GN=DYNLL2 PE=1 SV=1""	-79.03333	-4.378187612	0.00586161	0.0434324
G1SRB1$$60S ribosomal protein L11 OS=Homo sapiens GN=RPL11 PE=1 SV=2	39.466667	4.358253362	0.00598036	0.0438345
G1U3M5$$Syntrophin beta 2 OS=Oryctolagus cuniculus OX=9986 GN=SNTB2 PE=4 SV=1	36	4.356655262	0.00598999	0.0438345
G1TEP2$$Uncharacterized protein OS=Oryctolagus cuniculus OX=9986 GN=LOC100352842 PE=4 SV=1	39.533333	4.355752578	0.00599545	0.0438345
G1T2J6$$Uncharacterized protein OS=Oryctolagus cuniculus OX=9986 GN=MECR PE=4 SV=1	51.766667	4.353196231	0.00601092	0.0438345
G1U1F6$$Uncharacterized protein OS=Oryctolagus cuniculus OX=9986 GN=MTX2 PE=4 SV=2	-146.9333	-4.343249671	0.00607154	0.0441024
G1SF36$$Uncharacterized protein OS=Oryctolagus cuniculus OX=9986 PE=4 SV=2	42.6	4.319473366	0.00621932	0.0449986
G1SF95$$""NPL4 homolog, ubiquitin recognition factor OS=Oryctolagus cuniculus OX=9986 GN=NPLOC4 PE=4 SV=1""	41.5	4.300873259	0.00633778	0.0456766
G1TDR3$$40S ribosomal protein SA OS=Homo sapiens GN=RPSA PE=1 SV=1	-41.9	-4.281242636	0.00646561	0.046352
G1TET3$$Glutathione S-transferase OS=Oryctolagus cuniculus OX=9986 GN=GSTM3 PE=3 SV=2	45.433333	4.278796805	0.00648174	0.046352

G1TEW4$$Mitochondrial-processing peptidase subunit alpha OS=Homo sapiens GN=PMPCA PE=1 SV=2	49.433333	4.269726741	0.00654197	0.0466021
G1TCE9$$Cytoplasmic aconitate hydratase OS=Oryctolagus cuniculus OX=9986 GN=ACO1 PE=1 SV=1	37.066667	4.265882397	0.00656769	0.0466053
G1T4W2$$Beta-glucuronidase OS=Oryctolagus cuniculus OX=9986 GN=GUSB PE=3 SV=2	42.166667	4.243184799	0.0067219	0.0472806
G1TYV6$$Uncharacterized protein OS=Oryctolagus cuniculus OX=9986 GN=JAGN1 PE=4 SV=1	37.733333	4.241903485	0.00673073	0.0472806
G1U5M7$$Uncharacterized protein OS=Oryctolagus cuniculus OX=9986 PE=4 SV=1	64.666667	4.240600151	0.00673972	0.0472806
O97972$$PDGFA associated protein 1 OS=Oryctolagus cuniculus OX=9986 GN=PDAP1 PE=4 SV=1	80.466667	4.227695268	0.00682951	0.0474893
G1TB96$$""IBA57, iron-sulfur cluster assembly OS=Oryctolagus cuniculus OX=9986 GN=IBA57 PE=4 SV=1""	-74.9	-4.224021228	0.00685532	0.0474893
P06813$$Glycine cleavage system H protein OS=Oryctolagus cuniculus OX=9986 GN=LOC100338787 PE=3 SV=1	-34	-4.222800067	0.00686393	0.0474893
G1U1X6$$5-oxoprolinase OS=Homo sapiens GN=OPLAH PE=1 SV=3	43.5	4.221594304	0.00687243	0.0474893
G1STW2$$Uncharacterized protein OS=Oryctolagus cuniculus OX=9986 GN=HNRNPH3 PE=4 SV=1	46.5	4.2130064	0.00693337	0.047531
G1T7V5$$Chitinase domain containing 1 OS=Oryctolagus cuniculus OX=9986 GN=CHID1 PE=4 SV=1	41.2	4.210255902	0.00695302	0.047531
G1TA48$$""2',3'-cyclic nucleotide 3' phosphodiesterase OS=Oryctolagus cuniculus OX=9986 GN=CNP PE=4 SV=1""	59.966667	4.209874074	0.00695575	0.047531
G1SDZ3$$Uncharacterized protein OS=Oryctolagus cuniculus OX=9986 GN=CCDC93 PE=4 SV=1	-85.06667	-4.203575245	0.00700101	0.0476022
G1U2E6$$tRNA-splicing ligase RtcB homolog OS=Oryctolagus cuniculus OX=9986 GN=RTCB PE=3 SV=2	52.333333	4.200708161	0.00702172	0.0476022
U3KLT5$$Uncharacterized protein OS=Oryctolagus cuniculus OX=9986 GN=SPTAN1 PE=4 SV=2	73.866667	4.192295173	0.00708289	0.0476022
G1SE95$$Uncharacterized protein OS=Oryctolagus cuniculus OX=9986 GN=ACADL PE=3 SV=1	-162.9	-4.188138831	0.00711334	0.0476022

G1TLI7$$Uncharacterized protein OS=Oryctolagus cuniculus OX=9986 GN=RANGAP1 PE=4 SV=2	57.733333	4.183644758	0.00714643	0.0476022
P29694$$Uncharacterized protein OS=Oryctolagus cuniculus OX=9986 GN=BUB3 PE=4 SV=2	44.233333	4.181643981	0.00716121	0.0476022
G1SFH9$$Coatomer subunit delta OS=Oryctolagus cuniculus OX=9986 GN=ARCN1 PE=3 SV=1	40.566667	4.181433515	0.00716277	0.0476022
G1T376$$Uncharacterized protein OS=Oryctolagus cuniculus OX=9986 PE=4 SV=2	40.866667	4.180109143	0.00717258	0.0476022
G1SQP0$$Uncharacterized protein OS=Oryctolagus cuniculus OX=9986 GN=EFEMP1 PE=4 SV=2	103.86667	4.173101241	0.00722474	0.0477765
P04221$$Uncharacterized protein OS=Oryctolagus cuniculus OX=9986 GN=ADAR PE=4 SV=1	-46.86667	-4.164031436	0.00729289	0.048055
G1T3E2$$Ubiquitin-conjugating enzyme E2 N OS=Homo sapiens GN=UBE2N PE=1 SV=1	41.5	4.149852115	0.00740091	0.0485932
G1SRI8$$Uncharacterized protein OS=Oryctolagus cuniculus OX=9986 GN=GANAB PE=3 SV=1	36.5	4.145675234	0.00743307	0.0486313
G1U410$$Uncharacterized protein OS=Oryctolagus cuniculus OX=9986 GN=GSN PE=4 SV=2	95.1	4.1374065	0.00749722	0.0488776
G1SVH0$$Uncharacterized protein OS=Oryctolagus cuniculus OX=9986 PE=4 SV=2	42.466667	4.125161742	0.00759337	0.0492707
G1U9S2$$Cullin-associated NEDD8-dissociated protein 1 OS=Homo sapiens GN=CAND1 PE=1 SV=2	-80.3	-4.122945251	0.00761092	0.0492707
G1SR20$$Uncharacterized protein OS=Oryctolagus cuniculus OX=9986 GN=PGRMC1 PE=3 SV=1	31.733333	4.115404059	0.00767099	0.049486
G1SFJ9$$Sarcolemmal membrane-associated protein OS=Oryctolagus cuniculus OX=9986 GN=SLMAP PE=4 SV=1	46.166667	4.088217268	0.00789208	0.0504649
G1TLL7$$Uncharacterized protein OS=Oryctolagus cuniculus OX=9986 GN=BCAP31 PE=4 SV=1	46.766667	4.085049052	0.00791831	0.0504649
B6S6L6$$""Glycerol-3-phosphate dehydrogenase [NAD(+)], cytoplasmic OS=Oryctolagus cuniculus OX=9986 GN=GPD1 PE=3 SV=2""	-141.8667	-4.084163673	0.00792566	0.0504649
U3KNW1$$Uncharacterized protein OS=Oryctolagus cuniculus OX=9986 GN=TIMM10 PE=4 SV=1	38.966667	4.08338216	0.00793215	0.0504649
Q28619$$Uncharacterized protein OS=Oryctolagus cuniculus OX=9986 PE=4 SV=1	47.7	4.072530418	0.00802293	0.050867

G1T3N8$$Uncharacterized protein OS=Oryctolagus cuniculus OX=9986 GN=LOC100358162 PE=1 SV=1	53.233333	4.041737923	0.00828702	0.0523615
G1TF72$$Serine/arginine-rich splicing factor 10 OS=Homo sapiens GN=SRSF10 PE=1 SV=1	54.566667	4.038414185	0.00831611	0.052366
G1SZ16$$Polypyrimidine tract-binding protein 1 OS=Homo sapiens GN=PTBP1 PE=1 SV=1	76.333333	3.99624275	0.00869548	0.0545407
G1T103$$15 kDa protein A OS=Oryctolagus cuniculus OX=9986 PE=1 SV=2	42.333333	3.993523146	0.00872061	0.0545407
G1SFH6$$Small nuclear ribonucleoprotein U1 subunit 70 OS=Oryctolagus cuniculus OX=9986 GN=SNRNP70 PE=4 SV=2	33.433333	3.981803551	0.00882985	0.0548938
G1SUG3$$Fibronectin OS=Oryctolagus cuniculus OX=9986 GN=FN1 PE=4 SV=1	39.033333	3.981088119	0.00883657	0.0548938
G1ST49$$Uncharacterized protein OS=Oryctolagus cuniculus OX=9986 GN=PDHB PE=4 SV=2	34.966667	3.967410843	0.00896614	0.055485
G1T3X1$$Uncharacterized protein OS=Oryctolagus cuniculus OX=9986 GN=LOC100345328 PE=4 SV=1	-72.66667	-3.964720656	0.00899188	0.055485
G1SDL9$$Uncharacterized protein OS=Oryctolagus cuniculus OX=9986 GN=MYH9 PE=3 SV=1	45.9	3.953566019	0.0090995	0.0559619
G1T5J8$$Uncharacterized protein OS=Oryctolagus cuniculus OX=9986 GN=NARS PE=4 SV=1	33.5	3.94771503	0.00915653	0.0561256
G1SYR9$$Mitogen-activated protein kinase 3 OS=Homo sapiens GN=MAPK3 PE=1 SV=4	30.4	3.936070294	0.00927125	0.0566406
G1SRF7$$AU RNA binding methylglutaconyl-CoA hydratase OS=Oryctolagus cuniculus OX=9986 GN=AUH PE=3 SV=1	53.233333	3.931212931	0.00931958	0.0567479
G1TDH4$$ELAV-like protein OS=Oryctolagus cuniculus OX=9986 GN=ELAVL1 PE=3 SV=2	98.933333	3.92107589	0.00942137	0.0570002
G1TIC9$$Succinyl-CoA:3-ketoacid-coenzyme A transferase OS=Oryctolagus cuniculus OX=9986 GN=OXCT1 PE=3 SV=2	55.1	3.920934613	0.0094228	0.0570002
G1T2M9$$Uncharacterized protein OS=Oryctolagus cuniculus OX=9986 GN=LOC100008973 PE=3 SV=1	-38.83333	-3.896240169	0.00967612	0.0583413
G1T2E6$$Uncharacterized protein OS=Oryctolagus cuniculus OX=9986 GN=PFDN6 PE=4 SV=1	30.4	3.867228777	0.00998363	0.0599993
G1STH0$$Factor VIII intron 22 protein OS=Homo sapiens GN=F8A1 PE=1 SV=2	38.833333	3.851965267	0.01014984	0.0608002

O77819$$""Tropomyosin 1 (Alpha), isoform CRA_f OS=Homo sapiens GN=TPM1 PE=1 SV=1""	-35.23333	-3.846062702	0.01021496	0.0609922
G1T7D3$$Adipocyte plasma membrane associated protein OS=Oryctolagus cuniculus OX=9986 GN=APMAP PE=4 SV=2	-37.36667	-3.841025507	0.0102709	0.0611284
G1T3H3$$Atlastin GTPase 3 OS=Oryctolagus cuniculus OX=9986 GN=ATL3 PE=4 SV=1	40.1	3.825447819	0.01044611	0.061828
G1SRD2$$Uncharacterized protein OS=Oryctolagus cuniculus OX=9986 GN=VIM PE=3 SV=1	34.133333	3.822731301	0.01047701	0.061828
G1SSM4$$Uncharacterized protein OS=Oryctolagus cuniculus OX=9986 GN=LDAH PE=4 SV=2	114.43333	3.81951156	0.01051376	0.061828
G1SRA9$$Hepatoma-derived growth factor OS=Homo sapiens GN=HDGF PE=1 SV=1	-61.26667	-3.818748947	0.01052249	0.061828
G1STN6$$Uncharacterized protein OS=Oryctolagus cuniculus OX=9986 GN=S100A13 PE=3 SV=1	58.933333	3.814955403	0.01056602	0.0618867
G1SIN7$$Protein-L-isoaspartate O-methyltransferase OS=Oryctolagus cuniculus OX=9986 GN=PCMT1 PE=3 SV=2	31.566667	3.79395635	0.01081072	0.0628814
G1SL80$$Amine oxidase OS=Oryctolagus cuniculus OX=9986 GN=AOC2 PE=3 SV=2	38.2	3.79107516	0.01084479	0.0628814
G1U6M8$$Uncharacterized protein OS=Oryctolagus cuniculus OX=9986 GN=RDH11 PE=3 SV=1	-92.7	-3.79055399	0.01085097	0.0628814
G1U8Z2$$""Elongation factor Ts, mitochondrial OS=Oryctolagus cuniculus OX=9986 GN=TSFM PE=3 SV=2""	47.5	3.786107962	0.01090381	0.0628814
G1SQU6$$Uncharacterized protein OS=Oryctolagus cuniculus OX=9986 GN=SPR PE=4 SV=2	-30.6	-3.783900356	0.01093016	0.0628814
G1SQF8$$T-complex protein 1 subunit delta OS=Oryctolagus cuniculus OX=9986 GN=CCT4 PE=3 SV=1	59.8	3.783048898	0.01094034	0.0628814
G1SRS3$$Uncharacterized protein OS=Oryctolagus cuniculus OX=9986 GN=LPP PE=4 SV=2	34.833333	3.773637447	0.0110536	0.0633351
G1T090$$26S proteasome non-ATPase regulatory subunit 14 OS=Homo sapiens GN=PSMD14 PE=1 SV=1	32	3.764245126	0.01116795	0.0637921
G1SZ03$$Uncharacterized protein OS=Homo sapiens PE=4 SV=1	35.133333	3.759165861	0.01123034	0.0639506
G1SLM1$$Enoyl-CoA delta isomerase 1 OS=Oryctolagus cuniculus OX=9986 GN=ECI1 PE=3 SV=1	35.033333	3.745792406	0.01139651	0.0644555

P62943$$Uncharacterized protein OS=Oryctolagus cuniculus OX=9986 GN=CNDP2 PE=4 SV=1	30.566667	3.74428323	0.01141544	0.0644555
G1STQ6$$Ig kappa chain V region K29-213 OS=Oryctolagus cuniculus OX=9986 PE=1 SV=1	42.7	3.742197611	0.01144165	0.0644555
G1TJB7$$Tripartite motif-containing protein 72 OS=Oryctolagus cuniculus OX=9986 GN=TRIM72 PE=1 SV=1	53.866667	3.740840285	0.01145875	0.0644555
G1U6X4$$Ig kappa-b4 chain C region OS=Oryctolagus cuniculus OX=9986 PE=1 SV=1	36.333333	3.737947269	0.01149528	0.0644644
G1T0B4$$Uncharacterized protein OS=Oryctolagus cuniculus OX=9986 PE=4 SV=1	36.5	3.73468473	0.01153664	0.0644854
G1T9I9$$C-terminal-binding protein 1 OS=Homo sapiens GN=CTBP1 PE=1 SV=2	42.8	3.729929163	0.01159723	0.0644854
G1SQB1$$Heterogeneous nuclear ribonucleoprotein K OS=Oryctolagus cuniculus OX=9986 GN=HNRNPK PE=2 SV=1	33.666667	3.729409274	0.01160387	0.0644854
G1TU32$$""Pyruvate dehydrogenase protein X component, mitochondrial OS=Homo sapiens GN=PDHX PE=1 SV=3""	42.433333	3.72545857	0.01165451	0.0645723
G1TG66$$Uncharacterized protein OS=Oryctolagus cuniculus OX=9986 GN=MYO1C PE=3 SV=1	325.70877	3.709896826	0.01185641	0.0653688
G1SZ63$$Casein kinase II subunit beta OS=Homo sapiens GN=CSNK2B PE=1 SV=1	57.1	3.708926272	0.01186914	0.0653688
G1T398$$Perilipin OS=Oryctolagus cuniculus OX=9986 GN=PLIN2 PE=3 SV=2	31.266667	3.698272698	0.0120098	0.0656543
G1T7P9$$Carboxylic ester hydrolase OS=Oryctolagus cuniculus OX=9986 GN=LOC100357214 PE=3 SV=1	63.066667	3.697182323	0.0120243	0.0656543
G1SNT1$$Lysine--tRNA ligase OS=Homo sapiens GN=KARS PE=1 SV=3	30.833333	3.696924652	0.01202773	0.0656543
G1SIW1$$Hypoxanthine phosphoribosyltransferase OS=Oryctolagus cuniculus OX=9986 GN=HPRT PE=2 SV=1	29.333333	3.664648281	0.01246624	0.0678472
G1SJ57$$Exportin 7 OS=Oryctolagus cuniculus OX=9986 GN=XPO7 PE=4 SV=1	38.566667	3.658383037	0.01255343	0.0681208
G1SM77$$Coronin OS=Oryctolagus cuniculus OX=9986 GN=CORO1C PE=3 SV=1	37.333333	4.926266967	0.00283481	0.0056696

G1SPF9$$Uncharacterized protein OS=Oryctolagus cuniculus OX=9986 GN=MYBBP1A PE=4 SV=2	-97.36667	-3.645638206	0.01273292	0.0686907
G1TVW5$$Tropomyosin alpha-4 chain OS=Homo sapiens GN=TPM4 PE=1 SV=3	47.6	3.630178089	0.01295454	0.0696016
G1U415$$Uncharacterized protein OS=Oryctolagus cuniculus OX=9986 GN=HSD17B4 PE=4 SV=2	-48.73333	-3.62861393	0.0129772	0.0696016
G1U3I5$$Uncharacterized protein OS=Oryctolagus cuniculus OX=9986 PE=4 SV=1	70	3.608918107	0.01326642	0.0709465
G1T2V0$$COP9 signalosome subunit 6 OS=Oryctolagus cuniculus OX=9986 GN=COPS6 PE=4 SV=1	35.1	3.605389277	0.013319	0.0710218
G1TKE8$$Monoacylglycerol lipase ABHD12 OS=Homo sapiens GN=ABHD12 PE=1 SV=2	34	3.60062216	0.0133904	0.0711968
G1T161$$Uncharacterized protein OS=Oryctolagus cuniculus OX=9986 GN=NUCKS1 PE=4 SV=1	42.6	3.596861644	0.01344703	0.0712925
G1T361$$Uncharacterized protein OS=Oryctolagus cuniculus OX=9986 GN=VAMP2 PE=4 SV=1	38	3.590538449	0.01354286	0.0715948
G1U502$$Uncharacterized protein OS=Oryctolagus cuniculus OX=9986 GN=HSPE1 PE=3 SV=1	29.366667	3.586881664	0.01359863	0.0716842
G1T4V2$$Paraspeckle component 1 OS=Homo sapiens GN=PSPC1 PE=1 SV=1	54.1	3.574268016	0.01379299	0.0722349
G1T7I3$$Guanine nucleotide-binding protein subunit gamma OS=Oryctolagus cuniculus OX=9986 GN=GNG12 PE=3 SV=1	52.9	3.572877011	0.01381462	0.0722349
G1SW06$$Glucose-6-phosphate isomerase OS=Oryctolagus cuniculus OX=9986 GN=GPI PE=3 SV=1	32.4	3.572495613	0.01382055	0.0722349
G1SGV5$$Uncharacterized protein OS=Oryctolagus cuniculus OX=9986 GN=PSMD3 PE=4 SV=2	34.3	3.554868872	0.01409803	0.0734527
P30946$$Uncharacterized protein OS=Oryctolagus cuniculus OX=9986 GN=PMPCB PE=3 SV=1	41.6	3.55266434	0.01413317	0.0734527
G1U4Q4$$Uncharacterized protein OS=Oryctolagus cuniculus OX=9986 GN=A2M PE=4 SV=2	28.2	3.548253816	0.01420378	0.0736123
G1U4P7$$Isochorismatase domain containing 2 OS=Oryctolagus cuniculus OX=9986 GN=ISOC2 PE=4 SV=1	35.633333	3.544315856	0.01426715	0.0737336
G1U6H4$$Superoxide dismutase [Cu-Zn] OS=Oryctolagus cuniculus OX=9986 GN=SOD1 PE=1 SV=3	-28.53333	-3.540457587	0.01432955	0.0738492

G1T2K1$$Uncharacterized protein OS=Oryctolagus cuniculus OX=9986 PE=3 SV=1	42.5	3.53208223	0.01446605	0.074345
U3KMZ3$$Uncharacterized protein OS=Oryctolagus cuniculus OX=9986 GN=CTSB PE=3 SV=1	77.666667	3.511911988	0.01480083	0.0758542
G1T9M9$$Ras-related protein Rab-1A OS=Homo sapiens GN=RAB1A PE=1 SV=3	27.2	3.505731763	0.01490514	0.0761772
G1SU82$$Acyl-coenzyme A oxidase OS=Oryctolagus cuniculus OX=9986 GN=ACOX1 PE=3 SV=1	-67.9	-3.485074199	0.01525982	0.0777277
G1SMH9$$Syntaxin-binding protein 3 OS=Homo sapiens GN=STXBP3 PE=1 SV=2	46.866667	3.48318317	0.01529276	0.0777277
G1TMV1$$Phosphoribosyl pyrophosphate synthase-associated protein 2 OS=Homo sapiens GN=PRPSAP2 PE=1 SV=1	-70.4	-3.479551805	0.01535623	0.0778359
G1SP32$$Uncharacterized protein OS=Oryctolagus cuniculus OX=9986 GN=PDLIM1 PE=4 SV=1	47.933333	3.472336021	0.01548324	0.0782646
G1SCD7$$ATP-dependent 6-phosphofructokinase OS=Oryctolagus cuniculus OX=9986 GN=PFKM PE=3 SV=1	28.933333	3.459174631	0.01571794	0.0792339
G1SDU8$$Uncharacterized protein OS=Oryctolagus cuniculus OX=9986 GN=NUDT9 PE=4 SV=1	28.1	3.455703736	0.0157805	0.0793325
G1SUE4$$Uncharacterized protein OS=Oryctolagus cuniculus OX=9986 GN=ACADM PE=3 SV=1	-75.13333	-3.451840573	0.01585046	0.079388
P27124$$Solute carrier family 25 member 1 OS=Oryctolagus cuniculus OX=9986 GN=SLC25A1 PE=3 SV=1	44.4	3.448400947	0.01591303	0.079388
G1SL46$$Inosine-5'-monophosphate dehydrogenase OS=Oryctolagus cuniculus OX=9986 GN=IMPDH2 PE=3 SV=1	33.6	3.447800004	0.015924	0.079388
G1SE57$$Dihydrolipoamide acetyltransferase component of pyruvate dehydrogenase complex OS=Oryctolagus cuniculus OX=9986 GN=DBT PE=3 SV=1	28.4	3.445629671	0.01596365	0.079388
G1SS91$$Uncharacterized protein OS=Oryctolagus cuniculus OX=9986 GN=NANS PE=4 SV=1	-47.03333	-3.439452242	0.01607714	0.0796582
P08507$$""ATP synthase-coupling factor 6, mitochondrial OS=Oryctolagus cuniculus OX=9986 GN=ATP5PF PE=3 SV=1""	30.8	3.435846969	0.01614379	0.0796582
G1T2Y5$$Uncharacterized protein OS=Oryctolagus cuniculus OX=9986 GN=HSD17B12 PE=3 SV=1	34.1	3.435645648	0.01614752	0.0796582

G1U276$$Proteasome endopeptidase complex OS=Oryctolagus cuniculus OX=9986 GN=PSMA3 PE=3 SV=2	36.8	3.426436051	0.01631923	0.0802906
G1SKS9$$Cytochrome b5 OS=Oryctolagus cuniculus OX=9986 GN=CYB5A PE=1 SV=4	28.033333	3.419682787	0.01644644	0.0807013
G1T524$$Uncharacterized protein OS=Oryctolagus cuniculus OX=9986 GN=MYL9 PE=4 SV=1	37.866667	3.413940355	0.01655548	0.0810208
G1TKC9$$Uncharacterized protein OS=Oryctolagus cuniculus OX=9986 GN=NARS2 PE=3 SV=1	-100.2667	-3.384580225	0.01712577	0.0835901
G1SXQ1$$Uncharacterized protein OS=Oryctolagus cuniculus OX=9986 PE=4 SV=1	61.833333	3.378548824	0.01724563	0.0839039
G1SPZ7$$2-oxoisovalerate dehydrogenase subunit alpha OS=Oryctolagus cuniculus OX=9986 PE=3 SV=2	27.466667	3.376776391	0.01728103	0.0839039
G1SJ46$$Nucleoside diphosphate kinase OS=Homo sapiens GN=NME1-NME2 PE=1 SV=1	30.9	3.369862847	0.01741988	0.0843561
G1T6P5$$Proteasome endopeptidase complex OS=Oryctolagus cuniculus OX=9986 GN=SORT1 PE=3 SV=2	29.733333	3.364049999	0.0175376	0.0845629
G1SQ01$$Uncharacterized protein OS=Oryctolagus cuniculus OX=9986 GN=LDHD PE=4 SV=1	28.533333	3.363231657	0.01755424	0.0845629
G1T1U7$$Protein DDI1 homolog 2 OS=Homo sapiens GN=DDI2 PE=1 SV=1	30.1	3.354425889	0.01773445	0.0852085
G1TET2$$Uncharacterized protein OS=Oryctolagus cuniculus OX=9986 GN=STT3A PE=4 SV=1	-92.36667	-3.34221183	0.01798784	0.0862014
P46409$$Uncharacterized protein OS=Oryctolagus cuniculus OX=9986 GN=MARC2 PE=4 SV=1	80.433333	3.334945732	0.01814049	0.0867078
G1U5X6$$Prohibitin-2 OS=Homo sapiens GN=PHB2 PE=1 SV=2	40.666667	3.330302192	0.0182388	0.0869524
G1SCN8$$Fermitin family homolog 2 OS=Homo sapiens GN=FERMT2 PE=1 SV=1	30.166667	3.327430936	0.01829989	0.0870188
G1TC61$$Uncharacterized protein OS=Oryctolagus cuniculus OX=9986 GN=BANF1 PE=4 SV=1	37.833333	3.308835053	0.0187011	0.088698
G1SHV9$$Small nuclear ribonucleoprotein Sm D2 OS=Homo sapiens GN=SNRPD2 PE=1 SV=1	27.733333	3.296857113	0.01896471	0.0894919
G1SFF2$$Uncharacterized protein OS=Oryctolagus cuniculus OX=9986 GN=GNA13 PE=4 SV=2	-38	-3.296821583	0.0189655	0.0894919
G1TWK7$$""Malonyl-CoA decarboxylase, mitochondrial OS=Homo sapiens GN=MLYCD PE=1 SV=3""	25.333333	3.281561472	0.01930738	0.0908727

G1TSG1$$Uncharacterized protein OS=Oryctolagus cuniculus OX=9986 GN=NUDT2 PE=4 SV=1	29.533333	3.270003939	0.01957088	0.0918427
G1T7H9$$Uncharacterized protein OS=Oryctolagus cuniculus OX=9986 GN=ARL6IP1 PE=4 SV=1	66.966667	3.268171472	0.01961302	0.0918427
G1SXX5$$DnaJ homolog subfamily B member 1 OS=Homo sapiens GN=DNAJB1 PE=1 SV=4	47.766667	3.258822441	0.01982962	0.0926219
G1SEV2$$""Serine--tRNA ligase, cytoplasmic OS=Oryctolagus cuniculus OX=9986 GN=SARS PE=4 SV=1""	28.533333	3.255543005	0.01990623	0.0927449
G1TM00$$Uncharacterized protein OS=Oryctolagus cuniculus OX=9986 GN=TBCC PE=4 SV=1	27.933333	3.2486468	0.0200684	0.0930654
G1U3L3$$Uncharacterized protein OS=Oryctolagus cuniculus OX=9986 GN=STRN PE=4 SV=1	36.766667	3.246539569	0.02011824	0.0930654
G1STU4$$Uncharacterized protein OS=Oryctolagus cuniculus OX=9986 GN=IDE PE=3 SV=1	-87.76667	-3.246198038	0.02012633	0.0930654
G1SDX3$$Src substrate cortactin OS=Homo sapiens GN=CTTN PE=1 SV=2	68.3	3.220421001	0.02074764	0.095253
G1T4M1$$Gap junction alpha-1 protein OS=Homo sapiens GN=GJA1 PE=1 SV=2	-40.33333	-3.218807903	0.02078722	0.095253
G1SV13$$Uncharacterized protein OS=Oryctolagus cuniculus OX=9986 GN=SPTA1 PE=4 SV=2	28.966667	3.218087121	0.02080494	0.095253
G1SXT1$$X-ray repair cross-complementing protein 6 OS=Homo sapiens GN=XRCC6 PE=1 SV=2	52.533333	3.216098892	0.02085389	0.095253
P00169$$Extracellular superoxide dismutase [Cu-Zn] OS=Oryctolagus cuniculus OX=9986 GN=SOD3 PE=2 SV=2	50.066667	3.215949856	0.02085756	0.095253
G1SUZ1$$Uncharacterized protein OS=Oryctolagus cuniculus OX=9986 GN=LMOD1 PE=4 SV=2	-56.23333	-3.208183753	0.02105003	0.0958459
G1T3V5$$Alpha-parvin OS=Homo sapiens GN=PARVA PE=1 SV=1	37.433333	3.20652927	0.02109129	0.0958459
G1T143$$Glycerol-3-phosphate dehydrogenase [NAD(+)] OS=Oryctolagus cuniculus OX=9986 GN=GPD1 PE=3 SV=1	30.166667	3.202100528	0.02120217	0.096113
U3KLT3$$Uncharacterized protein OS=Oryctolagus cuniculus OX=9986 GN=CRYZ PE=4 SV=1	-39.8	-3.197846781	0.02130929	0.0963618
P02057$$Nucleolin OS=Homo sapiens GN=NCL PE=1 SV=3	-61.9	-3.178811907	0.02179601	0.0983219
G1SV22$$Apolipoprotein E OS=Oryctolagus cuniculus OX=9986 GN=APOE PE=2 SV=1	64.533333	3.160613755	0.0222729	0.1002281

G1TB81$$Uncharacterized protein OS=Oryctolagus cuniculus OX=9986 GN=ENPP1 PE=4 SV=1	108.1	3.148360432	0.02260052	0.1014138
G1SIX1$$Very-long-chain (3R)-3-hydroxyacyl-CoA dehydratase OS=Oryctolagus cuniculus OX=9986 GN=HACD3 PE=3 SV=1	34.2	3.143131701	0.02274195	0.1014138
G1SY96$$Methionine adenosyltransferase 2 subunit beta OS=Oryctolagus cuniculus OX=9986 GN=MAT2B PE=3 SV=2	50.8	3.140353561	0.02281749	0.1014138
P63150$$Haptoglobin OS=Oryctolagus cuniculus OX=9986 GN=HP PE=3 SV=1	30.666667	3.138031601	0.02288084	0.1014138
G1TLH1$$Uncharacterized protein OS=Oryctolagus cuniculus OX=9986 GN=FHL2 PE=4 SV=1	27.7	3.137601401	0.0228926	0.1014138
G1TL73$$Uncharacterized protein OS=Oryctolagus cuniculus OX=9986 GN=HK1 PE=3 SV=2	97.233333	3.137587748	0.02289297	0.1014138
G1T0M2$$Uncharacterized protein OS=Oryctolagus cuniculus OX=9986 GN=GPNMB PE=4 SV=2	38.7	3.136557215	0.02292117	0.1014138
G1TVP3$$Histidine-rich glycoprotein OS=Oryctolagus cuniculus OX=9986 GN=HRG PE=4 SV=2	60.8	3.131743797	0.02305337	0.1017547
G1SZK4$$""VPS37B, ESCRT-I subunit OS=Oryctolagus cuniculus OX=9986 GN=VPS37B PE=4 SV=2""	24.533333	3.107258418	0.02373909	0.1045313
G1T387$$Uncharacterized protein OS=Oryctolagus cuniculus OX=9986 GN=ABCB1 PE=4 SV=1	24.066667	3.093683561	0.02412896	0.1059951
G1SEK8$$Mitochondrial import receptor subunit TOM22 homolog OS=Homo sapiens GN=TOMM22 PE=1 SV=3	33.8	3.086035047	0.02435175	0.1067197
G1TMM5$$Mitogen-activated protein kinase OS=Oryctolagus cuniculus OX=9986 GN=MAPK3 PE=4 SV=1	30.433333	3.04900968	0.02546286	0.1113246
G1SW77$$UV excision repair protein RAD23 homolog A OS=Homo sapiens GN=RAD23A PE=1 SV=1	27.633333	3.041445462	0.02569668	0.1120159
G1SZS0$$Acetyltransferase component of pyruvate dehydrogenase complex OS=Oryctolagus cuniculus OX=9986 GN=DLAT PE=3 SV=1	38.166667	3.039975066	0.02574241	0.1120159
G1SJ56$$Uncharacterized protein OS=Oryctolagus cuniculus OX=9986 GN=GNB1 PE=4 SV=1	-55.6	-3.038024819	0.02580319	0.1120162
G1SRY1$$Uncharacterized protein OS=Oryctolagus cuniculus OX=9986 GN=HNRNPA1 PE=4 SV=1	50.333333	3.034136749	0.02592486	0.1122802

G1T8F7$$Pleckstrin homology domain containing A7 OS=Oryctolagus cuniculus OX=9986 GN=PLEKHA7 PE=4 SV=2	29.966667	3.025973116	0.02618237	0.1129426
G1THY2$$Uncharacterized protein OS=Oryctolagus cuniculus OX=9986 GN=NIT2 PE=4 SV=2	40	3.025409945	0.02620024	0.1129426
P62139$$Uncharacterized protein OS=Oryctolagus cuniculus OX=9986 GN=C1orf123 PE=4 SV=1	27.933333	3.022836603	0.02628206	0.1130312
G1SIM3$$Tubulin alpha-4A chain OS=Homo sapiens GN=TUBA4A PE=1 SV=1	50.6	3.008228621	0.02675189	0.1146405
G1SK25$$Uncharacterized protein OS=Oryctolagus cuniculus OX=9986 GN=EPRS PE=3 SV=1	24.733333	3.007347287	0.02678053	0.1146405
G1TF82$$60S acidic ribosomal protein P0 OS=Homo sapiens GN=RPLP0 PE=1 SV=1	38.8	3.003190459	0.02691607	0.1148405
G1U5I0$$Uncharacterized protein OS=Oryctolagus cuniculus OX=9986 PE=4 SV=1	46.333333	3.002100469	0.02695174	0.1148405
G1T7T6$$Uncharacterized protein OS=Oryctolagus cuniculus OX=9986 GN=NXN PE=4 SV=1	27.366667	2.997666419	0.02709736	0.115195
G1T9V1$$Calcium-regulated heat-stable protein 1 OS=Homo sapiens GN=CARHSP1 PE=1 SV=2	46.233333	2.988947896	0.02738624	0.1161554
G1TJP1$$Uncharacterized protein OS=Oryctolagus cuniculus OX=9986 GN=PAPSS2 PE=3 SV=2	35.533333	2.984995974	0.0275183	0.1164478
G1TTM0$$Uncharacterized protein OS=Oryctolagus cuniculus OX=9986 PE=4 SV=2	29.1	2.983046339	0.0275837	0.1164575
U3KPD5$$Uncharacterized protein OS=Oryctolagus cuniculus OX=9986 GN=TRIM25 PE=4 SV=1	-29.4	-2.973130365	0.02791903	0.1176041
G1SUI9$$Profilin OS=Homo sapiens GN=PFN2 PE=1 SV=1	-60.6	-2.951379075	0.02867041	0.1204941
G1TNV7$$Uncharacterized protein OS=Oryctolagus cuniculus OX=9986 PE=4 SV=1	110.23333	2.947814385	0.02879566	0.1207454
G1SZ72$$Profilin OS=Oryctolagus cuniculus OX=9986 GN=PFN1 PE=3 SV=1	29.233333	2.929697067	0.02944159	0.123174
G1SS07$$60S ribosomal protein L31 OS=Homo sapiens GN=RPL31 PE=1 SV=1	27.933333	2.925400215	0.02959711	0.1235445
G1T8E0$$Peptidylprolyl isomerase OS=Oryctolagus cuniculus OX=9986 GN=FKBP11 PE=3 SV=2	30.433333	2.9198695	0.02979862	0.1241049
G1SI79$$Uncharacterized protein OS=Oryctolagus cuniculus OX=9986 GN=SUOX PE=4 SV=1	85.166667	2.917909818	0.02987038	0.1241236

G1U6H0$$Uncharacterized protein OS=Oryctolagus cuniculus OX=9986 GN=NDUFB9 PE=3 SV=1	29.666667	2.915214136	0.02996941	0.1242552
G1SS94$$Serine/arginine-rich splicing factor 2 OS=Homo sapiens GN=SRSF2 PE=1 SV=4	43.8	2.911610475	0.03010234	0.1245265
G1SSK8$$Chloride intracellular channel protein OS=Oryctolagus cuniculus OX=9986 GN=CLIC1 PE=3 SV=1	41.2	3.388105182	0.01529445	0.0194814
G1TRV4$$Transportin-1 OS=Homo sapiens GN=TNPO1 PE=1 SV=2	112.73333	2.89476091	0.03073257	0.1265661
G1T5N7$$Uncharacterized protein OS=Oryctolagus cuniculus OX=9986 GN=FBN1 PE=4 SV=1	40.966667	2.89164778	0.03085059	0.1266709
G1T7R2$$Uncharacterized protein OS=Oryctolagus cuniculus OX=9986 GN=LAMP2 PE=3 SV=2	24.633333	2.890470439	0.03089535	0.1266709
G1SXN3$$Nuclear transport factor 2 OS=Homo sapiens GN=NUTF2 PE=1 SV=1	-46.33333	-2.886022078	0.03106512	0.1268267
G1T970$$Ubiquitin-conjugating enzyme E2 D3 OS=Homo sapiens GN=UBE2D3 PE=1 SV=1	39	2.88587332	0.03107082	0.1268267
G1SNB5$$Uncharacterized protein OS=Oryctolagus cuniculus OX=9986 PE=4 SV=1	23.566667	2.867202559	0.03179476	0.1294952
P00389$$Uncharacterized protein OS=Oryctolagus cuniculus OX=9986 GN=EFTUD2 PE=4 SV=1	29.333333	2.86516491	0.03187488	0.1295356
G1TJY2$$Glutaredoxin 3 OS=Oryctolagus cuniculus OX=9986 GN=GLRX3 PE=4 SV=2	31	2.860704123	0.03205105	0.1297268
G1T2K2$$Uncharacterized protein OS=Oryctolagus cuniculus OX=9986 GN=ATP6V1E1 PE=3 SV=1	49.466667	2.858459645	0.0321401	0.1297268
G1SF78$$Sulfurtransferase OS=Oryctolagus cuniculus OX=9986 GN=TST PE=4 SV=1	48.666667	2.858348427	0.03214452	0.1297268
G1TLE3$$Uncharacterized protein OS=Oryctolagus cuniculus OX=9986 GN=RPL4 PE=1 SV=2	22.9	2.856873631	0.03220318	0.1297268
G1T3H5$$Guanine nucleotide-binding protein G(s) subunit alpha isoforms XLas OS=Homo sapiens GN=GNAS PE=1 SV=2	25.833333	2.851891346	0.03240224	0.1298544
G1U446$$Uncharacterized protein OS=Oryctolagus cuniculus OX=9986 GN=CSAD PE=3 SV=1	26.766667	2.851606378	0.03241367	0.1298544
G1TSU1$$Uncharacterized protein OS=Oryctolagus cuniculus OX=9986 GN=PTER PE=3 SV=1	37.6	2.849532839	0.03249694	0.1298544
G1SIF2$$Voltage-dependent anion-selective channel protein 2 OS=Oryctolagus cuniculus OX=9986 GN=VDAC2 PE=4 SV=1	25.133333	2.849049448	0.03251639	0.1298544

G1SIZ2$$Acetyl-CoA acyltransferase 1 OS=Oryctolagus cuniculus OX=9986 GN=ACAA1 PE=3 SV=1	28.133333	2.838700122	0.0329358	0.1312453
G1T3D7$$Uncharacterized protein OS=Oryctolagus cuniculus OX=9986 GN=ANP32A PE=4 SV=2	23.2	2.835160462	0.03308061	0.1315382
G1SHI9$$4-trimethylaminobutyraldehyde dehydrogenase OS=Homo sapiens GN=ALDH9A1 PE=1 SV=3	38.566667	3.339057707	0.01623447	0.0194814
G1SLY0$$Prefoldin subunit 4 OS=Homo sapiens GN=PFDN4 PE=1 SV=1	-33.2	-2.821591519	0.03364219	0.1331971
G1U8X8$$Uncharacterized protein OS=Oryctolagus cuniculus OX=9986 GN=SARNP PE=4 SV=1	-95.23333	-2.815275421	0.03390714	0.1339586
G1T7L5$$Electron transfer flavoprotein subunit alpha OS=Oryctolagus cuniculus OX=9986 GN=ETFA PE=4 SV=2	53.666667	2.808515674	0.03419323	0.1348002
P01377$$Transmembrane 9 superfamily member OS=Oryctolagus cuniculus OX=9986 GN=TM9SF2 PE=3 SV=2	65.133333	2.803634177	0.03440147	0.1350819
G1SVA9$$Glutathione S-transferase OS=Oryctolagus cuniculus OX=9986 PE=3 SV=1	48.633333	2.80075952	0.03452474	0.1350819
G1TA83$$Uncharacterized protein OS=Oryctolagus cuniculus OX=9986 GN=CDK5RAP3 PE=4 SV=2	-72.5	-2.800350561	0.03454232	0.1350819
G1SHZ8$$Endoplasmin OS=Homo sapiens GN=HSP90B1 PE=1 SV=1	28.366667	2.798373356	0.03462744	0.1350819
G1TEU8$$Uncharacterized protein OS=Oryctolagus cuniculus OX=9986 GN=OPA1 PE=3 SV=1	41.466667	2.796276778	0.03471794	0.1350819
G1TI55$$Histone H1.3 OS=Oryctolagus cuniculus OX=9986 PE=1 SV=1	26.533333	2.796125058	0.0347245	0.1350819
G1TEM7$$Uncharacterized protein OS=Oryctolagus cuniculus OX=9986 GN=RPS18 PE=1 SV=1	-50.8	-2.793110149	0.03485513	0.1350819
G1SXU2$$Uncharacterized protein OS=Oryctolagus cuniculus OX=9986 GN=TWF2 PE=4 SV=2	33.033333	2.792184727	0.03489534	0.1350819
G1SPR7$$Phosphatidylinositol transfer protein beta isoform OS=Homo sapiens GN=PITPNB PE=1 SV=1	-102.5333	-2.790852751	0.03495329	0.1350819
G1SMM7$$Uncharacterized protein OS=Oryctolagus cuniculus OX=9986 GN=MGARP PE=4 SV=1	32.466667	2.789853693	0.03499683	0.1350819
G1U9Q9$$Uncharacterized protein OS=Oryctolagus cuniculus OX=9986 GN=NPC2 PE=4 SV=1	-54.3	-2.78746094	0.03510135	0.1352025

G1SSN2$$Superoxide dismutase OS=Oryctolagus cuniculus OX=9986 PE=3 SV=1	61.066667	2.774809471	0.03565966	0.1368409
G1T0Q0$$Succinate-semialdehyde dehydrogenase OS=Oryctolagus cuniculus OX=9986 GN=ALDH5A1 PE=3 SV=2	-114.3333	-2.774463772	0.03567505	0.1368409
G1U0Z7$$Adenosylhomocysteinase OS=Oryctolagus cuniculus OX=9986 GN=AHCYL2 PE=3 SV=2	21.833333	2.770222938	0.03586445	0.137282
G1SWS6$$COP9 signalosome complex subunit 1 OS=Homo sapiens GN=GPS1 PE=1 SV=1	-58.13333	-2.766167875	0.03604658	0.1376935
G1TV19$$Uncharacterized protein OS=Oryctolagus cuniculus OX=9986 GN=DDAH2 PE=4 SV=1	35.166667	2.755921098	0.03651131	0.138948
G1SER8$$Uncharacterized protein OS=Oryctolagus cuniculus OX=9986 GN=NUP210 PE=4 SV=1	-33.5	-2.75505129	0.03655106	0.138948
G1T5N5$$Clathrin interactor 1 OS=Homo sapiens GN=CLINT1 PE=1 SV=1	74.5	2.753000487	0.03664497	0.138948
G1TD13$$Oxysterol-binding protein 1 OS=Oryctolagus cuniculus OX=9986 GN=OSBP PE=1 SV=1	28.7	2.752318641	0.03667625	0.138948
G1TGT5$$Clathrin heavy chain OS=Oryctolagus cuniculus OX=9986 GN=CLTC PE=3 SV=2	45.5	2.747716882	0.03688811	0.1394643
G1TU12$$""ATP-dependent 6-phosphofructokinase, liver type OS=Homo sapiens GN=PFKL PE=1 SV=6""	33.833333	2.730268801	0.03770362	0.1420954
G1TX94$$Serine/threonine-protein kinase OSR1 OS=Homo sapiens GN=OXSR1 PE=1 SV=1	35.166667	2.729540664	0.03773807	0.1420954
G1SN83$$Citrate synthase (Fragment) OS=Homo sapiens GN=CS PE=1 SV=1	-46.56667	-2.722085695	0.03809283	0.143139
G1SWW8$$Uncharacterized protein OS=Oryctolagus cuniculus OX=9986 GN=SRSF3 PE=4 SV=1	23	2.71967132	0.03820849	0.1432818
G1TBW1$$60S ribosomal protein L24 OS=Homo sapiens GN=RPL24 PE=1 SV=1	21.366667	2.716114845	0.03837957	0.1436315
G1STB6$$Caveolin-1 OS=Oryctolagus cuniculus OX=9986 GN=CAV1 PE=3 SV=1	-93.86667	-2.712013671	0.03857788	0.1440779
Q9GKX2$$Protein SET OS=Homo sapiens GN=SET PE=1 SV=3	59.3	2.710424366	0.03865504	0.1440779
G1SR07$$""Isobutyryl-CoA dehydrogenase, mitochondrial OS=Homo sapiens GN=ACAD8 PE=1 SV=1""	73.733333	2.706518576	0.03884535	0.1444953
G1TL92$$Uncharacterized protein OS=Oryctolagus cuniculus OX=9986 GN=MYOF PE=4 SV=2	-185.1667	-2.693461239	0.03948901	0.1465002

G1T701$$Citrate synthase OS=Oryctolagus cuniculus OX=9986 GN=CS PE=3 SV=1	34.4	2.691651108	0.03957916	0.1465002
G1SMY1$$Heterogeneous nuclear ribonucleoprotein A3 OS=Homo sapiens GN=HNRNPA3 PE=1 SV=2	40.1	2.690781454	0.03962254	0.1465002
G1TAK1$$Actin-related protein 3 OS=Homo sapiens GN=ACTR3 PE=1 SV=3	25.733333	2.684338064	0.03994561	0.1473993
G1SUU7$$Uncharacterized protein OS=Oryctolagus cuniculus OX=9986 GN=AIFM1 PE=4 SV=1	27.066667	2.673014267	0.04052033	0.1485999
G1T332$$Perilipin-4 OS=Homo sapiens GN=PLIN4 PE=1 SV=1	33.7	2.671549679	0.04059532	0.1485999
G1T5W4$$Uncharacterized protein OS=Oryctolagus cuniculus OX=9986 GN=VPS26A PE=4 SV=2	23.6	2.671193937	0.04061356	0.1485999
O46503$$Angiotensin-converting enzyme OS=Oryctolagus cuniculus OX=9986 GN=ACE PE=1 SV=3	48.933333	2.671153663	0.04061562	0.1485999
A0A140TAV7$$Patatin like phospholipase domain containing 6 OS=Oryctolagus cuniculus OX=9986 GN=PNPLA6 PE=4 SV=1	39.766667	2.67002218	0.04067369	0.1485999
G1TES6$$Uncharacterized protein OS=Oryctolagus cuniculus OX=9986 GN=BOLA1 PE=3 SV=1	56.9	2.66490274	0.04093753	0.1492683
G1SPV2$$Elongation factor 1-alpha 1 OS=Oryctolagus cuniculus OX=9986 GN=EEF1A1 PE=1 SV=1	24.9	2.661534471	0.04111214	0.1493809
G1SM62$$Uncharacterized protein OS=Oryctolagus cuniculus OX=9986 GN=MVP PE=4 SV=1	80.866667	2.661183963	0.04113035	0.1493809
G1TCY4$$Lumican OS=Oryctolagus cuniculus OX=9986 GN=LUM PE=4 SV=1	25.2	2.640939305	0.04219746	0.1529554
G1SE67$$Uncharacterized protein OS=Oryctolagus cuniculus OX=9986 GN=ZNF207 PE=4 SV=1	21.2	2.639054415	0.04229833	0.1530204
G1T302$$Low-density lipoprotein receptor OS=Oryctolagus cuniculus OX=9986 GN=LDLR PE=4 SV=1	-21.86667	-2.631755014	0.04269143	0.1541403
G1TSP3$$N-acetylglucosamine-6-sulfatase OS=Oryctolagus cuniculus OX=9986 GN=GNS PE=3 SV=1	44.533333	2.626227595	0.04299172	0.1549213
G1SYS5$$Dolichol-phosphate mannosyltransferase subunit 3 OS=Oryctolagus cuniculus OX=9986 GN=DPM3 PE=3 SV=1	42.1	2.624561106	0.04308271	0.1549466
G1SMT7$$Ig mu chain C region membrane-bound form OS=Oryctolagus cuniculus OX=9986 PE=2 SV=2	29.2	2.621123958	0.04327103	0.1553211
G1TQP6$$Uncharacterized protein OS=Oryctolagus cuniculus OX=9986 GN=PRPS1 PE=3 SV=1	22.266667	2.617221149	0.04348594	0.1557894

G1TEJ1$$Uncharacterized protein OS=Oryctolagus cuniculus OX=9986 PE=4 SV=2	34.866667	2.61443846	0.04363987	0.1560379
G1T2C4$$Cofilin 2 OS=Oryctolagus cuniculus OX=9986 GN=CFL2 PE=3 SV=1	-108.5333	-2.604413919	0.04419932	0.1577326
G1TBU8$$Uncharacterized protein OS=Oryctolagus cuniculus OX=9986 GN=DHRS11 PE=3 SV=1	-36.66667	-2.601779033	0.04434765	0.1579564
Q01971$$Acylphosphatase OS=Oryctolagus cuniculus OX=9986 GN=ACYP2 PE=3 SV=1	21.5	2.593358681	0.04482528	0.15935
P16973$$Desmin OS=Homo sapiens GN=DES PE=1 SV=3	99.7	2.585979848	0.0452484	0.1605448
G1T845$$TAR DNA-binding protein 43 OS=Homo sapiens GN=TARDBP PE=1 SV=1	26.633333	2.582584593	0.04544454	0.1609312
G1SZE0$$Signal recognition particle subunit SRP68 OS=Homo sapiens GN=SRP68 PE=1 SV=2	21.533333	2.578967072	0.04565453	0.1613651
U3KNG6$$Serum paraoxonase/arylesterase 1 OS=Oryctolagus cuniculus OX=9986 GN=PON1 PE=1 SV=2	46.066667	2.573163039	0.04599363	0.1622529
G1TLD8$$Phospholipase D family member 3 OS=Oryctolagus cuniculus OX=9986 GN=PLD3 PE=4 SV=2	31.6	2.558651105	0.04685342	0.1649705
G1SHX1$$AP complex subunit sigma OS=Homo sapiens GN=AP2S1 PE=1 SV=1	29.2	2.552076912	0.04724861	0.1660451
G1SHD6$$Y-box binding protein 3 OS=Oryctolagus cuniculus OX=9986 GN=YBX3 PE=4 SV=1	22.933333	2.549513268	0.04740369	0.1662734
G1SKN0$$""Dihydrolipoyl dehydrogenase, mitochondrial OS=Homo sapiens GN=DLD PE=1 SV=2""	48.4	2.547230922	0.04754222	0.1664429
G1SPE6$$Calpain-2 catalytic subunit OS=Oryctolagus cuniculus OX=9986 GN=CAPN2 PE=3 SV=1	24.833333	2.537324718	0.04814852	0.1682463
G1T9V7$$3-hydroxy-3-methylglutaryl coenzyme A synthase OS=Oryctolagus cuniculus OX=9986 GN=HMGCS1 PE=3 SV=2	36.166667	2.535160978	0.04828206	0.168394
G1SUH5$$Uncharacterized protein OS=Oryctolagus cuniculus OX=9986 PE=4 SV=1	22.633333	2.526480839	0.04882177	0.169955
G1SV32$$Dehydrogenase/reductase 7B OS=Oryctolagus cuniculus OX=9986 GN=DHRS7B PE=3 SV=1	47.033333	2.523876388	0.04898498	0.170202
G1T7S1$$Aldehyde dehydrogenase family 16 member A1 OS=Oryctolagus cuniculus OX=9986 GN=ALDH16A1 PE=3 SV=1	60.2	2.520316658	0.04920899	0.170659

G1T8M9$$Uncharacterized protein OS=Oryctolagus cuniculus OX=9986 GN=TMED9 PE=3 SV=1	33.666667	2.51584006	0.04949226	0.1712503
G1TY77$$Biphenyl hydrolase like OS=Oryctolagus cuniculus OX=9986 GN=BPHL PE=4 SV=1	28.8	2.514346786	0.04958714	0.1712503
Q8SQG9$$Uncharacterized protein OS=Oryctolagus cuniculus OX=9986 GN=MCCC2 PE=4 SV=1	32.866667	2.513234451	0.04965795	0.1712503
G1U450$$Serum albumin OS=Homo sapiens GN=ALB PE=1 SV=2	36.833333	2.507652187	0.05001492	0.1720142
G1SSL0$$Uncharacterized protein OS=Oryctolagus cuniculus OX=9986 GN=PRKACB PE=3 SV=2	32.8	2.506858166	0.05006592	0.1720142
G1SIS2$$Uncharacterized protein OS=Oryctolagus cuniculus OX=9986 GN=LOC100340393 PE=4 SV=2	48.666667	2.502455274	0.05034973	0.1726677
G1U5U0$$EMILIN-1 OS=Homo sapiens GN=EMILIN1 PE=1 SV=3	28.6	2.4999406	0.0505126	0.1729049
G1T7L0$$Uncharacterized protein OS=Oryctolagus cuniculus OX=9986 GN=GOLIM4 PE=4 SV=2	-83.33333	-2.497310544	0.05068355	0.1731688
G1TPB1$$Uncharacterized protein OS=Oryctolagus cuniculus OX=9986 GN=NCALD PE=4 SV=1	25.633333	2.480364346	0.0518	0.1766562
G1U7P6$$Early endosome antigen 1 OS=Oryctolagus cuniculus OX=9986 GN=EEA1 PE=4 SV=1	22.566667	2.47602137	0.05209035	0.1773186
G1TAF8$$Small ubiquitin-related modifier OS=Oryctolagus cuniculus OX=9986 PE=3 SV=1	28.566667	2.470754717	0.05244479	0.1781964
G1TLK9$$ADP dependent glucokinase OS=Oryctolagus cuniculus OX=9986 GN=ADPGK PE=4 SV=1	35.966667	2.467898355	0.0526381	0.1785244
G1TCR0$$Basigin OS=Oryctolagus cuniculus OX=9986 GN=BSG PE=2 SV=1	38.833333	2.465989431	0.05276772	0.1786357
G1TNZ3$$Uncharacterized protein OS=Oryctolagus cuniculus OX=9986 GN=ILK PE=4 SV=1	27.7	2.456505981	0.05341673	0.1804128
G1T310$$Uncharacterized protein OS=Oryctolagus cuniculus OX=9986 GN=DCTN2 PE=4 SV=2	45.033333	2.455468735	0.05348823	0.1804128
G1SXZ9$$Uncharacterized protein OS=Oryctolagus cuniculus OX=9986 GN=PCCB PE=4 SV=1	33.033333	2.453991528	0.05359024	0.180427
G1SWS9$$Uncharacterized protein OS=Oryctolagus cuniculus OX=9986 GN=MIF PE=4 SV=1	-88.83333	-2.444494426	0.05425104	0.1823191
G1T8W7$$Thioredoxin reductase 2 OS=Oryctolagus cuniculus OX=9986 GN=TXNRD2 PE=3 SV=2	29.333333	2.436724939	0.05479812	0.1835512
G1SQI2$$Bisphosphoglycerate mutase OS=Oryctolagus cuniculus OX=9986 GN=BPGM PE=2 SV=2	-34.03333	-2.436463294	0.05481664	0.1835512

G1TE20$$Uncharacterized protein OS=Oryctolagus cuniculus OX=9986 GN=IARS2 PE=3 SV=2	23.833333	2.426337918	0.05553872	0.1856321
G1STU7$$Uncharacterized protein (Fragment) OS=Homo sapiens PE=4 SV=2	20.166667	2.408563787	0.05683094	0.1896077
G1TPN3$$Magnesium transporter protein 1 OS=Homo sapiens GN=MAGT1 PE=1 SV=1	40.633333	2.375166007	0.05934651	0.1976432
G1THF1$$""NADH dehydrogenase [ubiquinone] iron-sulfur protein 6, mitochondrial OS=Oryctolagus cuniculus OX=9986 GN=NDUFS6 PE=3 SV=1""	60.866667	2.357555872	0.06072041	0.2018543
G1SF06$$Uncharacterized protein OS=Oryctolagus cuniculus OX=9986 GN=DNAJB2 PE=4 SV=2	48.766667	2.354071136	0.06099626	0.2024067
G1SVT0$$Globin A1 OS=Oryctolagus cuniculus OX=9986 GN=HBB2 PE=3 SV=1	24.033333	2.3480297	0.06147767	0.2036379
G1SZW0$$Uncharacterized protein OS=Oryctolagus cuniculus OX=9986 GN=ARHGAP17 PE=4 SV=1	72.433333	2.340026724	0.06212159	0.205
G1SRE3$$Pyruvate kinase PKM OS=Homo sapiens GN=PKM PE=1 SV=4	31.033333	2.3387101	0.06222821	0.205
P55057$$Dystroglycan OS=Oryctolagus cuniculus OX=9986 GN=DAG1 PE=1 SV=1	-29.13333	-2.337893977	0.06229439	0.205
G1T2V6$$Uncharacterized protein OS=Oryctolagus cuniculus OX=9986 GN=PDLIM4 PE=4 SV=1	20.8	2.33741418	0.06233334	0.205
G1T550$$Uncharacterized protein OS=Oryctolagus cuniculus OX=9986 GN=RBP1 PE=3 SV=1	55.866667	2.335563299	0.06248382	0.2051293
G1SVK2$$Uncharacterized protein OS=Oryctolagus cuniculus OX=9986 PE=4 SV=2	22.9	2.330916749	0.0628633	0.2060085
G1SPB6$$Actin-related protein 2/3 complex subunit 1B OS=Homo sapiens GN=ARPC1B PE=1 SV=3	21.533333	2.328719844	0.06304356	0.2062329
Q1XH18$$Uncharacterized protein OS=Oryctolagus cuniculus OX=9986 GN=TMEM43 PE=4 SV=2	33.4	2.317452225	0.06397678	0.208702
G1U9S6$$Uncharacterized protein OS=Oryctolagus cuniculus OX=9986 GN=ACTN4 PE=4 SV=2	20.7	2.316880017	0.06402456	0.208702
G1SI29$$Adenosylhomocysteinase OS=Oryctolagus cuniculus OX=9986 GN=AHCY PE=3 SV=2	33.866667	2.309276624	0.06466305	0.2104115
G1SRP8$$Uncharacterized protein OS=Oryctolagus cuniculus OX=9986 GN=IST1 PE=4 SV=2	20.533333	2.305501676	0.06498255	0.2110789
G1U522$$Peptidylprolyl isomerase OS=Oryctolagus cuniculus OX=9986 GN=FKBP9 PE=4 SV=1	34.166667	2.303084182	0.06518804	0.2113742

G1TIQ4$$S-formylglutathione hydrolase OS=Oryctolagus cuniculus OX=9986 GN=ESD PE=3 SV=1	33.366667	2.293222717	0.06603341	0.2137397
G1T813$$Uncharacterized protein OS=Oryctolagus cuniculus OX=9986 GN=C4A PE=4 SV=1	50.266667	2.28697655	0.06657485	0.2150274
G1T8D7$$Profilin-2 OS=Homo sapiens GN=PFN2 PE=1 SV=3	-102.9667	-2.285949442	0.06666433	0.2150274
G1TI39$$Uncharacterized protein OS=Oryctolagus cuniculus OX=9986 GN=NNT PE=4 SV=2	44.066667	2.283921759	0.06684136	0.2152222
G1TKS5$$Glutathione S-transferase OS=Oryctolagus cuniculus OX=9986 GN=LOC100356307 PE=3 SV=2	22.466667	2.278098956	0.06735248	0.2162939
G1SG29$$Ezrin OS=Oryctolagus cuniculus OX=9986 GN=EZR PE=1 SV=3	26.266667	2.275889588	0.06754749	0.2162939
G1TFE0$$Uncharacterized protein OS=Oryctolagus cuniculus OX=9986 GN=NUMA1 PE=4 SV=2	26.6	2.274456881	0.06767427	0.2162939
G1TEA3$$Ribulose-phosphate 3-epimerase OS=Oryctolagus cuniculus OX=9986 GN=RPE PE=3 SV=1	-41.23333	-2.273316634	0.06777535	0.2162939
G1T2I5$$Uncharacterized protein OS=Oryctolagus cuniculus OX=9986 GN=LOC100358239 PE=4 SV=2	61.433333	2.272970948	0.06780602	0.2162939
G1TMC5$$""Threonine--tRNA ligase, cytoplasmic OS=Homo sapiens GN=TARS PE=1 SV=3""	-22.5	-2.272164852	0.06787761	0.2162939
G1T373$$Uncharacterized protein OS=Oryctolagus cuniculus OX=9986 GN=LOC100344509 PE=4 SV=2	17.3	2.268373175	0.06821541	0.2169956
G1T3Z6$$Cystatin B OS=Oryctolagus cuniculus OX=9986 GN=CSTB PE=4 SV=1	20.8	2.262219921	0.06876738	0.2175694
G1TCZ0$$Uncharacterized protein OS=Oryctolagus cuniculus OX=9986 GN=LOC100353361 PE=3 SV=1	42.433333	2.26182348	0.06880311	0.2175694
G1SCI0$$Quinoid dihydropteridine reductase OS=Oryctolagus cuniculus OX=9986 GN=QDPR PE=2 SV=2	27.966667	2.261235276	0.06885615	0.2175694
G1T379$$Aldehyde dehydrogenase family 1 member A3 OS=Homo sapiens GN=ALDH1A3 PE=1 SV=2	34.933333	2.259270537	0.06903362	0.2175694
G1SCI5$$Uncharacterized protein OS=Oryctolagus cuniculus OX=9986 GN=GSPT1 PE=4 SV=1	-19.83333	-2.258715201	0.06908387	0.2175694
G1TKT9$$Uncharacterized protein OS=Oryctolagus cuniculus OX=9986 GN=LOC100356907 PE=4 SV=1	18.433333	2.258500265	0.06910333	0.2175694

P09212$$Uncharacterized protein OS=Oryctolagus cuniculus OX=9986 GN=LOC100008830 PE=3 SV=1	81.933333	2.245261141	0.07031316	0.2210013
Q28719$$Arp2/3 complex 34 kDa subunit OS=Oryctolagus cuniculus OX=9986 GN=ARPC2 PE=3 SV=1	41.233333	2.239747825	0.07082353	0.2222269
U3KNL5$$Peptidyl-prolyl cis-trans isomerase B OS=Homo sapiens GN=PPIB PE=1 SV=2	36.066667	2.237674183	0.0710165	0.2224541
G1U724$$Glutathione peroxidase OS=Homo sapiens GN=GPX4 PE=1 SV=3	19.7	2.235730632	0.07119787	0.2226442
G1TZX4$$Uncharacterized protein OS=Oryctolagus cuniculus OX=9986 GN=CAVIN3 PE=4 SV=1	78.3	2.230584066	0.07168048	0.2237741
G1TN25$$Uncharacterized protein OS=Oryctolagus cuniculus OX=9986 GN=HADHA PE=3 SV=1	-27.73333	-2.21809993	0.07286551	0.2270893
G1U9T4$$Serine/threonine-protein phosphatase OS=Oryctolagus cuniculus OX=9986 GN=PPP4C PE=3 SV=1	25.633333	2.212841428	0.0733708	0.2282785
G1SYV0$$Uncharacterized protein OS=Oryctolagus cuniculus OX=9986 PE=3 SV=1	22.766667	2.20952215	0.07369165	0.2288907
P01696$$Annexin OS=Oryctolagus cuniculus OX=9986 GN=ANXA4 PE=3 SV=2	-83.7	-2.208184447	0.07382137	0.2289083
G1U9U2$$Potassium channel tetramerization domain containing 12 OS=Oryctolagus cuniculus OX=9986 GN=KCTD12 PE=4 SV=2	32.4	2.200725722	0.07454906	0.2307769
G1SXW0$$Uncharacterized protein OS=Oryctolagus cuniculus OX=9986 GN=ARHGAP1 PE=4 SV=2	31.833333	2.195658515	0.07504771	0.2319314
G1SQG5$$Spermine synthase OS=Homo sapiens GN=SMS PE=1 SV=2	21.433333	2.179596009	0.07665162	0.2364921
G1SZT8$$Programmed cell death 6-interacting protein OS=Homo sapiens GN=PDCD6IP PE=1 SV=1	19.333333	2.176712915	0.07694329	0.2369956
G1TDD2$$Heat shock protein family A (Hsp70) member 5 OS=Oryctolagus cuniculus OX=9986 GN=HSPA5 PE=3 SV=1	38.166667	2.17347248	0.0772725	0.2376129
G1SKW5$$Cytochrome c oxidase subunit OS=Oryctolagus cuniculus OX=9986 GN=COX6B1 PE=3 SV=1	34.433333	2.161075313	0.07854561	0.2411259
G1SEX0$$Uncharacterized protein OS=Oryctolagus cuniculus OX=9986 GN=FABP4 PE=3 SV=1	72.6	2.159125293	0.07874785	0.2413452
G1T9V4$$Uncharacterized protein OS=Oryctolagus cuniculus OX=9986 PE=3 SV=2	27.5	2.156849051	0.07898462	0.2416693

G1SHF1$$Uncharacterized protein OS=Oryctolagus cuniculus OX=9986 GN=DPYSL2 PE=4 SV=1	23.633333	2.155578675	0.07911708	0.2416739
G1TPV0$$Uncharacterized protein OS=Oryctolagus cuniculus OX=9986 GN=FYCO1 PE=4 SV=1	31.4	2.140460642	0.08071134	0.2457739
G1SL85$$Uncharacterized protein OS=Oryctolagus cuniculus OX=9986 GN=ECH1 PE=3 SV=1	72.866667	2.140325503	0.08072574	0.2457739
G1U2R2$$Uncharacterized protein OS=Oryctolagus cuniculus OX=9986 GN=CTSZ PE=3 SV=1	22.9	2.138045328	0.08096913	0.2461088
G1TD99$$Uncharacterized protein OS=Oryctolagus cuniculus OX=9986 GN=PRKAR2B PE=4 SV=1	47.3	2.132995619	0.08151088	0.247348
G1TCX0$$Uncharacterized protein OS=Oryctolagus cuniculus OX=9986 GN=HGD PE=4 SV=2	60.8	2.128635423	0.08198169	0.2483682
G1SY70$$Uncharacterized protein OS=Oryctolagus cuniculus OX=9986 GN=DNPEP PE=3 SV=2	17.133333	2.125595472	0.08231162	0.2489589
G1SW57$$Uncharacterized protein OS=Oryctolagus cuniculus OX=9986 GN=PGM5 PE=3 SV=1	-103.5667	-2.12290561	0.0826047	0.2494365
G1TRK3$$Alpha-galactosidase OS=Oryctolagus cuniculus OX=9986 GN=NAGA PE=3 SV=1	-18.86667	-2.118824202	0.08305148	0.2503758
G1TB36$$Phosphoglycerate kinase OS=Oryctolagus cuniculus OX=9986 GN=LOC100348124 PE=3 SV=2	-67.76667	-2.113461566	0.08364234	0.2517457
G1TN89$$Uncharacterized protein OS=Oryctolagus cuniculus OX=9986 GN=RPL8 PE=1 SV=1	-92.5	-2.111734306	0.08383358	0.2519104
G1TV79$$""[Pyruvate dehydrogenase (acetyl-transferring)] kinase isozyme 2, mitochondrial OS=Homo sapiens GN=PDK2 PE=1 SV=2""	-65.63333	-2.110221715	0.08400143	0.2520043
G1SF82$$Histone H2AX OS=Homo sapiens GN=H2AFX PE=1 SV=2	36.9	2.099198436	0.08523522	0.2552906
G1SZL3$$""Persulfide dioxygenase ETHE1, mitochondrial OS=Homo sapiens GN=ETHE1 PE=1 SV=1""	85.566667	2.096503471	0.08553971	0.2557873
O18757$$Metallothionein-3 OS=Homo sapiens GN=MT3 PE=1 SV=1	23.766667	2.092051548	0.08604519	0.2564596
G1SUD2$$""Metaxin 1, isoform CRA_b OS=Homo sapiens GN=MTX1 PE=1 SV=1""	59.4	2.090693362	0.08620002	0.2564596
G1TVQ6$$Uncharacterized protein OS=Oryctolagus cuniculus OX=9986 PE=4 SV=2	-21.23333	-2.089891082	0.08629161	0.2564596
G1U866$$Serum albumin OS=Oryctolagus cuniculus OX=9986 GN=ALB PE=1 SV=1	37.2	2.089637715	0.08632056	0.2564596

G1SHL8$$Uncharacterized protein OS=Oryctolagus cuniculus OX=9986 GN=ATP5F1E PE=4 SV=1	42.566667	2.086346035	0.08669754	0.2571655
G1U5Y9$$Uncharacterized protein OS=Oryctolagus cuniculus OX=9986 GN=OGDHL PE=4 SV=2	-19.56667	-2.082497789	0.08714042	0.2576282
G1SXG6$$UDP-glucose:glycoprotein glucosyltransferase 2 OS=Homo sapiens GN=UGGT2 PE=1 SV=4	110.53333	2.081705631	0.08723188	0.2576282
G1T432$$Uncharacterized protein OS=Oryctolagus cuniculus OX=9986 PE=4 SV=2	-33.6	-2.081202665	0.08729	0.2576282
G1TBS8$$Uncharacterized protein OS=Oryctolagus cuniculus OX=9986 GN=CYP51A1 PE=3 SV=1	-43.36667	-2.080147472	0.08741207	0.2576282
G1T7G3$$Uncharacterized protein OS=Oryctolagus cuniculus OX=9986 GN=MX1 PE=3 SV=1	23.533333	2.076443977	0.0878419	0.2584821
G1SCF4$$Uncharacterized protein OS=Oryctolagus cuniculus OX=9986 GN=FHL1 PE=4 SV=2	29.133333	2.073219158	0.08821795	0.2591754
G1T4D2$$Uncharacterized protein OS=Oryctolagus cuniculus OX=9986 GN=CYP21A2 PE=3 SV=1	23.933333	2.065352028	0.08914237	0.2614748
G1U0Y6$$Uncharacterized protein OS=Oryctolagus cuniculus OX=9986 GN=LARS PE=3 SV=2	56.533333	2.057976991	0.09001807	0.2636243
G1T7H0$$Eukaryotic initiation factor 4A-I OS=Oryctolagus cuniculus OX=9986 GN=EIF4A1 PE=3 SV=1	25.033333	2.056415096	0.09020466	0.2636644
G1SUK4$$Glycerol-3-phosphate dehydrogenase OS=Oryctolagus cuniculus OX=9986 GN=GPD2 PE=3 SV=2	17.933333	2.055471657	0.09031757	0.2636644
G1T0I5$$Collagen alpha-1(VI) chain OS=Homo sapiens GN=COL6A1 PE=1 SV=3	46.233333	2.050827769	0.09087545	0.2648739
G1U8T9$$Glycylpeptide N-tetradecanoyltransferase OS=Oryctolagus cuniculus OX=9986 GN=NMT1 PE=3 SV=2	46.133333	2.047477821	0.09128009	0.265181
G1SKJ4$$Uncharacterized protein OS=Oryctolagus cuniculus OX=9986 GN=PCK2 PE=3 SV=1	22.2	2.047013699	0.0913363	0.265181
G1TDJ3$$Uncharacterized protein OS=Oryctolagus cuniculus OX=9986 GN=AHSA1 PE=4 SV=1	16.566667	2.046389335	0.09141197	0.265181
G1T4X4$$Uncharacterized protein OS=Oryctolagus cuniculus OX=9986 GN=VWA8 PE=4 SV=2	41.633333	2.043255074	0.09179281	0.2658564
G1SPY1$$Calcium-transporting ATPase OS=Oryctolagus cuniculus OX=9986 GN=ATP2B4 PE=3 SV=1	27.066667	2.042104687	0.09193301	0.2658564

G1T587$$Thioredoxin OS=Oryctolagus cuniculus OX=9986 GN=LOC100359245 PE=3 SV=1	34.3	2.030306898	0.09338358	0.2696286
G1SUF4$$Uncharacterized protein OS=Oryctolagus cuniculus OX=9986 PE=4 SV=1	45	2.025072683	0.09403468	0.2710621
G1T501$$Glutathione S-transferase kappa OS=Oryctolagus cuniculus OX=9986 GN=GSTK1 PE=3 SV=1	23.033333	2.023522096	0.09422846	0.2710621
G1SVQ8$$LIM and SH3 domain protein 1 OS=Oryctolagus cuniculus OX=9986 GN=LASP1 PE=4 SV=1	42.233333	2.022784337	0.0943208	0.2710621
G1T9L4$$MICOS complex subunit OS=Oryctolagus cuniculus OX=9986 GN=APOOL PE=4 SV=1	-21.86667	-2.019724896	0.09470474	0.2714319
U3KPC4$$Far upstream element-binding protein 2 OS=Homo sapiens GN=KHSRP PE=1 SV=1	67.7	2.018975816	0.09479899	0.2714319
G1T188$$Prefoldin subunit 3 OS=Oryctolagus cuniculus OX=9986 PE=3 SV=1	26.766667	2.017206225	0.09502202	0.2714319
G1SEX5$$AP-3 complex subunit beta OS=Oryctolagus cuniculus OX=9986 GN=AP3B1 PE=3 SV=1	-18.33333	-2.017079942	0.09503796	0.2714319
G1TXI0$$Uncharacterized protein OS=Oryctolagus cuniculus OX=9986 GN=GLIPR2 PE=3 SV=1	32.933333	2.014783679	0.09532822	0.2718401
G1SER3$$Putative RNA-binding protein Luc7-like 2 OS=Homo sapiens GN=LUC7L2 PE=4 SV=1	74.866667	2.011693264	0.09572032	0.272537
G1SZD6$$Uncharacterized protein OS=Oryctolagus cuniculus OX=9986 GN=MGST1 PE=4 SV=1	26.7	1.999829772	0.09724097	0.2761218
G1SY50$$Uncharacterized protein OS=Oryctolagus cuniculus OX=9986 GN=CSRP1 PE=4 SV=1	20.266667	1.999537935	0.09727869	0.2761218
G1SK42$$Uncharacterized protein OS=Oryctolagus cuniculus OX=9986 GN=MTDH PE=4 SV=1	-63.2	-1.9953361	0.09782342	0.2772415
G1SVA3$$Uncharacterized protein OS=Oryctolagus cuniculus OX=9986 PE=1 SV=1	18.766667	1.982117099	0.09955768	0.2817238
G1U1W4$$N-acylethanolamine-hydrolyzing acid amidase OS=Oryctolagus cuniculus OX=9986 GN=NAAA PE=1 SV=2	23.033333	1.966756099	0.10161261	0.2870984
G1SIN4$$Uncharacterized protein OS=Oryctolagus cuniculus OX=9986 GN=TCP1 PE=3 SV=1	69.833333	1.959060439	0.10265837	0.2895902
G1TIZ1$$Uncharacterized protein OS=Oryctolagus cuniculus OX=9986 GN=TXNDC12 PE=4 SV=2	28.766667	1.957112022	0.10292489	0.2895902
O46373$$Malate dehydrogenase OS=Oryctolagus cuniculus OX=9986 GN=MDH2 PE=3 SV=1	30.033333	1.95681638	0.10296539	0.2895902

G1TA21$$""Electron transfer flavoprotein subunit alpha, mitochondrial OS=Homo sapiens GN=ETFA PE=1 SV=1""	24.5	1.951394762	0.10371101	0.2912432
G1TUD6$$Uncharacterized protein OS=Oryctolagus cuniculus OX=9986 GN=LRRC59 PE=4 SV=1	41.333333	1.94921654	0.10401212	0.2916449
G1SYA5$$Uncharacterized protein OS=Oryctolagus cuniculus OX=9986 GN=LOC100348005 PE=3 SV=1	17.766667	1.945016121	0.10459531	0.2928351
G1SIE8$$Dolichyl-diphosphooligosaccharide--protein glycosyltransferase subunit 1 OS=Oryctolagus cuniculus OX=9986 GN=RPN1 PE=3 SV=1	43	1.94073349	0.10519335	0.2940632
G1T667$$Uncharacterized protein OS=Oryctolagus cuniculus OX=9986 PE=4 SV=1	15.866667	1.937350298	0.10566825	0.2949439
G1TDK5$$Uncharacterized protein OS=Oryctolagus cuniculus OX=9986 GN=STIP1 PE=4 SV=2	17.166667	1.935142386	0.10597936	0.2953654
G1TVT1$$Creatine kinase B-type OS=Oryctolagus cuniculus OX=9986 GN=CKB PE=3 SV=2	18.733333	1.93333754	0.10623437	0.2954899
G1T182$$Uncharacterized protein OS=Oryctolagus cuniculus OX=9986 GN=DDX39B PE=4 SV=1	24.233333	1.932560571	0.10634434	0.2954899
G1SIJ2$$Vesicle amine transport 1 OS=Oryctolagus cuniculus OX=9986 GN=VAT1 PE=4 SV=1	27.366667	1.930741754	0.10660222	0.2956205
G1T958$$Purine nucleoside phosphorylase OS=Oryctolagus cuniculus OX=9986 GN=PNP PE=3 SV=1	45	1.929970241	0.1067118	0.2956205
G1SL49$$Serpin H1 OS=Homo sapiens GN=SERPINH1 PE=1 SV=2	-15.4	-1.920363638	0.10808594	0.2989783
G1SWF3$$Uncharacterized protein OS=Oryctolagus cuniculus OX=9986 GN=HINT2 PE=4 SV=1	48.033333	1.913184205	0.10912464	0.3013996
G1SH85$$Neurofilament light polypeptide OS=Homo sapiens GN=NEFL PE=1 SV=3	17.2	1.908369686	0.10982687	0.3028858
G1SZM0$$Uncharacterized protein OS=Oryctolagus cuniculus OX=9986 GN=SLC25A5 PE=3 SV=1	75.166667	1.906266751	0.11013503	0.3032823
G1T6I6$$Uncharacterized protein OS=Oryctolagus cuniculus OX=9986 GN=ATP6V1B2 PE=3 SV=1	29.566667	1.901076016	0.11089946	0.3049113
G1T671$$Uncharacterized protein OS=Oryctolagus cuniculus OX=9986 GN=HDGF PE=4 SV=1	33.966667	2.114487292	0.08006506	0.0800651
G1TV92$$Uncharacterized protein OS=Oryctolagus cuniculus OX=9986 GN=PRKAR2A PE=4 SV=2	29.733333	1.898894152	0.11122238	0.3049113

G1T8S4$$ELKS/Rab6-interacting/CAST family member 1 OS=Homo sapiens GN=ERC1 PE=1 SV=1	42.733333	1.897654455	0.11140628	0.3049623
G1U315$$Uncharacterized protein OS=Oryctolagus cuniculus OX=9986 GN=EMC2 PE=4 SV=1	-58.43333	-1.895301521	0.11175618	0.3054452
G1TAX9$$Carboxylic ester hydrolase OS=Oryctolagus cuniculus OX=9986 GN=LOC100357214 PE=3 SV=1	23.8	1.893448158	0.11203257	0.3054452
G1TBW9$$Uncharacterized protein OS=Oryctolagus cuniculus OX=9986 GN=SF3B2 PE=4 SV=2	38.533333	1.89313495	0.11207935	0.3054452
G1SYJ4$$""Tricarboxylate transport protein, mitochondrial OS=Homo sapiens GN=SLC25A1 PE=1 SV=2""	29.833333	1.888003257	0.11284858	0.307088
G1TI64$$40S ribosomal protein S2 OS=Homo sapiens GN=RPS2 PE=1 SV=2	-31.5	-1.883104799	0.11358785	0.3086445
G1SPJ2$$Cell cycle and apoptosis regulator 2 OS=Oryctolagus cuniculus OX=9986 GN=CCAR2 PE=4 SV=2	29.233333	1.881423055	0.11384279	0.3088823
G1TX91$$Uncharacterized protein OS=Oryctolagus cuniculus OX=9986 GN=LRP1 PE=4 SV=1	25.6	1.877136764	0.11449517	0.3098571
G1SI22$$Uncharacterized protein OS=Oryctolagus cuniculus OX=9986 GN=PSME2 PE=4 SV=1	36.366667	1.875333464	0.11477076	0.3098571
G1SM64$$AP-2 complex subunit alpha OS=Oryctolagus cuniculus OX=9986 GN=AP2A1 PE=3 SV=1	-58.36667	-1.874968717	0.11482659	0.3098571
G1SPJ5$$Uncharacterized protein OS=Oryctolagus cuniculus OX=9986 PE=4 SV=1	-65.26667	-1.874659994	0.11487386	0.3098571
G1SNK5$$NADH dehydrogenase [ubiquinone] 1 alpha subcomplex subunit 6 OS=Homo sapiens GN=NDUFA6 PE=1 SV=3	66.1	1.872805877	0.11515818	0.3101706
G1U6B2$$Uncharacterized protein OS=Oryctolagus cuniculus OX=9986 PE=4 SV=2	40.1	1.869883866	0.1156077	0.3103845
G1U5Y2$$Malic enzyme OS=Oryctolagus cuniculus OX=9986 GN=ME1 PE=3 SV=2	-70.36667	-1.869773201	0.11562476	0.3103845
G1SQK3$$Eukaryotic initiation factor 4A-II OS=Homo sapiens GN=EIF4A2 PE=1 SV=2	50.666667	1.868339733	0.11584597	0.3103845
G1SUY2$$Ras-related protein Rab-12 OS=Homo sapiens GN=RAB12 PE=1 SV=3	35.2	1.867921901	0.11591053	0.3103845
P67777$$60S ribosomal protein L13a (Fragment) OS=Homo sapiens GN=RPL13A PE=1 SV=2	50.6	1.866516776	0.1161279	0.3105159
G1TIT1$$Uncharacterized protein OS=Oryctolagus cuniculus OX=9986 GN=YWHAG PE=3 SV=1	17.333333	1.863351086	0.11661913	0.3109157

G1SPN9$$Fructose-bisphosphate aldolase A OS=Oryctolagus cuniculus OX=9986 GN=ALDOA PE=1 SV=2	29.966667	1.862574841	0.11673991	0.3109157
G1TUP7$$Putative cytochrome b-c1 complex subunit Rieske-like protein 1 OS=Homo sapiens GN=UQCRFS1P1 PE=5 SV=1	23.2	1.862298137	0.11678299	0.3109157
G1T0L1$$NEDD8-activating enzyme E1 regulatory subunit OS=Oryctolagus cuniculus OX=9986 GN=NAE1 PE=3 SV=2	17.466667	1.858366573	0.11739686	0.3120997
G1SE61$$Uncharacterized protein OS=Oryctolagus cuniculus OX=9986 GN=RANBP2 PE=4 SV=2	-46.06667	-1.853490126	0.1181628	0.313684
G1SGY8$$Eukaryotic translation initiation factor 2 subunit 3 OS=Oryctolagus cuniculus OX=9986 GN=EIF2S3 PE=1 SV=2	23.033333	1.851508717	0.11847546	0.3140621
G1TYR3$$Uncharacterized protein OS=Oryctolagus cuniculus OX=9986 GN=LZTFL1 PE=4 SV=2	-24.06667	-1.845407469	0.11944345	0.3161738
G1U8J5$$Ras-related protein Rab-11B OS=Homo sapiens GN=RAB11B PE=1 SV=4	35.066667	1.839785712	0.12034243	0.3180971
G1TFE8$$Uncharacterized protein OS=Oryctolagus cuniculus OX=9986 GN=CCDC134 PE=4 SV=1	15.433333	1.830298663	0.12187498	0.3216872
G1T7S5$$Uncharacterized protein OS=Oryctolagus cuniculus OX=9986 GN=LOC100346274 PE=4 SV=1	48.1	1.826430072	0.12250554	0.3228896
G1TQX1$$40S ribosomal protein S15a OS=Homo sapiens GN=RPS15A PE=1 SV=2	15.066667	1.823874549	0.12292387	0.3231556
P80456$$Ribosomal protein S6 kinase OS=Homo sapiens GN=RPS6KA2 PE=1 SV=1	17.4	1.823562863	0.12297499	0.3231556
G1SQV5$$Uncharacterized protein OS=Oryctolagus cuniculus OX=9986 GN=LOC100344160 PE=4 SV=1	20.1	1.822606991	0.1231319	0.3231556
G1SVN0$$Uncharacterized protein OS=Oryctolagus cuniculus OX=9986 GN=CCT8 PE=3 SV=2	51.366667	1.821416467	0.12332761	0.3232094
G1SWW7$$Uncharacterized protein OS=Oryctolagus cuniculus OX=9986 GN=BCKDHB PE=4 SV=1	18.533333	1.817587536	0.12395916	0.3244038
G1TLW0$$Uncharacterized protein OS=Oryctolagus cuniculus OX=9986 GN=LAMB2 PE=4 SV=1	21.133333	1.81315241	0.12469476	0.325089
G1T6S2$$Carnitine O-acetyltransferase OS=Oryctolagus cuniculus OX=9986 GN=CRAT PE=4 SV=1	-14.4	-1.812787092	0.12475555	0.325089

G1U711$$Uncharacterized protein OS=Oryctolagus cuniculus OX=9986 GN=PRDX6 PE=4 SV=1	50.933333	1.812690464	0.12477163	0.325089
G1SL02$$Uncharacterized protein OS=Oryctolagus cuniculus OX=9986 GN=MAGED2 PE=4 SV=2	26.466667	1.811764763	0.12492581	0.325089
G1T7T2$$Uncharacterized protein OS=Oryctolagus cuniculus OX=9986 GN=TRIM47 PE=4 SV=2	59.9	1.809220516	0.12535056	0.3257349
G1T6I0$$RuvB-like helicase OS=Oryctolagus cuniculus OX=9986 GN=RUVBL2 PE=3 SV=2	44.666667	1.80650943	0.12580475	0.3260475
G1U0A2$$Eukaryotic translation initiation factor 5A-1 OS=Homo sapiens GN=EIF5A PE=1 SV=1	66.066667	1.805877674	0.12591083	0.3260475
G1TKQ2$$Calpain-1 catalytic subunit OS=Oryctolagus cuniculus OX=9986 GN=CAPN1 PE=3 SV=1	22.3	1.805340888	0.12600103	0.3260475
G1T9D7$$40S ribosomal protein S3a OS=Oryctolagus cuniculus OX=9986 GN=RPS3A PE=3 SV=1	30.066667	1.792476723	0.12818217	0.331227
G1SZF7$$Uncharacterized protein OS=Oryctolagus cuniculus OX=9986 GN=SQOR PE=4 SV=1	-50.1	-1.789409944	0.1287077	0.3319543
P07452$$L-xylulose reductase OS=Homo sapiens GN=DCXR PE=1 SV=2	-16.03333	-1.787169303	0.12909302	0.3319543
G1T4F9$$Uncharacterized protein OS=Oryctolagus cuniculus OX=9986 GN=XPNPEP3 PE=3 SV=1	-31.53333	-1.786888143	0.12914146	0.3319543
G1SQH0$$Probable ATP-dependent RNA helicase DDX17 OS=Homo sapiens GN=DDX17 PE=1 SV=1	31.066667	1.786067493	0.12928292	0.3319543
G1TDB3$$Uncharacterized protein OS=Oryctolagus cuniculus OX=9986 GN=COL6A2 PE=4 SV=1	45.9	1.785602039	0.12936323	0.3319543
G1SZH0$$Elongation factor 1-gamma OS=Oryctolagus cuniculus OX=9986 GN=EEF1G PE=2 SV=3	-77	-1.778139379	0.13065762	0.3348102
G1SGP1$$Alpha-2-HS-glycoprotein OS=Oryctolagus cuniculus OX=9986 GN=AHSG PE=4 SV=1	51.5	1.774831885	0.13123543	0.3358244
G1SZJ5$$60S ribosomal protein L19 OS=Homo sapiens GN=RPL19 PE=1 SV=1	43.9	1.773496437	0.13146945	0.3359572
G1SIP1$$""NADH dehydrogenase [ubiquinone] flavoprotein 1, mitochondrial OS=Homo sapiens GN=NDUFV1 PE=1 SV=4""	20.3	1.766478688	0.13270608	0.3386483
G1SQM7$$Peptidylprolyl isomerase D OS=Oryctolagus cuniculus OX=9986 GN=PPID PE=4 SV=1	44.4	1.763173987	0.13329242	0.3396748

G1TA50$$Uncharacterized protein OS=Oryctolagus cuniculus OX=9986 GN=SORBS1 PE=4 SV=1	26.4	1.759931008	0.13387033	0.3406769
G1SHT4$$Prostaglandin E synthase 3 OS=Homo sapiens GN=PTGES3 PE=1 SV=1	62.933333	1.756860925	0.13441972	0.3415577
G1TMU2$$Elongin B OS=Oryctolagus cuniculus OX=9986 GN=ELOB PE=4 SV=2	14.633333	1.755930243	0.13458671	0.3415577
G1SPY9$$Calpain small subunit 1 OS=Homo sapiens GN=CAPNS1 PE=1 SV=1	34.7	1.750786425	0.13551337	0.343437
G1U8Y9$$Cathepsin K OS=Homo sapiens GN=CTSK PE=1 SV=1	67.333333	1.745681313	0.13643933	0.3453094
G1SL28$$Biglycan OS=Oryctolagus cuniculus OX=9986 GN=BGN PE=3 SV=1	52.266667	1.741681855	0.13716914	0.3466809
G1T567$$Platelet-activating factor acetylhydrolase IB subunit alpha OS=Oryctolagus cuniculus OX=9986 GN=RAP1GAP2 PE=3 SV=1	60.833333	1.731389001	0.1390652	0.3499471
G1TST2$$Nuclear pore complex protein Nup93 OS=Homo sapiens GN=NUP93 PE=1 SV=1	36.333333	1.731114113	0.1391162	0.3499471
B6V9S9$$Uncharacterized protein OS=Oryctolagus cuniculus OX=9986 GN=RPL35 PE=1 SV=1	24.266667	1.731035166	0.13913085	0.3499471
U3KNY1$$Uncharacterized protein OS=Oryctolagus cuniculus OX=9986 GN=CDC42 PE=4 SV=1	49.2	1.730554012	0.13922015	0.3499471
G1SD89$$Uncharacterized protein OS=Oryctolagus cuniculus OX=9986 GN=RPL3 PE=1 SV=1	-108.5333	-1.726138157	0.14004246	0.3515352
G1T2K5$$Procollagen galactosyltransferase 1 OS=Homo sapiens GN=COLGALT1 PE=1 SV=1	37.1	1.718848198	0.14141051	0.3544869
G1TD98$$Uncharacterized protein OS=Oryctolagus cuniculus OX=9986 GN=SUB1 PE=4 SV=2	24.1	1.712505469	0.14261155	0.3567914
G1TP25$$Uncharacterized protein OS=Oryctolagus cuniculus OX=9986 GN=EHD3 PE=3 SV=1	25.5	1.711953516	0.14271654	0.3567914
G1SIB0$$Uncharacterized protein OS=Oryctolagus cuniculus OX=9986 GN=NDUFA4 PE=4 SV=1	68.933333	1.709440076	0.14319561	0.3575046
G1T2X0$$""1-phosphatidylinositol 4,5-bisphosphate phosphodiesterase beta-1 OS=Homo sapiens GN=PLCB1 PE=1 SV=1""	40.766667	1.704642131	0.14411453	0.359142
G1SZR8$$Uncharacterized protein OS=Oryctolagus cuniculus OX=9986 GN=ADK PE=4 SV=1	39.866667	1.703985264	0.14424079	0.359142
G1TUX2$$Phosphomevalonate kinase OS=Oryctolagus cuniculus OX=9986 GN=PMVK PE=4 SV=1	21.366667	1.699667373	0.14507346	0.3607285

G1SSV4$$O-acetyl-ADP-ribose deacetylase MACROD1 OS=Homo sapiens GN=MACROD1 PE=1 SV=2	39	1.696289809	0.1457281	0.3618686
G1SJZ4$$Uncharacterized protein OS=Oryctolagus cuniculus OX=9986 GN=RPL7 PE=1 SV=1	80.8	1.679980228	0.1489304	0.3688931
G1SKK1$$Acyl-CoA synthetase bubblegum family member 1 OS=Oryctolagus cuniculus OX=9986 GN=ACSBG1 PE=4 SV=1	75.5	1.679846941	0.14895685	0.3688931
G1SCE4$$Mitochondrial proton/calcium exchanger protein OS=Homo sapiens GN=LETM1 PE=1 SV=1	23.866667	1.676971252	0.14952871	0.369813
G1T7Y5$$Uncharacterized protein OS=Oryctolagus cuniculus OX=9986 GN=RPS16 PE=1 SV=1	63.133333	1.674013584	0.15011913	0.3699418
A0A0B4J1Q3$$Ig gamma chain C region OS=Oryctolagus cuniculus OX=9986 PE=1 SV=1	-15.76667	-1.673384206	0.15024506	0.3699418
G1SDM2$$Uncharacterized protein OS=Oryctolagus cuniculus OX=9986 GN=PDLIM7 PE=4 SV=1	43.066667	1.672759475	0.15037017	0.3699418
G1SN26$$Poly(rC)-binding protein 3 OS=Homo sapiens GN=PCBP3 PE=2 SV=2	25.8	1.67213115	0.15049609	0.3699418
G1SYE0$$MICOS complex subunit MIC60 OS=Oryctolagus cuniculus OX=9986 GN=IMMT PE=3 SV=2	-16.6	-1.671695962	0.15058338	0.3699418
G1SNC4$$14-3-3 protein theta OS=Oryctolagus cuniculus OX=9986 GN=YWHAQ PE=3 SV=1	40.733333	1.66351318	0.15223378	0.3728125
G1T3U1$$Uncharacterized protein OS=Oryctolagus cuniculus OX=9986 GN=DSTN PE=3 SV=1	-18.93333	-1.663392109	0.15225833	0.3728125
G1T026$$Uncharacterized protein OS=Oryctolagus cuniculus OX=9986 GN=TMED5 PE=3 SV=1	19.5	1.66290046	0.15235807	0.3728125
G1SK04$$60S ribosomal protein L27 OS=Oryctolagus cuniculus OX=9986 GN=LOC100356974 PE=1 SV=1	90.4	1.659895346	0.15296911	0.3738119
G1SSP0$$26S proteasome non-ATPase regulatory subunit 2 OS=Oryctolagus cuniculus OX=9986 GN=PSMD2 PE=3 SV=2	22.366667	1.6566293	0.15363593	0.3749448
G1T7D0$$Glutathione synthetase OS=Oryctolagus cuniculus OX=9986 GN=GSS PE=3 SV=1	22.5	1.649292305	0.15514429	0.3781258
G1SN06$$Uncharacterized protein OS=Oryctolagus cuniculus OX=9986 GN=SCARB2 PE=3 SV=1	14.966667	1.644691599	0.1560975	0.3799471
G1SWD1$$Uncharacterized protein OS=Oryctolagus cuniculus OX=9986 GN=PAICS PE=3 SV=1	31.233333	1.639487327	0.15718266	0.381852

G1SZR7$$Protein transport protein SEC23 OS=Oryctolagus cuniculus OX=9986 GN=SEC23A PE=3 SV=1	28.166667	1.638954998	0.15729407	0.381852
G1SNV4$$Uncharacterized protein OS=Oryctolagus cuniculus OX=9986 GN=HSD17B8 PE=4 SV=1	105.63333	1.637000495	0.15770379	0.3820868
G1ST51$$EH domain-containing protein 2 OS=Homo sapiens GN=EHD2 PE=1 SV=2	18.466667	1.636270577	0.15785707	0.3820868
G1SD49$$Uncharacterized protein OS=Oryctolagus cuniculus OX=9986 GN=ACAT2 PE=4 SV=2	15.366667	1.635533358	0.15801203	0.3820868
U3KLX7$$Electron transfer flavoprotein subunit beta OS=Oryctolagus cuniculus OX=9986 GN=ETFB PE=4 SV=1	-17.63333	-1.628326035	0.15953481	0.385264
G1SHK8$$S-methyl-5'-thioadenosine phosphorylase OS=Homo sapiens GN=MTAP PE=1 SV=1	25.633333	1.626351023	0.15995459	0.385692
G1U0J7$$Uncharacterized protein OS=Oryctolagus cuniculus OX=9986 GN=GCDH PE=3 SV=1	30.7	1.625111597	0.16021857	0.385692
G1TB68$$""Cholesterol side-chain cleavage enzyme, mitochondrial OS=Oryctolagus cuniculus OX=9986 GN=CYP11A1 PE=3 SV=2""	22.966667	1.624546099	0.16033915	0.385692
G1T0F6$$Peptidyl-prolyl cis-trans isomerase A OS=Oryctolagus cuniculus OX=9986 GN=PPIA PE=2 SV=3	50.333333	1.622994218	0.16067053	0.385753
G1T4S6$$Sorting nexin OS=Homo sapiens GN=SNX6 PE=1 SV=1	34.833333	1.621693817	0.16094871	0.385753
G1TDQ3$$Sulfurtransferase OS=Oryctolagus cuniculus OX=9986 GN=MPST PE=4 SV=1	13.766667	1.619930567	0.16132666	0.385753
G1T139$$Uncharacterized protein OS=Oryctolagus cuniculus OX=9986 GN=RRBP1 PE=4 SV=1	36.233333	1.619858037	0.16134223	0.385753
G1T237$$Uncharacterized protein OS=Oryctolagus cuniculus OX=9986 GN=HARS PE=3 SV=1	39.8	1.619542617	0.16140993	0.385753
G1T0W8$$Signal recognition particle subunit SRP72 OS=Oryctolagus cuniculus OX=9986 GN=SRP72 PE=3 SV=2	-101.1667	-1.612304036	0.16297136	0.3889808
G1TVH9$$Aldose 1-epimerase OS=Oryctolagus cuniculus OX=9986 GN=GALM PE=3 SV=1	-47.26667	-1.607731533	0.16396524	0.3906958
G1T823$$Uncharacterized protein OS=Oryctolagus cuniculus OX=9986 GN=RAI14 PE=4 SV=1	-54.33333	-1.607052225	0.1641134	0.3906958
G1TRH5$$40S ribosomal protein S19 OS=Homo sapiens GN=RPS19 PE=1 SV=2	-81.36667	-1.601504145	0.1653283	0.3926226

G1SK12$$Uncharacterized protein (Fragment) OS=Homo sapiens PE=4 SV=1	-47.23333	-1.60141273	0.16534839	0.3926226
G1TRY5$$Uncharacterized protein OS=Oryctolagus cuniculus OX=9986 GN=PRELP PE=4 SV=1	-43.46667	-1.591355317	0.16757325	0.3973942
G1T6E8$$Ubiquitinyl hydrolase 1 OS=Oryctolagus cuniculus OX=9986 GN=USP5 PE=3 SV=2	43	1.588076926	0.16830473	0.3986165
G1TER3$$Uncharacterized protein OS=Oryctolagus cuniculus OX=9986 GN=PITRM1 PE=4 SV=1	18.133333	1.584545521	0.16909612	0.3999774
P15253$$""Aconitate hydratase, mitochondrial OS=Oryctolagus cuniculus OX=9986 GN=ACO2 PE=3 SV=2""	27.6	1.573415407	0.17161396	0.4049345
G1T9P2$$Glutathione S-transferase alpha I OS=Oryctolagus cuniculus OX=9986 PE=1 SV=1	33.766667	1.57334159	0.17163078	0.4049345
Q9N0Z6$$Carboxylic ester hydrolase OS=Oryctolagus cuniculus OX=9986 GN=LOC100351739 PE=3 SV=1	19.633333	1.565098937	0.17351887	0.4088663
U3KNR1$$Acetyl-coenzyme A synthetase OS=Oryctolagus cuniculus OX=9986 GN=ACSS1 PE=3 SV=1	27.866667	1.557129324	0.17536343	0.412426
G1TV31$$Na(+)/H(+) exchange regulatory cofactor NHE-RF1 OS=Oryctolagus cuniculus OX=9986 GN=SLC9A3R1 PE=1 SV=3	-43.06667	-1.556642868	0.17547663	0.412426
G1T8Y0$$26S proteasome non-ATPase regulatory subunit 11 OS=Homo sapiens GN=PSMD11 PE=1 SV=3	43.333333	1.553895602	0.17611724	0.413405
G1TGA8$$Heterogeneous nuclear ribonucleoprotein H OS=Homo sapiens GN=HNRNPH1 PE=1 SV=1	15.4	1.551986563	0.17656371	0.4134528
U3KNR7$$Uncharacterized protein OS=Oryctolagus cuniculus OX=9986 GN=PSAP PE=4 SV=2	33.433333	1.551283065	0.17672851	0.4134528
G1SQR6$$Coiled-coil domain-containing protein 6 OS=Homo sapiens GN=CCDC6 PE=1 SV=2	-36.63333	-1.550935947	0.17680988	0.4134528
G1SY53$$Rho GDP-dissociation inhibitor 1 OS=Homo sapiens GN=ARHGDIA PE=1 SV=1	13	1.548957368	0.17727439	0.4135606
G1TBR1$$Uncharacterized protein OS=Oryctolagus cuniculus OX=9986 GN=CLPX PE=4 SV=1	60.4	1.548830188	0.17730429	0.4135606
G1TB05$$Eukaryotic translation initiation factor 2 subunit 1 OS=Oryctolagus cuniculus OX=9986 GN=EIF2S1 PE=1 SV=1	33.166667	1.54327878	0.17861405	0.4160895
P68105$$""Keratin, type II cuticular Hb5 OS=Homo sapiens GN=KRT85 PE=1 SV=1""	12.566667	1.541201264	0.17910658	0.4167108

G1SYS6$$Uncharacterized protein OS=Oryctolagus cuniculus OX=9986 GN=NT5E PE=3 SV=1	54.266667	1.53708691	0.18008586	0.4177358
G1TEM5$$Uncharacterized protein OS=Oryctolagus cuniculus OX=9986 GN=LOC100349005 PE=4 SV=1	-19	-1.534436164	0.18071949	0.4177358
G1SDD0$$Nucleoside diphosphate kinase OS=Oryctolagus cuniculus OX=9986 GN=NME1 PE=3 SV=1	13.833333	1.533778071	0.18087713	0.4177358
G1TDI4$$Uncharacterized protein OS=Oryctolagus cuniculus OX=9986 GN=MARS PE=3 SV=2	-51.06667	-1.533513164	0.18094062	0.4177358
G1STI3$$Uncharacterized protein OS=Oryctolagus cuniculus OX=9986 GN=PSMC2 PE=3 SV=1	14.2	1.533195936	0.18101668	0.4177358
P16258$$Uncharacterized protein OS=Oryctolagus cuniculus OX=9986 GN=ACSL4 PE=4 SV=1	24.766667	1.53319567	0.18101674	0.4177358
G1SNE1$$Uncharacterized protein OS=Oryctolagus cuniculus OX=9986 GN=ITGA6 PE=3 SV=2	15.166667	1.532595309	0.18116078	0.4177358
G1U0V2$$Uncharacterized protein OS=Oryctolagus cuniculus OX=9986 GN=OCIAD1 PE=4 SV=1	-15.4	-1.531645137	0.18138895	0.4177358
G1U9T1$$Uncharacterized protein OS=Oryctolagus cuniculus OX=9986 GN=TNKS1BP1 PE=4 SV=1	15.666667	1.530830209	0.18158487	0.4177358
G1SPN1$$Uncharacterized protein OS=Oryctolagus cuniculus OX=9986 GN=LAMA2 PE=4 SV=2	14.5	1.526913741	0.18252927	0.4193854
G1SXR1$$Uncharacterized protein OS=Oryctolagus cuniculus OX=9986 GN=RARS PE=3 SV=2	-97.5	-1.52127779	0.18389653	0.4216459
P14519$$Serine/arginine-rich splicing factor 6 OS=Homo sapiens GN=SRSF6 PE=1 SV=2	32.1	1.520975426	0.18397016	0.4216459
G1T5T8$$Coronin-1B OS=Oryctolagus cuniculus OX=9986 GN=CORO1B PE=1 SV=1	-51.8	-1.517756849	0.18475566	0.4229208
G1T4S5$$Uncharacterized protein OS=Oryctolagus cuniculus OX=9986 GN=PNMT PE=4 SV=1	27.5	1.515021662	0.18542569	0.4238991
G1TSN0$$Uncharacterized protein OS=Oryctolagus cuniculus OX=9986 GN=ERP44 PE=4 SV=1	-61.96667	-1.513482155	0.18580385	0.4238991
G1SPF5$$Uncharacterized protein OS=Oryctolagus cuniculus OX=9986 GN=QARS PE=3 SV=1	21.766667	1.513203797	0.1858723	0.4238991
G1U5Z3$$Rho-associated protein kinase 2 OS=Homo sapiens GN=ROCK2 PE=1 SV=4	58.833333	1.509037837	0.18689964	0.4255378
G1SRQ2$$Hepatocyte growth factor-regulated tyrosine kinase substrate OS=Homo sapiens GN=HGS PE=1 SV=1	74.4	1.507999078	0.18715664	0.4255378

G1U8D4$$Uncharacterized protein OS=Oryctolagus cuniculus OX=9986 GN=LANCL1 PE=4 SV=1	-94.37031	-1.507489892	0.18728274	0.4255378
G1T2I4$$Uncharacterized protein OS=Oryctolagus cuniculus OX=9986 GN=CCS PE=4 SV=1	32.333333	1.504579956	0.18800495	0.42646
G1U159$$Hsp90 co-chaperone Cdc37 OS=Homo sapiens GN=CDC37 PE=1 SV=1	55	1.50332913	0.18831621	0.42646
G1SG80$$Uncharacterized protein OS=Oryctolagus cuniculus OX=9986 GN=EBP PE=4 SV=1	-14.23333	-1.503064787	0.18838205	0.42646
O19048$$Dopamine beta-hydroxylase OS=Homo sapiens GN=DBH PE=1 SV=3	19.866667	1.496438618	0.19003963	0.4292821
G1TJW1$$Uncharacterized protein OS=Oryctolagus cuniculus OX=9986 PE=3 SV=2	41.2	1.496222048	0.19009404	0.4292821
G1TAB7$$Pyruvate dehydrogenase E1 component subunit alpha OS=Oryctolagus cuniculus OX=9986 GN=PDHA1 PE=4 SV=1	24.3	1.491380016	0.19131439	0.4315098
G1SF08$$Protein S100 OS=Oryctolagus cuniculus OX=9986 GN=S100A4 PE=3 SV=1	31.333333	1.48933163	0.19183289	0.4317619
G1U945$$Heterogeneous nuclear ribonucleoprotein D-like OS=Homo sapiens GN=HNRNPDL PE=1 SV=3	30.633333	1.489089915	0.19189416	0.4317619
G1SN21$$Uncharacterized protein OS=Oryctolagus cuniculus OX=9986 GN=KTN1 PE=4 SV=1	56.4	1.487566094	0.19228085	0.432105
G1SYM2$$Uncharacterized protein OS=Oryctolagus cuniculus OX=9986 GN=ILF2 PE=4 SV=2	19.6	1.481870405	0.19373278	0.4348382
G1SHF3$$Cytoskeleton associated protein 4 OS=Oryctolagus cuniculus OX=9986 GN=CKAP4 PE=4 SV=1	29.366667	1.480037055	0.19420234	0.4348429
G1SFU0$$Uncharacterized protein OS=Oryctolagus cuniculus OX=9986 GN=NCSTN PE=4 SV=1	-13.1	-1.477516524	0.19484965	0.4348429
G1T5X6$$Uncharacterized protein OS=Oryctolagus cuniculus OX=9986 GN=RAB5A PE=4 SV=1	32.933333	1.477381906	0.19488428	0.4348429
G1TS38$$Filamin-C OS=Homo sapiens GN=FLNC PE=1 SV=3	18.166667	1.477094176	0.19495832	0.4348429
G1TYH7$$Uncharacterized protein OS=Oryctolagus cuniculus OX=9986 GN=LXN PE=4 SV=1	25.266667	1.476644681	0.19507403	0.4348429
G1SWD0$$Uncharacterized protein OS=Oryctolagus cuniculus OX=9986 GN=PFAS PE=3 SV=1	-57.8	-1.476175946	0.19519477	0.4348429
G1TH33$$Uncharacterized protein OS=Oryctolagus cuniculus OX=9986 GN=GNB2 PE=4 SV=1	31.966667	1.475439157	0.19538469	0.4348429

G1SRH7$$Uncharacterized protein OS=Oryctolagus cuniculus OX=9986 PE=3 SV=1	15.766667	1.469281177	0.19697888	0.4372682
G1SWI3$$Uncharacterized protein OS=Oryctolagus cuniculus OX=9986 GN=SARS2 PE=4 SV=1	27.9	1.469010397	0.19704926	0.4372682
G1TGK9$$Sodium/potassium-transporting ATPase subunit beta OS=Oryctolagus cuniculus OX=9986 GN=ATP1B1 PE=3 SV=1	46.233333	1.468486736	0.19718544	0.4372682
G1SM05$$Uncharacterized protein OS=Oryctolagus cuniculus OX=9986 GN=CLCC1 PE=4 SV=1	47.466667	1.466340362	0.19774453	0.4379816
G1SWL6$$Uncharacterized protein OS=Oryctolagus cuniculus OX=9986 GN=LOC108176887 PE=4 SV=1	51.966667	1.462276944	0.19880707	0.439525
G1U6Q9$$""Capping protein (Actin filament) muscle Z-line, beta, isoform CRA_d OS=Homo sapiens GN=CAPZB PE=1 SV=1""	15.566667	1.461481488	0.19901571	0.439525
G1SJ23$$Uncharacterized protein OS=Oryctolagus cuniculus OX=9986 GN=TSC22D3 PE=4 SV=2	-30.86667	-1.460946835	0.19915605	0.439525
G1T6L7$$Non-specific serine/threonine protein kinase OS=Oryctolagus cuniculus OX=9986 GN=CDC42BPA PE=3 SV=1	30.8	1.454397833	0.20088273	0.442806
G1T1F0$$Serine/threonine-protein kinase Nek9 OS=Homo sapiens GN=NEK9 PE=1 SV=2	54.2	1.445817131	0.2031664	0.4473055
G1SUN1$$Uncharacterized protein OS=Oryctolagus cuniculus OX=9986 GN=NQO1 PE=4 SV=1	32	1.443544132	0.20377542	0.4481116
G1SQ87$$Syntaxin-binding protein 1 OS=Homo sapiens GN=STXBP1 PE=1 SV=1	75.8	1.440092942	0.20470338	0.4496164
G1U8R2$$Proteasome subunit alpha type OS=Homo sapiens GN=PSMA6 PE=1 SV=1	43.633333	1.438424975	0.20515329	0.4497359
U3KMN4$$""Succinate dehydrogenase [ubiquinone] flavoprotein subunit, mitochondrial OS=Homo sapiens GN=SDHA PE=1 SV=2""	20.6	1.437517587	0.20539843	0.4497359
G1SJY8$$""NAD kinase 2, mitochondrial OS=Homo sapiens GN=NADK2 PE=1 SV=2""	16.2	1.4371823	0.20548908	0.4497359
G1SX88$$Ubiquitin carboxyl-terminal hydrolase OS=Oryctolagus cuniculus OX=9986 GN=UCHL1 PE=3 SV=1	37	1.430827558	0.20721431	0.4525733
G1SGB5$$ATP synthase subunit gamma OS=Oryctolagus cuniculus OX=9986 GN=ATP5F1C PE=3 SV=1	17.533333	1.43060075	0.20727613	0.4525733

G1TRH3$$Uncharacterized protein OS=Oryctolagus cuniculus OX=9986 GN=SAR1B PE=3 SV=1	-62.23333	-1.429258479	0.20764238	0.4528371
G1TPN2$$Endoplasmic reticulum resident protein 29 OS=Oryctolagus cuniculus OX=9986 GN=ERP29 PE=4 SV=1	56.833333	1.426364921	0.20843395	0.4540267
G1T798$$Uncharacterized protein OS=Oryctolagus cuniculus OX=9986 GN=PTGR2 PE=4 SV=1	73.570301	1.422167033	0.20958736	0.4555505
G1TTY7$$Uncharacterized protein OS=Oryctolagus cuniculus OX=9986 GN=PLG PE=3 SV=1	30.166667	1.422022064	0.2096273	0.4555505
G1ST99$$Uncharacterized protein OS=Oryctolagus cuniculus OX=9986 GN=ABAT PE=3 SV=1	13.033333	1.420048696	0.21017165	0.4561961
G1SEM0$$Membrane primary amine oxidase OS=Homo sapiens GN=AOC3 PE=1 SV=3	-46.9	-1.418149841	0.2106967	0.4567984
G1SV12$$Uncharacterized protein OS=Oryctolagus cuniculus OX=9986 GN=COL15A1 PE=4 SV=1	50.966667	1.417080021	0.21099305	0.456904
G1SWC9$$Fibrinogen alpha chain (Fragment) OS=Oryctolagus cuniculus OX=9986 GN=FGA PE=1 SV=1	11.966667	1.415291705	0.2114893	0.4569901
G1SX80$$Intercellular adhesion molecule 1 OS=Oryctolagus cuniculus OX=9986 GN=ICAM1 PE=4 SV=1	16.833333	1.415151726	0.21152819	0.4569901
G1TNM3$$Uncharacterized protein OS=Oryctolagus cuniculus OX=9986 GN=PSMC3 PE=3 SV=2	11.7	1.410383167	0.212857	0.459323
G1T8H3$$Elongation factor 1-delta OS=Oryctolagus cuniculus OX=9986 GN=EEF1D PE=2 SV=1	51.9	1.406283973	0.2140055	0.4605859
G1U3X5$$Uncharacterized protein OS=Oryctolagus cuniculus OX=9986 GN=FDFT1 PE=4 SV=1	29.633333	1.406070398	0.21406549	0.4605859
G1SQM2$$Transmembrane protein 109 OS=Oryctolagus cuniculus OX=9986 GN=TMEM109 PE=4 SV=1	25.833333	1.405623225	0.21419116	0.4605859
G1TN54$$Uncharacterized protein OS=Oryctolagus cuniculus OX=9986 GN=TGFBR3 PE=4 SV=2	40	1.401420471	0.21537561	0.4625937
G1TVH4$$Uncharacterized protein OS=Oryctolagus cuniculus OX=9986 GN=SACM1L PE=4 SV=1	-67.83333	-1.395147995	0.21715469	0.4658726
Q01059$$Histone H2B OS=Oryctolagus cuniculus OX=9986 GN=HIST2H2BE PE=3 SV=1	18.2	1.392211148	0.21799235	0.4670272
G1TUB8$$Uncharacterized protein OS=Oryctolagus cuniculus OX=9986 GN=ITGA1 PE=3 SV=1	24.566667	1.391487716	0.21819915	0.4670272

G1SVW5$$Uncharacterized protein OS=Oryctolagus cuniculus OX=9986 GN=CHGA PE=4 SV=1	22.133333	1.38934977	0.21881137	0.4676258
G1SI54$$Actin-related protein 2 OS=Oryctolagus cuniculus OX=9986 GN=ACTR2 PE=3 SV=1	46.166667	1.388354268	0.21909698	0.4676258
G1SMN6$$Uncharacterized protein OS=Oryctolagus cuniculus OX=9986 GN=XPO1 PE=4 SV=2	-21.83333	-1.387859104	0.21923917	0.4676258
P11974$$""Succinate--CoA ligase [ADP-forming] subunit beta, mitochondrial OS=Oryctolagus cuniculus OX=9986 GN=SUCLA2 PE=3 SV=2""	14.933333	1.386041063	0.21976198	0.4681996
G1T548$$Charged multivesicular body protein 1b OS=Homo sapiens GN=CHMP1B PE=1 SV=1	-61.2	-1.375892009	0.22270177	0.4739155
G1SQL0$$Uncharacterized protein OS=Oryctolagus cuniculus OX=9986 GN=SHTN1 PE=4 SV=1	24.733333	1.373750763	0.22332662	0.4742068
G1SHI0$$Uncharacterized protein OS=Oryctolagus cuniculus OX=9986 GN=HUWE1 PE=4 SV=1	15.5	1.37244236	0.22370924	0.4742068
G1SSA2$$Uncharacterized protein OS=Oryctolagus cuniculus OX=9986 GN=DARS PE=3 SV=2	31.033333	1.372212545	0.22377651	0.4742068
G1SPZ9$$Non-specific lipid-transfer protein OS=Oryctolagus cuniculus OX=9986 GN=SCP2 PE=3 SV=2	28.233333	1.37159616	0.22395701	0.4742068
G1SCT9$$Plasminogen activator inhibitor 1 RNA-binding protein OS=Homo sapiens GN=SERBP1 PE=1 SV=2	-16	-1.370498307	0.22427885	0.4742068
G1T1V9$$Adapter molecule crk OS=Homo sapiens GN=CRK PE=1 SV=2	-42.83333	-1.369649009	0.22452812	0.4742068
G1SYV9$$Uncharacterized protein OS=Oryctolagus cuniculus OX=9986 GN=NAGLU PE=4 SV=1	-40.5	-1.368848217	0.22476338	0.4742068
G1TQ79$$Uncharacterized protein OS=Oryctolagus cuniculus OX=9986 GN=PDLIM5 PE=4 SV=2	51.133333	1.366918792	0.22533116	0.4742068
Q8HZQ5$$Glutathione peroxidase OS=Oryctolagus cuniculus OX=9986 GN=GPX1 PE=3 SV=1	49.166667	1.366237271	0.22553203	0.4742068
G1U0B5$$Uncharacterized protein OS=Oryctolagus cuniculus OX=9986 PE=4 SV=1	-17.66667	-1.365994954	0.22560349	0.4742068
G1SIJ6$$""Cytochrome c oxidase subunit 4 isoform 1, mitochondrial OS=Oryctolagus cuniculus OX=9986 GN=COX4I1 PE=4 SV=1""	-16.93333	-1.365783393	0.2256659	0.4742068
G1SQF2$$Proteasome subunit beta OS=Oryctolagus cuniculus OX=9986 GN=PSMB1 PE=3 SV=1	34.366667	1.360793248	0.22714252	0.4767667

G1TAE2$$Uncharacterized protein OS=Oryctolagus cuniculus OX=9986 GN=BIN1 PE=4 SV=1	24.266667	1.358643536	0.22778137	0.4775643
G1SUD1$$Uncharacterized protein OS=Oryctolagus cuniculus OX=9986 GN=RTRAF PE=4 SV=1	58.966667	1.35739069	0.22815445	0.4778036
G1U0B3$$Uncharacterized protein OS=Oryctolagus cuniculus OX=9986 PE=4 SV=1	27.666667	1.35385731	0.22920967	0.4794692
G1T643$$40S ribosomal protein S4 OS=Oryctolagus cuniculus OX=9986 GN=LOC100338112 PE=1 SV=1	34.366667	1.350434801	0.23023605	0.4810708
G1T7Z0$$Uncharacterized protein OS=Oryctolagus cuniculus OX=9986 GN=RAB4A PE=4 SV=1	12.333333	1.348664395	0.23076864	0.4816382
G1T2F2$$Protein S100 OS=Oryctolagus cuniculus OX=9986 GN=S100A11 PE=3 SV=1	10.566667	1.338123968	0.23396291	0.4877532
G1TBW7$$Uncharacterized protein OS=Oryctolagus cuniculus OX=9986 GN=ENOSF1 PE=4 SV=1	52.533333	1.331922435	0.23586113	0.4902795
G1SZ37$$Galectin-3 OS=Oryctolagus cuniculus OX=9986 GN=LGALS3 PE=2 SV=2	49.533333	1.331868387	0.23587774	0.4902795
G1TGI6$$Uncharacterized protein OS=Oryctolagus cuniculus OX=9986 GN=HSD17B10 PE=3 SV=1	31.033333	1.331561878	0.23597193	0.4902795
G1U248$$Uncharacterized protein OS=Oryctolagus cuniculus OX=9986 GN=RPL12 PE=1 SV=1	16.933333	1.328529309	0.23690568	0.4916659
G1TBL6$$Uncharacterized protein OS=Oryctolagus cuniculus OX=9986 GN=GBE1 PE=4 SV=2	13.733333	1.327003985	0.23737661	0.4920897
G1T8R1$$Uncharacterized protein OS=Oryctolagus cuniculus OX=9986 GN=PNPT1 PE=4 SV=1	17.066667	1.325576669	0.23781805	0.4924515
G1SIW8$$Sidoreflexin OS=Oryctolagus cuniculus OX=9986 GN=SFXN2 PE=3 SV=2	23.066667	1.319543689	0.23969222	0.4957759
G1SGX4$$Uncharacterized protein OS=Oryctolagus cuniculus OX=9986 GN=WDR1 PE=4 SV=1	20.633333	1.315618969	0.24091866	0.4976165
G1U7G6$$Unc-45 myosin chaperone A OS=Oryctolagus cuniculus OX=9986 GN=UNC45A PE=4 SV=1	40.166667	1.313820276	0.24148263	0.4976165
G1SFV1$$AP-1 complex subunit beta-1 OS=Homo sapiens GN=AP1B1 PE=1 SV=2	23.866667	1.313784384	0.2414939	0.4976165
G1T837$$Uncharacterized protein OS=Oryctolagus cuniculus OX=9986 GN=OGDH PE=4 SV=1	90.433768	1.313252467	0.24166092	0.4976165
G1T169$$Uncharacterized protein OS=Oryctolagus cuniculus OX=9986 GN=ITIH4 PE=4 SV=1	-18.5	-1.311343789	0.2422611	0.4982962

G1SN14$$Uncharacterized protein OS=Oryctolagus cuniculus OX=9986 GN=DAB2 PE=4 SV=2	29.7	1.308616564	0.24312101	0.4995081
G1SJM1$$Uncharacterized protein OS=Oryctolagus cuniculus OX=9986 GN=UFL1 PE=4 SV=1	-33.26667	-1.307486146	0.24347825	0.4996856
G1SG11$$THO complex subunit 4 OS=Homo sapiens GN=ALYREF PE=1 SV=1	19.366667	1.304853835	0.24431197	0.5003776
G1T652$$Aldehyde oxidase 1 OS=Oryctolagus cuniculus OX=9986 GN=AOX1 PE=4 SV=2	14.166667	1.304709269	0.24435784	0.5003776
G1SPP0$$Eukaryotic translation initiation factor 4H OS=Homo sapiens GN=EIF4H PE=1 SV=5	44	1.298691851	0.24627376	0.5034987
G1SSA8$$Uncharacterized protein OS=Oryctolagus cuniculus OX=9986 GN=DNM1L PE=3 SV=2	-13.73333	-1.298209759	0.24642784	0.5034987
G1SNM1$$60S ribosomal protein L18 (Fragment) OS=Homo sapiens GN=RPL18 PE=1 SV=1	39.8	1.297023975	0.24680721	0.503716
G1T7W7$$ATPase ASNA1 OS=Oryctolagus cuniculus OX=9986 GN=ASNA1 PE=3 SV=1	40.8	1.295885913	0.2471718	0.5039027
G1TK17$$Uncharacterized protein OS=Oryctolagus cuniculus OX=9986 GN=ACTC1 PE=3 SV=1	32.533333	1.293687136	0.24787758	0.5047838
G1SR28$$Uncharacterized protein OS=Oryctolagus cuniculus OX=9986 GN=TMX1 PE=4 SV=2	29.2	1.292834262	0.24815184	0.5047852
G1T9N2$$Proteasome subunit beta OS=Oryctolagus cuniculus OX=9986 GN=PSMB6 PE=3 SV=1	27.7	1.29172381	0.24850933	0.5049556
G1STT8$$Uncharacterized protein OS=Oryctolagus cuniculus OX=9986 GN=AIMP1 PE=4 SV=2	17.733333	1.287017017	0.25002977	0.5074862
G1SN11$$Mannosyl-oligosaccharide glucosidase OS=Homo sapiens GN=MOGS PE=1 SV=5	13.7	1.285993244	0.25036159	0.5076013
G1SJN5$$NADH dehydrogenase [ubiquinone] 1 alpha subcomplex subunit 12 OS=Homo sapiens GN=NDUFA12 PE=1 SV=1	11.266667	1.284746974	0.25076606	0.5078632
G1SN09$$ATP-dependent (S)-NAD(P)H-hydrate dehydratase OS=Oryctolagus cuniculus OX=9986 GN=CARKD PE=3 SV=1	36.366667	1.283002342	0.25133326	0.5084355
G1T594$$Uncharacterized protein OS=Oryctolagus cuniculus OX=9986 GN=UTRN PE=4 SV=2	51.633333	1.282183714	0.25159981	0.5084355
G1SLD6$$Uncharacterized protein OS=Oryctolagus cuniculus OX=9986 GN=SLK PE=4 SV=1	33.733333	1.280519106	0.25214258	0.5089749
G1SML9$$Tubulin-specific chaperone A OS=Oryctolagus cuniculus OX=9986 PE=3 SV=1	30.766667	1.278663812	0.25274878	0.5092066

G1T8C8$$Core histone macro-H2A OS=Oryctolagus cuniculus OX=9986 GN=H2AFY2 PE=4 SV=1	19.733333	1.278478658	0.25280935	0.5092066
G1T2V2$$Uncharacterized protein OS=Oryctolagus cuniculus OX=9986 PE=4 SV=1	38.166667	1.277065471	0.25327207	0.5095823
G1TSP4$$NADH:ubiquinone oxidoreductase subunit B4 OS=Oryctolagus cuniculus OX=9986 GN=NDUFB4 PE=4 SV=1	39.433333	1.274945559	0.25396762	0.5104016
G1TVH1$$Long-chain-fatty-acid--CoA ligase 1 OS=Homo sapiens GN=ACSL1 PE=1 SV=1	9.8333333	1.27410393	0.25424424	0.5104016
G1STE1$$Uncharacterized protein OS=Oryctolagus cuniculus OX=9986 GN=NDUFB11 PE=4 SV=1	20	1.273298578	0.25450919	0.5104016
G1SRB6$$Collagen alpha-2(I) chain OS=Oryctolagus cuniculus OX=9986 GN=COL1A2 PE=4 SV=1	21	1.271867653	0.25498056	0.5107917
G1TDI0$$Phosphoglycerate mutase 1 OS=Homo sapiens GN=PGAM1 PE=1 SV=2	25.166667	1.270044974	0.2555821	0.5114414
G1TE35$$""6-phosphogluconate dehydrogenase, decarboxylating OS=Oryctolagus cuniculus OX=9986 GN=PGD PE=3 SV=2""	19.533333	1.264562894	0.25739906	0.5145192
G1T6S4$$Uncharacterized protein OS=Oryctolagus cuniculus OX=9986 GN=IQGAP1 PE=4 SV=1	14.266667	1.263188823	0.25785629	0.5148754
G1SGF8$$Uncharacterized protein OS=Oryctolagus cuniculus OX=9986 GN=RAP1GDS1 PE=4 SV=2	-13.86667	-1.261955375	0.25826734	0.5151386
G1SZI5$$Uncharacterized protein OS=Oryctolagus cuniculus OX=9986 GN=HADHB PE=3 SV=1	35.933333	1.259407897	0.25911816	0.5160738
G1SDJ7$$Uncharacterized protein OS=Oryctolagus cuniculus OX=9986 GN=EPB41 PE=4 SV=2	9.9106121	1.258877476	0.25929562	0.5160738
G1T748$$Uncharacterized protein OS=Oryctolagus cuniculus OX=9986 GN=GLG1 PE=4 SV=2	14.7	1.252887881	0.26130716	0.5191502
G1TS23$$Uncharacterized protein OS=Oryctolagus cuniculus OX=9986 PE=3 SV=1	11	1.252600352	0.26140408	0.5191502
G1SQZ4$$Uncharacterized protein OS=Oryctolagus cuniculus OX=9986 GN=NDUFS2 PE=3 SV=2	41.533333	1.245428552	0.26383177	0.5234082
G1U6N8$$Uncharacterized protein OS=Oryctolagus cuniculus OX=9986 GN=ARSA PE=4 SV=2	10.366667	1.239375848	0.26589624	0.5269372
G1T013$$Beta-enolase OS=Oryctolagus cuniculus OX=9986 GN=ENO3 PE=1 SV=4	57.266667	1.229861273	0.26917053	0.5328537
G1TAA4$$Uncharacterized protein OS=Oryctolagus cuniculus OX=9986 GN=GARS PE=4 SV=2	49.8	1.228390136	0.26967997	0.5330338

G1TX67$$ATP synthase subunit alpha OS=Oryctolagus cuniculus OX=9986 GN=ATP5F1A PE=3 SV=1	-61.23333	-1.2279304	0.26983935	0.5330338
G1TC10$$Uncharacterized protein OS=Oryctolagus cuniculus OX=9986 GN=CHMP4B PE=3 SV=1	23.166667	1.224263439	0.27111359	0.5349782
G1TUA3$$Uncharacterized protein OS=Oryctolagus cuniculus OX=9986 GN=FOLH1 PE=4 SV=1	10.6	1.220301823	0.2724962	0.5371319
G1TV43$$Rab GDP dissociation inhibitor OS=Oryctolagus cuniculus OX=9986 GN=GDI1 PE=3 SV=1	43.3	1.218116088	0.27326168	0.537216
G1SZW8$$Uncharacterized protein OS=Oryctolagus cuniculus OX=9986 GN=ACTBL2 PE=3 SV=1	-12.8	-1.217329822	0.27353751	0.537216
G1SSE1$$Uncharacterized protein OS=Oryctolagus cuniculus OX=9986 GN=LOC100347143 PE=3 SV=2	50.966667	1.217082351	0.27362438	0.537216
G1TL06$$Uncharacterized protein OS=Oryctolagus cuniculus OX=9986 GN=AARS PE=3 SV=1	16.1	1.21646857	0.27383993	0.537216
G1TAS1$$""2-amino-3-ketobutyrate coenzyme A ligase, mitochondrial OS=Homo sapiens GN=GCAT PE=1 SV=1""	23.6	1.216028081	0.27399472	0.537216
G1SXI9$$ATP synthase protein 8 OS=Oryctolagus cuniculus OX=9986 GN=MT-ATP8 PE=3 SV=1	24.1	1.215148434	0.27430405	0.5372516
G1SMI7$$Uncharacterized protein OS=Oryctolagus cuniculus OX=9986 GN=AAK1 PE=4 SV=2	17.418547	1.21028719	0.27601911	0.5398287
G1SE76$$GTP-binding nuclear protein Ran (Fragment) OS=Homo sapiens GN=RAN PE=1 SV=1	28.266667	1.209761773	0.27620505	0.5398287
G1TMM7$$Uncharacterized protein OS=Oryctolagus cuniculus OX=9986 GN=HIST1H1D PE=3 SV=1	27.333333	1.206310585	0.27742909	0.5405202
G1SIP2$$Enhancer of rudimentary homolog OS=Oryctolagus cuniculus OX=9986 PE=3 SV=1	14.366667	1.205575893	0.27769028	0.5405202
G1SS49$$Complement component C9 OS=Oryctolagus cuniculus OX=9986 GN=C9 PE=4 SV=1	10.5	1.205090546	0.27786294	0.5405202
G1TVT6$$Protein flightless-1 homolog OS=Homo sapiens GN=FLII PE=1 SV=2	39.433333	1.204693861	0.27800413	0.5405202
G1SDA8$$Uncharacterized protein OS=Oryctolagus cuniculus OX=9986 GN=RAB6A PE=4 SV=1	12.166667	1.203263129	0.2785139	0.5405202
P01948$$Uncharacterized protein OS=Oryctolagus cuniculus OX=9986 GN=DBNL PE=4 SV=1	-26.83333	-1.203177754	0.27854435	0.5405202

G1T2U6$$Uncharacterized protein OS=Oryctolagus cuniculus OX=9986 GN=GOLGA5 PE=4 SV=1	66.255198	1.202994772	0.27860961	0.5405202
G1SKZ8$$Uncharacterized protein OS=Oryctolagus cuniculus OX=9986 PE=4 SV=1	11.9	1.194342403	0.28171089	0.5456882
G1TYW1$$Uncharacterized protein OS=Oryctolagus cuniculus OX=9986 GN=ARHGEF11 PE=4 SV=2	10.966667	1.193800646	0.28190607	0.5456882
G1TWL0$$Uncharacterized protein OS=Oryctolagus cuniculus OX=9986 GN=PSMD5 PE=4 SV=1	12.433333	1.192284697	0.28245287	0.5456882
P01840$$Uncharacterized protein OS=Oryctolagus cuniculus OX=9986 GN=COASY PE=3 SV=1	-23.33333	-1.192274662	0.28245649	0.5456882
G1TBU9$$Alpha-actinin-4 OS=Homo sapiens GN=ACTN4 PE=1 SV=2	29.566667	1.19042837	0.28312371	0.5464051
G1TNY1$$Uncharacterized protein OS=Oryctolagus cuniculus OX=9986 GN=PARK7 PE=4 SV=1	49.4	1.186366774	0.28459635	0.5483331
U3KNQ3$$14-3-3 protein zeta/delta (Fragment) OS=Homo sapiens GN=YWHAZ PE=1 SV=1	103.56667	1.185990948	0.28473295	0.5483331
G1TPW2$$Proteasome subunit alpha type-1 OS=Homo sapiens GN=PSMA1 PE=1 SV=1	22.133333	1.185217349	0.28501432	0.5483331
G1THH7$$Uncharacterized protein OS=Oryctolagus cuniculus OX=9986 PE=4 SV=1	-23.56667	-1.180450804	0.28675332	0.551104
G1T7I4$$Uncharacterized protein OS=Oryctolagus cuniculus OX=9986 GN=RAB18 PE=4 SV=1	24.1	1.174403445	0.28897291	0.5547845
Q29502$$Prothrombin OS=Oryctolagus cuniculus OX=9986 GN=F2 PE=3 SV=1	-10.46667	-1.173597713	0.28926977	0.5547845
G1SE36$$Uncharacterized protein OS=Oryctolagus cuniculus OX=9986 GN=HSPA9 PE=3 SV=1	33	1.16460112	0.29260247	0.5605935
P41982$$Uncharacterized protein OS=Oryctolagus cuniculus OX=9986 GN=MAPRE1 PE=4 SV=1	26.2	1.162506772	0.29338306	0.5612285
G1TZ63$$Uncharacterized protein OS=Oryctolagus cuniculus OX=9986 PE=3 SV=2	36.933333	1.161018039	0.29393903	0.5612285
G1SES8$$Elongation factor Tu OS=Oryctolagus cuniculus OX=9986 GN=TUFM PE=3 SV=1	-14.03333	-1.160287229	0.29421229	0.5612285
G1SKV7$$Uncharacterized protein OS=Oryctolagus cuniculus OX=9986 PE=4 SV=2	-9.1	-1.159691884	0.29443505	0.5612285
G1SR79$$Uncharacterized protein OS=Oryctolagus cuniculus OX=9986 GN=SNRPA1 PE=4 SV=1	30.4	1.159638992	0.29445485	0.5612285
G1SDA4$$Uncharacterized protein OS=Oryctolagus cuniculus OX=9986 GN=LAMB1 PE=4 SV=2	31.533333	1.158086231	0.29503659	0.561757

G1SKK0$$Alpha-1-microglobulin/bikunin precursor OS=Oryctolagus cuniculus OX=9986 GN=AMBP PE=4 SV=1	39.5	1.154753451	0.29628856	0.5633935
G1T6Q8$$Trifunctional purine biosynthetic protein adenosine-3 OS=Oryctolagus cuniculus OX=9986 GN=GART PE=3 SV=2	-9.466667	-1.154173741	0.2965068	0.5633935
G1TP30$$Uncharacterized protein (Fragment) OS=Homo sapiens PE=1 SV=1	36.866667	1.151550532	0.29749608	0.5645987
G1STP6$$Uncharacterized protein OS=Oryctolagus cuniculus OX=9986 PE=4 SV=1	23.866667	1.150870098	0.29775315	0.5645987
O77622$$Uncharacterized protein OS=Oryctolagus cuniculus OX=9986 GN=STRAP PE=4 SV=2	14.7	1.146380936	0.299454	0.5672409
G1TTL1$$Uncharacterized protein OS=Oryctolagus cuniculus OX=9986 GN=NUDT21 PE=4 SV=1	53.595588	1.141004021	0.30150222	0.5701819
G1TZE2$$Uncharacterized protein OS=Oryctolagus cuniculus OX=9986 GN=CTSC PE=3 SV=1	24.733333	1.14068351	0.30162469	0.5701819
U3KMU6$$Uncharacterized protein OS=Oryctolagus cuniculus OX=9986 GN=COL14A1 PE=4 SV=1	27.433333	1.138996103	0.30227017	0.5707074
G1TDF3$$Ran-specific GTPase-activating protein OS=Homo sapiens GN=RANBP1 PE=1 SV=1	60.595139	1.138340425	0.30252131	0.5707074
G1TXF6$$Uncharacterized protein OS=Oryctolagus cuniculus OX=9986 PE=3 SV=1	28.366667	1.136060373	0.30339601	0.5716765
G1SLK2$$Hemopexin OS=Oryctolagus cuniculus OX=9986 GN=HPX PE=3 SV=1	17.9	1.135387111	0.30365471	0.5716765
G1THY3$$Plectin OS=Homo sapiens GN=PLEC PE=1 SV=3	51.1	1.130569619	0.30551135	0.5745856
G1THH9$$Uncharacterized protein OS=Oryctolagus cuniculus OX=9986 GN=DHCR24 PE=4 SV=1	17.166667	1.12955648	0.30590305	0.5747364
G1TM88$$AP-2 complex subunit alpha-2 OS=Homo sapiens GN=AP2A2 PE=1 SV=2	22.766667	1.127916893	0.30653785	0.5751424
G1TJS2$$Tropomyosin beta chain OS=Homo sapiens GN=TPM2 PE=1 SV=1	-8.933333	-1.127004687	0.30689152	0.5751424
G1TJP8$$Uncharacterized protein OS=Oryctolagus cuniculus OX=9986 GN=RPL14 PE=1 SV=1	42.6	1.126585059	0.30705433	0.5751424
P41035$$Uncharacterized protein OS=Oryctolagus cuniculus OX=9986 GN=AP3M1 PE=3 SV=1	28.866667	1.124817917	0.30774077	0.5758435
G1SNP9$$Thioredoxin-related transmembrane protein 4 OS=Homo sapiens GN=TMX4 PE=1 SV=1	40.166667	1.118609671	0.31016274	0.579159

G1SPR9$$Alpha-synuclein OS=Oryctolagus cuniculus OX=9986 GN=SNCA PE=3 SV=1	39.433333	1.118476879	0.31021472	0.579159
G1U4Z7$$Uncharacterized protein OS=Oryctolagus cuniculus OX=9986 GN=GALK2 PE=4 SV=1	32.233333	1.117461032	0.31061263	0.579159
G1SI83$$Uncharacterized protein OS=Oryctolagus cuniculus OX=9986 GN=CYP17A1 PE=3 SV=1	-18.4	-1.11706411	0.31076822	0.579159
G1SRK1$$Uncharacterized protein OS=Oryctolagus cuniculus OX=9986 GN=PSMD8 PE=4 SV=1	35.666667	1.11283632	0.31242962	0.5816677
G1SKN2$$Uncharacterized protein OS=Oryctolagus cuniculus OX=9986 GN=TIMM8A PE=3 SV=1	25.4	1.107734872	0.31444441	0.5842205
G1SVC5$$Proteasome subunit beta OS=Oryctolagus cuniculus OX=9986 GN=PSMB3 PE=3 SV=1	42.4	1.105898108	0.31517253	0.5842205
G1SMS2$$Uncharacterized protein OS=Oryctolagus cuniculus OX=9986 GN=RPN2 PE=4 SV=2	-33.6	-1.105675691	0.31526079	0.5842205
G1SD01$$Uncharacterized protein OS=Oryctolagus cuniculus OX=9986 PE=4 SV=2	-20.63333	-1.105223382	0.31544036	0.5842205
G1SLX6$$Uncharacterized protein OS=Oryctolagus cuniculus OX=9986 GN=DYNC1LI2 PE=4 SV=1	44.766667	1.105102902	0.3154882	0.5842205
G1SD95$$Uncharacterized protein OS=Oryctolagus cuniculus OX=9986 PE=4 SV=1	12.5	1.104091008	0.31589028	0.5842205
G1TKC4$$Uncharacterized protein OS=Oryctolagus cuniculus OX=9986 GN=MACF1 PE=4 SV=2	31.733333	1.102251522	0.31662233	0.5842205
G1TZI2$$Alpha-mannosidase 2C1 OS=Homo sapiens GN=MAN2C1 PE=1 SV=1	30.7	1.102209581	0.31663903	0.5842205
G1SQ11$$Uncharacterized protein OS=Oryctolagus cuniculus OX=9986 GN=HIST1H1A PE=3 SV=1	32.5	1.102180312	0.31665069	0.5842205
G1T9U5$$Uncharacterized protein OS=Oryctolagus cuniculus OX=9986 GN=TSG101 PE=4 SV=1	25.766667	1.098851264	0.31797928	0.5860857
G1SVM1$$Tyrosine--tRNA ligase OS=Oryctolagus cuniculus OX=9986 GN=YARS PE=3 SV=1	19.933333	1.091959844	0.32074457	0.5905925
G1T2Z8$$Carnitine O-acetyltransferase OS=Homo sapiens GN=CRAT PE=1 SV=5	36.5	1.089356059	0.32179465	0.5916405
G1TBQ6$$Uncharacterized protein OS=Oryctolagus cuniculus OX=9986 GN=PSMD9 PE=4 SV=1	31.266667	1.08895895	0.32195506	0.5916405
G1T545$$Cytochrome P450 family 2 subfamily F member 1 OS=Oryctolagus cuniculus OX=9986 GN=CYP2F1 PE=3 SV=1	12.233333	1.085387596	0.32340068	0.5937057

G1SZ59$$Polyadenylate-binding protein OS=Oryctolagus cuniculus OX=9986 GN=PABPC4 PE=3 SV=2	21.633333	1.083123092	0.32432014	0.5948018
G1TS93$$HCG2044781 OS=Homo sapiens GN=TMEM189-UBE2V1 PE=3 SV=1	26.966667	1.081469072	0.32499311	0.5954442
G1T040$$Uncharacterized protein OS=Oryctolagus cuniculus OX=9986 GN=RAB10 PE=4 SV=1	20.266667	1.076902707	0.32685713	0.5982653
G1TFZ6$$Alpha-mannosidase OS=Oryctolagus cuniculus OX=9986 GN=MAN2A1 PE=3 SV=2	14.033333	1.072711551	0.32857588	0.6004877
G1U0H7$$Caveolae-associated protein 1 OS=Homo sapiens GN=CAVIN1 PE=1 SV=1	41.066667	1.07214923	0.32880706	0.6004877
G1TE69$$Alpha-1B-glycoprotein OS=Oryctolagus cuniculus OX=9986 GN=A1BG PE=4 SV=1	22.5	1.07156415	0.32904773	0.6004877
G1SDG2$$Spermidine synthase OS=Homo sapiens GN=SRM PE=1 SV=1	29.233333	1.070199444	0.32960969	0.6009189
G1SW32$$Ankyrin repeat and MYND domain-containing protein 2 OS=Homo sapiens GN=ANKMY2 PE=1 SV=1	21.2	1.068564075	0.33028417	0.6015541
G1SZ64$$Uncharacterized protein OS=Oryctolagus cuniculus OX=9986 GN=ADIPOQ PE=4 SV=1	-30.66667	-1.067038431	0.33091442	0.6021076
G1SIF7$$Aldose reductase OS=Oryctolagus cuniculus OX=9986 GN=AKR1B1 PE=4 SV=1	10.166667	1.065711234	0.33146352	0.6025125
G1SGS7$$Calcium-transporting ATPase OS=Oryctolagus cuniculus OX=9986 GN=ATP2A2 PE=3 SV=1	33.133333	1.062997138	0.33258878	0.6029379
G1T3M3$$Glucosamine-6-phosphate isomerase OS=Oryctolagus cuniculus OX=9986 GN=GNPDA1 PE=3 SV=1	49.066667	1.062463773	0.33281029	0.6029379
G1SRE6$$Serine/threonine-protein phosphatase 2A catalytic subunit beta isoform OS=Oryctolagus cuniculus OX=9986 GN=PPP2CB PE=1 SV=1	-21.9	-1.062028512	0.33299114	0.6029379
G1U3V0$$Uncharacterized protein OS=Oryctolagus cuniculus OX=9986 GN=PFDN1 PE=4 SV=1	80.1	1.06199581	0.33300473	0.6029379
G1SCE1$$Uncharacterized protein OS=Oryctolagus cuniculus OX=9986 GN=SNRPB2 PE=4 SV=2	25.266667	1.060778837	0.33351085	0.603078
G1TAN8$$MICOS complex subunit OS=Oryctolagus cuniculus OX=9986 GN=CHCHD6 PE=3 SV=2	-23.6	-1.060238287	0.33373586	0.603078
G1TK53$$Uncharacterized protein OS=Oryctolagus cuniculus OX=9986 GN=SUMF2 PE=4 SV=2	23.733333	1.055399977	0.33575551	0.606134

G1SLC0$$Uncharacterized protein OS=Oryctolagus cuniculus OX=9986 GN=MRPS26 PE=4 SV=1	48.533333	1.053321292	0.33662633	0.6065194
G1TN62$$Uncharacterized protein OS=Oryctolagus cuniculus OX=9986 GN=ABHD14B PE=4 SV=1	25.833333	1.053320912	0.33662649	0.6065194
G1SI71$$Aldehyde oxidase 1 OS=Oryctolagus cuniculus OX=9986 GN=AOX1 PE=1 SV=2	40.833333	1.050498961	0.33781169	0.6072215
G1SXX2$$Uncharacterized protein OS=Oryctolagus cuniculus OX=9986 GN=UBE2V2 PE=4 SV=1	21.133333	1.050440087	0.33783645	0.6072215
G1SV05$$Tropomyosin alpha-1 chain (Fragment) OS=Homo sapiens GN=TPM1 PE=1 SV=1	15.366667	1.050042998	0.33800352	0.6072215
U3KN73$$Chaperonin-containing T-complex polypeptide beta subunit OS=Oryctolagus cuniculus OX=9986 GN=CCT2 PE=2 SV=1	11.833333	1.049197998	0.33835925	0.6072693
G1TX59$$Spectrin beta chain OS=Oryctolagus cuniculus OX=9986 GN=SPTBN1 PE=3 SV=2	51.833333	1.048051238	0.33884252	0.6073649
G1TFB5$$Echinoderm microtubule associated protein like 2 OS=Oryctolagus cuniculus OX=9986 GN=EML2 PE=4 SV=2	32.633333	1.046909811	0.33932411	0.6073649
G1SFS8$$Tubulin folding cofactor D OS=Oryctolagus cuniculus OX=9986 GN=TBCD PE=4 SV=2	14.633333	1.046270649	0.33959404	0.6073649
G1TTK6$$Calmodulin-2 OS=Homo sapiens GN=CALM2 PE=1 SV=1	38.333333	1.045950463	0.33972932	0.6073649
U3KM89$$Uncharacterized protein OS=Oryctolagus cuniculus OX=9986 GN=PPM1K PE=3 SV=1	23.7	1.044179341	0.34047846	0.608115
G1SYD6$$Uncharacterized protein OS=Oryctolagus cuniculus OX=9986 GN=NDUFS3 PE=3 SV=1	-50.46667	-1.034712682	0.3445058	0.6142758
G1SY94$$Histone H2A OS=Oryctolagus cuniculus OX=9986 GN=H2AFV PE=3 SV=1	33.866667	1.03346779	0.34503833	0.6142758
P09809$$Uncharacterized protein OS=Oryctolagus cuniculus OX=9986 GN=SF3A1 PE=4 SV=1	-56.06667	-1.033114612	0.34518953	0.6142758
G1SXQ0$$Prostaglandin reductase 1 OS=Oryctolagus cuniculus OX=9986 GN=PTGR1 PE=2 SV=1	19.666667	1.032950883	0.34525964	0.6142758
G1T359$$UTP--glucose-1-phosphate uridylyltransferase OS=Oryctolagus cuniculus OX=9986 GN=UGP2 PE=3 SV=1	16.5	1.027799956	0.34747141	0.6161977
G1U971$$Ubiquitin-like modifier-activating enzyme 1 OS=Oryctolagus cuniculus OX=9986 GN=UBA1 PE=3 SV=1	25.666667	1.027637486	0.34754136	0.6161977
G1T5J6$$Hypoxia up-regulated 1 OS=Oryctolagus cuniculus OX=9986 GN=HYOU1 PE=3 SV=1	29.833333	1.02759272	0.34756064	0.6161977

G1SZ25$$Uncharacterized protein OS=Oryctolagus cuniculus OX=9986 GN=ACOT13 PE=4 SV=1	57.88689	1.027310552	0.34768216	0.6161977
G1U723$$Uncharacterized protein OS=Oryctolagus cuniculus OX=9986 GN=TSC22D4 PE=4 SV=1	36.033333	1.026550311	0.34800975	0.6161977
G1T2G4$$Splicing factor 3B subunit 1 OS=Homo sapiens GN=SF3B1 PE=1 SV=3	18.766667	1.025062701	0.3486515	0.6167421
G1SMI6$$Fascin OS=Homo sapiens GN=FSCN1 PE=1 SV=3	15.1	1.023046832	0.34952269	0.617691
G1SYQ2$$Uncharacterized protein OS=Oryctolagus cuniculus OX=9986 GN=DNAJA2 PE=3 SV=1	54.184723	1.019708516	0.35096932	0.619654
U3KPE7$$Uncharacterized protein OS=Oryctolagus cuniculus OX=9986 GN=NAP1L4 PE=3 SV=1	28.733333	1.017436858	0.35195653	0.6208029
G1SIT9$$Uncharacterized protein OS=Oryctolagus cuniculus OX=9986 GN=PRPH PE=3 SV=2	17.7	1.01562848	0.35274403	0.6215976
G1T9Q3$$Serine/threonine-protein phosphatase 2A 65 kDa regulatory subunit A alpha isoform OS=Homo sapiens GN=PPP2R1A PE=1 SV=4	16.2	1.0139939	0.35345709	0.6219574
G1TDF6$$60S ribosomal protein L36 OS=Oryctolagus cuniculus OX=9986 GN=LOC100348796 PE=1 SV=1	37.4	1.013401846	0.35371565	0.6219574
G1SZ44$$Cytoplasmic dynein 1 heavy chain 1 OS=Homo sapiens GN=DYNC1H1 PE=1 SV=5	32	1.012843828	0.35395949	0.6219574
G1TQZ7$$Decorin OS=Oryctolagus cuniculus OX=9986 GN=DCN PE=2 SV=1	-42	-1.009877116	0.35525818	0.6223761
G1SGA5$$Glia maturation factor beta OS=Homo sapiens GN=GMFB PE=1 SV=2	20.3	1.008235316	0.35597855	0.6223761
G1SPQ9$$Uncharacterized protein OS=Oryctolagus cuniculus OX=9986 GN=LMO7 PE=4 SV=2	-16.66667	-1.007417205	0.35633796	0.6223761
G1TAY6$$Uncharacterized protein OS=Oryctolagus cuniculus OX=9986 GN=THYN1 PE=4 SV=1	-55.8	-1.007278133	0.35639909	0.6223761
G1TZ19$$Uncharacterized protein OS=Oryctolagus cuniculus OX=9986 GN=RA_M006_JSM7BED4F PE=4 SV=1	44	1.006518857	0.35673296	0.6223761
P27170$$Guanine nucleotide-binding protein G(q) subunit alpha OS=Homo sapiens GN=GNAQ PE=1 SV=4	-76.86667	-1.006181095	0.35688156	0.6223761
G1TBC6$$Uncharacterized protein OS=Oryctolagus cuniculus OX=9986 GN=COPS3 PE=4 SV=1	43.066667	1.006152189	0.35689428	0.6223761

G1T5F8$$Uncharacterized protein OS=Homo sapiens PE=1 SV=1	35.066667	1.006147328	0.35689642	0.6223761
G1SI20$$Serine/threonine-protein phosphatase OS=Oryctolagus cuniculus OX=9986 GN=PPP3CB PE=3 SV=1	37.833333	1.00284573	0.35835172	0.6243238
G1TKE0$$Uncharacterized protein OS=Oryctolagus cuniculus OX=9986 GN=NHLRC2 PE=4 SV=1	35.566667	1.001089749	0.35912769	0.6250439
G1T9S4$$Poly(rC)-binding protein 2 OS=Homo sapiens GN=PCBP2 PE=1 SV=1	29.933333	1.000377976	0.35944262	0.6250439
G1TWY9$$Creatine kinase M-type OS=Oryctolagus cuniculus OX=9986 GN=CKM PE=1 SV=1	-10.2	-0.99565337	0.3615387	0.6280969
G1SNP8$$Mitogen-activated protein kinase 14 OS=Homo sapiens GN=MAPK14 PE=1 SV=3	37.7	0.993106184	0.36267288	0.6294746
G1SM15$$Platelet-derived growth factor receptor beta OS=Oryctolagus cuniculus OX=9986 GN=PDGFRB PE=3 SV=2	20.633333	0.989111634	0.36445731	0.6319772
G1T1X2$$TRIM39-RPP21 readthrough OS=Oryctolagus cuniculus OX=9986 GN=TRIM39-RPP21 PE=3 SV=2	-24.86667	-0.988111764	0.36490508	0.6321595
G1SY68$$Catechol-O-methyltransferase OS=Oryctolagus cuniculus OX=9986 GN=COMT PE=4 SV=2	23.9	0.986943895	0.36542864	0.6324727
G1T7U7$$Proteasome subunit beta type-7 OS=Homo sapiens GN=PSMB7 PE=1 SV=1	26.566667	0.985762477	0.3659589	0.6327968
G1SQS1$$Uncharacterized protein OS=Oryctolagus cuniculus OX=9986 GN=PRKAR1A PE=4 SV=2	38	0.981755075	0.36776216	0.6353195
G1SUX8$$Uncharacterized protein OS=Oryctolagus cuniculus OX=9986 GN=PPA2 PE=4 SV=2	-31.83333	-0.980705882	0.36823546	0.635542
G1SE28$$Uncharacterized protein OS=Oryctolagus cuniculus OX=9986 GN=HNRNPU PE=4 SV=2	23.666667	0.979169843	0.36892927	0.635878
G1SFV7$$Uncharacterized protein OS=Oryctolagus cuniculus OX=9986 GN=RPS27A PE=1 SV=1	21.633333	0.978749255	0.36911943	0.635878
G1SNH7$$Uncharacterized protein OS=Oryctolagus cuniculus OX=9986 GN=ITIH2 PE=4 SV=1	38.833333	0.975338779	0.37066428	0.6376364
G1TR42$$Uncharacterized protein OS=Oryctolagus cuniculus OX=9986 GN=GGCT PE=4 SV=1	46.533333	0.974970669	0.37083134	0.6376364
G1SD44$$Uncharacterized protein OS=Oryctolagus cuniculus OX=9986 GN=TMEM205 PE=4 SV=1	38.366667	0.969828645	0.37317119	0.6406303

G1SRA8$$A-kinase anchoring protein 12 OS=Oryctolagus cuniculus OX=9986 GN=AKAP12 PE=4 SV=1	29.533333	0.969618673	0.37326699	0.6406303
G1SNE5$$Small nuclear ribonucleoprotein-associated protein OS=Oryctolagus cuniculus OX=9986 GN=SNRPN PE=3 SV=1	55.745082	0.968590645	0.3737363	0.6407684
G1TJG6$$Uncharacterized protein OS=Oryctolagus cuniculus OX=9986 GN=CANX PE=3 SV=2	22.033333	0.967921496	0.37404203	0.6407684
G1TS97$$Eukaryotic translation initiation factor 3 subunit F OS=Oryctolagus cuniculus OX=9986 GN=EIF3F PE=1 SV=1	45.066667	0.966865325	0.37452499	0.6410006
G1TLW3$$Microtubule-associated protein OS=Oryctolagus cuniculus OX=9986 GN=MAP4 PE=4 SV=2	31.5	0.961698813	0.37689472	0.6444585
G1SXL9$$Filamin-B OS=Oryctolagus cuniculus OX=9986 GN=FLNB PE=4 SV=1	34.666667	0.957609702	0.37877875	0.6470804
Q8WN94$$Uncharacterized protein OS=Oryctolagus cuniculus OX=9986 GN=ABCB6 PE=4 SV=2	30.6	0.954091425	0.38040576	0.6492587
G1TGV6$$Uncharacterized protein OS=Oryctolagus cuniculus OX=9986 GN=PRKAG1 PE=4 SV=1	-8.733333	-0.953106488	0.38086223	0.649437
G1T295$$ER membrane protein complex subunit 4 OS=Oryctolagus cuniculus OX=9986 GN=EMC4 PE=3 SV=1	-8.5	-0.951011664	0.38183453	0.6500188
G1T443$$Uncharacterized protein OS=Oryctolagus cuniculus OX=9986 GN=GALK1 PE=4 SV=1	39.833333	0.950853398	0.38190807	0.6500188
G1SLT2$$Uncharacterized protein OS=Oryctolagus cuniculus OX=9986 GN=APOH PE=4 SV=2	46.066667	0.946742	0.38382237	0.6526749
U3KMZ9$$Uncharacterized protein OS=Oryctolagus cuniculus OX=9986 GN=CUBN PE=4 SV=1	16.033333	0.942520166	0.38579598	0.6553791
G1SK00$$Serpin family B member 9 OS=Oryctolagus cuniculus OX=9986 GN=SERPINB9 PE=3 SV=2	12	0.941723254	0.38616942	0.6553791
G1SWM7$$Calponin OS=Oryctolagus cuniculus OX=9986 GN=CNN3 PE=3 SV=1	29.166667	0.941064508	0.38647832	0.6553791
G1TEI2$$Peroxiredoxin-2 OS=Homo sapiens GN=PRDX2 PE=1 SV=5	33.566667	0.938992274	0.38745133	0.6558655
G1TAM3$$Uncharacterized protein OS=Oryctolagus cuniculus OX=9986 GN=DPYSL3 PE=4 SV=1	56.366667	0.938434732	0.38771345	0.6558655
G1TZ26$$Uncharacterized protein OS=Oryctolagus cuniculus OX=9986 GN=GNB4 PE=4 SV=2	47.2	0.938183609	0.38783156	0.6558655

G1TXK3$$Proteasome subunit alpha type-4 OS=Homo sapiens GN=PSMA4 PE=1 SV=1	27.366667	0.936940008	0.38841687	0.6562538
B7NZJ1$$Uncharacterized protein OS=Oryctolagus cuniculus OX=9986 GN=PLAA PE=4 SV=2	-9.466667	-0.934718251	0.38946428	0.6574214
G1T3Y8$$Proteasome subunit beta OS=Oryctolagus cuniculus OX=9986 GN=PSMB4 PE=3 SV=1	22.766667	0.933657162	0.3899653	0.6576654
G1TPE4$$Ras-related protein Rap-1A OS=Homo sapiens GN=RAP1A PE=1 SV=1	51.910374	0.931822502	0.39083277	0.6584296
G1TLG5$$""Heat shock protein 75 kDa, mitochondrial OS=Homo sapiens GN=TRAP1 PE=1 SV=3""	25.433333	0.931190019	0.39113217	0.6584296
G1SQG1$$Hemoglobin subunit beta-1/2 OS=Oryctolagus cuniculus OX=9986 GN=HBB1 PE=1 SV=2	47.018464	0.927125447	0.39306055	0.6610727
G1SIL8$$Ferritin OS=Oryctolagus cuniculus OX=9986 PE=3 SV=1	36.866667	0.924832532	0.39415168	0.6615222
G1SXF7$$Uncharacterized protein OS=Oryctolagus cuniculus OX=9986 GN=TFG PE=4 SV=1	10.166667	0.924417458	0.39434946	0.6615222
G1TRM4$$Uncharacterized protein OS=Oryctolagus cuniculus OX=9986 GN=ITIH1 PE=4 SV=2	9.6666667	0.924085903	0.39450749	0.6615222
G1T0W0$$Uncharacterized protein OS=Oryctolagus cuniculus OX=9986 PE=4 SV=1	46.263807	0.923039198	0.39500672	0.6615222
G1TB64$$Uncharacterized protein OS=Oryctolagus cuniculus OX=9986 GN=COMMD9 PE=4 SV=1	37.1	0.92280059	0.3951206	0.6615222
G1T650$$Sorting nexin-3 OS=Homo sapiens GN=SNX3 PE=1 SV=3	53.163783	0.921118538	0.39592408	0.6622665
P53787$$Uncharacterized protein OS=Oryctolagus cuniculus OX=9986 GN=SLC25A20 PE=3 SV=2	21.3	0.913328877	0.39966169	0.6674528
G1T8D4$$tRNA-splicing ligase RtcB homolog OS=Homo sapiens GN=RTCB PE=1 SV=1	12.233333	0.913149412	0.39974812	0.6674528
G1TEZ4$$Uncharacterized protein OS=Oryctolagus cuniculus OX=9986 GN=IARS PE=3 SV=2	30.866667	0.911123848	0.40072468	0.6684783
G1SSG2$$Uncharacterized protein OS=Oryctolagus cuniculus OX=9986 PE=4 SV=1	46.5	0.908400346	0.40204063	0.6698973
A0A0A0MQQ6$$Far upstream element-binding protein 1 OS=Homo sapiens GN=FUBP1 PE=1 SV=1	33.166667	0.907177105	0.40263277	0.6698973
G1SVF2$$Anion exchange protein OS=Oryctolagus cuniculus OX=9986 GN=SLC4A1 PE=3 SV=2	26.8	0.905885469	0.40325876	0.6698973

G1SV03$$Uncharacterized protein OS=Oryctolagus cuniculus OX=9986 GN=CPT2 PE=3 SV=2	40.2	0.905556779	0.40341817	0.6698973
G1SLD1$$Uncharacterized protein OS=Oryctolagus cuniculus OX=9986 GN=SORBS2 PE=4 SV=1	20.733333	0.905373044	0.40350731	0.6698973
G1SQU0$$Uncharacterized protein OS=Oryctolagus cuniculus OX=9986 GN=RAB5B PE=4 SV=1	27.666667	0.904865067	0.40375382	0.6698973
G1TCK9$$V-type proton ATPase subunit a OS=Oryctolagus cuniculus OX=9986 GN=ATP6V0A1 PE=3 SV=2	27.5	0.901038252	0.40561466	0.6723801
G1TAC3$$Uncharacterized protein OS=Oryctolagus cuniculus OX=9986 GN=RDH14 PE=3 SV=1	40.1	0.897048201	0.40756192	0.6725137
G1SYT7$$ABHD14A-ACY1 readthrough (Fragment) OS=Homo sapiens GN=ABHD14A-ACY1 PE=4 SV=1	34.3	0.89696269	0.40760373	0.6725137
G1U9R0$$Uncharacterized protein OS=Oryctolagus cuniculus OX=9986 GN=DHRS1 PE=4 SV=1	7.6	0.896454006	0.40785252	0.6725137
G1SZ76$$Interleukin enhancer binding factor 3 OS=Oryctolagus cuniculus OX=9986 GN=ILF3 PE=4 SV=1	29.033333	0.896373819	0.40789175	0.6725137
G1T346$$Latent-transforming growth factor beta-binding protein 4 OS=Homo sapiens GN=LTBP4 PE=1 SV=2	13.966667	0.89584147	0.40815226	0.6725137
G1SWK5$$Aldehyde dehydrogenase OS=Oryctolagus cuniculus OX=9986 GN=ALDH3A2 PE=3 SV=2	44.954881	0.895721088	0.40821118	0.6725137
G1SIG2$$Uncharacterized protein OS=Oryctolagus cuniculus OX=9986 GN=HNRNPF PE=4 SV=1	8.3666667	0.895648336	0.4082468	0.6725137
G1SI26$$Uncharacterized protein OS=Oryctolagus cuniculus OX=9986 GN=BAG6 PE=4 SV=1	24.433333	0.894480265	0.40881895	0.6728555
G1SV26$$Uncharacterized protein OS=Oryctolagus cuniculus OX=9986 GN=TOM1 PE=3 SV=1	48.234958	0.890349762	0.41084714	0.6755397
G1STW0$$Uncharacterized protein OS=Oryctolagus cuniculus OX=9986 GN=NDUFA9 PE=4 SV=2	16.766667	0.889339201	0.41134453	0.6755397
G1TAQ7$$NIF3-like protein 1 OS=Oryctolagus cuniculus OX=9986 GN=NIF3L1 PE=3 SV=1	-9.366667	-0.888190393	0.41191053	0.6755397
G1T765$$Uncharacterized protein OS=Oryctolagus cuniculus OX=9986 GN=NAPG PE=4 SV=1	7.5333333	0.88818242	0.41191446	0.6755397
G1SKK9$$Uncharacterized protein OS=Oryctolagus cuniculus OX=9986 GN=SLC25A13 PE=3 SV=2	32.133333	0.887090128	0.41245316	0.6756647

G1T3E6$$Uncharacterized protein OS=Oryctolagus cuniculus OX=9986 GN=DNAJC19 PE=4 SV=1	17.7	0.886543199	0.41272311	0.6756647
G1U9R8$$Glutathione reductase OS=Oryctolagus cuniculus OX=9986 GN=GSR PE=3 SV=1	-44.8	-0.881986449	0.41497741	0.6787529
G1ST15$$Uncharacterized protein OS=Oryctolagus cuniculus OX=9986 GN=CPSF6 PE=4 SV=1	40.9	0.880187401	0.41587001	0.6796104
G1SH86$$Uncharacterized protein OS=Oryctolagus cuniculus OX=9986 GN=SEPT11 PE=3 SV=2	21.633333	0.878780973	0.41656884	0.68015
G1SXT7$$Electron transfer flavoprotein subunit beta OS=Homo sapiens GN=ETFB PE=1 SV=3	26.466667	0.877800468	0.41705657	0.6803443
G1SGL4$$Cytosolic 10-formyltetrahydrofolate dehydrogenase OS=Homo sapiens GN=ALDH1L1 PE=1 SV=2	34.266667	0.870204509	0.42084972	0.6858232
G1TPD4$$Uncharacterized protein OS=Oryctolagus cuniculus OX=9986 GN=VPS4A PE=3 SV=2	64.795758	0.869588143	0.42115865	0.6858232
G1SFE0$$Uncharacterized protein OS=Oryctolagus cuniculus OX=9986 GN=THRAP3 PE=4 SV=1	21.133333	0.868397969	0.42175569	0.6861898
G1SZ85$$ATPase family AAA domain-containing protein 3B OS=Homo sapiens GN=ATAD3B PE=1 SV=1	25.4	0.866685588	0.4226158	0.6867878
G1SIH1$$Uncharacterized protein OS=Oryctolagus cuniculus OX=9986 GN=KNG1 PE=4 SV=2	7.8	0.866184493	0.42286775	0.6867878
G1SU89$$Catenin alpha-1 OS=Oryctolagus cuniculus OX=9986 GN=CTNNA1 PE=4 SV=1	29.966667	0.86219434	0.42487803	0.6894459
G1SS85$$Echinoderm microtubule associated protein like 3 OS=Oryctolagus cuniculus OX=9986 GN=EML3 PE=4 SV=1	36.081529	0.85966184	0.42615767	0.6901419
G1TQR0$$Melanoma cell adhesion molecule OS=Oryctolagus cuniculus OX=9986 GN=MCAM PE=4 SV=1	-85.73333	-0.858734704	0.42662687	0.6901419
G1TCD4$$Protein disulfide-isomerase OS=Oryctolagus cuniculus OX=9986 GN=PDIA3 PE=3 SV=2	41.833333	0.858039096	0.42697915	0.6901419
G1U115$$Reticulon-3 OS=Homo sapiens GN=RTN3 PE=1 SV=2	35.366667	0.857812628	0.42709389	0.6901419
G1U001$$Uncharacterized protein OS=Oryctolagus cuniculus OX=9986 GN=TLN1 PE=4 SV=1	57.93429	0.857648069	0.42717728	0.6901419
G1SVP7$$Uncharacterized protein OS=Oryctolagus cuniculus OX=9986 GN=PCYOX1 PE=3 SV=2	21.166667	0.855782207	0.42812364	0.6910657

G1TFZ5$$Septin-7 OS=Homo sapiens GN=SEPT7 PE=1 SV=2	33.633333	0.85465297	0.42869716	0.6913866
G1T5V3$$Aspartate beta-hydroxylase OS=Oryctolagus cuniculus OX=9986 GN=ASPH PE=4 SV=1	22.266667	0.85009349	0.43101869	0.6945236
G1T593$$Uncharacterized protein OS=Oryctolagus cuniculus OX=9986 GN=PML PE=4 SV=1	36.933333	0.848839958	0.43165859	0.6949477
G1TP15$$Delta-1-pyrroline-5-carboxylate synthase OS=Oryctolagus cuniculus OX=9986 GN=ALDH18A1 PE=3 SV=1	15.066667	0.846080651	0.43306968	0.6952484
G1TJR3$$Uncharacterized protein OS=Oryctolagus cuniculus OX=9986 GN=TKT PE=4 SV=2	38.066667	0.845981937	0.43312023	0.6952484
G1TC19$$Uncharacterized protein OS=Oryctolagus cuniculus OX=9986 GN=ACIN1 PE=4 SV=1	22.233333	0.845629288	0.43330084	0.6952484
G1TRW8$$Uncharacterized protein OS=Oryctolagus cuniculus OX=9986 GN=PADI2 PE=4 SV=2	35.266667	0.844699448	0.43377732	0.6952484
G1T4T3$$Uncharacterized protein OS=Oryctolagus cuniculus OX=9986 GN=ACADVL PE=3 SV=1	33.966667	0.844443413	0.43390858	0.6952484
G1SEL8$$Uncharacterized protein OS=Oryctolagus cuniculus OX=9986 GN=RHEB PE=4 SV=1	25.766667	0.844057907	0.43410629	0.6952484
G1U6F4$$Sorting and assembly machinery component 50 homolog OS=Homo sapiens GN=SAMM50 PE=1 SV=3	41.933333	0.840566932	0.43589969	0.6975151
G1T2C3$$Uncharacterized protein OS=Oryctolagus cuniculus OX=9986 GN=HM13 PE=4 SV=1	22.766667	0.839832417	0.43627773	0.6975151
U3KPJ9$$Uncharacterized protein OS=Oryctolagus cuniculus OX=9986 GN=USP7 PE=3 SV=1	26.6	0.837610973	0.43742256	0.6987399
U3KMH8$$Uncharacterized protein OS=Oryctolagus cuniculus OX=9986 GN=VTI1B PE=4 SV=1	24.133333	0.833307668	0.43964664	0.7012261
G1T6Z1$$Liver carboxylesterase 1 OS=Oryctolagus cuniculus OX=9986 PE=1 SV=3	19.48258	0.832537442	0.4400456	0.7012261
G1T840$$Uncharacterized protein OS=Oryctolagus cuniculus OX=9986 GN=SRSF7 PE=4 SV=2	33.8	0.832229581	0.44020514	0.7012261
G1TZ38$$Cytochrome c heme lyase OS=Oryctolagus cuniculus OX=9986 GN=HCCS PE=3 SV=2	-18.16667	-0.831630799	0.44051556	0.7012261
G1U974$$60S ribosomal protein L18a OS=Oryctolagus cuniculus OX=9986 GN=RPL18A PE=1 SV=1	-17.76667	-0.830929672	0.44087925	0.7012261
G1SDU5$$Uncharacterized protein OS=Oryctolagus cuniculus OX=9986 GN=CFB PE=3 SV=2	34.733333	0.830049929	0.44133591	0.7013478

G1SHF9$$40S ribosomal protein S28 OS=Homo sapiens GN=RPS28 PE=1 SV=1	30.266667	0.828511049	0.44213556	0.7018618
G1SQ70$$Moesin OS=Homo sapiens GN=MSN PE=1 SV=3	-57.56667	-0.827852664	0.442478	0.7018618
G1U018$$Uncharacterized protein OS=Oryctolagus cuniculus OX=9986 GN=SRSF1 PE=4 SV=2	28.333333	0.825733658	0.44358148	0.7018618
G1T2T9$$ATP-dependent RNA helicase DDX3X OS=Homo sapiens GN=DDX3X PE=1 SV=1	38.450446	0.82531492	0.44379978	0.7018618
G1TUM0$$Uncharacterized protein OS=Oryctolagus cuniculus OX=9986 GN=RBM39 PE=4 SV=1	20.466667	0.824846612	0.44404402	0.7018618
G1TJN8$$""ATP synthase subunit delta, mitochondrial OS=Homo sapiens GN=ATP5D PE=1 SV=2""	8.2	0.82434739	0.44430449	0.7018618
G1TLQ8$$Uncharacterized protein OS=Oryctolagus cuniculus OX=9986 PE=3 SV=1	22.033333	0.824313285	0.44432229	0.7018618
G1T5C5$$Uncharacterized protein OS=Oryctolagus cuniculus OX=9986 GN=PLS3 PE=4 SV=2	12.9	0.821778781	0.44564644	0.7033513
G1TES2$$Ubiquitin thioesterase OTUB1 OS=Homo sapiens GN=OTUB1 PE=1 SV=1	14.766667	0.818813035	0.44719959	0.7051994
P47814$$40S ribosomal protein S8 OS=Oryctolagus cuniculus OX=9986 GN=LOC100352057 PE=1 SV=1	24.1	0.817361979	0.44796095	0.7057635
G1SH26$$Uncharacterized protein OS=Oryctolagus cuniculus OX=9986 GN=FGGY PE=4 SV=1	15.133333	0.816673748	0.4483224	0.7057635
G1TB18$$Malectin OS=Homo sapiens GN=MLEC PE=1 SV=1	17.833333	0.814052754	0.44970085	0.70733
G1SKD9$$""Succinate--CoA ligase [GDP-forming] subunit beta, mitochondrial OS=Oryctolagus cuniculus OX=9986 GN=SUCLG2 PE=3 SV=1""	22.033333	0.810125066	0.45177234	0.7088303
P02251$$Uncharacterized protein OS=Oryctolagus cuniculus OX=9986 GN=HNRNPA2B1 PE=4 SV=1	26.766667	0.810107762	0.45178149	0.7088303
G1U466$$Uncharacterized protein OS=Oryctolagus cuniculus OX=9986 GN=SCO1 PE=4 SV=1	9.4	0.808594753	0.45258133	0.7088303
G1T726$$Phosphomannomutase OS=Oryctolagus cuniculus OX=9986 GN=PMM2 PE=3 SV=1	32.266667	0.808456896	0.45265426	0.7088303
G1SL95$$Uncharacterized protein OS=Oryctolagus cuniculus OX=9986 GN=VSNL1 PE=4 SV=1	29.766667	0.808275758	0.4527501	0.7088303

G1TSL1$$""PGAM family member 5, mitochondrial serine/threonine protein phosphatase OS=Oryctolagus cuniculus OX=9986 GN=PGAM5 PE=4 SV=2""	26.933333	0.807879404	0.45295986	0.7088303
G1T8R2$$Apolipoprotein E OS=Homo sapiens GN=APOE PE=1 SV=1	27.166667	0.806115941	0.45389398	0.7088669
G1U535$$Superoxide dismutase [Cu-Zn] OS=Oryctolagus cuniculus OX=9986 GN=SOD1 PE=3 SV=1	28.533333	0.805967646	0.4539726	0.7088669
U3KMD1$$Uncharacterized protein OS=Oryctolagus cuniculus OX=9986 GN=CLPB PE=4 SV=2	19.566667	0.80547372	0.45423452	0.7088669
G1TPI0$$Uncharacterized protein OS=Oryctolagus cuniculus OX=9986 GN=TXNRD1 PE=3 SV=2	8.5666667	0.804616803	0.4546892	0.7088669
G1TCL7$$F-actin-capping protein subunit alpha-2 OS=Oryctolagus cuniculus OX=9986 GN=CAPZA2 PE=3 SV=3	33.566667	0.803952279	0.45504201	0.7088669
G1TCZ8$$Uncharacterized protein OS=Oryctolagus cuniculus OX=9986 GN=EPB42 PE=4 SV=2	23.2	0.800854957	0.45668912	0.7088669
G1T520$$""Alpha-1,4 glucan phosphorylase OS=Oryctolagus cuniculus OX=9986 GN=PYGB PE=3 SV=1""	37.7	0.798738366	0.45781719	0.7088669
P47845$$Uncharacterized protein OS=Oryctolagus cuniculus OX=9986 GN=METTL7A PE=4 SV=1	-32.6	-0.79853201	0.45792728	0.7088669
G1T134$$Uncharacterized protein OS=Oryctolagus cuniculus OX=9986 GN=PALLD PE=4 SV=2	32.266667	0.798171559	0.45811962	0.7088669
G1TWP4$$Indolethylamine N-methyltransferase OS=Oryctolagus cuniculus OX=9986 GN=INMT PE=1 SV=1	21.933333	0.797089757	0.45869724	0.7088669
G1T2V4$$S-adenosylmethionine synthase isoform type-2 OS=Homo sapiens GN=MAT2A PE=1 SV=1	8.0333333	0.796595136	0.45896151	0.7088669
G1TDU0$$Retinal dehydrogenase 1 OS=Oryctolagus cuniculus OX=9986 GN=ALDH1A1 PE=1 SV=1	16.833333	0.796346225	0.45909454	0.7088669
G1TYA7$$Uncharacterized protein OS=Oryctolagus cuniculus OX=9986 GN=MARC1 PE=4 SV=1	14.9	0.796269231	0.4591357	0.7088669
G1T338$$Uncharacterized protein OS=Oryctolagus cuniculus OX=9986 GN=DDX1 PE=4 SV=2	36.066667	0.79558315	0.45950255	0.7088669
G1SDF2$$Adenylosuccinate synthetase isozyme 2 OS=Oryctolagus cuniculus OX=9986 GN=ADSS PE=3 SV=1	21.9	0.79525137	0.45968004	0.7088669

G1SX73$$60S ribosomal protein L6 OS=Oryctolagus cuniculus OX=9986 GN=RPL6 PE=1 SV=1	30.233333	0.795236569	0.45968795	0.7088669
G1T461$$Uncharacterized protein OS=Oryctolagus cuniculus OX=9986 GN=RAB8B PE=4 SV=2	21.1	0.794055281	0.46032028	0.7088669
G1TED6$$40S ribosomal protein S12 OS=Homo sapiens GN=RPS12 PE=1 SV=3	-23.73333	-0.793623108	0.46055178	0.7088669
G1TKE3$$Small nuclear ribonucleoprotein Sm D3 OS=Oryctolagus cuniculus OX=9986 GN=SNRPD3 PE=3 SV=1	-27.36667	-0.793176844	0.46079091	0.7088669
G1T0L9$$""Succinate dehydrogenase [ubiquinone] flavoprotein subunit, mitochondrial OS=Oryctolagus cuniculus OX=9986 GN=SDHA PE=3 SV=1""	18.4	0.792972381	0.4609005	0.7088669
G1T0R9$$""Adenylate kinase 2, mitochondrial OS=Oryctolagus cuniculus OX=9986 GN=AK2 PE=3 SV=1""	30.7	0.792430595	0.46119098	0.7088669
G1TC33$$Uncharacterized protein OS=Oryctolagus cuniculus OX=9986 GN=C5 PE=4 SV=2	16.866667	0.791672672	0.46159758	0.7088669
G1T3I9$$Uncharacterized protein OS=Oryctolagus cuniculus OX=9986 GN=RAD23B PE=4 SV=1	16.2	0.791258143	0.46182006	0.7088669
G1SEE8$$Cysteine and glycine-rich protein 2 OS=Homo sapiens GN=CSRP2 PE=1 SV=1	6.6666667	0.786501463	0.46437862	0.7111943
G1TG27$$Uncharacterized protein OS=Oryctolagus cuniculus OX=9986 PE=4 SV=1	29.133333	0.78629966	0.46448739	0.7111943
G1SSL2$$Protein disulfide-isomerase OS=Oryctolagus cuniculus OX=9986 GN=PDIA4 PE=3 SV=1	30.333333	0.785602359	0.46486338	0.7111943
G1TEV2$$Uncharacterized protein OS=Oryctolagus cuniculus OX=9986 GN=NDRG2 PE=4 SV=1	28.6	0.78533242	0.46500899	0.7111943
G1T916$$SH3 domain-binding glutamic acid-rich-like protein OS=Oryctolagus cuniculus OX=9986 GN=SH3BGRL3 PE=3 SV=1	-19.3	-0.784860364	0.46526371	0.7111943
G1T4P7$$Microtubule-associated protein 1B OS=Homo sapiens GN=MAP1B PE=1 SV=2	20.9	0.781410581	0.46712823	0.7131931
G1T657$$Serine/threonine-protein phosphatase PP1-alpha catalytic subunit OS=Oryctolagus cuniculus OX=9986 GN=PPP1CA PE=1 SV=1	-6.6	-0.780583163	0.46757623	0.7131931
G1SWK8$$Uncharacterized protein OS=Oryctolagus cuniculus OX=9986 GN=SAR1A PE=3 SV=1	17.133333	0.780001101	0.46789156	0.7131931

G1SQY9$$Clathrin light chain OS=Oryctolagus cuniculus OX=9986 GN=CLTA PE=3 SV=1	19.333333	0.779584129	0.46811755	0.7131931
G1SNQ8$$Clathrin light chain B OS=Homo sapiens GN=CLTB PE=1 SV=1	-26.33333	-0.778094544	0.46892551	0.7138346
G1SS69$$DnaJ homolog subfamily C member 25 OS=Homo sapiens GN=DNAJC25 PE=1 SV=1	-23.96667	-0.77541688	0.47038041	0.714938
G1TA80$$Uncharacterized protein OS=Oryctolagus cuniculus OX=9986 PE=4 SV=2	16	0.775033491	0.47058899	0.714938
G1SRL4$$Peptidyl-prolyl cis-trans isomerase-like 1 OS=Homo sapiens GN=PPIL1 PE=1 SV=1	-26.6	-0.774622193	0.47081282	0.714938
G1U636$$Uncharacterized protein OS=Oryctolagus cuniculus OX=9986 GN=SNX2 PE=4 SV=1	27.733333	0.772306558	0.47207444	0.7151321
G1SKY7$$Hemoglobin subunit beta OS=Homo sapiens GN=HBB PE=1 SV=2	21.766667	0.772191323	0.47213728	0.7151321
G1SIB9$$Polypyrimidine tract-binding protein 3 OS=Homo sapiens GN=PTBP3 PE=1 SV=2	-21.33333	-0.772179326	0.47214383	0.7151321
G1T3G6$$rRNA 2'-O-methyltransferase fibrillarin OS=Homo sapiens GN=FBL PE=1 SV=2	37.066667	0.771542851	0.47249105	0.7151321
G1SN05$$Sulfotransferase OS=Oryctolagus cuniculus OX=9986 GN=SULT1C4 PE=3 SV=2	36.866667	0.768191105	0.47432258	0.7168748
G1TC03$$Protein disulfide-isomerase OS=Oryctolagus cuniculus OX=9986 GN=P4HB PE=2 SV=1	21.6	0.767983109	0.47443641	0.7168748
G1U1T9$$Uncharacterized protein OS=Oryctolagus cuniculus OX=9986 GN=DCTN4 PE=4 SV=2	45.6	0.767304097	0.47480813	0.7168748
G1TCE2$$Apoptosis inhibitor 5 OS=Homo sapiens GN=API5 PE=1 SV=3	30.133333	0.765593743	0.47574536	0.7177025
G1TP59$$Uncharacterized protein OS=Oryctolagus cuniculus OX=9986 GN=RPL35A PE=1 SV=1	27.833333	0.762624652	0.47737546	0.7185696
G1TWM4$$Glutathione S-transferase Mu 1 OS=Homo sapiens GN=GSTM1 PE=1 SV=1	21.933333	0.76257078	0.47740508	0.7185696
G1U8B3$$Uncharacterized protein OS=Oryctolagus cuniculus OX=9986 GN=PIP4K2A PE=4 SV=1	24.133333	0.762418997	0.47748852	0.7185696
G1SL57$$""ATP synthase subunit d, mitochondrial OS=Oryctolagus cuniculus OX=9986 GN=ATP5PD PE=3 SV=1""	7.4	0.761605786	0.47793576	0.7186565

G1TBL1$$Cytochrome b-245 chaperone 1 OS=Oryctolagus cuniculus OX=9986 GN=CYBC1 PE=4 SV=2	26.066667	0.760392742	0.47860344	0.7190744
G1TI40$$Uncharacterized protein OS=Oryctolagus cuniculus OX=9986 GN=NIPSNAP1 PE=4 SV=1	22.166667	0.758767883	0.47949883	0.7193341
G1SQ38$$""Sterol 26-hydroxylase, mitochondrial OS=Oryctolagus cuniculus OX=9986 GN=CYP27A1 PE=1 SV=1""	26.533333	0.758664063	0.47955608	0.7193341
G1TTJ1$$Alpha-actinin-1 OS=Homo sapiens GN=ACTN1 PE=1 SV=1	23.3	0.756798265	0.48058578	0.7202931
G1SRT1$$Uncharacterized protein OS=Oryctolagus cuniculus OX=9986 GN=SEC22B PE=3 SV=1	27.566667	0.755193503	0.48147267	0.7210366
G1T0Y9$$Coatomer subunit beta OS=Oryctolagus cuniculus OX=9986 GN=COPB1 PE=4 SV=1	18.266667	0.752426387	0.48300464	0.7227033
G1SKF1$$Transcription intermediary factor 1-beta OS=Homo sapiens GN=TRIM28 PE=1 SV=5	-29.93333	-0.751489144	0.48352431	0.7227033
G1SQA8$$FUS RNA binding protein OS=Oryctolagus cuniculus OX=9986 GN=FUS PE=4 SV=2	44.1	0.750709278	0.48395702	0.7227033
P30947$$Protein phosphatase methylesterase 1 OS=Oryctolagus cuniculus OX=9986 GN=PPME1 PE=3 SV=1	29.666667	0.749805482	0.48445883	0.7227033
G1T2U1$$Uncharacterized protein OS=Oryctolagus cuniculus OX=9986 PE=4 SV=1	31.133333	0.748904637	0.48495937	0.7227033
G1SL60$$Dolichyl-diphosphooligosaccharide--protein glycosyltransferase 48 kDa subunit OS=Oryctolagus cuniculus OX=9986 GN=DDOST PE=3 SV=1	24.833333	0.748439006	0.48521823	0.7227033
G1T4H0$$Biliverdin reductase B OS=Oryctolagus cuniculus OX=9986 GN=BLVRB PE=4 SV=2	39.994717	0.748242408	0.48532755	0.7227033
G1U7L1$$40S ribosomal protein S6 OS=Oryctolagus cuniculus OX=9986 GN=LOC108178879 PE=1 SV=1	24.566667	0.745373718	0.48692474	0.7235553
G1SHP3$$Uncharacterized protein OS=Oryctolagus cuniculus OX=9986 GN=ETFDH PE=4 SV=1	8.4	0.745102849	0.48707574	0.7235553
G1SWC0$$Uncharacterized protein OS=Oryctolagus cuniculus OX=9986 GN=PDIA6 PE=3 SV=2	38.200529	0.744616487	0.48734696	0.7235553
G1SD83$$S-phase kinase-associated protein 1 OS=Homo sapiens GN=SKP1 PE=1 SV=1	28.2	0.744398668	0.48746845	0.7235553

G1SST9$$""Serine hydroxymethyltransferase, mitochondrial OS=Oryctolagus cuniculus OX=9986 GN=SHMT2 PE=1 SV=2""	20.7	0.743402056	0.48802463	0.7237986
G1U9S7$$Eukaryotic translation initiation factor 3 subunit C-like protein OS=Homo sapiens GN=EIF3CL PE=3 SV=1	24.633333	0.741962522	0.48882876	0.7244089
G1SF30$$Vacuolar protein sorting-associated protein 35 OS=Oryctolagus cuniculus OX=9986 GN=VPS35 PE=3 SV=1	17.466667	0.739583887	0.49015951	0.725169
G1SIT6$$Uncharacterized protein OS=Oryctolagus cuniculus OX=9986 GN=TWF1 PE=4 SV=1	29.266667	0.738943353	0.49051829	0.725169
G1U8Y1$$Cellular nucleic acid-binding protein OS=Homo sapiens GN=CNBP PE=1 SV=1	-14.03333	-0.738938856	0.49052081	0.725169
G1T7Z6$$Ras-related protein Rab-21 OS=Homo sapiens GN=RAB21 PE=1 SV=3	9.4333333	0.736338843	0.49197904	0.7264062
G1TVS4$$Eukaryotic translation initiation factor 3 subunit H OS=Homo sapiens GN=EIF3S3 PE=1 SV=1	44.2	0.736043086	0.4921451	0.7264062
G1SVJ5$$Uncharacterized protein OS=Oryctolagus cuniculus OX=9986 PE=4 SV=2	19.766667	0.734600981	0.4929554	0.7270206
U3KMC6$$Uncharacterized protein OS=Oryctolagus cuniculus OX=9986 GN=SFXN3 PE=4 SV=2	29.9	0.729821006	0.49564781	0.7300449
G1SGQ5$$Nicalin OS=Homo sapiens GN=NCLN PE=1 SV=2	-46.96667	-0.729410046	0.49587976	0.7300449
G1SDU6$$""Elongation factor G, mitochondrial OS=Oryctolagus cuniculus OX=9986 GN=GFM1 PE=3 SV=1""	19.433333	0.728609992	0.49633155	0.7300449
G1TEU5$$Uncharacterized protein OS=Oryctolagus cuniculus OX=9986 GN=EIF5B PE=4 SV=1	27.4	0.727513419	0.49695123	0.7300449
G1SU30$$3alpha/17beta/20alpha-hydroxysteroid dehydrogenase OS=Oryctolagus cuniculus OX=9986 GN=PGER5 PE=2 SV=1	31.8	0.727454613	0.49698448	0.7300449
G1SDJ3$$Reticulocalbin-1 OS=Homo sapiens GN=RCN1 PE=1 SV=1	40.92865	0.726302571	0.49763611	0.7304205
G1TDX2$$Uncharacterized protein OS=Oryctolagus cuniculus OX=9986 GN=ACTN1 PE=4 SV=1	33.232796	0.724610998	0.49859399	0.7311805
G1SE27$$Carboxypeptidase OS=Oryctolagus cuniculus OX=9986 GN=SCPEP1 PE=3 SV=1	24.766667	0.723989128	0.49894646	0.7311805
G1T810$$Uncharacterized protein OS=Oryctolagus cuniculus OX=9986 GN=ACSM5 PE=4 SV=2	37.522662	0.723129059	0.49943421	0.7313144

G1SN68$$Leukotriene A(4) hydrolase OS=Oryctolagus cuniculus OX=9986 GN=LTA4H PE=3 SV=1	24.733333	0.721832085	0.50017035	0.7318115
G1SZ35$$Probable ATP-dependent RNA helicase DDX49 OS=Homo sapiens GN=DDX49 PE=1 SV=1	7.9666667	0.719955116	0.50123701	0.7322688
G1SH10$$Uncharacterized protein OS=Oryctolagus cuniculus OX=9986 GN=SAT2 PE=4 SV=1	37.633333	0.719307365	0.50160548	0.7322688
G1SPR5$$Eukaryotic translation initiation factor 3 subunit J OS=Oryctolagus cuniculus OX=9986 GN=EIF3J PE=3 SV=1	-25.5	-0.719187687	0.50167358	0.7322688
G1T706$$""Cytochrome c oxidase subunit 7A2, mitochondrial OS=Homo sapiens GN=COX7A2 PE=1 SV=1""	39.633333	0.716293521	0.50332233	0.733352
G1TH59$$Serine/threonine-protein kinase PAK 2 OS=Oryctolagus cuniculus OX=9986 GN=PAK2 PE=2 SV=1	29.533333	0.716031999	0.5034715	0.733352
G1U541$$Uncharacterized protein OS=Oryctolagus cuniculus OX=9986 GN=SERPINC1 PE=3 SV=1	32.733333	0.715792529	0.50360812	0.733352
G1SJJ9$$Signal recognition particle 9 kDa protein OS=Oryctolagus cuniculus OX=9986 GN=SRP9 PE=3 SV=1	27.933333	0.712188594	0.50566719	0.7352272
G1T4N5$$Uncharacterized protein OS=Oryctolagus cuniculus OX=9986 GN=TECR PE=4 SV=2	28.566667	0.710499423	0.50663425	0.7352272
G1SE51$$Uncharacterized protein OS=Oryctolagus cuniculus OX=9986 GN=TXNL1 PE=4 SV=1	13.051189	0.710348637	0.50672063	0.7352272
G1SCK0$$ATP synthase subunit beta OS=Oryctolagus cuniculus OX=9986 GN=ATP5F1B PE=3 SV=2	30.423301	0.710104683	0.50686042	0.7352272
G1T512$$Uncharacterized protein OS=Oryctolagus cuniculus OX=9986 GN=LAP3 PE=3 SV=2	21.2	0.71005594	0.50688835	0.7352272
G1TAP1$$Uncharacterized protein OS=Oryctolagus cuniculus OX=9986 GN=CAVIN1 PE=4 SV=1	17.4	0.708036943	0.50804629	0.7356195
G1TE96$$Abhydrolase domain containing 11 OS=Oryctolagus cuniculus OX=9986 GN=ABHD11 PE=4 SV=2	7.3666667	0.707695247	0.50824243	0.7356195
G1SNT8$$Glucose-6-phosphate 1-dehydrogenase OS=Oryctolagus cuniculus OX=9986 GN=G6PD PE=3 SV=2	6.2666667	0.707499244	0.50835497	0.7356195
G1SIV3$$Lactoylglutathione lyase OS=Oryctolagus cuniculus OX=9986 GN=BTBD9 PE=3 SV=1	42.667971	0.70633761	0.50902228	0.7357196

G1SZ14$$T-complex protein 1 subunit gamma OS=Oryctolagus cuniculus OX=9986 GN=CCT3 PE=3 SV=2	13.4	0.705990769	0.50922164	0.7357196
G1TPV7$$Latent-transforming growth factor beta-binding protein 1 OS=Homo sapiens GN=LTBP1 PE=1 SV=4	5.8666667	0.704692135	0.50996855	0.7358207
G1SCZ9$$Uncharacterized protein OS=Oryctolagus cuniculus OX=9986 GN=ATP5MG PE=4 SV=1	18.266667	0.704482399	0.51008925	0.7358207
G1SM21$$Poly(A) polymerase alpha OS=Oryctolagus cuniculus OX=9986 GN=PAPOLA PE=4 SV=1	6.8837781	0.702278787	0.51135856	0.7370064
G1TC70$$Ras-related protein Rap-1b OS=Homo sapiens GN=RAP1B PE=1 SV=1	-22.26667	-0.701669058	0.51171015	0.7370064
G1TVY5$$Uncharacterized protein OS=Oryctolagus cuniculus OX=9986 GN=C1QB PE=4 SV=2	26.733333	0.70044183	0.5124183	0.7374507
G1TFL3$$Uncharacterized protein OS=Oryctolagus cuniculus OX=9986 GN=PCBD1 PE=3 SV=1	-28.7	-0.693993088	0.51615031	0.7422427
G1TD51$$""Myosin light chain kinase, smooth muscle OS=Oryctolagus cuniculus OX=9986 GN=MYLK PE=1 SV=2""	-8.266667	-0.692922313	0.51677175	0.7425575
G1T1Z5$$Uncharacterized protein OS=Oryctolagus cuniculus OX=9986 GN=MAVS PE=4 SV=1	40.3253	0.687968199	0.51965348	0.7461172
G1SU97$$Phosphoglycerate mutase OS=Oryctolagus cuniculus OX=9986 GN=PGAM2 PE=3 SV=1	21.566667	0.686380802	0.52057911	0.746865
G1SNZ3$$Uncharacterized protein OS=Oryctolagus cuniculus OX=9986 GN=UQCRC1 PE=4 SV=1	21.9	0.685488911	0.52109966	0.747031
G1T3X8$$Eukaryotic translation initiation factor 3 subunit A OS=Oryctolagus cuniculus OX=9986 GN=EIF3A PE=1 SV=2	-9.433333	-0.682921449	0.5226001	0.7481717
G1SM91$$Uncharacterized protein OS=Oryctolagus cuniculus OX=9986 GN=RAB13 PE=4 SV=1	32.966667	0.68273971	0.52270642	0.7481717
G1SRB7$$Uncharacterized protein OS=Oryctolagus cuniculus OX=9986 PE=4 SV=1	9.2333333	0.68013307	0.5242329	0.749775
G1SVU0$$Glucose-6-phosphate 1-dehydrogenase OS=Homo sapiens GN=G6PD PE=1 SV=4	31	0.678574202	0.52514721	0.7503112
G1SN00$$Probable cytosolic iron-sulfur protein assembly protein CIAO1 OS=Oryctolagus cuniculus OX=9986 GN=CIAO1 PE=3 SV=1	-14.16667	-0.67810739	0.52542121	0.7503112
G1SGI6$$Uncharacterized protein OS=Oryctolagus cuniculus OX=9986 GN=RAB5C PE=4 SV=1	-37.63333	-0.676964543	0.52609241	0.7503764

G1TBC1$$ProSAAS OS=Homo sapiens GN=PCSK1N PE=1 SV=1	5.9	0.676644889	0.52628025	0.7503764
G1TKP3$$""Cytochrome c oxidase subunit 7C, mitochondrial OS=Homo sapiens GN=COX7C PE=1 SV=1""	-19.66667	-0.675134096	0.52716863	0.7510626
G1SK79$$Triosephosphate isomerase OS=Oryctolagus cuniculus OX=9986 GN=TPI1 PE=1 SV=2	-24.6	-0.671303749	0.5294254	0.7536959
G1T024$$Protein disulfide-isomerase A3 (Fragment) OS=Homo sapiens GN=PDIA3 PE=1 SV=1	7.4333333	0.670123921	0.53012181	0.7541054
G1U1M3$$Protein S100-A6 OS=Oryctolagus cuniculus OX=9986 GN=S100A6 PE=1 SV=2	15.6	0.668752534	0.53093205	0.7546761
G1TUC8$$Uncharacterized protein OS=Oryctolagus cuniculus OX=9986 GN=FAM120A PE=4 SV=2	-29.43333	-0.666280124	0.53239485	0.7561728
G1TKY3$$Aminopeptidase OS=Oryctolagus cuniculus OX=9986 GN=ERAP1 PE=3 SV=2	-10.23333	-0.661364975	0.53531073	0.7597295
G1SQP9$$Uncharacterized protein OS=Oryctolagus cuniculus OX=9986 GN=ECHDC3 PE=4 SV=1	14.433333	0.660285332	0.53595262	0.7600558
G1T822$$Uncharacterized protein OS=Oryctolagus cuniculus OX=9986 GN=CACYBP PE=4 SV=1	24.533333	0.658592562	0.53696004	0.7603616
G1SNS5$$Uncharacterized protein OS=Oryctolagus cuniculus OX=9986 GN=CENPV PE=4 SV=1	6.6666667	0.658401458	0.53707385	0.7603616
G1SM51$$Actin-related protein 2/3 complex subunit 4 OS=Oryctolagus cuniculus OX=9986 GN=ARPC4 PE=3 SV=1	24.4	0.657846155	0.53740464	0.7603616
G1SSJ8$$Small glutamine-rich tetratricopeptide repeat-containing protein alpha OS=Homo sapiens GN=SGTA PE=1 SV=1	20.933333	0.657108765	0.5378441	0.7604003
G1U5N5$$Carboxylic ester hydrolase OS=Oryctolagus cuniculus OX=9986 GN=LOC100351488 PE=3 SV=1	44.3	0.655792104	0.53862938	0.7609274
G1TME7$$""Tryptophan--tRNA ligase, cytoplasmic OS=Oryctolagus cuniculus OX=9986 GN=WARS PE=3 SV=1""	-27.56667	-0.653714219	0.53987018	0.7620968
G1SNE3$$Transmembrane 7 superfamily member 2 OS=Oryctolagus cuniculus OX=9986 GN=TM7SF2 PE=4 SV=1	8.2333333	0.650488788	0.54179989	0.7642361
G1T329$$Uncharacterized protein OS=Oryctolagus cuniculus OX=9986 GN=LYPLA2 PE=4 SV=1	23.766667	0.649192482	0.5425767	0.7643194

G1SLQ3$$Uncharacterized protein OS=Oryctolagus cuniculus OX=9986 GN=PRXL2A PE=4 SV=1	23.8	0.649007732	0.54268747	0.7643194
G1SHV1$$Core histone macro-H2A.1 OS=Homo sapiens GN=H2AFY PE=1 SV=4	29.1	0.647668902	0.54349062	0.7644892
G1SMY6$$Uncharacterized protein OS=Oryctolagus cuniculus OX=9986 GN=SF3B3 PE=4 SV=1	36.496201	0.647163216	0.54379418	0.7644892
G1TUU7$$Glyceraldehyde-3-phosphate dehydrogenase OS=Oryctolagus cuniculus OX=9986 PE=3 SV=1	6.3666667	0.64673528	0.54405115	0.7644892
G1SFW9$$Malic enzyme OS=Oryctolagus cuniculus OX=9986 GN=ME2 PE=3 SV=1	26.566667	0.645716434	0.54466326	0.7647669
G1T2M0$$Apolipoprotein A-IV (Predicted) OS=Oryctolagus cuniculus OX=9986 GN=APOA4 PE=3 SV=1	-6.8	-0.643699249	0.54587648	0.7647815
G1TA78$$Fumarylacetoacetase OS=Oryctolagus cuniculus OX=9986 GN=FAH PE=3 SV=1	36	0.643678548	0.54588894	0.7647815
A7X8X3$$Cytoplasmic dynein 1 intermediate chain 2 OS=Homo sapiens GN=DYNC1I2 PE=1 SV=3	-18	-0.643095779	0.54623977	0.7647815
G1SKM5$$Uncharacterized protein OS=Oryctolagus cuniculus OX=9986 GN=PRDX3 PE=4 SV=1	19.5	0.642943069	0.54633172	0.7647815
G1SGQ0$$Uncharacterized protein OS=Oryctolagus cuniculus OX=9986 GN=THY1 PE=4 SV=1	26.366667	0.640187181	0.54799291	0.7649199
G1TQR2$$CAAX prenyl protease 1 homolog OS=Homo sapiens GN=ZMPSTE24 PE=1 SV=2	21.566667	0.639555059	0.5483744	0.7649199
G1TJ20$$Tubulin beta chain OS=Oryctolagus cuniculus OX=9986 GN=TUBB2A PE=3 SV=1	33.533333	0.639552048	0.54837622	0.7649199
G1T994$$Uncharacterized protein OS=Oryctolagus cuniculus OX=9986 GN=GRN PE=4 SV=1	6.7333333	0.639440296	0.54844368	0.7649199
G1SR03$$Uncharacterized protein OS=Oryctolagus cuniculus OX=9986 GN=HSPA2 PE=3 SV=1	8.6333333	0.639341159	0.54850353	0.7649199
G1SKJ5$$Signal peptidase complex subunit 3 OS=Oryctolagus cuniculus OX=9986 GN=LOC100343250 PE=3 SV=1	28.566667	0.637760035	0.54945862	0.7656731
G1SP51$$Uncharacterized protein OS=Oryctolagus cuniculus OX=9986 PE=4 SV=1	15.433333	0.63499319	0.55113251	0.767426
G1SJU8$$""Long-chain specific acyl-CoA dehydrogenase, mitochondrial OS=Homo sapiens GN=ACADL PE=1 SV=2""	22.8	0.632922987	0.55238707	0.767611
G1TQ31$$Uncharacterized protein OS=Oryctolagus cuniculus OX=9986 GN=FETUB PE=4 SV=1	26.066667	0.632800182	0.55246155	0.767611

G1TA95$$""NAD-dependent malic enzyme, mitochondrial OS=Homo sapiens GN=ME2 PE=1 SV=1""	17.133333	0.632109262	0.55288069	0.767611
G1U0Q7$$Uncharacterized protein OS=Oryctolagus cuniculus OX=9986 GN=PSMC5 PE=3 SV=1	-12.13333	-0.631682418	0.55313974	0.767611
G1TVT8$$Uncharacterized protein OS=Oryctolagus cuniculus OX=9986 GN=OCIAD2 PE=4 SV=1	28.7	0.630596524	0.55379909	0.767611
G1T3Z2$$Target of rapamycin complex subunit LST8 OS=Homo sapiens GN=MLST8 PE=1 SV=1	14.366667	0.630271071	0.55399681	0.767611
G1T983$$Cysteine and glycine-rich protein 1 (Fragment) OS=Homo sapiens GN=CSRP1 PE=1 SV=1	21.433333	0.629973367	0.5541777	0.767611
G1T7F1$$60S ribosomal protein L5 OS=Oryctolagus cuniculus OX=9986 GN=RPL5 PE=1 SV=1	-10.1	-0.628741464	0.55492664	0.7680718
G1SMH6$$Uncharacterized protein OS=Oryctolagus cuniculus OX=9986 GN=DCTN1 PE=4 SV=1	11.9	0.624539793	0.55748587	0.7703779
G1T519$$Signal recognition particle receptor subunit alpha OS=Homo sapiens GN=SRPRA PE=1 SV=2	18.133333	0.624152694	0.55772203	0.7703779
G1T580$$Surfeit locus protein 4 OS=Homo sapiens GN=SURF4 PE=1 SV=3	9.5	0.623662829	0.55802097	0.7703779
G1TX78$$Uncharacterized protein OS=Oryctolagus cuniculus OX=9986 GN=NCKAP1 PE=4 SV=1	21.766667	0.623266288	0.55826303	0.7703779
G1SI37$$Small nuclear ribonucleoprotein polypeptide A OS=Oryctolagus cuniculus OX=9986 GN=SNRPA PE=4 SV=1	5.3666667	0.622327358	0.55883645	0.7704549
G1TTQ5$$Uncharacterized protein OS=Oryctolagus cuniculus OX=9986 GN=ACTR1B PE=3 SV=1	14.966667	0.621807728	0.55915396	0.7704549
G1T0U8$$Uncharacterized protein OS=Oryctolagus cuniculus OX=9986 GN=RPS14 PE=1 SV=1	18.766667	0.620611988	0.55988501	0.7707903
G1T4P8$$Proteasome subunit beta OS=Oryctolagus cuniculus OX=9986 GN=PSMB5 PE=3 SV=1	18.666667	0.620043264	0.56023293	0.7707903
G1SLJ9$$60S ribosomal protein L13 OS=Homo sapiens GN=RPL13 PE=1 SV=4	10.3	0.618238129	0.56133813	0.7714459
G1T6D1$$Amine oxidase OS=Oryctolagus cuniculus OX=9986 GN=MAOA PE=3 SV=2	19.566667	0.617899262	0.56154575	0.7714459
G1U146$$Thimet oligopeptidase OS=Homo sapiens GN=THOP1 PE=1 SV=2	-9.666667	-0.615080165	0.56327487	0.7732456
G1U3M3$$Eukaryotic translation initiation factor 3 subunit I OS=Oryctolagus cuniculus OX=9986 GN=EIF3I PE=3 SV=1	20.1	0.614016936	0.56392788	0.7735665

G1T3S1$$Uncharacterized protein OS=Oryctolagus cuniculus OX=9986 GN=LOC100346472 PE=4 SV=1	27.466667	0.611683568	0.56536262	0.7748522
P37153$$General vesicular transport factor p115 OS=Homo sapiens GN=USO1 PE=1 SV=2	-28.06667	-0.611127082	0.56570513	0.7748522
G1SES2$$Uncharacterized protein OS=Oryctolagus cuniculus OX=9986 GN=IGF2R PE=4 SV=1	33.966667	0.609907036	0.5664565	0.7753058
G1SXH7$$Uncharacterized protein OS=Oryctolagus cuniculus OX=9986 GN=CMAS PE=4 SV=2	26.266667	0.606198297	0.56874435	0.7769081
G1SM52$$Uncharacterized protein OS=Oryctolagus cuniculus OX=9986 GN=BDH2 PE=4 SV=1	24.033333	0.605628146	0.56909657	0.7769081
G1SZV5$$Ig kappa chain V region AH80-5 OS=Oryctolagus cuniculus OX=9986 PE=1 SV=1	-48.13333	-0.60544841	0.56920763	0.7769081
G1SYB4$$Laminin subunit gamma-1 OS=Homo sapiens GN=LAMC1 PE=1 SV=3	28.1	0.60528028	0.56931154	0.7769081
G1TDK8$$Methylthioribose-1-phosphate isomerase OS=Oryctolagus cuniculus OX=9986 GN=MRI1 PE=3 SV=2	31.1	0.60322307	0.57058383	0.7780275
G1U460$$Epoxide hydrolase OS=Oryctolagus cuniculus OX=9986 GN=EPHX1 PE=3 SV=1	33.7	0.602590846	0.57097519	0.7780275
G1T0N5$$Uncharacterized protein OS=Oryctolagus cuniculus OX=9986 GN=LOC100345716 PE=3 SV=1	18.2	0.600716267	0.57213655	0.7788032
G1SM27$$Ribosomal protein L15 OS=Oryctolagus cuniculus OX=9986 PE=3 SV=1	26.766667	0.600309601	0.57238869	0.7788032
G1TM29$$Corticosteroid 11-beta-dehydrogenase isozyme 2 OS=Oryctolagus cuniculus OX=9986 GN=HSD11B2 PE=4 SV=1	32.766667	0.597624023	0.57405547	0.7799318
G1SM08$$Uncharacterized protein OS=Oryctolagus cuniculus OX=9986 GN=HSPA4L PE=3 SV=2	18.6	0.59754998	0.57410147	0.7799318
G1TJW3$$Peptidyl-prolyl cis-trans isomerase FKBP8 OS=Homo sapiens GN=FKBP8 PE=1 SV=2	17.5	0.596423429	0.57480157	0.7799318
G1THY5$$Uncharacterized protein OS=Oryctolagus cuniculus OX=9986 GN=PIGS PE=4 SV=1	33.114291	0.596070204	0.5750212	0.7799318
G1U949$$Uncharacterized protein OS=Oryctolagus cuniculus OX=9986 GN=VARS PE=3 SV=1	-20.6	-0.593767029	0.57645449	0.7799318
G1U7C6$$Glutathione S-transferase Mu 1 OS=Oryctolagus cuniculus OX=9986 PE=1 SV=2	-14.86667	-0.593595047	0.5765616	0.7799318

G1SMR7$$Uncharacterized protein OS=Oryctolagus cuniculus OX=9986 GN=CYP20A1 PE=4 SV=1	13.5	0.593575566	0.57657374	0.7799318
G1TM60$$Uncharacterized protein OS=Oryctolagus cuniculus OX=9986 GN=ACAA2 PE=3 SV=1	17.833333	0.59353348	0.57659995	0.7799318
G1TBS2$$Uncharacterized protein OS=Oryctolagus cuniculus OX=9986 GN=ALDH2 PE=3 SV=2	19.1	0.591766392	0.57770128	0.780849
G1T4X8$$Mannose-6-phosphate isomerase OS=Oryctolagus cuniculus OX=9986 GN=MPI PE=3 SV=2	19.4	0.589481768	0.57912706	0.7819955
G1SL16$$Uncharacterized protein OS=Oryctolagus cuniculus OX=9986 GN=PPP1R7 PE=4 SV=2	27.633333	0.588835075	0.57953103	0.7819955
G1TYN0$$Uncharacterized protein OS=Oryctolagus cuniculus OX=9986 PE=4 SV=1	-20.63333	-0.586590013	0.58093479	0.7819955
G1T5J9$$Uncharacterized protein OS=Oryctolagus cuniculus OX=9986 GN=ATP5PB PE=4 SV=2	17.4	0.585697808	0.58149323	0.7819955
G1U120$$Carbonic anhydrase 1 (Fragment) OS=Oryctolagus cuniculus OX=9986 GN=CA1 PE=2 SV=1	26.315681	0.585661704	0.58151584	0.7819955
G1TJG3$$Terpene cyclase/mutase family member OS=Oryctolagus cuniculus OX=9986 GN=LSS PE=3 SV=2	18.166667	0.584869634	0.58201188	0.7819955
G1T846$$Serine/threonine-protein phosphatase OS=Oryctolagus cuniculus OX=9986 GN=PPP1CC PE=3 SV=1	10.633333	0.584252634	0.58239847	0.7819955
G1U5L3$$Uncharacterized protein OS=Oryctolagus cuniculus OX=9986 GN=AKR7L PE=4 SV=1	19.9	0.584168211	0.58245138	0.7819955
G1T616$$40S ribosomal protein S21 OS=Oryctolagus cuniculus OX=9986 GN=RPS21 PE=1 SV=1	14.233333	0.584083142	0.58250469	0.7819955
G1ST12$$Uncharacterized protein OS=Oryctolagus cuniculus OX=9986 GN=PSIP1 PE=4 SV=1	7.9661707	0.583631269	0.58278795	0.7819955
G1T4Z0$$Uncharacterized protein OS=Oryctolagus cuniculus OX=9986 GN=NSDHL PE=3 SV=1	28.166667	0.581153762	0.58434243	0.7831741
G1TLD3$$Clusterin OS=Oryctolagus cuniculus OX=9986 GN=CLU PE=3 SV=2	26.7	0.580608665	0.58468478	0.7831741
G1TB98$$""NADH dehydrogenase [ubiquinone] flavoprotein 2, mitochondrial OS=Homo sapiens GN=NDUFV2 PE=1 SV=1""	22	0.580202805	0.58493976	0.7831741
G1SRG3$$""60 kDa heat shock protein, mitochondrial OS=Homo sapiens GN=HSPD1 PE=1 SV=2""	23.94246	0.577021085	0.58694098	0.7852836

G1TA15$$General transcription factor IIH subunit 4 OS=Oryctolagus cuniculus OX=9986 GN=VARS2 PE=3 SV=1	21.566667	0.575410789	0.58795538	0.7860708
O97529$$Aminopeptidase OS=Oryctolagus cuniculus OX=9986 GN=NPEPPS PE=3 SV=1	36.6	0.573921516	0.58889448	0.7867562
G1U9J9$$Amine oxidase OS=Oryctolagus cuniculus OX=9986 GN=MAOB PE=3 SV=1	29.366667	0.573239304	0.58932496	0.7867616
G1TH06$$Ethanolamine-phosphate cytidylyltransferase OS=Homo sapiens GN=PCYT2 PE=1 SV=1	19.4	0.571995948	0.59011002	0.78724
G1SKF7$$Ring finger protein 114 OS=Oryctolagus cuniculus OX=9986 GN=RNF114 PE=4 SV=1	12.2	0.565379115	0.59429836	0.7920714
G1T918$$Amidophosphoribosyltransferase OS=Oryctolagus cuniculus OX=9986 GN=PPAT PE=3 SV=1	-5.3	-0.564919116	0.59459019	0.7920714
G1T0W7$$Uncharacterized protein OS=Oryctolagus cuniculus OX=9986 GN=RDX PE=4 SV=2	7.1333333	0.563740632	0.59533821	0.7923491
G1SH63$$Very-long-chain enoyl-CoA reductase OS=Homo sapiens GN=TECR PE=1 SV=1	29.366667	0.563237826	0.59565753	0.7923491
P14461$$Uncharacterized protein OS=Oryctolagus cuniculus OX=9986 GN=DCPS PE=4 SV=1	6.4333333	0.560948405	0.59711275	0.7937125
G1U1C0$$Uncharacterized protein OS=Oryctolagus cuniculus OX=9986 GN=SUN2 PE=4 SV=2	13.933333	0.559229901	0.59820645	0.7945939
G1TJV3$$Dipeptidyl peptidase 3 OS=Oryctolagus cuniculus OX=9986 GN=DPP3 PE=3 SV=1	25.933333	0.55749811	0.5993098	0.7954868
G1SFW1$$ATP-citrate synthase OS=Oryctolagus cuniculus OX=9986 GN=ACLY PE=3 SV=2	9.5	0.554987262	0.60091162	0.7970395
G1T5D3$$Voltage-dependent anion-selective channel protein 3 OS=Oryctolagus cuniculus OX=9986 GN=VDAC3 PE=2 SV=1	26.591244	0.553268205	0.60200975	0.7977283
B7NZJ4$$Eukaryotic translation initiation factor 3 subunit L OS=Homo sapiens GN=EIF3L PE=1 SV=1	18.833333	0.552258032	0.6026556	0.7977283
G1SH00$$Uncharacterized protein OS=Oryctolagus cuniculus OX=9986 GN=LOC100347836 PE=4 SV=1	20.5	0.552144735	0.60272806	0.7977283
G1T8V6$$Uncharacterized protein OS=Oryctolagus cuniculus OX=9986 GN=ARHGAP18 PE=4 SV=2	22.366667	0.548037671	0.60535821	0.800635
G1SNM8$$Lipoma-preferred partner (Fragment) OS=Homo sapiens GN=LPP PE=1 SV=8	27.778571	0.546785939	0.60616114	0.8011227

G1T6C0$$Uncharacterized protein OS=Oryctolagus cuniculus OX=9986 GN=NQO2 PE=4 SV=1	-5.933333	-0.545820492	0.60678085	0.8013677
U3KPG6$$V-type proton ATPase catalytic subunit A OS=Homo sapiens GN=ATP6V1A PE=1 SV=2	30.121156	0.544100285	0.60788594	0.8014735
G1T7Y7$$Tubulin alpha chain OS=Homo sapiens GN=TUBA1C PE=1 SV=1	6.8333333	0.543879902	0.6080276	0.8014735
G1SCP0$$Sorting nexin OS=Oryctolagus cuniculus OX=9986 GN=SNX5 PE=3 SV=1	23.3	0.543667457	0.60816418	0.8014735
U3KN22$$Golgi-associated PDZ and coiled-coil motif-containing protein OS=Homo sapiens GN=GOPC PE=1 SV=1	9.4	0.541303541	0.60968509	0.8026877
G1T5Y2$$Spectrin beta chain OS=Homo sapiens GN=SPTBN1 PE=1 SV=1	27.41597	0.540297755	0.61033287	0.8026877
G1TZP0$$Uncharacterized protein OS=Oryctolagus cuniculus OX=9986 GN=MRPS22 PE=4 SV=1	25.366667	0.540008651	0.61051914	0.8026877
G1SL62$$Uncharacterized protein OS=Oryctolagus cuniculus OX=9986 PE=4 SV=1	-33.86667	-0.539513918	0.61083797	0.8026877
G1TP36$$Uncharacterized protein OS=Oryctolagus cuniculus OX=9986 GN=RACK1 PE=4 SV=1	-8.066667	-0.538858036	0.61126081	0.8026877
G1TUC2$$Uncharacterized protein OS=Oryctolagus cuniculus OX=9986 GN=TMEM126A PE=4 SV=1	14.866667	0.536354187	0.61287653	0.8038728
G1SVJ1$$Mth938 domain-containing protein OS=Homo sapiens GN=AAMDC PE=1 SV=1	27.437456	0.536109321	0.61303467	0.8038728
G1U4Y5$$V-type proton ATPase subunit D OS=Oryctolagus cuniculus OX=9986 GN=ATP6V1D PE=4 SV=1	-27.19213	-0.53471572	0.61393515	0.8044818
G1SSX2$$Uncharacterized protein OS=Oryctolagus cuniculus OX=9986 GN=HSPB1 PE=3 SV=1	26.945481	0.533933591	0.61444085	0.804573
G1SCY3$$Uncharacterized protein OS=Oryctolagus cuniculus OX=9986 GN=VAPA PE=4 SV=1	18.966667	0.532716127	0.61522851	0.805033
G1T5Y1$$60S acidic ribosomal protein P2 OS=Homo sapiens GN=RPLP2 PE=1 SV=1	22.3	0.529719475	0.61716967	0.8070007
G1SP30$$Uncharacterized protein OS=Oryctolagus cuniculus OX=9986 GN=DLST PE=4 SV=1	17.066667	0.528764229	0.61778919	0.8072387
G1TK30$$Synaptic vesicle membrane protein VAT-1 homolog OS=Homo sapiens GN=VAT1 PE=1 SV=2	-10.3	-0.52782202	0.6184006	0.8073666
G1U5B3$$Uncharacterized protein OS=Oryctolagus cuniculus OX=9986 GN=APPL1 PE=4 SV=1	9.4666667	0.526048338	0.61955249	0.8073666

G1SYI2$$Uncharacterized protein OS=Oryctolagus cuniculus OX=9986 GN=ERMP1 PE=4 SV=1	5.5	0.525998407	0.61958493	0.8073666
G1SF32$$Transaldolase OS=Homo sapiens GN=TALDO1 PE=1 SV=2	20.6	0.525557912	0.6198712	0.8073666
G1SFE6$$Mitochondrial Rho GTPase OS=Oryctolagus cuniculus OX=9986 GN=RHOT1 PE=3 SV=2	16.333333	0.52524433	0.62007504	0.8073666
G1TDN3$$Carbonic anhydrase 2 OS=Oryctolagus cuniculus OX=9986 GN=CA2 PE=1 SV=3	17.066667	0.523408468	0.62126915	0.808178
G1T096$$UMP-CMP kinase OS=Oryctolagus cuniculus OX=9986 GN=CMPK1 PE=3 SV=1	-5.233333	-0.522939649	0.62157429	0.808178
G1SU17$$Uncharacterized protein OS=Oryctolagus cuniculus OX=9986 PE=4 SV=2	9.5	0.52016308	0.62338323	0.8089222
G1T087$$V-type proton ATPase subunit F OS=Oryctolagus cuniculus OX=9986 GN=ATP6V1F PE=3 SV=1	27.366667	0.519653356	0.62371564	0.8089222
U3KM53$$Uncharacterized protein OS=Oryctolagus cuniculus OX=9986 GN=DPT PE=4 SV=1	-20.03333	-0.519615246	0.62374049	0.8089222
G1T8P1$$Pyrroline-5-carboxylate reductase OS=Homo sapiens GN=PYCR3 PE=1 SV=1	26.433333	0.519370056	0.62390043	0.8089222
G1TZQ6$$Uncharacterized protein OS=Oryctolagus cuniculus OX=9986 GN=BAG3 PE=4 SV=2	34.566667	0.518542803	0.6244402	0.8090535
G1TCW5$$Uncharacterized protein OS=Oryctolagus cuniculus OX=9986 GN=HDLBP PE=4 SV=1	-9.666667	-0.514091134	0.62734937	0.8120383
G1TQC7$$Apolipoprotein C-IV OS=Oryctolagus cuniculus OX=9986 GN=APOC4 PE=1 SV=1	27.539705	0.513387722	0.62780974	0.8120383
G1SFR8$$Uncharacterized protein OS=Oryctolagus cuniculus OX=9986 GN=MARS2 PE=3 SV=1	11.033333	0.512998914	0.62806429	0.8120383
G1TB49$$RNA-binding protein 47 OS=Homo sapiens GN=RBM47 PE=1 SV=2	19.266667	0.511651265	0.62894702	0.8126101
G1SKS8$$60S ribosomal protein L23a OS=Homo sapiens GN=RPL23A PE=1 SV=1	22.166667	0.509462022	0.63038247	0.8138948
G1TDN1$$Uncharacterized protein OS=Oryctolagus cuniculus OX=9986 GN=MSRA PE=3 SV=1	21.633333	0.507229407	0.63184823	0.8152168
G1SN67$$Phosphatidylinositol transfer protein alpha isoform OS=Oryctolagus cuniculus OX=9986 GN=PITPNA PE=4 SV=1	-5.466667	-0.502545904	0.63492914	0.8186193
G1U9C1$$Acylamino-acid-releasing enzyme OS=Oryctolagus cuniculus OX=9986 GN=APEH PE=4 SV=1	13.8	0.50110518	0.63587853	0.8192709

G1TWQ3$$""Proteasome 26S subunit, ATPase 4 OS=Oryctolagus cuniculus OX=9986 GN=PSMC4 PE=3 SV=2""	8.9	0.497903843	0.63799089	0.8214188
G1SJN4$$Uncharacterized protein OS=Oryctolagus cuniculus OX=9986 PE=3 SV=1	19.533333	0.496397823	0.63898593	0.8220796
G1SUC8$$Uncharacterized protein OS=Oryctolagus cuniculus OX=9986 GN=PFDN2 PE=4 SV=1	15.2	0.495778745	0.63939521	0.8220796
G1TTU6$$Uncharacterized protein OS=Oryctolagus cuniculus OX=9986 GN=LOC100357845 PE=4 SV=1	15.033333	0.493521422	0.64088875	0.8230974
G1SP54$$Uncharacterized protein OS=Oryctolagus cuniculus OX=9986 GN=GCN1 PE=4 SV=1	-12.66667	-0.493233963	0.64107908	0.8230974
G1U0U5$$Lambda-crystallin OS=Oryctolagus cuniculus OX=9986 GN=CRYL1 PE=4 SV=2	26.133333	0.492097792	0.64183165	0.8234905
G1SNY0$$40S ribosomal protein S9 OS=Homo sapiens GN=RPS9 PE=1 SV=3	22.133333	0.49087539	0.64264187	0.8237679
G1U797$$Uncharacterized protein OS=Oryctolagus cuniculus OX=9986 GN=KRAS PE=4 SV=1	21.679343	0.490424569	0.64294082	0.8237679
G1SMS3$$Reactive oxygen species modulator 1 OS=Homo sapiens GN=ROMO1 PE=1 SV=1	-23.56667	-0.488667662	0.64410658	0.824232
G1TDJ2$$Fragile X mental retardation syndrome-related protein 2 OS=Homo sapiens GN=FXR2 PE=1 SV=2	30.270956	0.488493088	0.64422247	0.824232
G1T336$$Uncharacterized protein OS=Oryctolagus cuniculus OX=9986 PE=4 SV=2	11.266667	0.487679336	0.64476286	0.824232
G1SI19$$Uncharacterized protein OS=Oryctolagus cuniculus OX=9986 GN=CBR4 PE=4 SV=1	36.366667	0.487186898	0.64508999	0.824232
G1T125$$Hydroxymethylbilane synthase OS=Oryctolagus cuniculus OX=9986 GN=HMBS PE=3 SV=1	26.233333	0.486097282	0.64581414	0.8245711
G1TA11$$Uncharacterized protein OS=Oryctolagus cuniculus OX=9986 GN=PGRMC2 PE=3 SV=2	17.633333	0.48511785	0.64646543	0.8245711
G1SQS9$$""Isocitrate dehydrogenase [NAD] subunit, mitochondrial OS=Oryctolagus cuniculus OX=9986 GN=IDH3A PE=3 SV=1""	29.233333	0.484771021	0.64669615	0.8245711
G1SW65$$Uncharacterized protein OS=Oryctolagus cuniculus OX=9986 GN=CTNNB1 PE=4 SV=1	20.166667	0.483376101	0.64762452	0.8251846

G1U612$$Eukaryotic translation initiation factor 3 subunit E OS=Oryctolagus cuniculus OX=9986 GN=EIF3E PE=1 SV=2	-35.23333	-0.48204545	0.64851077	0.8257435
Q9XS70$$Uncharacterized protein OS=Oryctolagus cuniculus OX=9986 GN=DMAC2L PE=4 SV=1	-14.1	-0.480077915	0.6498224	0.8257859
G1T3Y0$$Dynamin-2 OS=Homo sapiens GN=DNM2 PE=1 SV=2	34.233333	0.478415507	0.65093171	0.8257859
G1TDC3$$Tyrosine-protein kinase OS=Oryctolagus cuniculus OX=9986 GN=SRC PE=3 SV=1	21.733333	0.478153253	0.6511068	0.8257859
Q6SQH4$$Uncharacterized protein OS=Oryctolagus cuniculus OX=9986 GN=PRPF19 PE=4 SV=1	-29.73071	-0.477034402	0.65185408	0.8257859
G1TT64$$Uncharacterized protein OS=Oryctolagus cuniculus OX=9986 GN=FABP5 PE=3 SV=2	14.666667	0.476974699	0.65189397	0.8257859
G1TA04$$AP complex subunit beta OS=Oryctolagus cuniculus OX=9986 GN=AP2B1 PE=3 SV=1	-11.06667	-0.476801998	0.65200936	0.8257859
G1TMM0$$Polypeptide N-acetylgalactosaminyltransferase OS=Oryctolagus cuniculus OX=9986 GN=GALNT2 PE=3 SV=1	25.733333	0.476682504	0.6520892	0.8257859
G1SZB1$$""Isovaleryl-CoA dehydrogenase, mitochondrial OS=Homo sapiens GN=IVD PE=1 SV=1""	20.533679	0.476629399	0.65212469	0.8257859
G1SLU3$$Probable ATP-dependent RNA helicase DDX5 OS=Homo sapiens GN=DDX5 PE=1 SV=1	-19.96667	-0.474388742	0.6536229	0.8271154
G1T9I3$$Apolipoprotein B OS=Oryctolagus cuniculus OX=9986 GN=APOB PE=4 SV=1	16.533333	0.472456301	0.65491647	0.8281843
G1SRX2$$Myosin light polypeptide 6 OS=Homo sapiens GN=MYL6 PE=1 SV=1	18.3	0.469449671	0.65693178	0.8301638
G1SW24$$Histone-binding protein RBBP4 OS=Homo sapiens GN=RBBP4 PE=1 SV=3	8.4666667	0.468483185	0.65758029	0.8303829
G1TT27$$Uncharacterized protein OS=Oryctolagus cuniculus OX=9986 GN=MSN PE=4 SV=1	8.8	0.467194517	0.65844551	0.8303829
P62493$$Uncharacterized protein OS=Oryctolagus cuniculus OX=9986 GN=IPO7 PE=4 SV=1	-14.86667	-0.466804437	0.65870753	0.8303829
U3KMP1$$V-type proton ATPase subunit C OS=Oryctolagus cuniculus OX=9986 GN=ATP6V1C1 PE=3 SV=2	-3.666667	-0.466509779	0.65890549	0.8303829
G1SY36$$GDP-L-fucose synthase OS=Homo sapiens GN=TSTA3 PE=1 SV=1	4.4333333	0.46520159	0.65978474	0.8309234

G1TF32$$Phospholipid transfer protein OS=Oryctolagus cuniculus OX=9986 GN=PLTP PE=4 SV=1	15.4	0.464212582	0.66044986	0.8309906
G1SZN0$$Uncharacterized protein OS=Oryctolagus cuniculus OX=9986 PE=4 SV=1	20.1	0.463783003	0.66073887	0.8309906
G1SIY9$$Uncharacterized protein OS=Oryctolagus cuniculus OX=9986 GN=PSMC1 PE=3 SV=1	5.6	0.461399023	0.66234394	0.8318094
G1TX70$$Proteasome subunit beta OS=Oryctolagus cuniculus OX=9986 GN=PSMB2 PE=3 SV=2	16.866667	0.460876379	0.66269609	0.8318094
G1SGI8$$ATP-binding cassette sub-family F member 1 OS=Homo sapiens GN=ABCF1 PE=1 SV=2	4	0.460807547	0.66274247	0.8318094
G1T277$$60S ribosomal protein L18a OS=Homo sapiens GN=RPL18A PE=1 SV=2	-20	-0.458627701	0.66421232	0.8327515
G1T0K1$$Parathymosin OS=Homo sapiens GN=PTMS PE=1 SV=1	17	0.458355809	0.66439577	0.8327515
G1SS33$$Myosin-11 OS=Oryctolagus cuniculus OX=9986 GN=MYH11 PE=2 SV=2	6.9333333	0.45763707	0.66488085	0.8327937
G1SCT0$$Calumenin OS=Oryctolagus cuniculus OX=9986 GN=CALU PE=4 SV=1	14	0.455892345	0.66605911	0.8337036
G1SMM5$$Heat shock protein HSP 90-alpha OS=Oryctolagus cuniculus OX=9986 GN=HSP90AA1 PE=1 SV=2	10.733333	0.452097991	0.66862523	0.8363482
G1SCT1$$Eukaryotic translation initiation factor 3 subunit D OS=Oryctolagus cuniculus OX=9986 GN=EIF3D PE=3 SV=1	26.766667	0.450657547	0.66960073	0.8363606
G1U8C4$$Uncharacterized protein OS=Oryctolagus cuniculus OX=9986 GN=ACSS3 PE=4 SV=2	34.833333	0.450602333	0.66963813	0.8363606
G1TLB2$$Uncharacterized protein OS=Oryctolagus cuniculus OX=9986 PE=4 SV=2	25.98037	0.449632278	0.67029549	0.8363606
G1TZN7$$Uncharacterized protein OS=Oryctolagus cuniculus OX=9986 GN=UBA2 PE=4 SV=2	13.3	0.449406648	0.67044844	0.8363606
G1SY84$$ADP-ribosylhydrolase like 2 OS=Oryctolagus cuniculus OX=9986 GN=ADPRHL2 PE=4 SV=1	3.5	0.448122927	0.67131896	0.8367937
G1U153$$""Acetyl-CoA acetyltransferase, mitochondrial OS=Homo sapiens GN=ACAT1 PE=1 SV=1""	9.5	0.44755728	0.67170272	0.8367937
P07952$$Uncharacterized protein OS=Oryctolagus cuniculus OX=9986 GN=MAP1B PE=4 SV=2	-4.033333	-0.443462259	0.67448428	0.8388634
G1T6B3$$Cortactin OS=Oryctolagus cuniculus OX=9986 GN=CTTN PE=4 SV=2	12.4	0.443067189	0.67475294	0.8388634

G1SYL8$$Uncharacterized protein OS=Oryctolagus cuniculus OX=9986 GN=ACACA PE=4 SV=2	19.166667	0.442785098	0.6749448	0.8388634
G1SKS0$$Acyl-CoA dehydrogenase family member 8 OS=Oryctolagus cuniculus OX=9986 GN=ACAD8 PE=3 SV=1	16.033333	0.442121982	0.67539592	0.8388634
Q6TYA7$$Uncharacterized protein OS=Oryctolagus cuniculus OX=9986 GN=LOC100354435 PE=3 SV=1	18.6	0.441750564	0.67564866	0.8388634
G1U8F0$$Uncharacterized protein OS=Oryctolagus cuniculus OX=9986 GN=KHDRBS1 PE=4 SV=1	14	0.441099178	0.67609204	0.8388634
G1SLQ4$$Acyl-CoA-binding protein OS=Oryctolagus cuniculus OX=9986 GN=DBI PE=3 SV=3	-8.2	-0.439591592	0.67711875	0.8391525
G1SF70$$Uncharacterized protein OS=Oryctolagus cuniculus OX=9986 GN=DHRS7 PE=3 SV=1	20.946338	0.438302884	0.67799701	0.8391525
G1T974$$Isocitrate dehydrogenase [NADP] OS=Oryctolagus cuniculus OX=9986 GN=IDH2 PE=3 SV=1	25.033333	0.438149317	0.67810171	0.8391525
G1SL38$$Uncharacterized protein OS=Oryctolagus cuniculus OX=9986 GN=MMAB PE=4 SV=1	22.533333	0.43808675	0.67814437	0.8391525
G1TSJ0$$Uncharacterized protein OS=Oryctolagus cuniculus OX=9986 GN=DCTN3 PE=4 SV=1	-12.5	-0.437378942	0.67862704	0.8391869
G1SU71$$Uncharacterized protein OS=Oryctolagus cuniculus OX=9986 GN=LCN2 PE=3 SV=1	8.2666667	0.436404613	0.67929173	0.8394462
G1SZ91$$Uncharacterized protein OS=Oryctolagus cuniculus OX=9986 GN=ABHD6 PE=4 SV=2	-28.93333	-0.435355966	0.68000749	0.8397683
G1TMS5$$Uncharacterized protein OS=Oryctolagus cuniculus OX=9986 GN=CAPRIN1 PE=4 SV=1	23.366667	0.431432057	0.68268909	0.842516
G1TW98$$CDP-diacylglycerol--inositol 3-phosphatidyltransferase OS=Homo sapiens GN=CDIPT PE=1 SV=1	19.233333	0.428997512	0.68435547	0.8440079
G1SEJ4$$Alpha-crystallin B chain OS=Oryctolagus cuniculus OX=9986 GN=CRYAB PE=3 SV=1	-4.166667	-0.424864025	0.68718929	0.8469367
G1SN16$$Uncharacterized protein OS=Oryctolagus cuniculus OX=9986 GN=SEC62 PE=4 SV=1	13.466667	0.422967691	0.68849128	0.8479749
G1SJQ2$$Heat shock cognate 71 kDa protein (Fragment) OS=Homo sapiens GN=HSPA8 PE=1 SV=8	11.266667	0.420094471	0.69046627	0.8498401
G1SH05$$""Splicing factor, proline- and glutamine-rich OS=Homo sapiens GN=SFPQ PE=1 SV=2""	8.9	0.416611138	0.69286431	0.8513535

U3KLU4$$Uncharacterized protein OS=Oryctolagus cuniculus OX=9986 GN=YWHAB PE=3 SV=1	19.233333	0.416334886	0.69305466	0.8513535
G1TDN4$$Uncharacterized protein OS=Oryctolagus cuniculus OX=9986 GN=THBS1 PE=4 SV=2	13.333333	0.415897398	0.69335616	0.8513535
U3KM96$$Uncharacterized protein OS=Oryctolagus cuniculus OX=9986 GN=LOC100349893 PE=3 SV=1	-8.466667	-0.415628375	0.69354159	0.8513535
G1U6N2$$Uncharacterized protein OS=Oryctolagus cuniculus OX=9986 GN=NONO PE=4 SV=1	7.1333333	0.413859399	0.69476151	0.8518329
G1T235$$Uncharacterized protein OS=Oryctolagus cuniculus OX=9986 GN=ACAT1 PE=3 SV=2	26	0.413723114	0.69485554	0.8518329
P11909$$Uncharacterized protein OS=Oryctolagus cuniculus OX=9986 GN=ERLIN2 PE=4 SV=1	20.668526	0.411020962	0.69672109	0.8528628
G1U4G9$$Histone H4 OS=Oryctolagus cuniculus OX=9986 GN=HIST4H4 PE=3 SV=1	5.4666667	0.410536305	0.69705595	0.8528628
G1SV24$$Delta-aminolevulinic acid dehydratase OS=Oryctolagus cuniculus OX=9986 GN=ALAD PE=3 SV=2	-17	-0.410455243	0.69711197	0.8528628
G1SJZ9$$Uncharacterized protein OS=Oryctolagus cuniculus OX=9986 GN=NAP1L1 PE=3 SV=1	9.3	0.409829156	0.69754467	0.8528628
G1TG89$$Fatty acid synthase OS=Homo sapiens GN=FASN PE=1 SV=3	-9.466667	-0.408942929	0.69815738	0.8530466
G1U7S8$$Uncharacterized protein OS=Oryctolagus cuniculus OX=9986 GN=ENO2 PE=3 SV=1	-16.91589	-0.405906071	0.7002589	0.8550481
G1SFG6$$Uncharacterized protein OS=Oryctolagus cuniculus OX=9986 GN=UBE2M PE=3 SV=1	9.1333333	0.40457402	0.70118163	0.8556085
G1T926$$Rab GTPase-binding effector protein 2 OS=Homo sapiens GN=RABEP2 PE=1 SV=2	18.4	0.402071629	0.7029166	0.856148
G1SXA5$$Uroporphyrinogen decarboxylase OS=Oryctolagus cuniculus OX=9986 GN=UROD PE=3 SV=1	10.933333	0.401690925	0.70318073	0.856148
G1T060$$Uncharacterized protein OS=Oryctolagus cuniculus OX=9986 GN=YWHAE PE=3 SV=1	7.6666667	0.40123534	0.70349687	0.856148
G1TEG8$$Uncharacterized protein OS=Oryctolagus cuniculus OX=9986 GN=NDUFS4 PE=4 SV=1	19.433333	0.400924695	0.70371248	0.856148
G1SJR4$$Neuroblast differentiation-associated protein AHNAK OS=Homo sapiens GN=AHNAK PE=1 SV=2	-3.3	-0.400386877	0.70408582	0.856148
G1TVG8$$Uncharacterized protein OS=Oryctolagus cuniculus OX=9986 GN=HNRNPL PE=4 SV=1	15.333333	0.399922944	0.70440795	0.856148

Q9TT13$$Uncharacterized protein OS=Oryctolagus cuniculus OX=9986 GN=C4BPA PE=4 SV=2	3.6	0.397808464	0.70587699	0.8569091
U3KNL6$$Uncharacterized protein OS=Oryctolagus cuniculus OX=9986 GN=HSPG2 PE=4 SV=2	-4.833333	-0.3976847	0.70596302	0.8569091
G1T7W2$$Uncharacterized protein OS=Oryctolagus cuniculus OX=9986 GN=ANKFY1 PE=4 SV=2	11.766667	0.396670701	0.70666805	0.8572009
G1SZD2$$Uncharacterized protein OS=Oryctolagus cuniculus OX=9986 GN=TMEM245 PE=4 SV=1	14.966667	0.395544182	0.70745169	0.8575876
P62160$$Actin-related protein 2/3 complex subunit 5 OS=Homo sapiens GN=ARPC5 PE=1 SV=3	-10.13333	-0.393616852	0.70879333	0.858043
G1SK33$$Uncharacterized protein OS=Oryctolagus cuniculus OX=9986 GN=LOC100354714 PE=1 SV=1	-10.5	-0.392791872	0.70936797	0.858043
G1STZ4$$Uncharacterized protein OS=Oryctolagus cuniculus OX=9986 GN=ALDH1L1 PE=4 SV=2	21.3	0.392275531	0.70972773	0.858043
G1T508$$MICOS complex subunit MIC10 OS=Oryctolagus cuniculus OX=9986 GN=NBL1 PE=3 SV=2	10.633333	0.391680841	0.71014219	0.858043
G1T127$$Uncharacterized protein OS=Oryctolagus cuniculus OX=9986 GN=ENO1 PE=3 SV=1	-20.89681	-0.391639666	0.71017089	0.858043
G1U7U3$$Uncharacterized protein OS=Oryctolagus cuniculus OX=9986 PE=4 SV=1	3.4666667	0.390998767	0.71061769	0.858043
G1TMF1$$Synaptosomal-associated protein 25 OS=Homo sapiens GN=SNAP25 PE=1 SV=1	19.940681	0.38770352	0.71291698	0.8593232
G1SR29$$Serrate RNA effector molecule homolog OS=Homo sapiens GN=SRRT PE=1 SV=1	8.2	0.386382525	0.71383966	0.8593232
G1T0H0$$Uncharacterized protein OS=Oryctolagus cuniculus OX=9986 GN=SCCPDH PE=4 SV=1	-5.033333	-0.386272274	0.7139167	0.8593232
G1SY27$$Uncharacterized protein OS=Oryctolagus cuniculus OX=9986 GN=EEF1B2 PE=3 SV=1	28.933333	0.385942274	0.71414729	0.8593232
G1TII2$$Transforming acidic coiled-coil-containing protein 1 OS=Homo sapiens GN=TACC1 PE=1 SV=2	-4.566667	-0.385526409	0.71443793	0.8593232
G1SIT5$$Uncharacterized protein OS=Oryctolagus cuniculus OX=9986 GN=SLC25A11 PE=3 SV=1	12.933333	0.38547699	0.71447248	0.8593232
G1T1L4$$Gamma-synuclein OS=Oryctolagus cuniculus OX=9986 GN=SNCG PE=3 SV=2	19.214553	0.384122239	0.71541968	0.8599018
U3KNQ7$$Tetranectin OS=Homo sapiens GN=CLEC3B PE=1 SV=3	25	0.381927451	0.71695543	0.8611867

G1SDS3$$""Isocitrate dehydrogenase [NAD] subunit, mitochondrial OS=Oryctolagus cuniculus OX=9986 GN=IDH3B PE=3 SV=1""	12.133333	0.37958435	0.7185966	0.8622192
G1T2B8$$Glutathione S-transferase OS=Oryctolagus cuniculus OX=9986 GN=LOC100357917 PE=3 SV=1	18.390972	0.379007344	0.719001	0.8622192
G1STW7$$Uncharacterized protein OS=Oryctolagus cuniculus OX=9986 GN=PAIP1 PE=4 SV=2	13.8	0.378699235	0.71921699	0.8622192
G1SVB0$$Eukaryotic initiation factor 4A-III OS=Homo sapiens GN=EIF4A3 PE=1 SV=4	-7.3	-0.377852686	0.71981058	0.8622322
G1SJW7$$Uncharacterized protein OS=Oryctolagus cuniculus OX=9986 GN=COX5B PE=4 SV=1	17.783501	0.377350984	0.72016247	0.8622322
G1SMJ5$$Uncharacterized protein OS=Oryctolagus cuniculus OX=9986 GN=UBL5 PE=4 SV=1	15.633333	0.376342103	0.72087032	0.8625199
G1SLM0$$Uncharacterized protein OS=Oryctolagus cuniculus OX=9986 GN=LMNB1 PE=3 SV=1	4.7333333	0.373435349	0.72291149	0.8644016
G1T8R3$$Uncharacterized protein OS=Oryctolagus cuniculus OX=9986 GN=SEPT6 PE=3 SV=2	16.433333	0.371005321	0.72461986	0.8647132
G1TM55$$Uncharacterized protein OS=Oryctolagus cuniculus OX=9986 GN=RPS10 PE=1 SV=2	30	0.370588107	0.72491335	0.8647132
Q28685$$Uncharacterized protein OS=Oryctolagus cuniculus OX=9986 GN=ENTPD1 PE=3 SV=2	-6.966667	-0.370508279	0.72496951	0.8647132
P00919$$Uncharacterized protein OS=Oryctolagus cuniculus OX=9986 GN=PTGFRN PE=4 SV=1	-22.36667	-0.370398481	0.72504676	0.8647132
G1TIB4$$Uncharacterized protein OS=Oryctolagus cuniculus OX=9986 GN=AFG3L2 PE=4 SV=1	7.6	0.367688194	0.72695475	0.8653134
G1SQ22$$Uncharacterized protein OS=Oryctolagus cuniculus OX=9986 GN=MUT PE=4 SV=2	12.6	0.367143789	0.72733827	0.8653134
G1SY02$$Uncharacterized protein OS=Oryctolagus cuniculus OX=9986 GN=GCLM PE=4 SV=2	14.933333	0.366975019	0.72745718	0.8653134
G1SNQ9$$Coatomer subunit alpha OS=Oryctolagus cuniculus OX=9986 GN=COPA PE=4 SV=2	-3.633333	-0.3660901	0.72808081	0.8653134
G1SJ41$$Uncharacterized protein OS=Oryctolagus cuniculus OX=9986 GN=EIF4B PE=4 SV=2	22.530435	0.365434438	0.72854303	0.8653134
G1SHK6$$Uncharacterized protein OS=Oryctolagus cuniculus OX=9986 GN=TMOD3 PE=4 SV=1	16.7	0.36517073	0.72872897	0.8653134
G1TRG8$$Glutathione peroxidase 1 OS=Oryctolagus cuniculus OX=9986 GN=GPX1 PE=2 SV=2	22.646169	0.365023063	0.7288331	0.8653134

G1SFF7$$Stathmin OS=Oryctolagus cuniculus OX=9986 GN=STMN2 PE=3 SV=2	-3.033333	-0.363170002	0.73014035	0.866308
G1SR77$$T-complex protein 1 subunit eta OS=Oryctolagus cuniculus OX=9986 GN=CCT7 PE=3 SV=2	-16.23333	-0.361063604	0.73162756	0.8675147
G1TTJ4$$Histone H3 OS=Homo sapiens GN=HIST2H3PS2 PE=1 SV=1	20.566667	0.357086773	0.73443894	0.8699507
G1TR31$$Plasminogen OS=Homo sapiens GN=PLG PE=1 SV=2	9.8666667	0.356823768	0.73462503	0.8699507
G1SH49$$Endophilin-A2 OS=Homo sapiens GN=SH3GL1 PE=1 SV=1	16.633333	0.353298049	0.73712163	0.8708136
G1T782$$Uncharacterized protein OS=Oryctolagus cuniculus OX=9986 PE=3 SV=1	25.633333	0.352533499	0.7376635	0.8708136
G1TQG1$$Elongation factor 2 OS=Homo sapiens GN=EEF2 PE=1 SV=4	-5.766667	-0.352178505	0.73791515	0.8708136
G1SSR8$$Apolipoprotein C-I OS=Oryctolagus cuniculus OX=9986 GN=apoCI PE=2 SV=1	19.733333	0.351839962	0.73815518	0.8708136
G1T239$$Uncharacterized protein OS=Oryctolagus cuniculus OX=9986 GN=UBR4 PE=4 SV=1	17.166667	0.351830162	0.73816212	0.8708136
G1SS73$$Apolipoprotein D OS=Oryctolagus cuniculus OX=9986 GN=APOD PE=2 SV=1	4.6	0.351796991	0.73818564	0.8708136
G1TPY7$$Uncharacterized protein OS=Oryctolagus cuniculus OX=9986 GN=NCL PE=4 SV=1	-4.933333	-0.3496258	0.73972582	0.8709489
G1TW43$$Acyl-protein thioesterase 1 OS=Oryctolagus cuniculus OX=9986 GN=LYPLA1 PE=4 SV=1	24.433333	0.349411184	0.73987814	0.8709489
U3KLY7$$Lysozyme C OS=Oryctolagus cuniculus OX=9986 GN=LYZ PE=1 SV=1	3.6666667	0.349290433	0.73996384	0.8709489
G1TX53$$Uncharacterized protein OS=Oryctolagus cuniculus OX=9986 GN=SELENBP1 PE=4 SV=1	12.466667	0.347006744	0.74158551	0.8709489
G1SWT9$$Protein transport protein SEC23 OS=Oryctolagus cuniculus OX=9986 GN=SEC23B PE=3 SV=1	17.733333	0.347000742	0.74158977	0.8709489
G1SV28$$Uncharacterized protein OS=Oryctolagus cuniculus OX=9986 GN=TTC19 PE=4 SV=1	-19.83333	-0.3467036	0.74180088	0.8709489
G1TAH7$$40S ribosomal protein S26 OS=Oryctolagus cuniculus OX=9986 GN=LOC100346996 PE=1 SV=1	11.633333	0.346368907	0.7420387	0.8709489
G1SMZ5$$Uncharacterized protein OS=Oryctolagus cuniculus OX=9986 GN=APOC3 PE=4 SV=1	9.9333333	0.345666906	0.74253763	0.8709489

G1T7M3$$Septin-9 OS=Homo sapiens GN=SEPT9 PE=1 SV=2	23.180476	0.345651082	0.74254887	0.8709489
G1SD02$$Uncharacterized protein OS=Oryctolagus cuniculus OX=9986 GN=BZW1 PE=4 SV=1	15.6	0.343815161	0.74385436	0.8715509
G1TD24$$Far upstream element-binding protein 3 OS=Homo sapiens GN=FUBP3 PE=1 SV=2	14.798301	0.34360072	0.74400691	0.8715509
G1TKY2$$Syntaxin binding protein 2 OS=Oryctolagus cuniculus OX=9986 GN=STXBP2 PE=3 SV=1	13.833333	0.340643994	0.74611156	0.8732948
G1TBS1$$Uncharacterized protein OS=Oryctolagus cuniculus OX=9986 GN=HNRNPUL1 PE=4 SV=1	14.833333	0.340179726	0.74644226	0.8732948
G1U2A1$$Uncharacterized protein OS=Oryctolagus cuniculus OX=9986 GN=NPC1 PE=4 SV=1	16.833333	0.339022191	0.74726704	0.8737058
G1SHG0$$Uncharacterized protein OS=Oryctolagus cuniculus OX=9986 GN=ECHDC2 PE=3 SV=1	7.3333333	0.336274328	0.74922647	0.8754419
G1SSK9$$KN motif and ankyrin repeat domain-containing protein 2 OS=Homo sapiens GN=KANK2 PE=1 SV=1	19.766667	0.335529192	0.74975817	0.8755087
G1TKL0$$Uncharacterized protein OS=Oryctolagus cuniculus OX=9986 PE=3 SV=1	9.5333333	0.334542673	0.75046234	0.8757767
P12822$$Uncharacterized protein OS=Oryctolagus cuniculus OX=9986 GN=TNC PE=4 SV=2	15.6	0.331630294	0.75254277	0.8771843
G1T704$$Tropomyosin alpha-1 chain OS=Homo sapiens GN=TPM1 PE=1 SV=1	22.066667	0.331523067	0.75261941	0.8771843
G1TBY1$$Uncharacterized protein OS=Oryctolagus cuniculus OX=9986 GN=HNRNPAB PE=4 SV=1	4.1666667	0.330074454	0.75365513	0.8778369
G1TCR3$$Annexin OS=Oryctolagus cuniculus OX=9986 GN=ANXA7 PE=3 SV=1	15.184236	0.328285354	0.75493507	0.878773
G1SK22$$Protein S100 OS=Oryctolagus cuniculus OX=9986 GN=S100A8 PE=3 SV=1	3.9666667	0.326860492	0.75595506	0.8788757
G1TE43$$Uncharacterized protein OS=Oryctolagus cuniculus OX=9986 GN=MCCC1 PE=4 SV=1	8.4333333	0.326831255	0.755976	0.8788757
U3KNB6$$Uncharacterized protein OS=Oryctolagus cuniculus OX=9986 GN=HNRNPD PE=4 SV=1	6.1333333	0.324605276	0.75757059	0.8799525
G1STQ7$$Uncharacterized protein OS=Oryctolagus cuniculus OX=9986 GN=AGFG2 PE=4 SV=2	17.633333	0.32420696	0.75785607	0.8799525
G1TT67$$Uncharacterized protein OS=Oryctolagus cuniculus OX=9986 GN=RPL32 PE=4 SV=1	-20.86667	-0.322247246	0.75926124	0.8806014

G1SS70$$Uncharacterized protein OS=Oryctolagus cuniculus OX=9986 GN=VCAN PE=4 SV=1	17.666667	0.322096255	0.75936954	0.8806014
G1TDQ5$$Uncharacterized protein OS=Oryctolagus cuniculus OX=9986 GN=ECHS1 PE=3 SV=1	7.4666667	0.319235215	0.76142295	0.882105
G1U2E3$$Superoxide dismutase OS=Homo sapiens GN=SOD2 PE=1 SV=1	19.6	0.318957559	0.76162235	0.882105
G1SW67$$Rho-associated protein kinase 1 OS=Oryctolagus cuniculus OX=9986 GN=ROCK1 PE=1 SV=1	-7.233333	-0.317212352	0.76287611	0.8828063
G1SP11$$""Peroxiredoxin-5, mitochondrial OS=Homo sapiens GN=PRDX5 PE=1 SV=4""	18.756273	0.316782817	0.76318481	0.8828063
G1SUZ7$$Ubiquitin-fold modifier 1 OS=Oryctolagus cuniculus OX=9986 PE=3 SV=1	-10.3	-0.315202486	0.764321	0.8833137
G1TEE4$$Nucleophosmin OS=Homo sapiens GN=NPM1 PE=1 SV=2	16.981419	0.314840951	0.76458102	0.8833137
G1SD43$$Uncharacterized protein OS=Oryctolagus cuniculus OX=9986 GN=LOC100353846 PE=4 SV=2	-13.5	-0.31364959	0.76543811	0.8837505
G1SP48$$Coactosin like F-actin binding protein 1 OS=Oryctolagus cuniculus OX=9986 GN=COTL1 PE=4 SV=2	12.9	0.308167086	0.76938709	0.8877543
G1TEH2$$NHP2-like protein 1 OS=Homo sapiens GN=SNU13 PE=1 SV=1	-9.433333	-0.307327053	0.76999285	0.887898
G1SR27$$Uncharacterized protein OS=Oryctolagus cuniculus OX=9986 GN=SERPINA3 PE=3 SV=2	17.133333	0.306009251	0.77094349	0.8881978
G1TCP3$$Peptidyl-prolyl cis-trans isomerase FKBP1A OS=Homo sapiens GN=FKBP1A PE=1 SV=2	17.5	0.305632112	0.77121564	0.8881978
G1T4T5$$Uncharacterized protein OS=Oryctolagus cuniculus OX=9986 GN=RPL27A PE=1 SV=1	12.766667	0.301384741	0.77428307	0.890491
Q9TTC6$$Collagen alpha-1(XII) chain OS=Oryctolagus cuniculus OX=9986 GN=COL12A1 PE=4 SV=2	12.966667	0.301194213	0.77442078	0.890491
G1TE64$$Tubulin beta-4B chain OS=Homo sapiens GN=TUBB4B PE=1 SV=1	10.3	0.300870461	0.77465479	0.890491
G1TI53$$Cullin-2 OS=Homo sapiens GN=CUL2 PE=1 SV=1	15.371461	0.297508166	0.77708671	0.8924169
G1SVD5$$Adducin 1 OS=Oryctolagus cuniculus OX=9986 GN=ADD1 PE=4 SV=1	22.633333	0.297216926	0.7772975	0.8924169
G1STF7$$Lamin-B2 OS=Homo sapiens GN=LMNB2 PE=1 SV=4	-4.633333	-0.296441151	0.77785907	0.8925062

G1TTS1$$""Trifunctional enzyme subunit beta, mitochondrial OS=Homo sapiens GN=HADHB PE=1 SV=3""	-2.9	-0.293731208	0.77982193	0.893879
G1T2W1$$Uncharacterized protein OS=Oryctolagus cuniculus OX=9986 GN=CSDE1 PE=4 SV=2	19.066667	0.290985015	0.78181291	0.893879
G1SP02$$Uncharacterized protein OS=Oryctolagus cuniculus OX=9986 GN=MLYCD PE=4 SV=1	12.433333	0.290932948	0.78185068	0.893879
G1SY93$$Alpha-2-macroglobulin OS=Homo sapiens GN=A2M PE=1 SV=3	7.1666667	0.290703993	0.78201676	0.893879
G1U9U0$$Annexin OS=Oryctolagus cuniculus OX=9986 GN=ANXA5 PE=3 SV=2	16.8	0.290377694	0.78225347	0.893879
G1T0Z8$$Uncharacterized protein OS=Oryctolagus cuniculus OX=9986 GN=AEBP1 PE=4 SV=1	14.776558	0.29027783	0.78232592	0.893879
G1SHU8$$Uncharacterized protein OS=Oryctolagus cuniculus OX=9986 GN=PPCS PE=4 SV=1	-10.56667	-0.289550754	0.78285348	0.893879
G1TUD2$$Eukaryotic translation initiation factor 2 subunit 2 OS=Homo sapiens GN=EIF2S2 PE=1 SV=2	12.166667	0.289443369	0.78293141	0.893879
G1SN95$$""NADH dehydrogenase [ubiquinone] 1 alpha subcomplex subunit 10, mitochondrial OS=Oryctolagus cuniculus OX=9986 GN=NDUFA10 PE=3 SV=2""	-16.70408	-0.288313345	0.78375164	0.8942621
P40826$$Poly(rC)-binding protein 1 OS=Oryctolagus cuniculus OX=9986 GN=PCBP1 PE=2 SV=1	16.433333	0.284653142	0.78641054	0.8967413
G1T6N3$$Uncharacterized protein OS=Oryctolagus cuniculus OX=9986 GN=EFCAB7 PE=4 SV=2	-13.36667	-0.281342964	0.78881795	0.896919
G1SE12$$Uncharacterized protein OS=Oryctolagus cuniculus OX=9986 GN=FDX1 PE=4 SV=2	8.9666667	0.281135843	0.78896867	0.896919
G1SX00$$Uncharacterized protein OS=Oryctolagus cuniculus OX=9986 GN=HMGCL PE=4 SV=1	13.639474	0.28095597	0.78909957	0.896919
Q9TT15$$cAMP-dependent protein kinase type II-alpha regulatory subunit OS=Homo sapiens GN=PRKAR2A PE=1 SV=2	13.733333	0.280584975	0.78936958	0.896919
G1TXB6$$Uncharacterized protein OS=Oryctolagus cuniculus OX=9986 GN=BCS1L PE=3 SV=1	-6.233333	-0.279715435	0.79000257	0.896919
G1TX43$$Uncharacterized protein OS=Oryctolagus cuniculus OX=9986 GN=MAPKAPK3 PE=3 SV=1	14.423825	0.279409887	0.79022504	0.896919

G1SMA1$$""Tyrosine 3-monooxygenase/tryptophan 5-monooxygenase activation protein, eta polypeptide (Predicted) OS=Oryctolagus cuniculus OX=9986 GN=YWHAH PE=3 SV=1""	13.733333	0.278814693	0.79065846	0.896919
G1SQ10$$Uncharacterized protein OS=Oryctolagus cuniculus OX=9986 GN=GLB1 PE=3 SV=1	16.557747	0.278485042	0.79089855	0.896919
G1SQF9$$Uncharacterized protein OS=Oryctolagus cuniculus OX=9986 GN=NUDC PE=4 SV=1	13.366667	0.278323846	0.79101596	0.896919
G1THV8$$40S ribosomal protein S25 OS=Homo sapiens GN=RPS25 PE=1 SV=1	14.4	0.277758591	0.79142772	0.896919
G1SUM3$$Uncharacterized protein OS=Oryctolagus cuniculus OX=9986 PE=4 SV=1	12.766667	0.274656532	0.79368878	0.8988963
G1TIR7$$Uncharacterized protein OS=Oryctolagus cuniculus OX=9986 GN=CRYM PE=4 SV=1	7	0.274028402	0.79414689	0.8988963
P43236$$Fructose-bisphosphate aldolase OS=Oryctolagus cuniculus OX=9986 GN=ALDOC PE=3 SV=1	17	0.272826067	0.79502404	0.8993374
G1T1P3$$Uncharacterized protein OS=Oryctolagus cuniculus OX=9986 GN=KPNB1 PE=4 SV=2	16.633333	0.269893143	0.79716512	0.9012069
G1TRS4$$TPD52 like 2 OS=Oryctolagus cuniculus OX=9986 GN=TPD52L2 PE=4 SV=2	6.7333333	0.268270633	0.79835044	0.9019942
G1STR7$$Uncharacterized protein OS=Oryctolagus cuniculus OX=9986 GN=ECHDC1 PE=3 SV=2	-14.96667	-0.266497312	0.79964662	0.9027482
G1SZ47$$Uncharacterized protein OS=Oryctolagus cuniculus OX=9986 GN=STIM1 PE=4 SV=2	11.066667	0.265572229	0.80032307	0.9027482
G1SDK8$$Uncharacterized protein OS=Oryctolagus cuniculus OX=9986 GN=MTHFD1 PE=3 SV=1	12.1	0.265349969	0.80048563	0.9027482
G1T641$$Immunoglobulin J chain OS=Oryctolagus cuniculus OX=9986 GN=JCHAIN PE=4 SV=1	8.5666667	0.260956508	0.80370116	0.9058208
G1U1Q8$$40S ribosomal protein S7 OS=Oryctolagus cuniculus OX=9986 PE=3 SV=1	-3.6	-0.259395532	0.80484466	0.906492
G1TZV1$$Uncharacterized protein OS=Oryctolagus cuniculus OX=9986 GN=GM2A PE=4 SV=1	-7.233333	-0.257994361	0.80587156	0.906492
G1SYM4$$14 kDa phosphohistidine phosphatase OS=Oryctolagus cuniculus OX=9986 GN=PHPT1 PE=1 SV=1	10	0.257784228	0.80602561	0.906492
G1SEW3$$Transcriptional activator protein Pur-alpha OS=Homo sapiens GN=PURA PE=1 SV=2	-7.833333	-0.256760308	0.80677634	0.906492

G1TA10$$Uncharacterized protein OS=Oryctolagus cuniculus OX=9986 GN=WDR77 PE=4 SV=1	-8.933333	-0.256737475	0.80679309	0.906492
G1U354$$Uncharacterized protein OS=Oryctolagus cuniculus OX=9986 GN=EIF2B3 PE=4 SV=1	-2.9	-0.255545148	0.8076676	0.906492
G1SSB4$$Tensin-1 OS=Homo sapiens GN=TNS1 PE=1 SV=2	10.066667	0.255452003	0.80773593	0.906492
G1T9I4$$60S ribosomal protein L7a OS=Homo sapiens GN=RPL7A PE=1 SV=2	-17.33333	-0.25306886	0.80948483	0.9077484
G1TKQ5$$Uncharacterized protein OS=Oryctolagus cuniculus OX=9986 GN=SLC27A3 PE=4 SV=1	10.1	0.252585833	0.80983946	0.9077484
G1THU6$$Uncharacterized protein OS=Oryctolagus cuniculus OX=9986 GN=STX7 PE=3 SV=1	-2.3	-0.251786451	0.81042645	0.9078548
G1T7C0$$Vesicular integral-membrane protein VIP36 OS=Homo sapiens GN=LMAN2 PE=1 SV=1	13.633333	0.250608184	0.81129192	0.9082728
G1SMG5$$Vacuolar protein sorting-associated protein 29 OS=Homo sapiens GN=VPS29 PE=1 SV=1	6.0333333	0.247207443	0.81379153	0.9101474
G1SWN1$$ATP-dependent 6-phosphofructokinase OS=Oryctolagus cuniculus OX=9986 GN=PFKP PE=3 SV=1	12.911124	0.246530503	0.81428939	0.9101474
G1SUE8$$Adenylyl cyclase-associated protein OS=Oryctolagus cuniculus OX=9986 GN=CAP1 PE=3 SV=1	10.4	0.246317285	0.81444622	0.9101474
G1TKH3$$Sidoreflexin OS=Oryctolagus cuniculus OX=9986 GN=SFXN1 PE=3 SV=1	5.7	0.243269731	0.8166889	0.9121011
U3KMS5$$SWI/SNF complex subunit SMARCC2 OS=Homo sapiens GN=SMARCC2 PE=1 SV=1	11.266667	0.241297122	0.81814156	0.9131707
G1SRR2$$Uncharacterized protein OS=Oryctolagus cuniculus OX=9986 GN=PAFAH1B3 PE=4 SV=1	14.166667	0.237197098	0.82116349	0.9159895
G1U3Q6$$""Hydroxyacylglutathione hydrolase, mitochondrial OS=Homo sapiens GN=HAGH PE=1 SV=2""	11.4	0.236196952	0.82190117	0.9162383
G1T542$$Dynein cytoplasmic 1 heavy chain 1 OS=Oryctolagus cuniculus OX=9986 GN=DYNC1H1 PE=4 SV=1	-9.333333	-0.23527928	0.8225782	0.9162383
G1SUJ3$$Uncharacterized protein OS=Oryctolagus cuniculus OX=9986 GN=COPS8 PE=4 SV=1	-4.433333	-0.234875198	0.82287638	0.9162383
G1TVW1$$""cDNA FLJ60124, highly similar to Mitochondrial dicarboxylate carrier OS=Homo sapiens PE=2 SV=1""	10.066667	0.232590418	0.82456295	0.917517

G1SYJ6$$Uncharacterized protein OS=Oryctolagus cuniculus OX=9986 GN=GCLC PE=4 SV=1	3.9333333	0.231972356	0.82501937	0.917517
G1TAB2$$Integrin beta OS=Oryctolagus cuniculus OX=9986 GN=ITGB1 PE=3 SV=1	-2.866667	-0.230437433	0.8261532	0.917861
G1SRN2$$Uncharacterized protein OS=Oryctolagus cuniculus OX=9986 GN=ACSL1 PE=4 SV=2	16.266667	0.230206771	0.82632363	0.917861
G1T4Q9$$Uncharacterized protein OS=Oryctolagus cuniculus OX=9986 GN=PCBD2 PE=3 SV=1	-10.63333	-0.229200671	0.82706712	0.9181341
G1TE61$$Phosphodiesterase OS=Oryctolagus cuniculus OX=9986 GN=PDE2A PE=3 SV=1	2.2666667	0.226717744	0.82890283	0.9196186
G1T6X7$$Vitamin D-binding protein OS=Oryctolagus cuniculus OX=9986 GN=GC PE=4 SV=1	4.1	0.225001011	0.83017277	0.920474
G1U3G0$$Inter-alpha-trypsin inhibitor heavy chain H3 OS=Oryctolagus cuniculus OX=9986 GN=ITIH3 PE=4 SV=1	11.403798	0.223664145	0.83116211	0.9210175
G1T6H0$$Uncharacterized protein OS=Oryctolagus cuniculus OX=9986 GN=UBXN4 PE=4 SV=1	9.2333333	0.222389582	0.83210566	0.9215096
G1T3G8$$Uncharacterized protein OS=Oryctolagus cuniculus OX=9986 GN=CRK PE=4 SV=2	5.1666667	0.221375972	0.83285626	0.9215221
G1TDQ2$$Uncharacterized protein OS=Oryctolagus cuniculus OX=9986 PE=3 SV=2	2.6666667	0.220621128	0.83341536	0.9215221
G1SZ12$$Uncharacterized protein OS=Oryctolagus cuniculus OX=9986 GN=FAM136A PE=4 SV=1	4.5333333	0.219183331	0.83448062	0.9215221
G1SQD1$$Ras-related protein Rab-35 OS=Homo sapiens GN=RAB35 PE=1 SV=1	11	0.218990693	0.83462338	0.9215221
G1STH6$$Peripherin OS=Homo sapiens GN=PRPH PE=1 SV=2	12.459625	0.217929986	0.83540954	0.9215221
G1T8P3$$Uncharacterized protein OS=Oryctolagus cuniculus OX=9986 GN=RPL23 PE=1 SV=1	12.3	0.217690395	0.83558715	0.9215221
Q9TTJ6$$Uncharacterized protein OS=Oryctolagus cuniculus OX=9986 PE=4 SV=1	-10.18105	-0.217655171	0.83561326	0.9215221
U3KPE6$$Methyl-CpG-binding protein 2 OS=Oryctolagus cuniculus OX=9986 GN=MECP2 PE=4 SV=2	9.2759819	0.216446707	0.83650927	0.9219591
G1SLX0$$Actin-related protein 2/3 complex subunit 3 OS=Oryctolagus cuniculus OX=9986 GN=ARPC3 PE=3 SV=1	-3.2	-0.212810892	0.83920666	0.9230186
G1T868$$Nuclease-sensitive element-binding protein 1 (Fragment) OS=Homo sapiens GN=YBX1 PE=1 SV=1	-7.333333	-0.212344757	0.83955266	0.9230186

G1U5W5$$Heterogeneous nuclear ribonucleoprotein Q OS=Homo sapiens GN=SYNCRIP PE=1 SV=2	7.9955613	0.212236204	0.83963324	0.9230186
G1T9F3$$Uncharacterized protein OS=Oryctolagus cuniculus OX=9986 GN=C11orf54 PE=4 SV=1	5.2333333	0.211831017	0.83993404	0.9230186
G1TVX2$$Dehydrogenase/reductase SDR family member 4 (Fragment) OS=Oryctolagus cuniculus OX=9986 GN=DHRS4 PE=1 SV=1	4.4	0.21038769	0.84100578	0.9230186
G1T5V8$$Collagen alpha-3(VI) chain OS=Homo sapiens GN=COL6A3 PE=1 SV=5	7.6333333	0.210358635	0.84102736	0.9230186
G1TNH9$$Uncharacterized protein OS=Oryctolagus cuniculus OX=9986 GN=ACY1 PE=4 SV=1	-4.4	-0.210222583	0.8411284	0.9230186
G1SG55$$Lysosomal associated membrane protein 1 OS=Oryctolagus cuniculus OX=9986 GN=LAMP1 PE=3 SV=2	6.6333333	0.209758947	0.84147277	0.9230186
G1SL68$$PDZ and LIM domain protein 3 OS=Homo sapiens GN=PDLIM3 PE=1 SV=1	14.933333	0.208126803	0.84268536	0.9236124
G1SYD3$$Uncharacterized protein OS=Oryctolagus cuniculus OX=9986 GN=GSTM4 PE=4 SV=1	13.866667	0.207580213	0.84309155	0.9236124
G1TVF7$$""Glycogen phosphorylase, brain form OS=Homo sapiens GN=PYGB PE=1 SV=5""	11.576777	0.206685613	0.84375648	0.9236124
P00939$$Tubulin beta chain OS=Homo sapiens GN=TUBB PE=1 SV=2	13.366667	0.206335809	0.84401652	0.9236124
G1T5E6$$Plasmalemma vesicle associated protein OS=Oryctolagus cuniculus OX=9986 GN=PLVAP PE=4 SV=1	7.9333333	0.202659433	0.84675081	0.925977
G1SLF1$$Rho guanine nucleotide exchange factor 7 OS=Homo sapiens GN=ARHGEF7 PE=1 SV=2	3.2	0.201235443	0.84781054	0.925977
G1SZT4$$Uncharacterized protein OS=Oryctolagus cuniculus OX=9986 GN=PDPR PE=4 SV=2	-3.1	-0.200899847	0.84806034	0.925977
G1SET0$$Protein transport protein Sec61 subunit alpha isoform 1 OS=Homo sapiens GN=SEC61A1 PE=1 SV=1	10.2	0.200226889	0.84856132	0.925977
G1T489$$T-complex protein 1 subunit epsilon OS=Oryctolagus cuniculus OX=9986 GN=CCT5 PE=3 SV=1	15.3	0.200058354	0.8486868	0.925977
G1T4Z7$$Annexin OS=Oryctolagus cuniculus OX=9986 GN=ANXA6 PE=3 SV=2	11.033333	0.196188615	0.85156924	0.9285728
G1T3U7$$Uncharacterized protein OS=Oryctolagus cuniculus OX=9986 PE=4 SV=1	3.3	0.195045018	0.85242156	0.9287424

G1TI71$$Uncharacterized protein OS=Oryctolagus cuniculus OX=9986 GN=LOC100355470 PE=4 SV=2	-3.233333	-0.194629237	0.8527315	0.9287424
G1T5K3$$Uncharacterized protein OS=Oryctolagus cuniculus OX=9986 GN=TPP2 PE=4 SV=2	-10.93235	-0.193914963	0.85326401	0.9287741
G1T168$$Bifunctional glutamate/proline--tRNA ligase OS=Homo sapiens GN=EPRS PE=1 SV=5	-11.73333	-0.192694611	0.85417402	0.9292164
G1SV75$$Ras-related protein Rab-2A OS=Homo sapiens GN=RAB2A PE=1 SV=1	5.9333333	0.190745717	0.85562782	0.9302495
G1SYI3$$Uncharacterized protein OS=Oryctolagus cuniculus OX=9986 GN=CHGB PE=4 SV=1	-8.433333	-0.189515218	0.85654605	0.9306993
G1SIT0$$Cadherin-13 OS=Homo sapiens GN=CDH13 PE=1 SV=1	3.5333333	0.187322413	0.85818301	0.9307153
G1SWM1$$Mitochondrial import inner membrane translocase subunit TIM50 OS=Oryctolagus cuniculus OX=9986 GN=TIMM50 PE=3 SV=1	7.3	0.186944362	0.85846531	0.9307153
G1SCQ1$$Vacuolar ATPase assembly integral membrane protein VMA21 OS=Oryctolagus cuniculus OX=9986 GN=VMA21 PE=3 SV=2	6.7333333	0.186625465	0.85870345	0.9307153
G1T5Z7$$Uncharacterized protein OS=Oryctolagus cuniculus OX=9986 GN=NPM1 PE=4 SV=1	1.6333333	0.186431235	0.85884851	0.9307153
G1SDG8$$""Procollagen-lysine,2-oxoglutarate 5-dioxygenase 3 OS=Oryctolagus cuniculus OX=9986 GN=PLOD3 PE=4 SV=1""	6.9333333	0.186117205	0.85908305	0.9307153
G1TBR5$$Cysteine-rich and transmembrane domain-containing protein 1 OS=Homo sapiens GN=CYSTM1 PE=1 SV=1	-2.066667	-0.184836399	0.8600398	0.9308766
U3KNE2$$Uncharacterized protein OS=Oryctolagus cuniculus OX=9986 GN=GSTZ1 PE=3 SV=1	-4.633333	-0.182455441	0.86181907	0.9308766
G1TEA8$$ADP/ATP translocase 1 OS=Oryctolagus cuniculus OX=9986 GN=SLC25A4 PE=2 SV=3	-8.4	-0.182414573	0.86184962	0.9308766
G1SV99$$Dihydrolipoamide acetyltransferase component of pyruvate dehydrogenase complex OS=Oryctolagus cuniculus OX=9986 GN=PDHX PE=3 SV=2	-9.846498	-0.181831808	0.86228526	0.9308766
G1SI85$$Uncharacterized protein OS=Oryctolagus cuniculus OX=9986 GN=GSTO1 PE=3 SV=1	9.2019828	0.181495253	0.86253687	0.9308766

G1TVT0$$""Iron-sulfur cluster assembly enzyme ISCU, mitochondrial OS=Homo sapiens GN=ISCU PE=1 SV=2""	6.5333333	0.181347434	0.86264738	0.9308766
G1TCS8$$Adenosylhomocysteinase OS=Oryctolagus cuniculus OX=9986 GN=AHCYL1 PE=3 SV=1	-4.033333	-0.180285421	0.8634415	0.9308766
G1SLI0$$Uncharacterized protein OS=Oryctolagus cuniculus OX=9986 GN=FADS2 PE=4 SV=1	6.6666667	0.179804027	0.86380152	0.9308766
G1SXD6$$Peroxiredoxin-4 OS=Homo sapiens GN=PRDX4 PE=1 SV=1	6.4	0.178713452	0.86461726	0.9308766
G1T3A2$$Uncharacterized protein OS=Oryctolagus cuniculus OX=9986 GN=SLC25A3 PE=3 SV=2	5.1333333	0.178132791	0.86505166	0.9308766
G1SSH5$$40S ribosomal protein S11 OS=Homo sapiens GN=RPS11 PE=1 SV=3	8.7	0.177143342	0.86579202	0.9308766
G1SZ19$$Uncharacterized protein OS=Oryctolagus cuniculus OX=9986 GN=NID2 PE=4 SV=1	-9.409529	-0.176559519	0.86622893	0.9308766
B7NZG7$$60S ribosomal protein L26 OS=Homo sapiens GN=RPL26 PE=1 SV=1	9.4666667	0.175927424	0.86670203	0.9308766
G1T601$$Uncharacterized protein OS=Oryctolagus cuniculus OX=9986 GN=SPTLC1 PE=4 SV=2	-10.20824	-0.174824007	0.86752805	0.9308766
G1TDM9$$Annexin OS=Oryctolagus cuniculus OX=9986 GN=ANXA1 PE=3 SV=1	10.024023	0.17375308	0.86832992	0.9308766
G1TSK0$$Serine/threonine-protein phosphatase 2A activator OS=Oryctolagus cuniculus OX=9986 GN=PTPA PE=3 SV=2	-16.30217	-0.173695826	0.86837279	0.9308766
G1TVG7$$Echinoderm microtubule-associated protein-like 2 OS=Homo sapiens GN=EML2 PE=1 SV=1	1.4666667	0.173607518	0.86843892	0.9308766
G1T312$$Uncharacterized protein OS=Oryctolagus cuniculus OX=9986 GN=CBX1 PE=4 SV=1	6.1333333	0.172626451	0.86917369	0.9308766
G1SKL7$$Uncharacterized protein OS=Oryctolagus cuniculus OX=9986 GN=RALA PE=4 SV=1	13.066667	0.172505503	0.86926428	0.9308766
G1U448$$""Succinate--CoA ligase [ADP/GDP-forming] subunit alpha, mitochondrial OS=Oryctolagus cuniculus OX=9986 GN=SUCLG1 PE=3 SV=1""	3.5666667	0.172427471	0.86932273	0.9308766
G1T1B8$$Nucleobindin 1 OS=Oryctolagus cuniculus OX=9986 GN=NUCB1 PE=4 SV=1	8.4808985	0.170046136	0.8711069	0.9322461
G1T3V2$$Uncharacterized protein OS=Oryctolagus cuniculus OX=9986 GN=FAM107B PE=4 SV=1	-3.533333	-0.168231555	0.87246701	0.9331604

G1TI68$$Uncharacterized protein OS=Oryctolagus cuniculus OX=9986 GN=LOC100354715 PE=3 SV=1	2.8	0.167073152	0.87333554	0.9335481
G1TF20$$""Acylglycerol kinase, mitochondrial OS=Homo sapiens GN=AGK PE=1 SV=2""	10.200188	0.1639327	0.87569114	0.9348849
G1T156$$Myosin light chain 12B OS=Oryctolagus cuniculus OX=9986 GN=MYL12B PE=4 SV=2	-3.666667	-0.163873148	0.87573582	0.9348849
G1SP27$$Transgelin OS=Oryctolagus cuniculus OX=9986 GN=TAGLN PE=3 SV=1	8.093159	0.163160351	0.87627068	0.9348849
G1SZL5$$Dihydropyrimidine dehydrogenase [NADP(+)] OS=Oryctolagus cuniculus OX=9986 GN=DPYD PE=3 SV=1	-2.566667	-0.162704342	0.8766129	0.9348849
G1SIV7$$Uncharacterized protein OS=Oryctolagus cuniculus OX=9986 GN=USP39 PE=4 SV=1	4.5	0.161635065	0.87741546	0.9352002
G1T5I9$$Uncharacterized protein OS=Oryctolagus cuniculus OX=9986 GN=HSPA8 PE=3 SV=1	-7.064673	-0.160814119	0.87803174	0.9353167
G1SPM5$$Uncharacterized protein OS=Oryctolagus cuniculus OX=9986 GN=EPB41L2 PE=4 SV=1	3.6666667	0.159631856	0.87891944	0.9356906
G1SWU1$$Uncharacterized protein OS=Oryctolagus cuniculus OX=9986 GN=LOC100346986 PE=3 SV=2	2.0333333	0.158819139	0.87952977	0.9356906
G1STX4$$Phosphoserine aminotransferase OS=Oryctolagus cuniculus OX=9986 GN=PSAT1 PE=3 SV=2	8.9908405	0.15832072	0.87990412	0.9356906
G1T5H8$$Uncharacterized protein OS=Oryctolagus cuniculus OX=9986 GN=RAP2C PE=4 SV=2	4.1333333	0.155656962	0.88190539	0.9367519
G1T1H9$$Acylphosphatase OS=Oryctolagus cuniculus OX=9986 GN=ACYP1 PE=3 SV=1	-7.366667	-0.155567156	0.88197288	0.9367519
G1SY88$$Uncharacterized protein OS=Oryctolagus cuniculus OX=9986 GN=GORASP2 PE=4 SV=1	-1.333333	-0.154965026	0.8824254	0.9367519
G1SS37$$Uncharacterized protein OS=Oryctolagus cuniculus OX=9986 GN=PCYOX1L PE=3 SV=1	8.083446	0.153649502	0.88341424	0.9370924
G1U7I9$$Uncharacterized protein OS=Oryctolagus cuniculus OX=9986 GN=NUDT12 PE=4 SV=2	4.6333333	0.153186976	0.88376196	0.9370924
G1SVE6$$Uncharacterized protein OS=Oryctolagus cuniculus OX=9986 GN=RPS3 PE=1 SV=2	-1.766667	-0.147952895	0.88769887	0.9407263
G1STF9$$Uncharacterized protein OS=Oryctolagus cuniculus OX=9986 PE=4 SV=1	3.4333333	0.145495266	0.88954867	0.9419929

G1T6S0$$Eukaryotic translation initiation factor 3 subunit B OS=Oryctolagus cuniculus OX=9986 GN=EIF3B PE=1 SV=1	6.4333333	0.145008416	0.88991521	0.9419929
G1SKQ9$$Uncharacterized protein OS=Oryctolagus cuniculus OX=9986 GN=RHOG PE=4 SV=1	8.1992298	0.142269857	0.89197755	0.9435841
G1T4Z1$$Uncharacterized protein OS=Oryctolagus cuniculus OX=9986 GN=AIMP2 PE=4 SV=2	2.7	0.141138147	0.89283009	0.9435841
G1SWR0$$Uncharacterized protein OS=Oryctolagus cuniculus OX=9986 GN=CUTC PE=3 SV=1	-9.833142	-0.140711412	0.8931516	0.9435841
G1TQP4$$Cytochrome b-c1 complex subunit 7 OS=Oryctolagus cuniculus OX=9986 GN=UQCRB PE=3 SV=2	7.3816498	0.140296553	0.89346418	0.9435841
G1SDY5$$Uncharacterized protein OS=Oryctolagus cuniculus OX=9986 PE=4 SV=1	-3.133333	-0.138541482	0.89478681	0.9442496
G1T8K2$$Uncharacterized protein OS=Oryctolagus cuniculus OX=9986 GN=PACSIN2 PE=4 SV=1	2.3666667	0.13800983	0.89518754	0.9442496
G1TUE1$$Uncharacterized protein OS=Oryctolagus cuniculus OX=9986 GN=CALD1 PE=4 SV=1	7.2040315	0.137330669	0.89569951	0.9442496
G1TUM2$$Uncharacterized protein OS=Oryctolagus cuniculus OX=9986 GN=THEM4 PE=4 SV=1	-6.4	-0.136744455	0.89614145	0.9442496
G1T3D8$$Laminin subunit gamma 1 OS=Oryctolagus cuniculus OX=9986 GN=LAMC1 PE=4 SV=2	4.5333333	0.134725849	0.8976636	0.9453135
G1SJL6$$Uncharacterized protein OS=Oryctolagus cuniculus OX=9986 GN=CD68 PE=3 SV=1	8.3724826	0.13313809	0.8988612	0.9458407
G1U9B4$$Uncharacterized protein OS=Oryctolagus cuniculus OX=9986 GN=HSDL2 PE=4 SV=2	5.7	0.132702903	0.8991895	0.9458407
G1U7Q6$$L-lactate dehydrogenase OS=Oryctolagus cuniculus OX=9986 GN=LDHA PE=3 SV=1	7.6157066	0.128922512	0.90204233	0.9483009
G1TM95$$26S proteasome non-ATPase regulatory subunit 1 OS=Oryctolagus cuniculus OX=9986 GN=PSMD1 PE=3 SV=1	6.9507245	0.127829751	0.90286728	0.9486276
G1T0C5$$Sulfotransferase 1C2 OS=Oryctolagus cuniculus OX=9986 GN=SULT1C2 PE=1 SV=1	10.233333	0.126589428	0.90380379	0.9488794
G1TX63$$Uncharacterized protein OS=Oryctolagus cuniculus OX=9986 GN=NSFL1C PE=4 SV=2	6.544378	0.126150126	0.90413553	0.9488794
G1TCM9$$Uncharacterized protein OS=Oryctolagus cuniculus OX=9986 GN=DECR1 PE=4 SV=1	-3.266667	-0.124450165	0.90541946	0.9491609

G1TE13$$Uncharacterized protein OS=Oryctolagus cuniculus OX=9986 GN=LOC100346892 PE=4 SV=1	3.0333333	0.124275133	0.90555167	0.9491609
G1SGV9$$Uncharacterized protein OS=Oryctolagus cuniculus OX=9986 GN=UQCRQ PE=4 SV=1	6.4	0.123429019	0.90619085	0.9491609
G1SPG6$$Uncharacterized protein OS=Oryctolagus cuniculus OX=9986 GN=NFS1 PE=3 SV=1	-5.633333	-0.122794256	0.90667042	0.9491609
Q28740$$Ras-related C3 botulinum toxin substrate 3 OS=Homo sapiens GN=RAC3 PE=1 SV=1	5.7	0.122389871	0.90697596	0.9491609
G1TMZ6$$Protein RPL17-C18orf32 OS=Homo sapiens GN=RPL17-C18orf32 PE=3 SV=1	5.3	0.121302072	0.90779795	0.9494826
P17177$$""HLA-B associated transcript 5, isoform CRA_b OS=Homo sapiens GN=ABHD16A PE=1 SV=1""	4.0333333	0.119829799	0.90891068	0.9499496
P42675$$Calreticulin OS=Oryctolagus cuniculus OX=9986 GN=CALR PE=1 SV=1	7.1819635	0.119348867	0.90927421	0.9499496
G1SUY8$$RuvB-like 1 OS=Homo sapiens GN=RUVBL1 PE=1 SV=1	4.0666667	0.109867786	0.91644579	0.9564479
G1TCQ4$$N-acetylglucosamine-6-phosphate deacetylase OS=Oryctolagus cuniculus OX=9986 GN=AMDHD2 PE=3 SV=1	4.6666667	0.109243009	0.9169187	0.9564479
G1SEI0$$Nucleoplasmin-3 OS=Homo sapiens GN=NPM3 PE=1 SV=3	3.9	0.109070193	0.91704951	0.9564479
G1TFV7$$Uncharacterized protein OS=Oryctolagus cuniculus OX=9986 GN=PGM2 PE=4 SV=2	-7.066667	-0.107229459	0.91844305	0.9567474
G1T6Q9$$Uncharacterized protein OS=Oryctolagus cuniculus OX=9986 GN=NSF PE=4 SV=1	9.1333333	0.107125252	0.91852195	0.9567474
G1SYK4$$Polyadenylate-binding protein OS=Oryctolagus cuniculus OX=9986 GN=PABPC1 PE=3 SV=1	-4.554873	-0.106636108	0.91889232	0.9567474
G1SNP4$$""Lon protease homolog, mitochondrial OS=Homo sapiens GN=LONP1 PE=1 SV=2""	-3.433333	-0.103733674	0.92109044	0.9584951
G1TR00$$Serine/arginine repetitive matrix 2 OS=Oryctolagus cuniculus OX=9986 GN=SRRM2 PE=4 SV=2	-4.801492	-0.102436638	0.92207299	0.9589767
G1SLD5$$Profilin-1 OS=Homo sapiens GN=PFN1 PE=1 SV=2	2.3333333	0.099978265	0.92393571	0.9600542
G1SF47$$Ribosomal protein OS=Oryctolagus cuniculus OX=9986 GN=RPL10A PE=1 SV=2	3.1333333	0.099644484	0.92418866	0.9600542
G1SWY6$$Uncharacterized protein OS=Oryctolagus cuniculus OX=9986 GN=MYH10 PE=3 SV=2	3.9333333	0.098840247	0.92479818	0.9600542

G1T7X6$$Uncharacterized protein OS=Oryctolagus cuniculus OX=9986 GN=SRI PE=4 SV=2	-1.9	-0.098322671	0.92519047	0.9600542
G1SQR7$$Uncharacterized protein OS=Oryctolagus cuniculus OX=9986 GN=C12orf10 PE=4 SV=1	-5.966667	-0.090878275	0.93083543	0.965142
G1T6W7$$Uncharacterized protein OS=Oryctolagus cuniculus OX=9986 GN=FBLN5 PE=4 SV=1	5.0666667	0.090477126	0.93113975	0.965142
G1SH95$$Hemoglobin subunit alpha-1/2 OS=Oryctolagus cuniculus OX=9986 PE=1 SV=2	5	0.088142745	0.93291089	0.9653597
P29751$$Uncharacterized protein OS=Oryctolagus cuniculus OX=9986 GN=RTCA PE=3 SV=1	-3.866667	-0.087314657	0.93353928	0.9653597
G1U7K9$$Uncharacterized protein OS=Oryctolagus cuniculus OX=9986 GN=SPATS2L PE=4 SV=2	-4.233333	-0.08716078	0.93365606	0.9653597
G1SK48$$""Cytochrome c1, heme protein, mitochondrial OS=Homo sapiens GN=CYC1 PE=1 SV=3""	-4.133333	-0.085870513	0.93463529	0.9653597
G1SY85$$Voltage-dependent anion-selective channel protein 1 OS=Homo sapiens GN=VDAC1 PE=1 SV=2	4.5064087	0.085671905	0.93478604	0.9653597
G1TPR9$$Uncharacterized protein OS=Oryctolagus cuniculus OX=9986 GN=ASAH1 PE=4 SV=2	2.4333333	0.085662604	0.93479309	0.9653597
G1TM82$$Uncharacterized protein OS=Oryctolagus cuniculus OX=9986 GN=SH3BGRL PE=4 SV=2	-0.7	-0.084380899	0.93576598	0.9653597
G1STX7$$Eukaryotic translation initiation factor 4 gamma 1 OS=Oryctolagus cuniculus OX=9986 GN=EIF4G1 PE=4 SV=2	0.8333333	0.084351915	0.93578798	0.9653597
G1TC48$$Uncharacterized protein OS=Oryctolagus cuniculus OX=9986 GN=VPS4B PE=3 SV=2	3.8140866	0.08399512	0.93605883	0.9653597
G1SHS7$$Uncharacterized protein OS=Oryctolagus cuniculus OX=9986 GN=LOC100344410 PE=3 SV=1	-3.566667	-0.082253136	0.93738135	0.9661689
P21195$$Uncharacterized protein OS=Oryctolagus cuniculus OX=9986 GN=TGM2 PE=4 SV=2	-0.933333	-0.080909298	0.93840174	0.9661689
G1STV0$$Uncharacterized protein OS=Oryctolagus cuniculus OX=9986 GN=COPS4 PE=4 SV=1	-2.966667	-0.080892544	0.93841446	0.9661689
G1TYY5$$Mitochondrial carrier homolog 1 OS=Homo sapiens GN=MTCH1 PE=1 SV=1	-2.2	-0.078000655	0.94061076	0.96789
G1SPB2$$Methylmalonyl-CoA epimerase OS=Oryctolagus cuniculus OX=9986 GN=MCEE PE=4 SV=1	3.9868135	0.076602101	0.94167312	0.9684431

O19049$$Uncharacterized protein OS=Oryctolagus cuniculus OX=9986 GN=HADH PE=4 SV=2	2.5666667	0.075182753	0.94275142	0.9690119
Q09YN4$$Uncharacterized protein OS=Oryctolagus cuniculus OX=9986 GN=RPS13 PE=1 SV=1	-2.966667	-0.074122856	0.94355673	0.9692997
G1SS79$$Uncharacterized protein OS=Oryctolagus cuniculus OX=9986 GN=RPS23 PE=1 SV=1	0.6	0.071563271	0.94550181	0.9707573
G1T1D9$$Endophilin-B1 OS=Homo sapiens GN=SH3GLB1 PE=1 SV=1	0.6666667	0.069750667	0.9468795	0.9716311
G1T3V0$$Uncharacterized protein OS=Oryctolagus cuniculus OX=9986 GN=PSME1 PE=4 SV=1	-1.8	-0.065823048	0.94986542	0.9740032
G1TMQ4$$Uncharacterized protein OS=Oryctolagus cuniculus OX=9986 PE=4 SV=1	0.5666667	0.064494836	0.95087539	0.9740032
G1SVB6$$Ras-related protein Rab-7a OS=Oryctolagus cuniculus OX=9986 GN=RAB7A PE=4 SV=1	3.1666667	0.064226857	0.95107917	0.9740032
G1SD60$$Lupus La protein homolog OS=Oryctolagus cuniculus OX=9986 GN=SSB PE=4 SV=1	-1.666667	-0.063932699	0.95130287	0.9740032
G1SWF0$$Pyridoxal phosphate homeostasis protein OS=Oryctolagus cuniculus OX=9986 GN=PLPBP PE=3 SV=1	0.5	0.062732392	0.9522157	0.9743971
G1TJW8$$Uncharacterized protein OS=Oryctolagus cuniculus OX=9986 GN=ALDH9A1 PE=3 SV=2	-2.815675	-0.060352135	0.95402613	0.9757085
G1TJH2$$40S ribosomal protein S24 OS=Oryctolagus cuniculus OX=9986 GN=RPS24 PE=1 SV=1	-1.833333	-0.0596331	0.95457309	0.9757271
G1SIB6$$""ATP-dependent Clp protease proteolytic subunit, mitochondrial OS=Homo sapiens GN=CLPP PE=1 SV=1""	-3.566667	-0.056663722	0.95683215	0.9774946
B7NZM8$$Uncharacterized protein OS=Oryctolagus cuniculus OX=9986 GN=ACADS PE=3 SV=1	0.7	0.055374225	0.95781332	0.9779555
G1ST69$$Uncharacterized protein OS=Oryctolagus cuniculus OX=9986 GN=CBX5 PE=4 SV=2	0.7	0.053722307	0.95907037	0.9786974
G1ST95$$Uncharacterized protein OS=Oryctolagus cuniculus OX=9986 GN=VAT1L PE=4 SV=1	2.4	0.052860988	0.95972586	0.9788249
G1SVV6$$Uncharacterized protein OS=Oryctolagus cuniculus OX=9986 GN=MTCH2 PE=3 SV=1	-2.166667	-0.048720904	0.96287706	0.9814701
G1SG48$$Phosphatidate cytidylyltransferase OS=Oryctolagus cuniculus OX=9986 GN=CDS2 PE=3 SV=1	2.2839405	0.048055838	0.96338334	0.9814701

G1SEA8$$Tropomyosin alpha-1 chain OS=Oryctolagus cuniculus OX=9986 GN=TPM1 PE=3 SV=2	2.4666667	0.046267221	0.96474503	0.9823149
G1T0E9$$Uncharacterized protein OS=Oryctolagus cuniculus OX=9986 GN=MARCKS PE=4 SV=1	0.6666667	0.04530117	0.96548054	0.9823365
G1T6G1$$Beta-hexosaminidase subunit beta OS=Homo sapiens GN=HEXB PE=1 SV=3	2.8666667	0.044840764	0.96583109	0.9823365
G1SLT8$$Nipsnap homolog 2 OS=Oryctolagus cuniculus OX=9986 GN=NIPSNAP2 PE=4 SV=2	-1	-0.04129524	0.96853091	0.9845397
G1TZJ6$$Adenylate kinase isoenzyme 1 OS=Oryctolagus cuniculus OX=9986 GN=AK1 PE=3 SV=1	-0.9	-0.038062428	0.97099301	0.986499
G1T625$$Uncharacterized protein OS=Oryctolagus cuniculus OX=9986 GN=TPR PE=4 SV=1	-1.888059	-0.036776835	0.97197221	0.9869503
G1SLF5$$Apolipoprotein A-I OS=Oryctolagus cuniculus OX=9986 GN=APOA1 PE=3 SV=1	-1.710123	-0.033638777	0.97436262	0.9888334
G1U603$$Uncharacterized protein OS=Oryctolagus cuniculus OX=9986 GN=VPS13C PE=4 SV=2	-1.133333	-0.031561523	0.97594514	0.9898949
G1T0N4$$Uncharacterized protein OS=Oryctolagus cuniculus OX=9986 GN=HP1BP3 PE=4 SV=1	0.5333333	0.029602338	0.97743781	0.9904281
G1TA59$$DNA-(apurinic or apyrimidinic site) lyase OS=Oryctolagus cuniculus OX=9986 GN=APEX1 PE=3 SV=1	0.7333333	0.029425753	0.97757235	0.9904281
G1T5Q8$$Uncharacterized protein OS=Oryctolagus cuniculus OX=9986 GN=DHX9 PE=4 SV=1	0.6666667	0.028757765	0.97808131	0.9904281
G1T8N8$$Apolipoprotein A-I OS=Oryctolagus cuniculus OX=9986 GN=APOA1 PE=1 SV=2	-1.244562	-0.026990644	0.97942778	0.9908147
G1T6L5$$Testin OS=Oryctolagus cuniculus OX=9986 GN=TES PE=4 SV=1	-2.066667	-0.026847157	0.97953712	0.9908147
G1TGF1$$Retinol-binding protein OS=Oryctolagus cuniculus OX=9986 GN=RBP4 PE=3 SV=1	0.7666667	0.023518659	0.9820735	0.992836
P00883$$Uncharacterized protein OS=Oryctolagus cuniculus OX=9986 GN=COQ9 PE=4 SV=1	-1.033333	-0.021052838	0.98395266	0.9935604
G1SED9$$Uncharacterized protein OS=Oryctolagus cuniculus OX=9986 GN=GHITM PE=3 SV=1	-0.9	-0.020800955	0.98414463	0.9935604
G1TLE4$$Uncharacterized protein OS=Oryctolagus cuniculus OX=9986 GN=FITM2 PE=4 SV=1	0.2333333	0.020458458	0.98440565	0.9935604
G1T4Y7$$Kinesin-1 heavy chain OS=Homo sapiens GN=KIF5B PE=1 SV=1	-0.966667	-0.018330041	0.9860278	0.9939153

G1TK63$$Uncharacterized protein OS=Oryctolagus cuniculus OX=9986 GN=CD300LG PE=4 SV=2	-1.044761	-0.01750077	0.98665983	0.9939153
P83468$$Uncharacterized protein OS=Oryctolagus cuniculus OX=9986 GN=SEC31A PE=4 SV=1	0.6333333	0.015538298	0.9881556	0.9939153
G1TD16$$""Glutamate dehydrogenase 1, mitochondrial OS=Homo sapiens GN=GLUD1 PE=1 SV=2""	0.7	0.015204857	0.98840975	0.9939153
G1TD91$$Uncharacterized protein OS=Oryctolagus cuniculus OX=9986 GN=FAM210B PE=4 SV=1	0.1666667	0.014725195	0.98877535	0.9939153
G1T4H3$$Uncharacterized protein OS=Oryctolagus cuniculus OX=9986 GN=TLN2 PE=4 SV=1	0.4	0.013920277	0.98938887	0.9939153
G1SHQ2$$Uncharacterized protein OS=Oryctolagus cuniculus OX=9986 GN=DDX23 PE=4 SV=1	0.3	0.01389339	0.98940937	0.9939153
G1SZQ7$$Queuosine salvage protein OS=Oryctolagus cuniculus OX=9986 GN=C9orf64 PE=3 SV=1	-0.433333	-0.013146713	0.9899785	0.9939153
G1SPU6$$Uncharacterized protein OS=Oryctolagus cuniculus OX=9986 GN=TNXB PE=4 SV=1	0.1	0.013117225	0.99000098	0.9939153
G1TMP7$$Uncharacterized protein OS=Oryctolagus cuniculus OX=9986 PE=4 SV=1	0.8	0.012379041	0.99056365	0.9939153
G1TQS9$$Malate dehydrogenase OS=Oryctolagus cuniculus OX=9986 GN=MDH1 PE=3 SV=1	-0.233333	-0.012222405	0.99068304	0.9939153
G1U6T0$$Protein phosphatase 2 scaffold subunit Aalpha OS=Oryctolagus cuniculus OX=9986 GN=PPP2R1A PE=4 SV=1	-0.483892	-0.009754538	0.99256418	0.9951072
G1TWK1$$Uncharacterized protein OS=Oryctolagus cuniculus OX=9986 GN=TMEM159 PE=4 SV=2	-0.633333	-0.008876349	0.9932336	0.9951072
G1T0T5$$ADP-ribosylation factor-like protein 8B OS=Homo sapiens GN=ARL8B PE=1 SV=1	0.4594872	0.008541059	0.99348918	0.9951072
P12337$$RNA-binding protein 14 OS=Homo sapiens GN=RBM14 PE=1 SV=2	0.1	0.007737045	0.99410206	0.9951808
G1U7C1$$Transthyretin OS=Oryctolagus cuniculus OX=9986 GN=TTR PE=3 SV=1	0.0666667	0.006734048	0.99486663	0.9954061
G1STH4$$GMP reductase OS=Oryctolagus cuniculus OX=9986 GN=GMPR2 PE=3 SV=1	0.2333333	0.005850809	0.99553991	0.9955399

Supplement 5

Protein ID	Gene Name	Description
G1U0B3	SLC25A1	Solute carrier family 25 member 1
G1U866	EFCAB7	Uncharacterized protein
G1U001	LOC100352574	Uncharacterized protein
G1SS33	ATP6V1E1	Uncharacterized protein
U3KLT5	EIF5B	Uncharacterized protein
G1TY57		Uncharacterized protein
G1SDL9	VTI1B	Uncharacterized protein
G1U8P2	RPL19	Ribosomal protein L19
G1SRG3	DIS3	Uncharacterized protein
G1SUG3	PLGRKT	Uncharacterized protein
G1SRD2	ACADL	Uncharacterized protein
G1TI39	RDX	Uncharacterized protein
G1TX59	PTPA	Serine/threonine-protein phosphatase 2A activator
G1T5I9	CPSF6	Uncharacterized protein
G1TCD4	FAM114A2	Uncharacterized protein
G1T9I3	PLAA	Uncharacterized protein
G1SE36	H6PD	GDH/6PGL endoplasmic bifunctional protein
G1SZ44	HSPE1	Uncharacterized protein
G1TK17	LOC100338112	40S ribosomal protein S4
U3KNL6	CCDC93	Uncharacterized protein
G1SMM5	DNAJA2	Uncharacterized protein
G1SFW1	TKFC	Triokinase and FMN cyclase
G1T5Y2	FAM49B	Uncharacterized protein
G1TS23	RPS17	Ribosomal protein S17
P15253	CALR	Calreticulin
G1SPQ0	PDLIM3	Uncharacterized protein
G1SZ93	IARS2	Uncharacterized protein
P16973	LYZ	Lysozyme C
G1SD01	SPTB	Spectrin beta chain
G1SGG6	LSS	Terpene cyclase/mutase family member
U3KMU6		Uncharacterized protein
G1U6R8	LOC100352842	Uncharacterized protein
G1SWY6	EEA1	Early endosome antigen 1
G1T9V4	PSMA6	Proteasome subunit alpha type
G1T4H3	PDIA6	Uncharacterized protein
G1U9S6	LOC100357230	Uncharacterized protein
G1TBU9	ACAA2	Uncharacterized protein
G1TDD2	UBXN1	Uncharacterized protein
G1SQG1	EMC4	ER membrane protein complex subunit 4
G1SVT0	TMEM205	Uncharacterized protein
G1THY3	UBE2V1	Ubiquitin conjugating enzyme E2 V1
G1TJP1	ATXN2	Uncharacterized protein
G1TX67	LDHA	L-lactate dehydrogenase
G1SHZ8	CCT8	Uncharacterized protein
G1SPN9	ARL3	Uncharacterized protein
G1T387	CTTN	Cortactin
G1TJG3		Uncharacterized protein
G1T127	KLKB1	Uncharacterized protein
G1TN25	AZGP1	Uncharacterized protein
G1T5N7	GRN	Uncharacterized protein

G1TGT5	HSD17B12	Uncharacterized protein
G1SCK0	PRPF8	Uncharacterized protein
G1SYE0	ABHD14B	Uncharacterized protein
G1SVN0	ENOSF1	Uncharacterized protein
G1T4Z7		Uncharacterized protein
G1TA37	BANF1	Uncharacterized protein
G1T235	PSMB6	Proteasome subunit beta
G1THZ6		Uncharacterized protein
G1TF67	CORO1A	Coronin
G1SDK8	NAE1	NEDD8-activating enzyme E1 regulatory subunit
G1T542	SNCG	Gamma-synuclein
G1SH95	CLIP1	CAP-Gly domain containing linker protein 1
G1U971	EIF3C	Eukaryotic translation initiation factor 3 subunit C
G1TB36	F2	Prothrombin
G1TL73	STAR	Steroidogenic acute regulatory protein, mitochondrial
G1SJQ2	GLG1	Uncharacterized protein
G1TX53	NDUFA8	NADH dehydrogenase [ubiquinone] 1 alpha subcomplex subunit 8
O46373	SLC25A4	ADP/ATP translocase 1
G1TDX2	AGK	Acylglycerol kinase
G1TJR3		Ribosomal protein L19
G1SK79	NID2	Uncharacterized protein
G1TV43	AKR1B10	Uncharacterized protein
G1SG31	NIPSNAP2	Nipsnap homolog 2
G1TQG1	NDUFB9	Uncharacterized protein
G1TB68	RABEPK	Uncharacterized protein
G1T4T3	BCKDHB	Uncharacterized protein
G1TUU7		Uncharacterized protein
G1T652	ALDOC	Fructose-bisphosphate aldolase
G1TDK5	PLEKHA7	Pleckstrin homology domain containing A7
G1T7Y7	CAPZB	F-actin-capping protein subunit beta
G1TM29	ALDH16A1	Aldehyde dehydrogenase family 16 member A1
G1SIF7	DCAF11	Uncharacterized protein
G1SZ14	PSMA3	Proteasome endopeptidase complex
G1SI71	PAIP1	Uncharacterized protein
G1T143	TRIM25	Uncharacterized protein
G1SPV2	YES1	Tyrosine-protein kinase
G1TUH9	DPYSL3	Uncharacterized protein
G1U5X6		2-oxoisovalerate dehydrogenase subunit alpha
G1SVE6	LSM12	Uncharacterized protein
G1T8N8	FXN	Uncharacterized protein
G1SSL2	CNPY2	Uncharacterized protein
G1STZ4	G6PD	Glucose-6-phosphate 1-dehydrogenase
G1T8T5	C1QB	Uncharacterized protein

G1TTJ1	DHRS11	Uncharacterized protein
G1T813	PDHA1	Pyruvate dehydrogenase E1 component subunit alpha
G1TQS9	RRBP1	Uncharacterized protein
G1T2A9	FLNC	Uncharacterized protein
G1U4Q4	PDE2A	Phosphodiesterase
G1TE64	PTBP3	Uncharacterized protein
G1TZ19	DIABLO	Uncharacterized protein
G1TE35	NUDT12	Uncharacterized protein
G1TIT1	ERLIN1	Uncharacterized protein
G1TBS2	RUVBL1	RuvB-like helicase
P00939	TPI1	Triosephosphate isomerase
O77819	ROCK1	Rho-associated protein kinase 1
G1SGS7	UFL1	Uncharacterized protein
G1SZM0	MIEN1	Uncharacterized protein
G1T489		Uncharacterized protein
G1SLF5	REXO2	Uncharacterized protein
G1SGL4	BCS1L	Uncharacterized protein
G1TEP2	FAM210B	Uncharacterized protein
G1SQH0	RPL26	Uncharacterized protein
G1TC03	TRIM28	Tripartite motif containing 28
G1TAQ9	ADAM10	Uncharacterized protein
U3KLX7	CUBN	Uncharacterized protein
G1SR03	VCP	Uncharacterized protein
G1T6S4	DST	Uncharacterized protein
G1SIM3	AIFM1	Uncharacterized protein
G1T060	HSD17B4	Uncharacterized protein
G1TLB2		Uncharacterized protein
G1TA01		Uncharacterized protein
G1TPY7	SUMF2	Uncharacterized protein
G1T894	BIN1	Uncharacterized protein
G1SI29	TUFM	Elongation factor Tu
G1T2H2	RBM47	Uncharacterized protein
G1SSE1	DIAPH2	Uncharacterized protein
G1SE28	RPL24	Uncharacterized protein
G1TJ20	MX1	Uncharacterized protein
G1SRE3	SLC23A2	Uncharacterized protein
G1TI68	EXOC1	Uncharacterized protein
G1SKD9	SUCLG1	Succinate--CoA ligase [ADP/GDP-forming] subunit alpha, mitochondrial
G1U9U0	CCT4	T-complex protein 1 subunit delta
G1T276	ALDH3A2	Aldehyde dehydrogenase
G1SQB1	MUT	Uncharacterized protein
G1T7D0	SRRM2	Serine/arginine repetitive matrix 2
G1TD16	PIP4K2A	Uncharacterized protein
G1TER3	BCAP31	Uncharacterized protein
G1T359	NDUFS1	Uncharacterized protein
G1TEA8	IMPDH2	Inosine-5'-monophosphate dehydrogenase
G1SZI5	RPS18	Uncharacterized protein
G1TX91	LZIC	Uncharacterized protein

G1SMA1	TMED10	Transmembrane emp24 domain-containing protein 10
G1SWN7	NADK2	NAD kinase 2, mitochondrial
G1T9V7	SRP19	Signal recognition particle 19
G1TJS2	PLD3	Phospholipase D family member 3
G1T2B8	ECE1	Uncharacterized protein
G1SY85	AP3M1	Uncharacterized protein
G1T512	CNP	2',3'-cyclic nucleotide 3' phosphodiesterase
G1TVW5	GMPPA	Uncharacterized protein
G1T398	CMPK1	UMP-CMP kinase
P26202		15 kDa protein A
G1ST81	HACD3	Very-long-chain (3R)-3-hydroxyacyl-CoA dehydratase
G1SIF2	PCK2	Uncharacterized protein
G1SJ46	CUTC	Uncharacterized protein
G1SKY7		Uncharacterized protein
G1SNT8	ANXA6	Annexin
G1SQF8	TRIM47	Uncharacterized protein
G1SZT8	SEC13	Uncharacterized protein
G1TLW0	AMDHD2	N-acetylglucosamine-6-phosphate deacetylase
G1T701	DBT	Dihydrolipoamide acetyltransferase component of pyruvate dehydrogenase complex
G1T139		Uncharacterized protein
B6S6L6	apoCI	Apolipoprotein C-I
Q9TTC6	PPIA	Peptidyl-prolyl cis-trans isomerase A
G1SJ74	HM13	Uncharacterized protein
G1T3D8	RPS24	40S ribosomal protein S24
G1SRI8	HK1	Uncharacterized protein
G1U8K3	CLPP	ATP-dependent Clp protease proteolytic subunit
G1SVM2	TMLHE	Uncharacterized protein
P02251		Histone H1.3
G1SR77	ATP2B4	Calcium-transporting ATPase
G1SQ27	CLU	Clusterin
G1TS38	CCDC90B	Uncharacterized protein
G1T024	PFAS	Uncharacterized protein
G1U8C4	PSME1	Uncharacterized protein
P35748	MYH11	Myosin-11
G1U9B4	TRA2B	Uncharacterized protein
P11974	PKM	Pyruvate kinase PKM
G1SP34	ARPC2	Arp2/3 complex 34 kDa subunit
G1U153		Uncharacterized protein
G1T2M0	KRAS	Uncharacterized protein
G1SUK4	MPI	Mannose-6-phosphate isomerase
G1T9T5	DLST	Uncharacterized protein

G1U711	TSC22D1	Uncharacterized protein
G1TXK8		Uncharacterized protein
G1T4D2	ACOX1	Acyl-coenzyme A oxidase
U3KM96	RAP1B	Uncharacterized protein
G1SHP3	CD36	Uncharacterized protein
G1SIN4	ERC1	Uncharacterized protein
G1SRX2	EFTUD2	Uncharacterized protein
G1U9R8	GSN	Uncharacterized protein
G1TB78	MT3	Metallothionein
G1TMM7	ANP32A	Uncharacterized protein
G1T837	PADI2	Uncharacterized protein
G1SQ70	A2M	Uncharacterized protein
G1TRK9		Uncharacterized protein
G1T2U1	PPAT	Amidophosphoribosyltransferas e
G1TDU0	KIF5B	Kinesin-like protein
G1SUE8	MAT2B	Methionine adenosyltransferase 2 subunit beta
G1TVP3	LOC100348853	Uncharacterized protein
G1SNP4	THRAP3	Uncharacterized protein
U3KN73	VPS29	Vacuolar protein sorting-associated protein 29
G1TM60	NDUFA9	Uncharacterized protein
U3KLU4		Uncharacterized protein
G1SKN0	LOC100347143	Uncharacterized protein
G1T6J2	APOO	MICOS complex subunit
O97529	ANXA8	Annexin A8
G1TT27	RPL8	Uncharacterized protein
G1U9R0	GSTP1	Uncharacterized protein
G1SLK2	PSMC5	Uncharacterized protein
G1SNC4	AK1	Adenylate kinase isoenzyme 1
G1T704	SORD	Uncharacterized protein
G1SKM5	NPLOC4	NPL4 homolog, ubiquitin recognition factor
G1SV13	UBA1	Ubiquitin-like modifier-activating enzyme 1
G1SH78	VKORC1	Uncharacterized protein
G1TUE1	ATP1B1	Sodium/potassium-transporting ATPase subunit beta
G1SXT7	P4HA1	Uncharacterized protein
G1SP27	CCDC6	Uncharacterized protein
G1TUY5	OCIAD1	Uncharacterized protein
G1U410	TUBB4A	Tubulin beta chain
G1SD89	COL6A2	Uncharacterized protein
G1SZW8	EPS15L1	Epidermal growth factor receptor pathway substrate 15 like 1
G1SMH6	H2AFY2	Core histone macro-H2A
G1T0L9	RPN1	Dolichyl-diphosphooligosaccharide--protein glycosyltransferase subunit 1
G1U502	FAM185A	Uncharacterized protein
G1SGJ7	PTER	Uncharacterized protein
G1TFE0	RPL18	Uncharacterized protein

G1SD77	ARMC1	Armadillo repeat-containing protein 1
G1SY50	NDUFV2	Uncharacterized protein
G1SDD0	ECHS1	Uncharacterized protein
G1T0N5	PURA	Uncharacterized protein
G1SIH1	PSME2	Uncharacterized protein
G1TPW2	MRI1	Methylthioribose-1-phosphate isomerase
G1SCF4	DNM1L	Uncharacterized protein
G1SSX2	HUWE1	Uncharacterized protein
G1T7H0	HNRNPU	Uncharacterized protein
G1SV05	HSPA4	Uncharacterized protein
G1U7C6	HIST1H1D	Uncharacterized protein
G1TCY4	SNX5	Sorting nexin
P07952	BPGM	Bisphosphoglycerate mutase
G1TDQ5	PFKM	ATP-dependent 6-phosphofructokinase
G1SUE4	CST3	Cystatin
G1SM62	PAPSS1	Uncharacterized protein
G1SYI3	USP39	Uncharacterized protein
G1SFW9	ACIN1	Uncharacterized protein
G1SJ56	VCL	Uncharacterized protein
G1SZ76	SSB	Lupus La protein homolog
G1TBS8	CFH	Uncharacterized protein
P83468	PHPT1	14 kDa phosphohistidine phosphatase
G1SEA8	GPHN	Molybdopterin molybdenumtransferase
G1SW06	LOC100344160	Uncharacterized protein
G1SES8	MRPS22	Uncharacterized protein
G1SUU7	RTCB	tRNA-splicing ligase RtcB homolog
U3KMN4	RPS5	Uncharacterized protein
G1TE13	RHOG	Uncharacterized protein
G1T6N3	NPC2	Uncharacterized protein
G1SN21	PNP	Purine nucleoside phosphorylase
G1U5I0	MTCH2	Uncharacterized protein
G1SXL9	XPNPEP3	Uncharacterized protein
G1SPE6	LOC100343250	Signal peptidase complex subunit 3
G1TLL7	TMX4	Uncharacterized protein
G1TCM9	SH3BGRL3	SH3 domain-binding glutamic acid-rich-like protein
B7NZM8	YWHAH	Tyrosine 3-monooxygenase/tryptophan 5-monooxygenase activation protein, eta polypeptide (Predicted)
G1SH05	TUBB	Tubulin beta chain
G1TKS5	OCIAD2	Uncharacterized protein
G1TLQ8	PSMC3	Uncharacterized protein
U3KLT3		Uncharacterized protein
G1SSG2	VPS4B	Uncharacterized protein
G1SUF4	SPTLC1	Uncharacterized protein
G1SPD1	MTPN	Uncharacterized protein

G1SLM0	PABPC1	Polyadenylate-binding protein
P42675	NLN	Neurolysin, mitochondrial
G1U9C1	DNM2	Dynamin 2
G1SJR4	TMED2	Transmembrane p24 trafficking protein 2
Q6TYA7	GJA1	Gap junction alpha-1 protein
G1STU0	WDR77	Uncharacterized protein
G1SQK3	NUDT2	Uncharacterized protein
G1TKL0	SNRPA	Small nuclear ribonucleoprotein polypeptide A
G1STI3	KHDRBS1	Uncharacterized protein
G1TH03	ATP2A2	Calcium-transporting ATPase
G1U5M7	SNU13	Uncharacterized protein
G1TAB2	GM2A	Uncharacterized protein
G1SLU3	LIMS1	LIM and senescent cell antigen-like-containing domain protein
G1T810	CUL2	Uncharacterized protein
G1U4G9	CLIC1	Chloride intracellular channel protein
G1U9S2	ALB	Serum albumin
G1TDN3	SNRPA1	Uncharacterized protein
G1SRA8	EIF2S3	Eukaryotic translation initiation factor 2 subunit 3
P40826	USP14	Ubiquitin carboxyl-terminal hydrolase 14
G1U8J5	ATP5PF	ATP synthase-coupling factor 6, mitochondrial
G1SWC0	HGD	Uncharacterized protein
G1TA95	PRPSAP2	Phosphoribosyl pyrophosphate synthetase associated protein 2
G1SRY1	PNPT1	Uncharacterized protein
G1SQ11	ALDH18A1	Delta-1-pyrroline-5-carboxylate synthase
G1SGB5	SFPQ	Splicing factor proline and glutamine rich
G1SQ90	COL4A3BP	Collagen type IV alpha 3 binding protein
G1T338	PPIF	Peptidyl-prolyl cis-trans isomerase
G1T6X7	LOC100351739	Carboxylic ester hydrolase
G1SZB1	TTC19	Uncharacterized protein
G1TQZ7	GYG1	Glycogenin-1
G1SV22	PEBP1	Phosphatidylethanolamine-binding protein 1
G1SK00	USP5	Ubiquitinyl hydrolase 1
G1TPN2	CDC37	Cell division cycle 37
G1TTS1	FUNDC2	FUN14 domain containing 2
G1TP36		Uncharacterized protein
G1TVX2	ACAD11	Uncharacterized protein
G1T7L5	IMPA1	Inositol-1-monophosphatase
G1TAB7	OPA1	Uncharacterized protein

G1T4Q8	PARVA	Uncharacterized protein
G1TDQ2		Prefoldin subunit 3
G1TBL6	CLTC	Clathrin heavy chain
G1TH33	ETFB	Electron transfer flavoprotein subunit beta
G1SIE8	IDE	Uncharacterized protein
G1TA04	PRPF19	Uncharacterized protein
G1U6M8		Uncharacterized protein
G1SGI6	TNS1	Uncharacterized protein
G1TCR3	PSMG3	Uncharacterized protein
G1TAK1	COA3	Uncharacterized protein
G1T642	CMC2	COX assembly mitochondrial protein
G1SFV1	PDIA4	Protein disulfide-isomerase
G1SYK4	LOC100346672	Uncharacterized protein
G1SFS8	SND1	Staphylococcal nuclease domain-containing protein
G1TDR3	EPB42	Uncharacterized protein
G1SNZ3	CSE1L	Chromosome segregation 1 like
U3KNU8	LOC100347914	Uncharacterized protein
G1SF78	VPS13A	Uncharacterized protein
G1SFP0	LAP3	Uncharacterized protein
G1SPB8	NAP1L4	Uncharacterized protein
G1U6Q9	SNRPC	U1 small nuclear ribonucleoprotein C
G1SP30	FKBP5	Peptidylprolyl isomerase
G1TKT9	PPCS	Uncharacterized protein
G1TDM9		Uncharacterized protein
G1U5L3	LMAN1	Uncharacterized protein
G1SE12	ETFA	Electron transfer flavoprotein subunit alpha
U3KNR7	NPC1	Uncharacterized protein
G1U5Q7	ARPC4	Actin-related protein 2/3 complex subunit 4
G1SE95		Uncharacterized protein
G1TDB3	RPS25	Uncharacterized protein
G1T5V8	LYPLA2	Uncharacterized protein
G1U4E6	LOC100358336	Uncharacterized protein
G1SHF1	PFDN2	Uncharacterized protein
G1SKK1	DUT	Uncharacterized protein
G1SK25	PRKACB	Uncharacterized protein
P02057	HBB1	Hemoglobin subunit beta-1/2
G1SKV7	CNDP2	Uncharacterized protein
G1ST52		Uncharacterized protein
Q01059	ACO1	Cytoplasmic aconitate hydratase
G1U685	CHGB	Uncharacterized protein
O19049	HNRNPK	Heterogeneous nuclear ribonucleoprotein K
G1TBD3	PRKAB1	Uncharacterized protein
G1T9D7	GRAMD1C	Uncharacterized protein
G1SKQ9	6-Sep	Uncharacterized protein
G1TEU8	TBCC	Uncharacterized protein
G1SGX4	RPS16	Uncharacterized protein
G1U8R2		Uncharacterized protein
G1TL06	RPL3	Uncharacterized protein

G1TFZ5	FLOT2	Uncharacterized protein
G1SRH7	SRSF3	Uncharacterized protein
G1SLD5	HYOU1	Hypoxia up-regulated 1
G1SJI4		Uncharacterized protein
Q8MI17	ALDH1A1	Retinal dehydrogenase 1
G1TBY1	CTSB	Uncharacterized protein
G1TX63	CDC42BPA	Non-specific serine/threonine protein kinase
G1TA53	ACSS3	Uncharacterized protein
G1T0F6	SRP68	Signal recognition particle subunit SRP68
G1TLZ2	VAMP2	Uncharacterized protein
G1TC33	MYH10	Uncharacterized protein
G1T7D3	MPZ	Uncharacterized protein
G1SWU1	OXCT1	Succinyl-CoA:3-ketoacid-coenzyme A transferase
G1U9J9	UBL5	Uncharacterized protein
G1SIJ6	PTK7	Uncharacterized protein
G1TJB7	ACSBG1	Acyl-CoA synthetase bubblegum family member 1
G1SN26	FKBP15	Peptidylprolyl isomerase
G1TVT1	ADD1	Adducin 1
G1TRH5	CORO1C	Coronin
G1TG89	RPS15A	Uncharacterized protein
G1T4Y7		Uncharacterized protein
G1SGV5	ABCF1	Uncharacterized protein
G1SX73	PFDN1	Uncharacterized protein
G1U7Q6	TSN	Uncharacterized protein
G1TEE4	PIR	Uncharacterized protein
G1U450	XYLB	Xylulokinase
G1SQ02	PRDX1	Uncharacterized protein
U3KPC4	MAVS	Uncharacterized protein
G1SL85	ASAH1	Uncharacterized protein
G1U5W5	NRDC	Uncharacterized protein
Q8SQG9	SLC9A3R2	Na(+)/H(+) exchange regulatory cofactor NHE-RF2
P46409		Glutathione S-transferase Mu 1
G1T8W7	ATP6V0A1	V-type proton ATPase subunit a
G1SEX0	HACD2	Very-long-chain (3R)-3-hydroxyacyl-CoA dehydratase
G1T5Z7	MAPK3	Mitogen-activated protein kinase
G1T2V2	PAICS	Uncharacterized protein
G1SE76	LOC108177184	Uncharacterized protein
G1SRF7	HSPA9	Uncharacterized protein
G1TLG5	LOC100346274	Uncharacterized protein
P11909	GPX1	Glutathione peroxidase 1
G1SYB4	CDC42	Uncharacterized protein
G1SUU2	ASNA1	ATPase ASNA1
G1TB05		Uncharacterized protein
G1T1P3	ACAD9	Uncharacterized protein
G1TM88	SERPINA3	Uncharacterized protein

G1SVB6	GLUD1	Glutamate dehydrogenase
G1TI64		Uncharacterized protein
G1SF30		Ribosomal protein L15
G1TA11	RARS	Uncharacterized protein
G1TPR9	PM20D2	Peptidase M20 domain-containing protein 2
G1SI85	SFXN3	Uncharacterized protein
G1T134	ABHD11	Abhydrolase domain containing 11
G1T437	PPT1	Uncharacterized protein
G1U248	HNRNPH1	Uncharacterized protein
G1ST99	AMBP	Alpha-1-microglobulin/bikunin precursor
G1T5J9	NDUFS2	Uncharacterized protein
G1SMT7	NHLRC2	Uncharacterized protein
G1SL57	7-Sep	Uncharacterized protein
G1TG27	RPE	Ribulose-phosphate 3-epimerase
G1T983	HEBP1	Uncharacterized protein
G1TI10	STT3B	Uncharacterized protein
G1TPZ1	LGALS1	Galectin
G1TTY7	RPL18A	60S ribosomal protein L18a
G1TPI0	FOLH1	Uncharacterized protein
G1U6T0	F5	Uncharacterized protein
G1STH6	DEPTOR	Uncharacterized protein
G1SSA2	PSMD2	26S proteasome non-ATPase regulatory subunit 2
G1T933	COL14A1	Uncharacterized protein
G1T9S4	DLAT	Acetyltransferase component of pyruvate dehydrogenase complex
G1T0W7	EMC8	ER membrane protein complex subunit 8
G1SJ41	SEC23IP	Uncharacterized protein
Q28719	PTGR1	Prostaglandin reductase 1
G1SJZ4	OLA1	Obg-like ATPase 1
G1T4P8	GLRX3	Glutaredoxin 3
G1SFV7	DDB1	Damage specific DNA binding protein 1
G1T567	RHOA	Uncharacterized protein
G1SYJ4	ENO1	Uncharacterized protein
G1SVQ8	SEC62	Uncharacterized protein
G1SR20	GALK2	Uncharacterized protein
G1TGI6	RPSA	40S ribosomal protein SA
A0A0A0MQQ6	EIF4E	Eukaryotic translation initiation factor 4E
G1T2M9	SPARC	SPARC
G1T587	CCDC134	Uncharacterized protein
G1T156	SCPEP1	Carboxypeptidase
G1T9M9	HSPA8	Uncharacterized protein
Q28740	BSG	Basigin
G1SV03	MLEC	Uncharacterized protein
G1TMC5	LOC100357214	Carboxylic ester hydrolase
U3KNQ3	TACC1	Uncharacterized protein
U3KPG6	ICAM1	Intercellular adhesion molecule 1

Q9TT15	VDAC1	Voltage-dependent anion-selective channel protein 1
G1T6M1	DNAJC19	Uncharacterized protein
G1U0Q7	2-Sep	Uncharacterized protein
G1SHV1	LSM2	U6 snRNA-associated Sm-like protein LSm2
G1SIP1	DHRS1	Uncharacterized protein
G1TRJ6	GPD1L	Glycerol-3-phosphate dehydrogenase [NAD(+)]
G1SYV0	PSMC2	Uncharacterized protein
G1TG66		Uncharacterized protein
G1T6Q9	STX7	Uncharacterized protein
G1T3A6	ME2	Malic enzyme
G1U448	FUBP1	Uncharacterized protein
G1T9Q3	KCNJ5	Uncharacterized protein
G1T013	ME1	Malic enzyme
G1T4Z0	FMNL2	Uncharacterized protein
G1T1W6	AHCYL2	Adenosylhomocysteinase
G1T4S5	CMAS	Uncharacterized protein
G1TLE3	LOC100348124	Phosphoglycerate kinase
G1SLD1	NDUFA2	NADH dehydrogenase [ubiquinone] 1 alpha subcomplex subunit 2
G1SNV4	SF3A3	Uncharacterized protein
G1SD49	DCTN3	Uncharacterized protein
G1TET3	MAIP1	Uncharacterized protein
G1SR53	FUCA1	Alpha-L-fucosidase
G1T726	HADH	Uncharacterized protein
G1TZ26	GUK1	Guanylate kinase 1
G1SST9	RAD23B	Uncharacterized protein
G1SIY9	ALDH5A1	Succinate-semialdehyde dehydrogenase
G1U460	ALDH6A1	Uncharacterized protein
G1T974	MOGS	Uncharacterized protein
G1SVB0	RPS7	40S ribosomal protein S7
G1T9N2	ATP5PD	ATP synthase subunit d, mitochondrial
G1TPE4		Uncharacterized protein
G1SZ72	FXR2	Uncharacterized protein
G1TC70	PPP1R12A	Uncharacterized protein
G1T0W8	FGB	Fibrinogen beta chain
G1TE20	ERMP1	Uncharacterized protein
G1SX00	PSIP1	Uncharacterized protein
G1SW57	NT5E	Uncharacterized protein
G1U120	CYBC1	Cytochrome b-245 chaperone 1
P98049	MT-CO2	Cytochrome c oxidase subunit 2
G1T6I0	AOX1	Aldehyde oxidase 1
G1T4H0	TMOD3	Uncharacterized protein
P62943	FKBP1A	Peptidyl-prolyl cis-trans isomerase FKBP1A
G1TDI0	PA2G4	Uncharacterized protein
G1SSP0	STRN	Uncharacterized protein
G1SSB4		Uncharacterized protein
G1T7P9	SNRNP40	Uncharacterized protein
G1SYD3	YARS	Tyrosine--tRNA ligase
G1SVZ8	C9orf64	Queuosine salvage protein

G1SM52	LRRC59	Uncharacterized protein
G1T9I4	SRI	Uncharacterized protein
G1SM64	ITIH2	Uncharacterized protein
G1SXW0	GPD2	Glycerol-3-phosphate dehydrogenase
G1T643	UNC45A	Unc-45 myosin chaperone A
G1TCP3	ALDH1A2	Uncharacterized protein
G1TXI0	FKBP11	Peptidylprolyl isomerase
G1TCS8	RAB1A	Uncharacterized protein
A0A0B4J1Q3		Uncharacterized protein
G1TI55	TIMM9	Uncharacterized protein
G1SPZ7	GPX1	Glutathione peroxidase
G1T6E8	TSG101	Uncharacterized protein
G1ST49	TCEA1	Uncharacterized protein
G1THY2	CLNS1A	Methylosome subunit pICln
O77622	CCT6	T-complex protein 1 subunit zeta
G1SQM7	SUB1	Uncharacterized protein
G1TT75	PGRMC1	Uncharacterized protein
G1T5D3	NAMPT	Nicotinamide phosphoribosyltransferase
G1SH81		Uncharacterized protein
G1TEI2	FDX1	Uncharacterized protein
P53787	EEF1D	Elongation factor 1-delta
G1SNQ9	NCEH1	Uncharacterized protein
G1SDA4	COPB1	Coatomer subunit beta
P27124	FKBP4	Peptidyl-prolyl cis-trans isomerase FKBP4
G1TV92	TNKS1BP1	Uncharacterized protein
P41975	SOD3	Extracellular superoxide dismutase [Cu-Zn]
G1SZL3	ABAT	Uncharacterized protein
G1TP25	UBAP2L	Uncharacterized protein
G1TDK8	SMARCC2	Uncharacterized protein
G1T0C5	DCTN6	Uncharacterized protein
P01696		Ig kappa chain V region K29-213
G1THH9	RABEP2	Uncharacterized protein
G1SNC7	DPT	Uncharacterized protein
G1TGA8	SEC22B	Uncharacterized protein
G1T3A2	TMED5	Uncharacterized protein
G1T2T9	RAB5A	Uncharacterized protein
G1T3E2	TM9SF3	Transmembrane 9 superfamily member
G1SIG2	CRK	Uncharacterized protein
G1U7L1	LOC100358162	Uncharacterized protein
G1T2Y5	TPR	Uncharacterized protein
G1TBU8	RAP1GDS1	Uncharacterized protein
G1SGY8	TIMM50	Mitochondrial import inner membrane translocase subunit TIM50
G1T8H3	GALK1	Uncharacterized protein
G1U9S7	TCP1	Uncharacterized protein
G1SE57	ARF4	Uncharacterized protein
G1TLD3	NUCB1	Nucleobindin 1
P67873	CSNK2B	Casein kinase II subunit beta

G1TXK3	AAMDC	Uncharacterized protein
G1TDJ3	ESYT1	Uncharacterized protein
G1SZH0	RBP4	Retinol-binding protein
G1SQS9	DECR1	Uncharacterized protein
P14519	SHMT2	Serine hydroxymethyltransferase, mitochondrial
G1T6I6	ILF3	Interleukin enhancer binding factor 3
Q08863		Glutathione S-transferase alpha I
G1SL49	LOC100346986	Uncharacterized protein
G1SX88	ARHGAP18	Uncharacterized protein
G1SFG6	GPI	Glucose-6-phosphate isomerase
G1TVH9	ARPC1B	Actin-related protein 2/3 complex subunit
G1TRY5	PLS3	Uncharacterized protein
G1SKZ8	RPL10A	Ribosomal protein
G1SSH5	LOC100357419	Uncharacterized protein
G1TGH1	D2HGDH	D-2-hydroxyglutarate dehydrogenase
P63150	PPP2R2A	Serine/threonine-protein phosphatase 2A 55 kDa regulatory subunit B alpha isoform
G1SPU6		Uncharacterized protein
G1SZ37	HIBADH	3-hydroxyisobutyrate dehydrogenase
G1TAM3	TBCB	Uncharacterized protein
G1T2V4		Uncharacterized protein
U3KNE2	NDUFA4	Uncharacterized protein
G1U636	CAPRIN1	Uncharacterized protein
G1SXH7	SRC	Tyrosine-protein kinase
G1TTM0	STMN2	Stathmin
G1TDH4	PRDX3	Uncharacterized protein
Q9N0Z6	ATP1A1	Sodium/potassium-transporting ATPase subunit alpha-1
G1TMB7	CYP2F1	Cytochrome P450 family 2 subfamily F member 1
G1SEN8	SCCPDH	Uncharacterized protein
G1SRS3	RAB4A	Uncharacterized protein
G1T168	RPS10	Uncharacterized protein
Q9TT13	VDAC3	Voltage-dependent anion-selective channel protein 3
G1U3X5	MAP1B	Uncharacterized protein
G1U5Y9	NAV1	Uncharacterized protein
G1TZX4	HSD17B8	Uncharacterized protein
G1TWP4	VARS	Uncharacterized protein
G1U949	MAPRE1	Uncharacterized protein
P43236	CTSK	Cathepsin K
P01840		Ig kappa-b4 chain C region
G1T8E0	VWA8	Uncharacterized protein
G1T524	SLC25A5	Uncharacterized protein
G1SM51	PSMD13	Proteasome 26S subunit, non-ATPase 13
G1TV19	FUS	FUS RNA binding protein

G1T6S0	TPM1	Tropomyosin alpha-1 chain
G1SRT1	APPL1	Uncharacterized protein
P00169	CYB5A	Cytochrome b5
G1SZS0	NIF3L1	NIF3-like protein 1
Q29502	PAK2	Serine/threonine-protein kinase PAK 2
G1T3Y0	DAB2	Uncharacterized protein
G1TNY1	TIMM8A	Uncharacterized protein
G1TF82	LOC100353962	Uncharacterized protein
G1SLJ9	EMC3	ER membrane protein complex subunit 3
G1SL28	FITM2	Uncharacterized protein
G1T5J6	MGST1	Uncharacterized protein
G1SQ22	COPG1	Coatomer subunit gamma
G1T3V0	HDLBP	Uncharacterized protein
G1SNH7	QDPR	Quinoid dihydropteridine reductase
G1T6B3	STRAP	Uncharacterized protein
G1SV12	ACADS	Uncharacterized protein
G1SMY6	XPO1	Uncharacterized protein
G1SPM5	ACTR1A	Uncharacterized protein
G1TTU6	SKP1	Uncharacterized protein
G1TJW1	LOC100352057	40S ribosomal protein S8
G1T0R9	LOC100357917	Glutathione S-transferase
G1SN14	CAND1	Uncharacterized protein
G1TP30	RANGAP1	Uncharacterized protein
G1TK30	CSAD	Uncharacterized protein
G1U2E6	PHB2	Uncharacterized protein
G1STU7	ATP5PB	Uncharacterized protein
G1T7F1	LOC100348835	Histone H2B
G1T9I9	FABP4	Uncharacterized protein
G1TIR7	LSM8	U6 snRNA-associated Sm-like protein LSm8
G1T657	HNRNPDL	Uncharacterized protein
G1U018	IGF2R	Uncharacterized protein
U3KNL5	EIF3F	Eukaryotic translation initiation factor 3 subunit F
G1TA21	SCO1	Uncharacterized protein
G1T2W1	SRP72	Signal recognition particle subunit SRP72
G1SG29		Uncharacterized protein
G1TFE8	LOC100346996	40S ribosomal protein S26
G1T040	LOC100353469	Uncharacterized protein
G1SRB6	PPID	Peptidylprolyl isomerase D
G1SW65	ATP5MC2	Uncharacterized protein
G1SVU0	MTX2	Uncharacterized protein
G1TD51	HIST4H4	Histone H4
G1SV24	LOC100008830	Uncharacterized protein
G1T461	C1orf123	Uncharacterized protein
G1SPG6	PIGK	GPI-anchor transamidase
G1SK33	ITGB1	Integrin beta
G1T0I5	PITRM1	Uncharacterized protein
G1SMY1	DDX17	Uncharacterized protein
G1SY94	PPM1A	Protein phosphatase 1A
G1SNT1	EIF3J	Eukaryotic translation initiation factor 3 subunit J
G1TVH4		Uncharacterized protein
G1U0Z7	CENPV	Uncharacterized protein

G1SHF3	NIT1	Uncharacterized protein
G1TJV3	PTGES2	Prostaglandin E synthase 2
G1SUM3	UBA3	Uncharacterized protein
G1TRG9		Uncharacterized protein
G1U0U5	HSPA4L	Uncharacterized protein
G1T443	C11orf54	Uncharacterized protein
U3KNY1	ASPH	Aspartate beta-hydroxylase
G1ST51	CLIC2	Chloride intracellular channel protein
G1SUH5	UQCRB	Cytochrome b-c1 complex subunit 7
G1SRL3	NMT1	Glycylpeptide N-tetradecanoyltransferase
G1SPY9	ACSM5	Uncharacterized protein
G1ST95	EIF3H	Eukaryotic translation initiation factor 3 subunit H
G1TW43	TNC	Uncharacterized protein
G1SQV5	EIF3D	Eukaryotic translation initiation factor 3 subunit D
G1TIZ5	TXNDC5	Uncharacterized protein
P12822	ACE	Angiotensin-converting enzyme
G1SP51	RPS13	Uncharacterized protein
G1TZJ6		Uncharacterized protein
G1TE69	SRSF1	Uncharacterized protein
G1STR7	SPTA1	Uncharacterized protein
G1SN09	GMPPB	Uncharacterized protein
G1T8D4	SEC31A	Uncharacterized protein
G1SV75	NDUFAB1	Acyl carrier protein
G1THV8	CNPY3	Uncharacterized protein
G1SS66	C2	Uncharacterized protein
G1U9T1	CCT7	T-complex protein 1 subunit eta
G1T5K3	APOA2	Uncharacterized protein
G1SLX6	PYCARD	Uncharacterized protein
G1SNE3	DHCR24	Uncharacterized protein
G1T8R2	ABCB6	Uncharacterized protein
G1SIX1	SLK	Uncharacterized protein
G1SHI9	OGDH	Uncharacterized protein
G1U754	HRG	Histidine-rich glycoprotein
G1TE43	VPS4A	Uncharacterized protein
G1T4P7	TLN2	Uncharacterized protein
G1SU97	PDHX	Dihydrolipoamide acetyltransferase component of pyruvate dehydrogenase complex
G1T647	GCLM	Uncharacterized protein
G1TAD3	C3H5orf63	Glutaredoxin-like protein
G1T3U1	GORASP2	Uncharacterized protein
G1SE27	NDUFS4	Uncharacterized protein
G1SJU8	FADS2	Uncharacterized protein
G1SML9	DNAJA1	Uncharacterized protein
G1TSU1	PTGES	Prostaglandin E synthase
G1T4S6	ACSS1	Acetyl-coenzyme A synthetase
G1T0Q0		Uncharacterized protein
G1SQF2	DYNC1LI2	Uncharacterized protein
G1U7P6	AAMP	Uncharacterized protein

G1U0V2	SNCA	Alpha-synuclein
G1T8H8	ABCB1	Uncharacterized protein
G1SVA9	CD300LG	Uncharacterized protein
G1SV99	TES	Testin
G1SIJ2	ACAT1	Uncharacterized protein
G1SSJ8	TET1	Uncharacterized protein
G1TKH3	SOD1	Superoxide dismutase [Cu-Zn]
G1SI22	AKAP12	A-kinase anchoring protein 12
G1SI37	ACSF2	Uncharacterized protein
G1TFV7	LOC100008973	Uncharacterized protein
G1SLM1	CACYBP	Uncharacterized protein
G1T3N8	AIMP2	Uncharacterized protein
G1TJW8	PLPBP	Pyridoxal phosphate homeostasis protein
Q8HZQ5	EZR	Ezrin
G1SMG5	GSPT1	Uncharacterized protein
G1SU30	BAG6	Uncharacterized protein
G1U276	SUCLA2	Succinate--CoA ligase [ADP-forming] subunit beta, mitochondrial
G1T501	CCAR2	Cell cycle and apoptosis regulator 2
G1TDI4	TTR	Transthyretin
G1STW2	ERAP1	Aminopeptidase
G1TQP6		Uncharacterized protein
G1ST64	LMOD1	Uncharacterized protein
G1SWM7	ECHDC3	Uncharacterized protein
G1SKS0	ARPC1A	Actin-related protein 2/3 complex subunit
G1SH86	TBCD	Tubulin folding cofactor D
G1U5B3	PSAP	Uncharacterized protein
G1T594	STIM1	Uncharacterized protein
U3KND5	ABHD12	Abhydrolase domain containing 12
G1TRM4	LOC100343123	Uncharacterized protein
G1SN83	LAMB2	Uncharacterized protein
G1T3I9	ANXA7	Annexin
G1T169	ITGA1	Uncharacterized protein
G1TM82	RPS21	40S ribosomal protein S21
G1T5Q8	STX12	Uncharacterized protein
G1SXQ0	GSTM3	Glutathione S-transferase
G1U1Q8	MIF	Uncharacterized protein
G1SXR1	PRELP	Uncharacterized protein
G1SJW7	SNRPN	Small nuclear ribonucleoprotein-associated protein
G1T373	PRPS1	Uncharacterized protein
G1T9R5	MXRA7	Matrix remodeling associated 7
G1SHV9	PSMB3	Proteasome subunit beta
G1SRB7	TPP1	Uncharacterized protein
G1TDN4	PRKAR1A	Uncharacterized protein
G1U5N5		Uncharacterized protein
G1SKM2	FBN1	Uncharacterized protein
G1SI79	HNRNPA3	Uncharacterized protein
G1T5X6	HMGCL	Uncharacterized protein

G1T521		Uncharacterized protein
G1SNB5	FDFT1	Uncharacterized protein
G1T2C4	TAGLN	Transgelin
G1T310	PRXL2A	Uncharacterized protein
G1U541	LOC100344983	Coatomer subunit gamma
G1SUY8	ADHFE1	Uncharacterized protein
G1SRB1	ETFDH	Uncharacterized protein
G1TA50	UBA5	Uncharacterized protein
G1T520	DNAJC7	Uncharacterized protein
G1SKK9	SON	SON DNA binding protein
G1SDU6	TARS	Uncharacterized protein
G1TTK6	PCCB	Uncharacterized protein
G1TYN0	TMED9	Uncharacterized protein
G1TAA4	ZMPSTE24	CAAX prenyl protease
G1TT06	SELENBP1	Uncharacterized protein
G1T5T8	PDLIM1	Uncharacterized protein
G1SWK5	PIGT	Uncharacterized protein
G1SHK8	GLS	Uncharacterized protein
Q1XH18	TRIM72	Tripartite motif-containing protein 72
P30946	HSP90AA1	Heat shock protein HSP 90-alpha
G1SIT9	YWHAB	Uncharacterized protein
G1TCE9	HSDL2	Uncharacterized protein
G1SN68	QARS	Uncharacterized protein
G1SUD1	MCEE	Methylmalonyl-CoA epimerase
G1SQD1	EIF4G1	Eukaryotic translation initiation factor 4 gamma 1
G1SS07	UBE2V2	Uncharacterized protein
G1SV04	NDUFB11	Uncharacterized protein
G1SKJ4	MRPL1	Uncharacterized protein
G1SL16	SRSF7	Uncharacterized protein
G1SNE8	S100A11	Protein S100
G1U4R5	DDOST	Dolichyl-diphosphooligosaccharide--protein glycosyltransferase 48 kDa subunit
G1TQX1	SRP14	Uncharacterized protein
G1TIB4	RPS28	Ribosomal protein S28
G1SRE6	EPB41	Uncharacterized protein
P47845	LGALS3	Galectin-3
G1T8Y0	COASY	Uncharacterized protein
G1T6W7	CAT	Catalase
G1TJG6		Glyceraldehyde-3-phosphate dehydrogenase
G1SCT9	MAGT1	Uncharacterized protein
G1SKW5		Uncharacterized protein
G1SVH8	RAB31	Uncharacterized protein
G1SQU0	GNS	N-acetylglucosamine-6-sulfatase
G1TMQ4	GOPC	Uncharacterized protein
G1SQS1	GALM	Aldose 1-epimerase
G1U2A1		Uncharacterized protein
G1SQF9	PSPC1	Uncharacterized protein
G1TNH9		Uncharacterized protein
P47814	EIF1A	Eukaryotic translation initiation factor 1A

G1STS9	SPRYD4	Uncharacterized protein
G1T4X4	SULT1C4	Sulfotransferase
G1TWM4	DHRS7B	Dehydrogenase/reductase 7B
U3KNR1	USP7	Uncharacterized protein
G1T3Y8	HSPD1	Uncharacterized protein
G1U6N2	LOC100358778	60S acidic ribosomal protein P0
G1TQ31	CSNK2A1	Casein kinase II subunit alpha
G1T277	ARPC3	Actin-related protein 2/3 complex subunit 3
G1SEC9	GNB4	Uncharacterized protein
G1SY53	RPL37A	Uncharacterized protein
G1TSN0	CHGA	Uncharacterized protein
U3KNG6	PFKP	ATP-dependent 6-phosphofructokinase
G1TJP8	NUCKS1	Uncharacterized protein
G1SZ12	RPL14	Uncharacterized protein
G1U3M3		Tubulin-specific chaperone A
G1SYA5	ESD	S-formylglutathione hydrolase
A7X8X3	HPRT	Hypoxanthine phosphoribosyltransferase
G1TU12	MAGED2	Uncharacterized protein
G1SP11	PRKRA	Uncharacterized protein
G1T8V6	HMBS	Hydroxymethylbilane synthase
G1TSP3	TECR	Uncharacterized protein
G1SZ47	RPS23	Uncharacterized protein
G1TR31	LOC100353846	Uncharacterized protein
G1TBW1	TXNDC17	Uncharacterized protein
G1TCZ8	PPP2R1A	Protein phosphatase 2 scaffold subunit Aalpha
B7NZM0	APOA4	Apolipoprotein A-IV (Predicted)
G1SIN7	RABGGTA	Uncharacterized protein
G1TM95	LOC100358239	Uncharacterized protein
G1SS49	HDHD2	Uncharacterized protein
G1TN62	LOC100345774	Uncharacterized protein
G1U0J7		Uncharacterized protein
G1SXD6		Uncharacterized protein
G1TCW5	PEPD	Uncharacterized protein
G1TA78	WARS	Tryptophan--tRNA ligase, cytoplasmic
G1SVW5	RPL4	Uncharacterized protein
G1U0H7	SH3GL3	SH3 domain containing GRB2 like 3, endophilin A3
G1SSK8	CS	Citrate synthase
G1TIZ1	UCHL1	Ubiquitin carboxyl-terminal hydrolase
G1SUX8	LOC100354435	Uncharacterized protein
U3KNB6	ARCN1	Coatomer subunit delta
G1SIW1	DHX15	Uncharacterized protein
G1SFJ9	CIAO1	Probable cytosolic iron-sulfur protein assembly protein CIAO1

G1SQ38	DNM3	Uncharacterized protein
G1SIZ2	LOC100354063	Uncharacterized protein
G1U522	PRKAR2A	Uncharacterized protein
G1T846	DARS	Uncharacterized protein
G1SYM2	STXBP2	Syntaxin binding protein 2
G1SXU2	PDLIM5	Uncharacterized protein
G1SRP8	PTGR2	Uncharacterized protein
G1SQP0	RAB13	Uncharacterized protein
G1U6H6	FHL1	Uncharacterized protein
G1TIC9	TST	Sulfurtransferase
G1SEV2	PDIA3	Protein disulfide-isomerase
U3KPE6	CCDC91	Uncharacterized protein
G1TT64	ARHGAP17	Uncharacterized protein
G1SDM2	RDH11	Uncharacterized protein
G1U6X4	METAP2	Methionine aminopeptidase 2
G1U8Z2	CLPB	Uncharacterized protein
G1T8C8	SNX12	Uncharacterized protein
G1T8D7	AEBP1	Uncharacterized protein
G1SGE9	LDHD	Uncharacterized protein
G1SM27	NDUFS6	NADH dehydrogenase [ubiquinone] iron-sulfur protein 6, mitochondrial
G1T5V3	RAB14	Uncharacterized protein
G1T7M3	TIPRL	Uncharacterized protein
G1T8P3	SNRNP70	Small nuclear ribonucleoprotein U1 subunit 70
P12345	GOT2	Aspartate aminotransferase, mitochondrial
G1SD44	MTX1	Uncharacterized protein
G1SDY5	YWHAZ	Uncharacterized protein
G1SQ10	KIF13B	Uncharacterized protein
G1TD99	PCCA	Uncharacterized protein
G1TVT0	NCL	Uncharacterized protein
G1TKC4	KARS	Uncharacterized protein
G1SFH9	CTNNB1	Uncharacterized protein
G1TMP7	STXBP1	Syntaxin binding protein 1
G1SY86	TMEM159	Uncharacterized protein
G1U415	VTN	Vitronectin
G1SPN1	NUDCD2	Uncharacterized protein
G1SS79	MARCKS	Uncharacterized protein
B6V9S9	CCT2	Chaperonin-containing T-complex polypeptide beta subunit
G1T096	SNRPF	Uncharacterized protein
G1T237	SLC25A3	Uncharacterized protein
G1T3S1	PSMC6	Uncharacterized protein
G1T5S9	UQCRQ	Uncharacterized protein
G1TIQ4	RPS6KA3	Ribosomal protein S6 kinase
G1TAX9	SNAP25	Synaptosomal-associated protein
G1TC10	UBE2M	Uncharacterized protein
G1TB98	RCN1	Uncharacterized protein
Q09YN4	CAPZA2	F-actin-capping protein subunit alpha-2
G1TNZ3		Uncharacterized protein

G1SCN8	CCT3	T-complex protein 1 subunit gamma
G1STF7	TF	Serotransferrin
G1SZF7	IDH2	Isocitrate dehydrogenase [NADP]
G1SSX5	MARS	Uncharacterized protein
G1T361	SUCLG2	Succinate--CoA ligase [GDP-forming] subunit beta, mitochondrial
U3KMZ3	ATIC	Uncharacterized protein
G1SUN1	ARL6IP5	PRA1 family protein
G1SGP1	UQCRC1	Uncharacterized protein
G1U6I6		Uncharacterized protein
P67777	PPP2CA	Serine/threonine-protein phosphatase 2A catalytic subunit alpha isoform
G1SKK0	GART	Trifunctional purine biosynthetic protein adenosine-3
G1T3X1	C9	Complement component C9
G1TM55	LOC108178879	40S ribosomal protein S6
G1T7U7	NAAA	N-acylethanolamine-hydrolyzing acid amidase
G1SPR7		Uncharacterized protein
G1SZ19	RAP2C	Uncharacterized protein
G1STB6	ACTC1	Uncharacterized protein
G1SQY9	DHDH	Trans-1,2-dihydrobenzene-1,2-diol dehydrogenase
G1SF08	RPL35A	Uncharacterized protein
G1TED6	ANXA5	Annexin
G1T6P5	ARL8B	ADP ribosylation factor like GTPase 8B
G1TNV7	GCAT	Glycine C-acetyltransferase
G1SG72	ABCE1	Uncharacterized protein
G1TUC8	ACTN4	Uncharacterized protein
G1T4T5	MECP2	Methyl-CpG-binding protein 2
P08507	GPD1	Glycerol-3-phosphate dehydrogenase [NAD(+)], cytoplasmic
G1TUB8	RPL11	Uncharacterized protein
G1TDC3	GANAB	Uncharacterized protein
G1TMU2	HNRNPF	Uncharacterized protein
G1SY93	RAC1	Rac family small GTPase 1
U3KNQ7	DDX23	Uncharacterized protein
G1SFE0	PSMD14	Uncharacterized protein
G1T0E9	LRRC57	Uncharacterized protein
G1TA80	GRIPAP1	Uncharacterized protein
G1SIT0	CLCC1	Uncharacterized protein
G1T7X6	LAMB1	Uncharacterized protein
G1T822	CEP290	Uncharacterized protein
G1SF82	TIMM10	Uncharacterized protein
G1SP48	UGDH	UDP-glucose 6-dehydrogenase
G1U3I5	ECH1	Uncharacterized protein
G1SCD7	LOC100356907	Uncharacterized protein
G1SWF6	HP	Haptoglobin

G1SM91	FAH	Fumarylacetoacetase
G1TVG8	PGRMC2	Uncharacterized protein
G1SZD2	HCCS	Cytochrome c heme lyase
G1SDX3	TOP2B	DNA topoisomerase 2
G1T3X8	HSPA12B	Uncharacterized protein
G1SHT4	SAT2	Uncharacterized protein
G1SSR8	CLPX	Uncharacterized protein
G1TVS4	HPX	Hemopexin
G1THU6	CD59	CD59 glycoprotein
G1SN05	EIF4A2	Uncharacterized protein
G1SHL9	EMC2	Uncharacterized protein
G1SY68	SF3B2	Uncharacterized protein
G1SEL8	ATP5F1E	Uncharacterized protein
Q6SQH4	S100a10	Protein S100-A10
G1T4A5	COL1A1	Collagen alpha-1(I) chain
G1T3G6	LOC100349005	Uncharacterized protein
G1SWW7	SRSF5	Uncharacterized protein
G1SVI9	VAPA	Uncharacterized protein
P07452	CA1	Carbonic anhydrase 1 (Fragment)
G1SZE0	VPS35	Vacuolar protein sorting-associated protein 35
G1SNY0	RPL27A	Uncharacterized protein
G1T0U8	PCYOX1	Uncharacterized protein
G1TRK3		Uncharacterized protein
G1T3H5	EIF2B3	Uncharacterized protein
G1SS91	C4A	Uncharacterized protein
G1SFH6	UBA2	Uncharacterized protein
G1SXZ9	BPHL	Biphenyl hydrolase like
G1TXW6	GPNMB	Uncharacterized protein
G1SP32	HIBCH	3-hydroxyisobutyryl-CoA hydrolase, mitochondrial
G1SEE8	LOC100338666	Uncharacterized protein
G1T748	TPP2	Uncharacterized protein
G1SN95	NUCB2	Nucleobindin 2
G1U5Y2		Uncharacterized protein
G1SF95	HNRNPD	Uncharacterized protein
G1SUI9	CALD1	Uncharacterized protein
G1SL62	ANXA2	Annexin
G1SJL6	SCARB2	Uncharacterized protein
G1SMI6	PRDX4	Uncharacterized protein
G1TYY5	LASP1	LIM and SH3 domain protein 1
G1SNE1	NXN	Uncharacterized protein
G1T8X9	VMA21	Vacuolar ATPase assembly integral membrane protein VMA21
G1T7Y5	DPM1	Dolichol-phosphate mannosyltransferase subunit 1
G1SE63	ABHD6	Uncharacterized protein
G1U9U2	F9	Coagulation factor IX
G1T4W2	RHEB	Uncharacterized protein
G1T7S0	MYL12B	Myosin light chain 12B
P62160	CALM	Calmodulin
P29694	EEF1G	Elongation factor 1-gamma
G1T8I8		Uncharacterized protein
G1T7W2	RTRAF	Uncharacterized protein

G1SKS8	MAP4	Microtubule-associated protein
G1SFF2	MAOA	Amine oxidase
G1T7W7	STT3A	Uncharacterized protein
G1SZ59	EIF4A1	Eukaryotic initiation factor 4A-I
G1SFE9	AHSA1	Uncharacterized protein
G1SCE1	GLIPR2	Uncharacterized protein
G1SMS2	PALLD	Uncharacterized protein
G1SZR7	KTN1	Uncharacterized protein
G1SY02	TUBB2A	Tubulin beta chain
G1SPR9	RPN2	Uncharacterized protein
G1SRN2		Uncharacterized protein
G1T994	COL12A1	Collagen alpha-1(XII) chain
G1T671	ACAT2	Uncharacterized protein
G1T782	SQOR	Uncharacterized protein
G1SWS9	VIM	Uncharacterized protein
G1STQ6	PSAT1	Phosphoserine aminotransferase
G1SN16	APEX1	DNA-(apurinic or apyrimidinic site) lyase
G1T379	MCCC1	Uncharacterized protein
G1TKQ5	PCBD1	Uncharacterized protein
G1U1T9	SORBS3	Sorbin and SH3 domain containing 3
G1T6Q8		Ferritin
G1T3M3	RAB21	Uncharacterized protein
G1SKF1	THBS1	Uncharacterized protein
G1U7S8	SPATS2L	Uncharacterized protein
G1T840	HSPH1	Uncharacterized protein
G1T090	DPP3	Dipeptidyl peptidase 3
G1SU89	SHPK	Uncharacterized protein
G1SE61	FLNB	Filamin-B
G1T6D1	RPL23	Uncharacterized protein
G1TE76	EIF4H	Eukaryotic translation initiation factor 4H
G1T798	STX4	Uncharacterized protein
P41982	SOD2	Superoxide dismutase [Mn], mitochondrial (Fragment)
G1T1T4	ADPGK	ADP dependent glucokinase
G1SD43	GPLD1	Uncharacterized protein
G1SR28	PAFAH1B3	Uncharacterized protein
G1T676	TMOD1	Uncharacterized protein
G1SQT0	PPP4C	Serine/threonine-protein phosphatase
G1SEM0	FERMT2	Uncharacterized protein
G1TD98	GSR	Glutathione reductase
G1TRG8	GNAI2	Uncharacterized protein
G1T1L4	GRWD1	Uncharacterized protein
G1U2R2	NPEPPS	Aminopeptidase
G1T0W0	RNF114	Ring finger protein 114
G1SEX5	SH3GL2	Uncharacterized protein
G1SIT5	RPL35	Uncharacterized protein
G1TEM7	ANXA3	Annexin
G1SMM7	SNRPD3	Small nuclear ribonucleoprotein Sm D3
G1TVT6	LOC108175352	Uncharacterized protein
G1TAV9	TGFBR3	Uncharacterized protein

U3KML1	BGN	Biglycan
Q9TTJ6	RGN	Regucalcin
G1T3D7	NANS	Uncharacterized protein
G1SWY0	RAB6A	Uncharacterized protein
G1STX4	SRP54	Signal recognition particle 54 kDa protein
G1TVH1	RACK1	Uncharacterized protein
G1SZ25	LOC100351756	Uncharacterized protein
G1TCF3	NPM3	Uncharacterized protein
P01697		Ig kappa chain V region AH80-5
B7NZJ1	CPNE1	Copine I, isoform 8 (Predicted)
G1U1M3	AIMP1	Uncharacterized protein
G1SHS7	WDR1	Uncharacterized protein
G1TW98		Uncharacterized protein
G1SWD1	FBL	Fibrillarin
G1U7C1	EML2	Echinoderm microtubule associated protein like 2
G1SYI2	GNB1	Uncharacterized protein
G1SZ91	FABP5	Uncharacterized protein
G1SSA8	TNXB	Uncharacterized protein
G1SNQ8	HEXB	Hexosaminidase subunit beta
G1SQM2	EPHX2	Uncharacterized protein
G1TVF7	PLCG1	1-phosphatidylinositol 4,5-bisphosphate phosphodiesterase gamma
G1SCT0	CKAP4	Cytoskeleton associated protein 4
G1SW24	AARS	Uncharacterized protein
G1SL80	UROD	Uroporphyrinogen decarboxylase
G1U0B5	PPME1	Protein phosphatase methylesterase 1
G1T2K1	PAPSS2	Uncharacterized protein
G1T4Z1	LRP1	Uncharacterized protein
G1U3G0	UBE2O	Ubiquitin conjugating enzyme E2 O
G1T2N1	ATOX1	Antioxidant 1 copper chaperone
G1SF70	SREK1	Uncharacterized protein
G1SI41		Uncharacterized protein
G1SVP7	GSTO1	Uncharacterized protein
G1T7H9	C1R	Uncharacterized protein
G1SNS5		Rab GDP dissociation inhibitor
G1TNM3	RPS3	Uncharacterized protein
G1TX74	TWF2	Uncharacterized protein
G1TEG8	PDCD6IP	Uncharacterized protein
G1U1X6	NT5C3A	5'-nucleotidase
G1SGA5	VAT1L	Uncharacterized protein
G1SVF2	PSMD1	26S proteasome non-ATPase regulatory subunit 1
G1SVK5	S100A4	Protein S100
G1T7Z0	PGD	6-phosphogluconate dehydrogenase, decarboxylating

G1T336	DDX3X	Uncharacterized protein
G1SLT8	HNRNPH3	Uncharacterized protein
G1TDQ3	BUB3	Uncharacterized protein
G1U612	LOC100344979	Uncharacterized protein
G1SFR8	RPS12	40S ribosomal protein S12
G1T182	MTDH	Uncharacterized protein
G1SD95		Uncharacterized protein
G1U2V8	APOC3	Uncharacterized protein
G1SGF8	C6	Uncharacterized protein
P27170	PON1	Serum paraoxonase/arylesterase 1
G1TSJ0		Uncharacterized protein
G1SH85	VPS33A	Uncharacterized protein
G1TA83	ANXA4	Annexin
G1T2Z8	MTAP	S-methyl-5'-thioadenosine phosphorylase
G1T4N5	MTOR	Serine/threonine-protein kinase mTOR
P58776	TPM2	Tropomyosin beta chain
G1SS94	CYP21A2	Uncharacterized protein
G1SQ01	LOC100346892	Uncharacterized protein
G1T7I3	ACACA	Uncharacterized protein
G1TMM5	ABHD16A	Uncharacterized protein
G1T3E6	DNPEP	Uncharacterized protein
G1T3Z6	PRKAR2B	Uncharacterized protein
G1SS85	GRHPR	Uncharacterized protein
G1SP54	LTA4H	Leukotriene A(4) hydrolase
G1SL41	GUSB	Beta-glucuronidase
G1SDU5	MACF1	Uncharacterized protein
G1SK48	BLMH	Bleomycin hydrolase
G1SCY3	UBR4	Uncharacterized protein
G1SMH9	NHLRC3	Uncharacterized protein
G1SZJ5	PFDN6	Uncharacterized protein
G1SLQ3	NQO1	Uncharacterized protein
G1STW0		Uncharacterized protein
G1T7G3	LPCAT3	Uncharacterized protein
G1TY77	ROCK2	Rho-associated protein kinase
G1TD24	ARHGAP35	Rho GTPase activating protein 35
G1SKT4	ATP5F1A	ATP synthase subunit alpha
G1T7I4	COPA	Coatomer subunit alpha
G1SE51	SPAG9	Uncharacterized protein
G1SYQ2	FADS6	Fatty acid desaturase 6
G1T6S6	ATP6V1F	V-type proton ATPase subunit F
G1U115	PSMD6	Uncharacterized protein
G1SUJ3	H2AFV	Histone H2A
U3KM53	TOM1	Uncharacterized protein
G1TZE2	GSTZ1	Uncharacterized protein
U3KMC6	CP	Uncharacterized protein
G1SHF9	UFC1	Ubiquitin-fold modifier-conjugating enzyme 1
G1T625	PLA2G12A	Uncharacterized protein
G1SPB6	ACSL1	Uncharacterized protein
Q01971	RAB2A	Ras-related protein Rab-2A
G1SSL0	ERP29	Endoplasmic reticulum resident protein 29

G1T593	COPZ1	Uncharacterized protein
G1T4F9	CRYAB	Alpha-crystallin B chain
G1SM77	ATP5F1C	ATP synthase subunit gamma
U3KMY5	NDUFB4	NADH:ubiquinone oxidoreductase subunit B4
G1TAY6	KRT19	Uncharacterized protein
G1T6G1	MMAB	Uncharacterized protein
G1SW82	SYNPO2	Uncharacterized protein
G1SS73	DDX1	Uncharacterized protein
G1T5E6	SNX2	Uncharacterized protein
G1TUP7	LOC100345716	Uncharacterized protein
G1TJY2	CHID1	Chitinase domain containing 1
G1SZ16	TXNRD2	Thioredoxin reductase 2
G1SJN4	COPS4	Uncharacterized protein
G1SRQ2	UCHL3	Ubiquitin carboxyl-terminal hydrolase
G1T0T5	COPS7A	Uncharacterized protein
G1T7A2	C4BPA	Uncharacterized protein
B7NZG7	SNX3	Sorting nexin 3 (Predicted)
G1SMZ5	EIF3A	Eukaryotic translation initiation factor 3 subunit A
G1SQI0	FUBP3	Far upstream element binding protein 3
G1SJ23	CAPN1	Calpain-1 catalytic subunit
G1T667	CCS	Uncharacterized protein
G1TRV4		Uncharacterized protein
G1STH4	GNA13	Uncharacterized protein
G1TXB6		Uncharacterized protein
G1SRA9	ILK	Uncharacterized protein
G1STP6	CTNNA1	Catenin alpha-1
G1TWR0		Uncharacterized protein
G1TFB5	RAB18	Uncharacterized protein
G1TSG1	RPL22	Uncharacterized protein
G1TUM0	LOC100359118	Uncharacterized protein
G1T2G4	EIF2S1	Eukaryotic translation initiation factor 2 subunit 1
G1T888	ANKFY1	Uncharacterized protein
G1TYE2	LXN	Uncharacterized protein
G1SN70	SERPINB9	Serpin family B member 9
G1SUD2	CYP20A1	Uncharacterized protein
G1T2F2	PPIB	Peptidyl-prolyl cis-trans isomerase
G1SWK8	PSMB7	Proteasome subunit beta
G1T8F7	NOP56	Uncharacterized protein
G1TYA7	LDHB	L-lactate dehydrogenase
Q28685	DAG1	Dystroglycan
G1SLT2		Uncharacterized protein
G1TDF3	COQ8A	Uncharacterized protein
G1SQ87	PFN2	Profilin
G1SRR2	LMO7	Uncharacterized protein
G1T8V2	MFAP4	Uncharacterized protein
G1SED9	EIF3L	Eukaryotic translation initiation factor 3 subunit L
G1SYR9	IMMT	MICOS complex subunit MIC60
G1SF47	11-Sep	Uncharacterized protein

G1T8T3	C1QC	Complement C1q C chain
G1T6S2	PGM5	Uncharacterized protein
G1SZD6	YWHAQ	14-3-3 protein theta
G1TEV2		Uncharacterized protein
G1T9P2	CARKD	ATP-dependent (S)-NAD(P)H-hydrate dehydratase
G1SHD6	MAT2A	S-adenosylmethionine synthase
G1SJY8	HMGCS1	3-hydroxy-3-methylglutaryl coenzyme A synthase
G1T2K5	MYBBP1A	Uncharacterized protein
G1STV0	CAST	Calpastatin
G1T7S5	ABCC4	Uncharacterized protein
G1T706	RTCA	Uncharacterized protein
P04221		Ig mu chain C region membrane-bound form
P00389	POR	NADPH--cytochrome P450 reductase
G1SIT6	ARPC5L	Actin-related protein 2/3 complex subunit 5
G1T0N4	NPM1	Uncharacterized protein
G1SVM1	MVP	Uncharacterized protein
G1SY96	SLC25A13	Uncharacterized protein
G1TLW3	DDX5	Uncharacterized protein
G1SES2	NAA10	Uncharacterized protein
G1SVA3	PSMD11	Uncharacterized protein
G1T1B8		Uncharacterized protein
G1SK09	ACSL5	Uncharacterized protein
G1TUD2	GNG12	Guanine nucleotide-binding protein subunit gamma
G1TNJ2	ZYX	Uncharacterized protein
G1T0H0	ADPRHL2	ADP-ribosylhydrolase like 2
U3KN22	GSTK1	Glutathione S-transferase kappa
G1TED0	MPST	Sulfurtransferase
G1T926	DCTN1	Uncharacterized protein
G1TST2	TM7SF2	Transmembrane 7 superfamily member 2
G1SN00	KNG1	Uncharacterized protein
G1TBW9	DYNLL2	Dynein light chain
G1SCE4	CLTA	Clathrin light chain
G1SQA8	ATP5F1B	ATP synthase subunit beta
G1TQR0	ACTN1	Uncharacterized protein
G1SLS3	AP1G1	AP-1 complex subunit gamma
G1T8R3	UTRN	Uncharacterized protein
G1SV35	PRKAG1	Uncharacterized protein
G1TLH1	GSTM4	Uncharacterized protein
G1TVS8		Uncharacterized protein
G1TAC3	AFG3L2	Uncharacterized protein
G1U8B3	RBP1	Uncharacterized protein
G1T550	RAB5C	Uncharacterized protein
A0A0G2JH24		Uncharacterized protein
G1SHG0	RPL31	Uncharacterized protein
G1SWN1	SNF8	Vacuolar-sorting protein SNF8

G1TMZ6	LAMP2	Uncharacterized protein
G1TI71	UGP2	UTP--glucose-1-phosphate uridylyltransferase
G1SF32	TOMM70	Uncharacterized protein
G1T432	CAP1	Adenylyl cyclase-associated protein
G1TVQ3	DYNLRB1	Dynein light chain roadblock
G1TSP4	TMEM245	Uncharacterized protein
G1T4V2	2-Mar	Uncharacterized protein
G1SSN2	SIRT5	NAD-dependent protein deacylase sirtuin-5, mitochondrial
G1TX70		Uncharacterized protein
G1TD91	NUDT21	Uncharacterized protein
G1SR07	CBR4	Uncharacterized protein
G1T2V0	MRPS26	Uncharacterized protein
G1SZQ7	BZW1	Uncharacterized protein
G1SJI7	DMAC2L	Uncharacterized protein
G1T7V5	DLD	Dihydrolipoyl dehydrogenase
P21195	P4HB	Protein disulfide-isomerase
G1SDA8	PSMA1	Proteasome endopeptidase complex
G1SL60	SF3B3	Uncharacterized protein
G1TTJ4	EML3	Echinoderm microtubule associated protein like 3
G1SER3	SLC25A11	Uncharacterized protein
P09212	SOD1	Superoxide dismutase [Cu-Zn]
G1SPQ9	SORBS2	Uncharacterized protein
G1TWL0	HNRNPA2B1	Uncharacterized protein
G1STX7	KYAT3	Uncharacterized protein
G1SH00	PML	Uncharacterized protein
G1SDS3	NDRG2	Uncharacterized protein
G1TV17	ADIPOQ	Uncharacterized protein
G1T845	NSF	Uncharacterized protein
G1U9Q9	PLG	Uncharacterized protein
G1SJM1	APOH	Uncharacterized protein
G1SZ00	CSRP1	Uncharacterized protein
G1U3V0	HINT2	Uncharacterized protein
G1SCQ1	AKR7L	Uncharacterized protein
G1SL95	PMM2	Phosphomannomutase
G1TF72	CHCHD4	Uncharacterized protein
G1TP15	PSMD3	Uncharacterized protein
G1T2J6	SLC25A12	Uncharacterized protein
G1TDJ2	TIMM8B	Uncharacterized protein
G1TWQ3	SIRT2	NAD-dependent protein deacetylase
G1SPY1	GBE1	Uncharacterized protein
G1SET0	COPB2	Coatomer subunit beta'
G1T6H0	MVK	Mevalonate kinase
G1TWK7	MESD	Uncharacterized protein
G1SUP4	ZNF207	Uncharacterized protein
G1SU71	PSMB1	Proteasome subunit beta
G1SZ63	ALDH9A1	Uncharacterized protein
G1T2C3	CSDE1	Uncharacterized protein
G1SR79	ECHDC1	Uncharacterized protein

G1T0K1	TMEM43	Uncharacterized protein
G1SU82	GC	Vitamin D-binding protein
G1T823	COTL1	Coactosin like F-actin binding protein 1
G1T545	BTBD9	Lactoylglutathione lyase
P58772	TPM1	Tropomyosin alpha-1 chain
G1T650	OGDHL	Uncharacterized protein
U3KPE7	AKR1B1	Aldose reductase
G1SL02	AP2B1	AP complex subunit beta
G1SYD6	LMNA	Uncharacterized protein
G1SKN2		Uncharacterized protein
G1TA59	IDH3A	Isocitrate dehydrogenase [NAD] subunit, mitochondrial
G1U9R6	FN1	Fibronectin
G1T765	MDH2	Malate dehydrogenase
G1SML5	SFXN1	Sidoreflexin
G1TAP1	PPA1	Uncharacterized protein
G1SW77	SERBP1	Uncharacterized protein
G1SK04	ENO2	Uncharacterized protein
P17177	CYP27A1	Sterol 26-hydroxylase, mitochondrial
G1TEM5	DPY30	Uncharacterized protein
G1SVK2	AGFG2	Uncharacterized protein
G1SEK8	FETUB	Uncharacterized protein
G1SZV5	APOA1	Apolipoprotein A-I
G1SVJ1	DPYD	Dihydropyrimidine dehydrogenase [NADP(+)]
G1TK53	ACY1	Uncharacterized protein
G1SGV9	PSMB8	Proteasome subunit beta
G1U159		Uncharacterized protein
G1SH49	DHRS7	Uncharacterized protein
G1T7T2	SAFB	Scaffold attachment factor B
O97972	INMT	Indolethylamine N-methyltransferase
G1TCK9	IARS	Uncharacterized protein
G1STU4	CAVIN3	Uncharacterized protein
G1TLI7	MGARP	Uncharacterized protein
G1U1W4	APEH	Acylamino-acid-releasing enzyme
G1T332	GOT1	Aspartate aminotransferase
G1SSV1	COPS8	Uncharacterized protein
G1U723	PGER5	3alpha/17beta/20alpha-hydroxysteroid dehydrogenase
G1T0Z8	PFDN5	Uncharacterized protein
G1SV32	RPL7	Uncharacterized protein
G1T5N5	GFM1	Elongation factor G, mitochondrial
G1SIK0	SERPINC1	Uncharacterized protein
G1T8R1	RAP1GAP2	Platelet-activating factor acetylhydrolase IB subunit alpha
G1SMN6		Uncharacterized protein
G1T5C5	ERLIN2	Uncharacterized protein
G1TAN8	AOC2	Amine oxidase
G1TTQ5	LOC100348796	60S ribosomal protein L36

G1SI19	NUDT9	Uncharacterized protein
G1SHC5	RCC1	Uncharacterized protein
G1U797	FXR1	Uncharacterized protein
G1SDJ7	PABPC4	Polyadenylate-binding protein
G1TJN8	SYNCRIP	Uncharacterized protein
G1SUC8	EIF3E	Eukaryotic translation initiation factor 3 subunit E
G1TKE8	LOC100357845	Uncharacterized protein
G1SR27	GRB2	Uncharacterized protein
G1TAF8	STIP1	Uncharacterized protein
G1SSK9	LOC100349893	Uncharacterized protein
G1SWT9		Uncharacterized protein
G1SQR7	NUTF2	Uncharacterized protein
G1SCI5	MYO1C	Uncharacterized protein
G1TTL1	NAA15	Uncharacterized protein
G1SEW3	RANBP2	Uncharacterized protein
G1TUA3		Uncharacterized protein
G1TP59	LOC100345328	Uncharacterized protein
G1TC19	CPT2	Uncharacterized protein
G1U2Q6	SMTN	Uncharacterized protein
G1T8Z0	PRDX6	Uncharacterized protein
G1SG11	COX4I1	Cytochrome c oxidase subunit 4 isoform 1, mitochondrial
G1T508	AASS	Uncharacterized protein
G1TNW2	SLMAP	Sarcolemmal membrane-associated protein
G1SPB2	RNMT	mRNA cap guanine-N7 methyltransferase
G1SP02	PDPR	Uncharacterized protein
G1U2E3	CHMP4B	Uncharacterized protein
G1T302	THYN1	Uncharacterized protein
G1TKY2	LOC100341515	Uncharacterized protein
G1TWC3	TMX1	Uncharacterized protein
Q28619	SLC9A3R1	Na(+)/H(+) exchange regulatory cofactor NHE-RF1
G1SY36	TPM4	Uncharacterized protein
G1TN89	HSPG2	Uncharacterized protein
G1TR42	RNPEP	Uncharacterized protein
G1THF1	COQ9	Uncharacterized protein
G1SZW0	CYFIP1	Cytoplasmic FMR1-interacting protein
G1U974	DPYSL2	Uncharacterized protein
G1TZN7		Uncharacterized protein
P80456	AOX1	Aldehyde oxidase 1
G1TBS1	PARK7	Uncharacterized protein
G1SD83	ITGA6	Uncharacterized protein
G1T7Z6	PGK1	Phosphoglycerate kinase
G1T5J8	DCTN4	Uncharacterized protein
G1SI54	ILVBL	IlvB acetolactate synthase like
G1U7I9	DDX39B	Uncharacterized protein
O79431	MT-ATP8	ATP synthase protein 8
G1T188	AK3	GTP:AMP phosphotransferase AK3, mitochondrial

G1SFV8	GHITM	Uncharacterized protein
G1TZ63		Uncharacterized protein
G1TH59	RBM39	Uncharacterized protein
G1TPN3	HNRNPAB	Uncharacterized protein
G1SW61	SMU1	Uncharacterized protein
G1TCC2	ECHDC2	Uncharacterized protein
G1TKE0	DPM3	Dolichol-phosphate mannosyltransferase subunit 3
G1TMF1	KANK1	Uncharacterized protein
G1TCQ4	GNPDA1	Glucosamine-6-phosphate isomerase
Q28888	DCN	Decorin
G1TBR5	SEC61A1	Uncharacterized protein
G1SMR7	RPL12	Uncharacterized protein
G1T580	CBX5	Uncharacterized protein
G1SNM1	GARS	Uncharacterized protein
G1U0Y6	ECI1	Enoyl-CoA delta isomerase 1
G1THY5	PPIL1	Peptidyl-prolyl cis-trans isomerase
G1TJH2		60S ribosomal protein L29
G1SWD0	PCSK1N	Uncharacterized protein
G1T8S4	CLUH	Clustered mitochondria protein homolog
G1SX37	CFL2	Cofilin 2
G1SWC9	IST1	Uncharacterized protein
G1T3H3	RHOT1	Mitochondrial Rho GTPase
G1TT67	LOC100357801	Uncharacterized protein
G1SGQ5	AHSG	Alpha-2-HS-glycoprotein
G1T9F3	KPNB1	Uncharacterized protein
P30947	HSP90AB1	Heat shock protein HSP 90-beta
G1U945	LOC100354714	Uncharacterized protein
G1SRK1	LPCAT2	Uncharacterized protein
G1U4Y5	FCGRT	Fc fragment of IgG receptor and transporter
P16258	OSBP	Oxysterol-binding protein 1
G1SHB9	CAPG	Uncharacterized protein
P01948		Hemoglobin subunit alpha-1/2
G1SVD5	ADAR	Uncharacterized protein
G1T868	CLTB	Clathrin light chain
U3KMD1	SNX6	Uncharacterized protein
G1TBQ6	VAPB	VAMP associated protein B and C
G1SI83	S100A13	Uncharacterized protein
G1SKS9	TXNRD1	Uncharacterized protein
G1SV26	ZFYVE1	Uncharacterized protein
G1TB49	APOA1BP	NAD(P)H-hydrate epimerase
G1SU17	NSDHL	Uncharacterized protein
G1STQ7	TMEM97	Transmembrane protein 97
G1TH06	RANBP1	RAN binding protein 1
G1SIB6	BLVRB	Biliverdin reductase B
G1SM08	CYP51A1	Uncharacterized protein
G1SHI0	ACAA1	Acetyl-CoA acyltransferase 1

G1STT8	HOGA1	Uncharacterized protein
G1T7U6	EFEMP1	Uncharacterized protein
G1SXK6	PDLIM7	Uncharacterized protein
G1T7L0	CTSC	Uncharacterized protein
G1T548	COL4A2	Uncharacterized protein
G1SNX5	ILF2	Uncharacterized protein
G1SYM7	LMCD1	Uncharacterized protein
G1T4X8	PSMB2	Proteasome subunit beta
G1SWL6	RAI14	Uncharacterized protein
G1TTR4	LOC100338787	Glycine cleavage system H protein
G1SWM1		Uncharacterized protein
G1T8K2	PACSIN2	Uncharacterized protein
G1TFL3		GTP-binding nuclear protein Ran
G1T6C0	RAB5B	Uncharacterized protein
G1U6B2	ALAD	Delta-aminolevulinic acid dehydratase
G1SVH0	AHCY	Adenosylhomocysteinase
G1STW7	FARSB	Uncharacterized protein
G1TUD6	PSMC4	Proteasome 26S subunit, ATPase 4
G1TJ91		Uncharacterized protein
G1TVT8	GPD1	Glycerol-3-phosphate dehydrogenase [NAD(+)]
G1T0M2	TRMT10C	Uncharacterized protein
G1SZT4	PAPLN	Uncharacterized protein
G1SKL7	VAT1	Vesicle amine transport 1
G1TXN1	NIT2	Uncharacterized protein
G1TLK9	ACADVL	Uncharacterized protein
G1T1F0	RPS14	Uncharacterized protein
G1T1X2	LPP	Uncharacterized protein
G1SQU6	NDUFS3	Uncharacterized protein
G1SYT7	PMPCB	Uncharacterized protein
G1SCP0	AP3B1	AP-3 complex subunit beta
G1TCL7	RMDN3	Uncharacterized protein
G1ST12	SNX27	Uncharacterized protein
U3KMZ9	LOC100346472	Uncharacterized protein
G1TEH2	ATP6V1C1	V-type proton ATPase subunit C
G1TU32	LAMTOR2	Uncharacterized protein
G1SU66	FHL2	Uncharacterized protein
G1TMS5	CCT5	T-complex protein 1 subunit epsilon
G1TEU5	CPNE3	Uncharacterized protein
G1TA10	TMEM109	Transmembrane protein 109
G1TPB1	CRAT	Carnitine O-acetyltransferase
G1SUZ1		Uncharacterized protein
G1U6H0	CANX	Uncharacterized protein
G1TYH7	APMAP	Adipocyte plasma membrane associated protein
G1SLS1		Uncharacterized protein
G1TWS0	CDIPT	CDP-diacylglycerol--inositol 3-phosphatidyltransferase

G1U3Q6		Uncharacterized protein
G1SER8	PFN1	Profilin
G1SXQ1	MCCC2	Uncharacterized protein
G1TB81	CDA	Cytidine deaminase
G1SXF7	AAK1	Uncharacterized protein
G1SWW8	PPP1CC	Serine/threonine-protein phosphatase
G1U5U0	NOP58	Uncharacterized protein
G1TAS1	COA7	Uncharacterized protein
G1SDZ3	PLTP	Phospholipid transfer protein
G1SG80	AK2	Adenylate kinase 2, mitochondrial
G1U535	RDH14	Uncharacterized protein
G1SUY2	ALDH2	Uncharacterized protein
G1TXF6	LOC100356974	60S ribosomal protein L27
G1TDN1	FAM136A	Uncharacterized protein
O46503	SULT1C2	Sulfotransferase 1C2
G1TKY3	CAPN2	Calpain-2 catalytic subunit
G1T616	PGM2	Uncharacterized protein
P18055		Metallothionein-2A
G1SD02	COPS6	COP9 signalosome subunit 6
G1SHQ2	RPL21	Uncharacterized protein
G1SXI9	COX6B1	Cytochrome c oxidase subunit
G1U7K4		Uncharacterized protein
G1TRZ2	LAMP1	Lysosomal associated membrane protein 1
G1ST69	LMNB1	Uncharacterized protein
P18287	APOE	Apolipoprotein E
G1SK42	TGM2	Uncharacterized protein
G1SUY3	CSRP2	Uncharacterized protein
G1SY84	DYNC1I2	Uncharacterized protein
U3KNW1	ACOT7	Uncharacterized protein
G1TVG7	SF3B1	Uncharacterized protein
G1SIS2	STXBP3	Uncharacterized protein
G1TRS4	FKBP9	Peptidylprolyl isomerase
G1TKP3		Uncharacterized protein
G1TKA4	CYSTM1	Uncharacterized protein
G1TPD4	SETD7	Histone-lysine N-methyltransferase SETD7
G1SPJ2	ARL6IP1	Uncharacterized protein
G1SM15	AP2M1	Uncharacterized protein
G1SR29	ATP6V1A	Uncharacterized protein
G1SMH2	C7	Uncharacterized protein
G1U4Z7	NBL1	MICOS complex subunit MIC10
G1SNP9	ACADSB	Acyl-CoA dehydrogenase short/branched chain
G1SK12	PTPN6	Tyrosine-protein phosphatase non-receptor type
G1T7Q3	RAB35	Uncharacterized protein
G1TR00	YIPF5	Protein YIPF
G1TQC7	CPSF7	Cleavage and polyadenylation specific factor 7

G1SPF5	PGAM1	Uncharacterized protein
G1T1H9	ITGB2	Integrin beta
G1TEG0	CYP17A1	Uncharacterized protein
G1T5W4	ACADM	Uncharacterized protein
G1T006	NENF	Neudesin neurotrophic factor
G1SQF0	CTSH	Cathepsin H
G1SM21		Uncharacterized protein
G1U8D4	ITGA7	Uncharacterized protein
G1SVY8	CKB	Creatine kinase B-type
G1TBR1	NQO2	Uncharacterized protein
G1TAL6	GALNT2	Polypeptide N-acetylgalactosaminyltransferase
G1TD94	LRP1B	Uncharacterized protein
G1SXG6	S100A8	Protein S100
G1SQI2	PRPH	Uncharacterized protein
Q9XS70	CORO1B	Coronin-1B
G1SMI7	DAP3	Uncharacterized protein
G1TEA3	CAVIN2	Uncharacterized protein
G1STN6	LOC100342438	Uncharacterized protein
U3KPD5	RPL37	Ribosomal protein L37
G1TCZ0	COX5B	Uncharacterized protein
G1SCE7	HADHB	Uncharacterized protein
G1TRH3	CTSZ	Uncharacterized protein
G1SZR8	HDGF	Uncharacterized protein
G1SF06	EHD3	Uncharacterized protein
G1TDF6	FLOT1	Uncharacterized protein
G1SQG6	SERPINA5	Serpin family A member 5
G1SYL8	ELOB	Elongin B
U3KPJ9	PLVAP	Plasmalemma vesicle associated protein
G1T1U7	MTHFD1	Uncharacterized protein
U3KMS5	COPS3	Uncharacterized protein
G1TB18	ARHGAP1	Uncharacterized protein
G1SIV7	NARS	Uncharacterized protein
G1TES2	ATP5IF1	Uncharacterized protein
G1SVJ5	SARS	Serine--tRNA ligase, cytoplasmic
G1TKQ2		Uncharacterized protein
G1T295	EPHX1	Epoxide hydrolase
G1U9D3	VPS28	Vacuolar protein sorting-associated protein 28 homolog
G1T2I4	EPRS	Uncharacterized protein
G1TKE3	TAGLN2	Transgelin
G1SYM4	A1BG	Alpha-1B-glycoprotein
G1U354	PSMD8	Uncharacterized protein
G1SRL4	NAGA	Alpha-galactosidase
G1SY27	TRIAP1	Uncharacterized protein
G1U8Y9	PSPH	Uncharacterized protein
G1SDJ3		Uncharacterized protein
G1TAE2	ELAVL1	ELAV-like protein
G1TWY9	GOLIM4	Uncharacterized protein
G1SPP0	STOM	Uncharacterized protein
G1SLC0	ERP44	Uncharacterized protein
G1SWR0	HEXA	Beta-hexosaminidase

G1TLD8		Small ubiquitin-related modifier
G1THL4	LOC100343655	Uncharacterized protein
G1TYV6	ATP13A1	Cation-transporting ATPase
G1U8T9	KCTD12	Potassium channel tetramerization domain containing 12
G1SJN5	MAN2A1	Alpha-mannosidase
G1T103	C12orf10	Uncharacterized protein
G1TV31	ARPC5	Actin-related protein 2/3 complex subunit 5
G1SVC5	DNAJC25	Uncharacterized protein
G1U3L3	CYP11A1	Cholesterol side-chain cleavage enzyme, mitochondrial
P62139	PPP1CA	Serine/threonine-protein phosphatase PP1-alpha catalytic subunit
G1T8P1	ALDH1L1	Uncharacterized protein
U3KMP1	RAB10	Uncharacterized protein
G1T9V1	PDHB	Uncharacterized protein
G1T6L7	CHCHD3	MICOS complex subunit
G1SJK0	APOOL	MICOS complex subunit
G1SXX2		Uncharacterized protein
G1TC48	HNRNPUL1	Uncharacterized protein
G1TB96	COL15A1	Uncharacterized protein
P14461	FGA	Fibrinogen alpha chain (Fragment)
P37153	APOD	Apolipoprotein D
G1TX43	IPO7	Uncharacterized protein
G1SQP9	COMT	Catechol-O-methyltransferase
G1SWS6	FMOD	Fibromodulin
G1T7T6	USO1	Uncharacterized protein
G1SGQ0	DNAJB11	Uncharacterized protein
G1U3B8	TXNL1	Uncharacterized protein
G1T0L1	LSM3	U6 snRNA-associated Sm-like protein LSm3
G1T087	CUL5	Cullin-5
U3KLY7	ANP32B	Uncharacterized protein
G1TSK0	RAVER2	Uncharacterized protein
G1TB64	MLYCD	Uncharacterized protein
G1SNM8	JAGN1	Uncharacterized protein
G1TI53	MYO6	Uncharacterized protein
G1SYS5	ALDH7A1	Uncharacterized protein
G1T641	ACOT13	Uncharacterized protein
G1TE61	KPNA6	Importin subunit alpha
G1SZ35	CHMP3	Uncharacterized protein
G1T376	TSC22D3	Uncharacterized protein
G1THH7	SUN2	Uncharacterized protein
G1TFD8		Uncharacterized protein
G1SRW4	EMILIN1	Uncharacterized protein
G1SZL5	ELF1	Uncharacterized protein
A0A140TAW0	CALU	Calumenin
G1TV79		Uncharacterized protein
G1SSJ7	PHB	Uncharacterized protein
G1SG48	WIPI2	Uncharacterized protein
G1SS70	RPS3A	40S ribosomal protein S3a

G1T4Z2	ACLY	ATP-citrate synthase
G1SD60		Uncharacterized protein
G1SR13	STOML2	Uncharacterized protein
G1TS93	PPP1R7	Uncharacterized protein
G1SCT1	PREP	Uncharacterized protein
G1SHU8	RPL32	Uncharacterized protein
G1T958	EBP	Uncharacterized protein
G1TCX0	NFS1	Uncharacterized protein
G1SS69	CFB	Uncharacterized protein
G1T7C0	TFAM	Uncharacterized protein
G1T125	HSCB	Uncharacterized protein
G1SZN0	FAM120A	Uncharacterized protein
G1SFU0	RAB7A	Ras-related protein Rab-7a
G1T329	CDK5RAP3	Uncharacterized protein
G1SKJ5	SNAP29	Uncharacterized protein
G1SZ03	EIF3B	Eukaryotic translation initiation factor 3 subunit B
P00883	ALDOA	Fructose-bisphosphate aldolase A
G1SN67	SERPINB1	Serpin family B member 1
G1TN54	NIPSNAP1	Uncharacterized protein
G1SSV4	PLCB1	1-phosphatidylinositol 4,5-bisphosphate phosphodiesterase
G1U7K9		Uncharacterized protein
G1SPZ9	PPA2	Uncharacterized protein
G1TVQ6		Uncharacterized protein
G1T5H8	EIF4B	Uncharacterized protein
G1U446	CLINT1	Uncharacterized protein
G1SDR2	MYL9	Uncharacterized protein
G1T2V6	NAPG	Uncharacterized protein
G1T6L5	SH3BGRL	Uncharacterized protein
G1U1F6	HSPB6	Uncharacterized protein
G1SVR6		Uncharacterized protein
G1TAH7	TKT	Uncharacterized protein
G1TUX2	ACO2	Aconitate hydratase, mitochondrial
G1SCI0	DYNC1H1	Dynein cytoplasmic 1 heavy chain 1
G1TK63	ENPP1	Uncharacterized protein
P01377		Corticostatin-4
G1SIB9	PYGB	Alpha-1,4 glucan phosphorylase
G1SEF9	DES	Uncharacterized protein
G1SVV6	MAP2K1	Dual-specificity mitogen-activated protein kinase kinase 1
G1SLQ4	ACTR3	Uncharacterized protein
G1SM05	SAR1A	Uncharacterized protein
G1U6N8	NUMA1	Uncharacterized protein
G1U8Y1		Uncharacterized protein
G1U3M5	PRMT1	Uncharacterized protein
G1TKX3	FGG	Uncharacterized protein
G1U8Y2	TJP1	Uncharacterized protein
G1SEI0	SUOX	Uncharacterized protein
G1SLX0	GMFB	Uncharacterized protein
G1TVY5	CTNND1	Uncharacterized protein
G1TX78	TOMM20	Uncharacterized protein

G1TBW7	GNAQ	Uncharacterized protein
G1U6F4	PURB	Uncharacterized protein
G1TZV1	LOC100351488	Carboxylic ester hydrolase
G1U724	SAE1	SUMO1 activating enzyme subunit 1
P00563	CKM	Creatine kinase M-type
G1T916	EIF5A	Eukaryotic translation initiation factor 5A
G1T7R2	YWHAE	Uncharacterized protein
G1T519	PSMA4	Proteasome subunit alpha type
G1T346	SPTAN1	Uncharacterized protein
G1TME7	CSTB	Cystatin B
G1TLE4	GNB2	Uncharacterized protein
G1TZI2	PGAM5	PGAM family member 5, mitochondrial serine/threonine protein phosphatase
G1SW32	LOC100340393	Uncharacterized protein
G1SL46	PSMD9	Uncharacterized protein
G1TA48	EHD4	Uncharacterized protein
G1U1W3	PDK2	Uncharacterized protein
G1SIP2	HNRNPL	Uncharacterized protein
G1ST15	AUH	AU RNA binding methylglutaconyl-CoA hydratase
G1U315	CAVIN1	Uncharacterized protein
G1TWK1	SACM1L	Uncharacterized protein
G1T2K2		Uncharacterized protein
G1SH63	GSS	Glutathione synthetase
G1TA15	LARS	Uncharacterized protein
G1T4M1	LAMC1	Laminin subunit gamma 1
G1SQZ4	RUVBL2	RuvB-like helicase
G1SCP8	MSN	Uncharacterized protein
G1SH10	NUP93	Nuclear pore complex protein Nup93
G1SJJ9	MSRA	Uncharacterized protein
G1SZK4	PDAP1	PDGFA associated protein 1
G1SM70		Enhancer of rudimentary homolog
G1T035	PRKCD	Protein kinase C delta type
G1SV28		Uncharacterized protein
G1SP97	LUM	Lumican
G1SFE6	SNRPB2	Uncharacterized protein
G1U7G6	SDHA	Succinate dehydrogenase [ubiquinone] flavoprotein subunit, mitochondrial
G1T4Q9	PSMB5	Proteasome subunit beta
G1T918	PSMB4	Proteasome subunit beta
G1STE1	LOC100343982	Cytochrome b-c1 complex subunit 6
G1SGI8	TXNDC12	Uncharacterized protein
G1TE96	LANCL1	Uncharacterized protein
G1SNE5	THEM4	Uncharacterized protein
G1T6Z1	CARMIL1	Uncharacterized protein
G1T2E6	GCDH	Uncharacterized protein
G1SZ64	CD68	Uncharacterized protein
G1T673	CHP1	Uncharacterized protein

G1TMM0	ITIH3	Inter-alpha-trypsin inhibitor heavy chain H3
G1TY29	ITIH4	Uncharacterized protein
G1SDG2	PITPNB	Phosphatidylinositol transfer protein beta
P68105	EEF1A1	Elongation factor 1-alpha 1
G1T3V2	HSPB1	Uncharacterized protein
U3KM89	PPOX	Protoporphyrinogen oxidase
G1T0Y9	DCTN2	Uncharacterized protein
G1SIV3	FECH	Ferrochelatase
G1TFZ6		Uncharacterized protein
P09809	APOA1	Apolipoprotein A-I
G1SI20	IDH1	Isocitrate dehydrogenase [NADP]
G1SFF7	MAOB	Amine oxidase
G1TII2	RTN3	Reticulon
G1SWF0	SERPIND1	Heparin cofactor 2
G1U7U3	NME1	Nucleoside diphosphate kinase
U3KMH8	LOC100338456	Uncharacterized protein
G1T2K6	ATAD1	Uncharacterized protein
G1TBL1	SLC25A20	Uncharacterized protein
G1T824	TMSB15A	Thymosin beta
G1TF32	ATL3	Atlastin GTPase 3
G1T5F8	CRYL1	Lambda-crystallin
G1SW97	IVD	Uncharacterized protein
Q09YN6	CAV1	Caveolin-1
G1SNK5	GGCT	Uncharacterized protein
G1SJ57	SBDS	Uncharacterized protein
G1SQG5	MDH1	Malate dehydrogenase
G1SPJ5	GCN1	Uncharacterized protein
G1SN06	ACSL4	Uncharacterized protein
G1TSL1	IDH3B	Isocitrate dehydrogenase [NAD] subunit, mitochondrial
G1U4P7		Superoxide dismutase
G1TYR3		Uncharacterized protein
G1TEZ4	NUDC	Uncharacterized protein
G1U7L4	HSPA5	Heat shock protein family A (Hsp70) member 5
G1TUC2	CNBP	Uncharacterized protein
G1T1V9	HSPA2	Uncharacterized protein
G1SS37	ALDH1L2	10-formyltetrahydrofolate dehydrogenase
G1SZ85	BPNT1	Uncharacterized protein
P62493	RAB11A	Ras-related protein Rab-11A
O19048	PCBP1	Poly(rC)-binding protein 1
G1U0A2	VSNL1	Uncharacterized protein
G1SXX5	HADHA	Uncharacterized protein
G1TI40	SNRPD2	Small nuclear ribonucleoprotein Sm D2
G1TQ79	CHCHD6	MICOS complex subunit
G1U6H4	DDAH2	Uncharacterized protein
G1SPF9	C5	Uncharacterized protein
G1T2U6	DDX46	Uncharacterized protein
G1SXN3	LAMA4	Laminin subunit alpha 4

G1TUM2		Uncharacterized protein
G1SY88	ADK	Uncharacterized protein
G1T0X2	FGA	Fibrinogen alpha chain
G1TZQ6	NDUFA10	NADH dehydrogenase [ubiquinone] 1 alpha subcomplex subunit 10, mitochondrial
Q8MJF1	OGN	Mimecan
G1SCZ9	UBXN4	Uncharacterized protein
G1SK22	RPS27A	Uncharacterized protein
G1TX94	MATR3	Uncharacterized protein
G1U603	DDI2	DNA damage inducible 1 homolog 2
G1SLD6	HARS	Uncharacterized protein
G1T9N3	EML4	Uncharacterized protein
P01870		Ig gamma chain C region
Q9GKX2	DHRS4	Dehydrogenase/reductase SDR family member 4 (Fragment)
G1SLI0	CCDC47	Uncharacterized protein
G1SWI3	VDAC2	Voltage-dependent anion-selective channel protein 2
G1SH26	GLOD4	Uncharacterized protein
G1SY70	SEC23A	Protein transport protein SEC23
G1T2I5	RTN4	Reticulon
G1T5V5	SORBS1	Uncharacterized protein
G1TBC1	HSP90B1	Endoplasmin
G1SLF1	ADH5	S-(hydroxymethyl)glutathione dehydrogenase
G1U8F0	AP2A1	AP-2 complex subunit alpha
G1SW67	H2AFY	Core histone macro-H2A
G1SI26	CCDC58	Uncharacterized protein
G1U2V6	PCBP2	Uncharacterized protein
G1SIL8	GMPR2	GMP reductase
G1T4W4	FBLN5	Uncharacterized protein
G1SQR6	CNN3	Calponin
G1SYS6	ACYP1	Acylphosphatase
G1T239	ATP6V0D1	V-type proton ATPase subunit
G1T161	ACAD10	Uncharacterized protein
G1SL68	MYH9	Uncharacterized protein
G1SEJ4	ATP6V1B2	Uncharacterized protein
G1TES6	HSD17B10	Uncharacterized protein
G1STF9	EIF3I	Eukaryotic translation initiation factor 3 subunit I
G1TCE2	CYB5R1	NADH-cytochrome b5 reductase
G1T5Y1	MAPK1	Mitogen-activated protein kinase
G1TMV1	DSTN	Uncharacterized protein
G1TYW1	TPD52L2	TPD52 like 2
P55057	APOC4	Apolipoprotein C-IV
G1SMS3	ACTR2	Actin-related protein 2
G1SDG8	NOLC1	Uncharacterized protein
G1TQP4	HSPA12A	Uncharacterized protein

P29294	MYLK	Myosin light chain kinase, smooth muscle
G1SF36	NNT	Uncharacterized protein
G1T3V5	TMPO	Uncharacterized protein
P30801	S100A6	Protein S100-A6
G1TDK0	MYL6	Uncharacterized protein
G1TCR0	CRYM	Uncharacterized protein
G1SIW8	CRYZ	Uncharacterized protein
G1U5Z3	MARS2	Uncharacterized protein
G1TEW4	USP9X	Uncharacterized protein
G1T3G8	TSFM	Elongation factor Ts, mitochondrial
G1TM00	VPS26A	Uncharacterized protein
G1SX80	LAMA2	Uncharacterized protein
G1TZ38	ANK1	Ankyrin 1
G1TQR2	EEF1B2	Uncharacterized protein
G1U146	MCAM	Melanoma cell adhesion molecule
G1SDV3	BDH2	Uncharacterized protein
G1U328		Uncharacterized protein
G1SQL0	PSMC1	Uncharacterized protein
O18757	SLC25A24	Calcium-binding mitochondrial carrier protein SCaMC-1
G1T026	PSMD10	Uncharacterized protein
Q8WN94	DBI	Acyl-CoA-binding protein
G1TZP0	YWHAG	Uncharacterized protein
G1SXT1	AHCYL1	Adenosylhomocysteinase
G1SDL3	NAGLU	Uncharacterized protein
G1TC61	LOC100359337	Uncharacterized protein
G1SIB0	LMAN2	Uncharacterized protein
G1U466	TUBA1A	Tubulin alpha chain
G1U8X8		Uncharacterized protein
G1T3Z2	NSFL1C	Uncharacterized protein
G1TD13	B2M	Beta-2-microglobulin
P41035	EIF2S2	Eukaryotic translation initiation factor 2 subunit 2
G1T601	CLYBL	Uncharacterized protein
G1SXA5		Uncharacterized protein
G1U487	IBA57	IBA57, iron-sulfur cluster assembly
P00919	CA2	Carbonic anhydrase 2
G1TPV7	LOC108176887	Uncharacterized protein
G1SLY0	SLC4A1	Anion exchange protein
G1SJZ9	LOC100359245	Thioredoxin
G1SHL8	STAT1	Signal transducer and activator of transcription
G1TJW3	NONO	Uncharacterized protein
G1SG55	EPB41L2	Uncharacterized protein
G1SKF7	RPL6	60S ribosomal protein L6
G1SE67	SPR	Uncharacterized protein
G1T312	ACAD8	Acyl-CoA dehydrogenase family member 8
P12337		Liver carboxylesterase 1
P06813	CAPNS1	Calpain small subunit 1
G1SNP8	TFG	Uncharacterized protein
G1U2R1	TM9SF2	Transmembrane 9 superfamily member

G1T0B4	TMED7	Uncharacterized protein
G1TVW1	PLOD3	Procollagen-lysine,2-oxoglutarate 5-dioxygenase 3
G1SN11	SPTBN1	Spectrin beta chain
G1T8M9	LRPPRC	Uncharacterized protein
G1SMJ5		Ubiquitin-fold modifier 1
G1SDU8	SARS2	Uncharacterized protein
G1SSM4	LOC100348005	Uncharacterized protein
G1U1C0	RBBP7	Uncharacterized protein
G1TL92		Uncharacterized protein
G1TGF1	PTGES3	Prostaglandin E synthase 3
G1TPV0	LOC100342098	60S ribosomal protein L13
G1TRW8	LCN2	Uncharacterized protein
G1TGV6	SLC27A3	Uncharacterized protein
G1SHK6	HP1BP3	Uncharacterized protein
G1U9T4	NME2	Nucleoside diphosphate kinase
G1SXE6	DYNC1LI1	Uncharacterized protein
G1SRM4	LYPLA1	Acyl-protein thioesterase 1
G1T1Z5	ECM1	Uncharacterized protein
G1TAQ7		Uncharacterized protein
G1SHX8	VCAN	Uncharacterized protein
G1SHH5	MFAP5	Uncharacterized protein
P29751	ACTB	Actin, cytoplasmic 1
G1SYJ6	RPL5	60S ribosomal protein L5
G1T2Z5	COL1A2	Collagen alpha-2(I) chain
G1T2X0	PLIN2	Perilipin
B7NZJ4	RA_r333_jsm669B4f	Uncharacterized protein RA_r333_jsm669B4f
G1SHX1		Uncharacterized protein
G1TBC6	ISCU	Uncharacterized protein
G1SPR5	GLB1	Uncharacterized protein
G1SWF3	DHODH	Dihydroorotate dehydrogenase (quinone), mitochondrial
G1TKC9	TGFBI	Transforming growth factor-beta-induced protein ig-h3
G1T7S1	LOC100353185	Uncharacterized protein
G1TF20	PLEKHO2	Uncharacterized protein
G1T970	ALDH4A1	Uncharacterized protein
G1TS97	YBX3	Y-box binding protein 3
B7NZQ6	GDI1	Rab GDP dissociation inhibitor
G1SYV9	TLN1	Uncharacterized protein
G1T1D9	COPS5	Uncharacterized protein
A0A140TAV7	SLC3A2	4F2 cell-surface antigen heavy chain
G1T9U5	METTL7A	Uncharacterized protein
G1T3U7	ENTPD1	Uncharacterized protein
G1SUZ7	ARSA	Uncharacterized protein
G1STH0	SF3A1	Uncharacterized protein
G1T9L4	CAMP	Uncharacterized protein
G1U9R4	APOB	Apolipoprotein B
G1TT82	PNMT	Uncharacterized protein
G1TEJ1	NECTIN2	Nectin cell adhesion molecule 2

G1SDF2	PCMT1	Protein-L-isoaspartate O-methyltransferase
G1TET2	LCP1	Uncharacterized protein
G1SL38	TWF1	Uncharacterized protein
G1TGK9	DHX9	Uncharacterized protein

Supplement 6

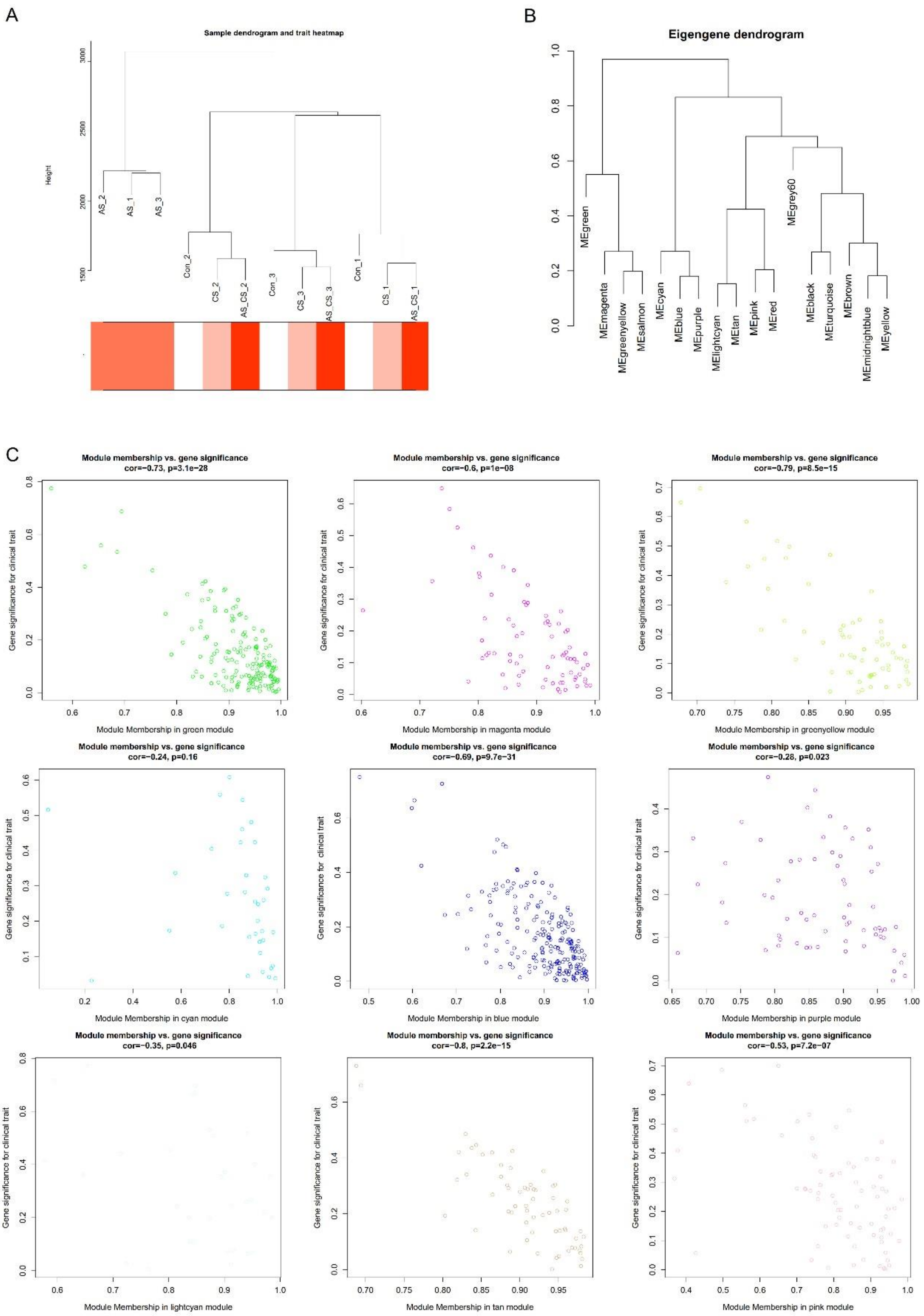

Figure S2: Weighted Co-expression Network Construction. (A) Sample dendrogram and trait heatmap. (B) The eigengene dendrogram could cluster the 17 modules. (C) The scatter diagrams show the membership of the other nine modules vs. gene

significance.

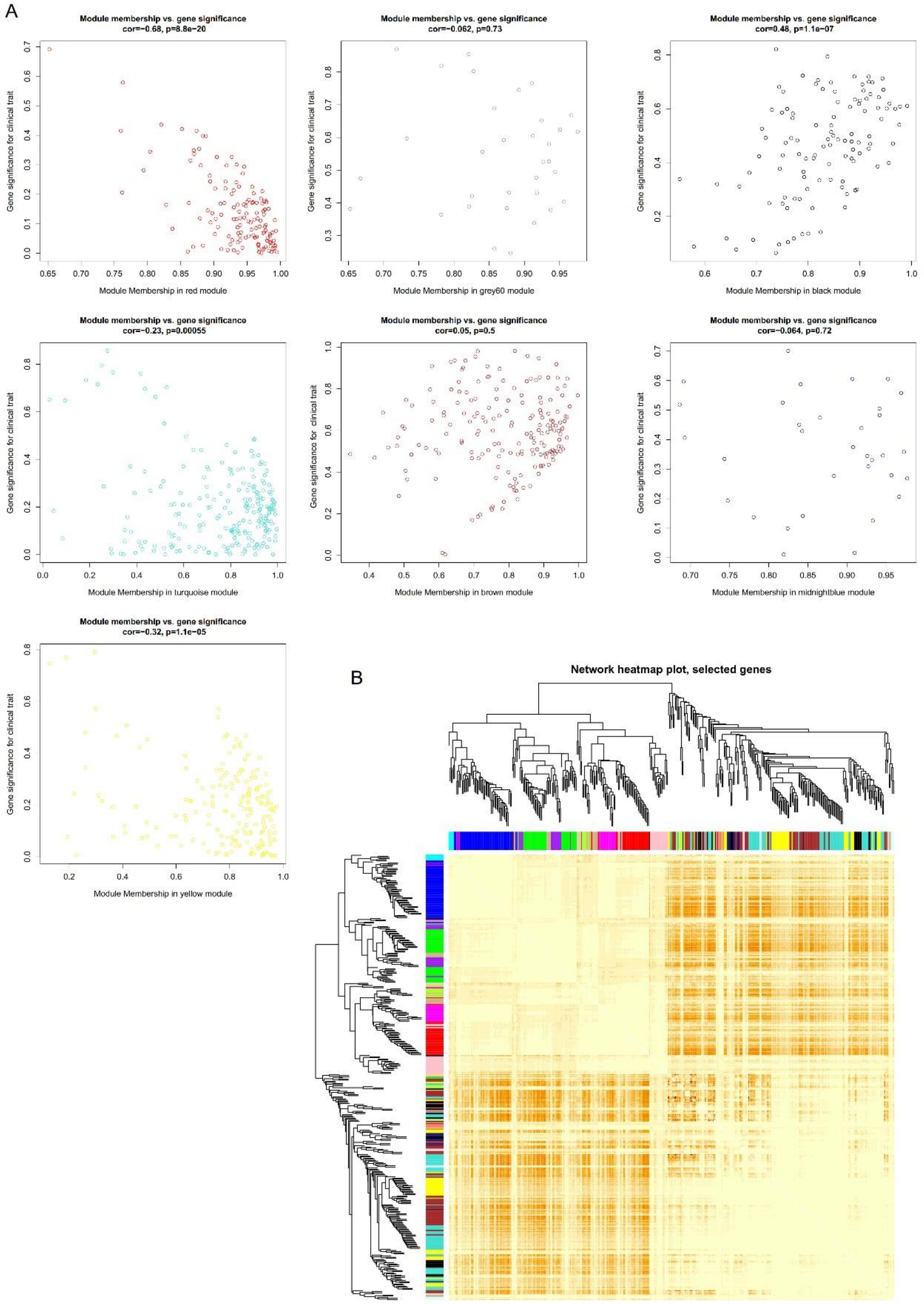

Figure S3: Module Preservation Analysis. (A) The scatter diagrams show the membership of the other seven modules vs. gene significance. (B) Interactions of the 17 co-expression modules were analyzed with the selected genes; light colors represent

high overlap and progressively darker red color indicates lower overlap. Blocks of lighter colors along the diagonal represent the co-expression modules.

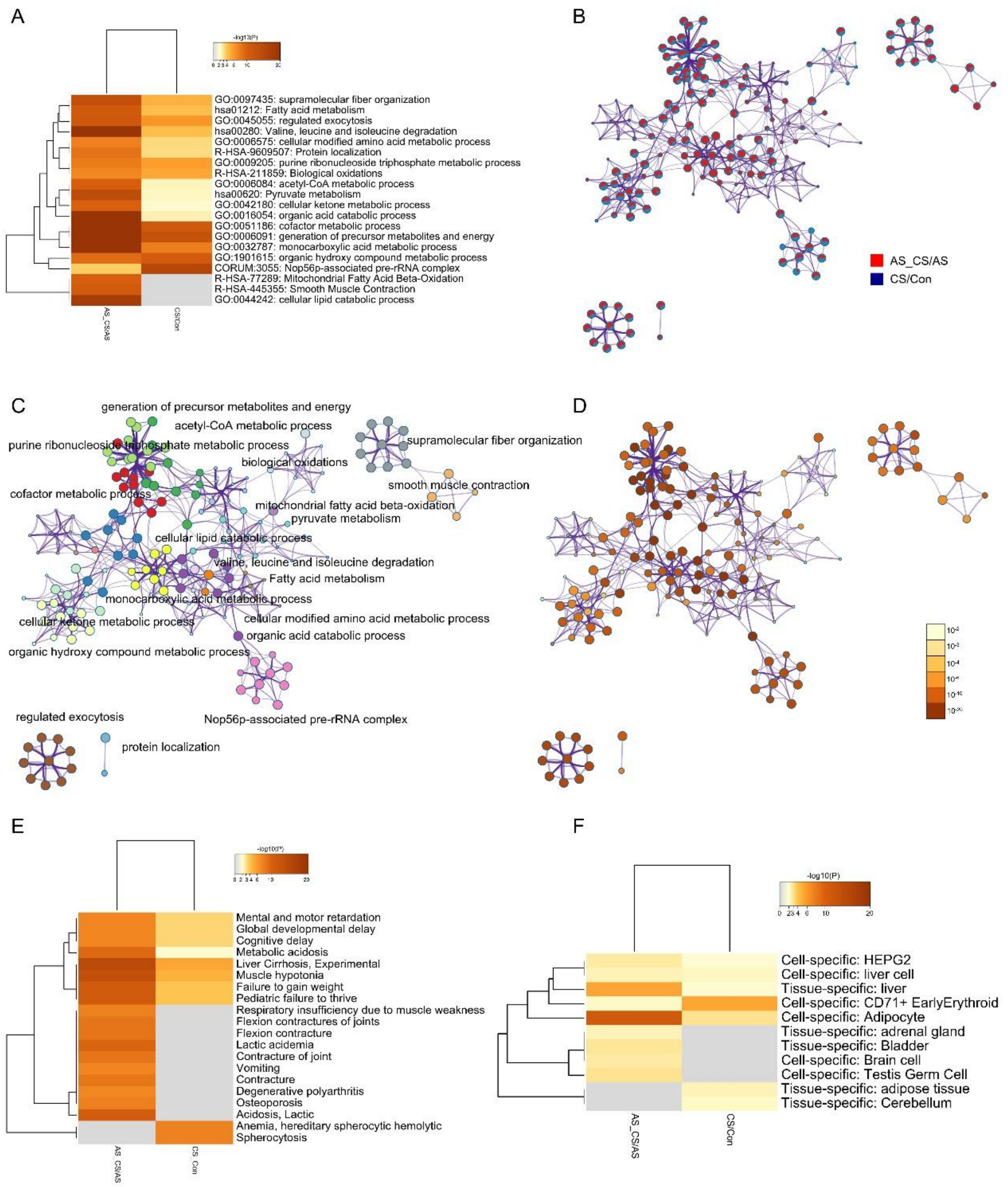

Figure S4: Enrichment analysis for the DEPs using Metascape. (A) Heatmap of enriched terms across input gene lists, colored by p-values. (B) The network of enriched terms represented as pie charts, where the charts are color-coded based on the identities of the gene lists. (C) The network of enriched terms colored by cluster ID, where nodes that share the same cluster ID are typically close to each other. (D) The network of enriched terms colored by p-values, where terms containing more genes tend to have a

more significant p-value. (E) Summary of enrichment analysis using DisGeNET. (F) Summary of enrichment analysis using PaGenBase.
